# Structure–Reactivity Studies of 2-Sulfonylpyrimidines Allow Selective Protein Arylation

**DOI:** 10.1021/acs.bioconjchem.3c00322

**Published:** 2023-09-01

**Authors:** Maëva M. Pichon, Dawid Drelinkiewicz, David Lozano, Ruxandra Moraru, Laura J. Hayward, Megan Jones, Michael A. McCoy, Samuel Allstrum-Graves, Dimitrios-Ilias Balourdas, Andreas C. Joerger, Richard J. Whitby, Stephen M. Goldup, Neil Wells, Graham J. Langley, Julie M. Herniman, Matthias G. J. Baud

**Affiliations:** † School of Chemistry, University of Southampton, Highfield, SO17 1BJ Southampton, United Kingdom; ‡ Institute of Pharmaceutical Chemistry, Johann Wolfgang Goethe University, Max-von-Laue-Str. 9, Frankfurt am Main 60438, Germany; § Structural Genomics Consortium (SGC), Buchmann Institute for Molecular Life Sciences (BMLS), Max-von-Laue-Str. 15, 60438 Frankfurt am Main, Germany

## Abstract

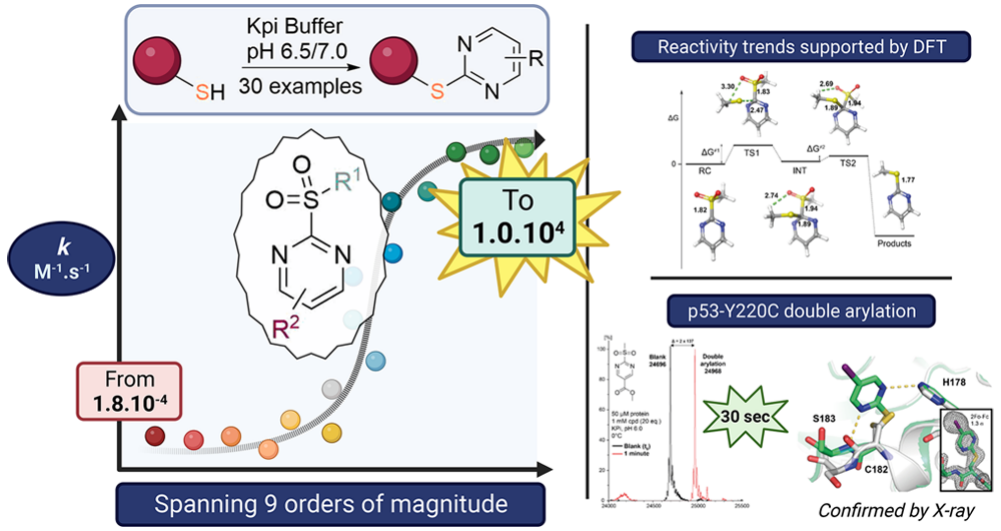

Protein arylation has attracted much attention for developing new classes of bioconjugates with improved properties. Here, we have evaluated 2-sulfonylpyrimidines as covalent warheads for the mild, chemoselective, and metal free cysteine *S*-arylation. 2-Sulfonylpyrimidines react rapidly with cysteine, resulting in stable *S*-heteroarylated adducts at neutral pH. Fine tuning the heterocyclic core and exocyclic leaving group allowed predictable S_*N*_Ar reactivity *in vitro*, covering >9 orders of magnitude. Finally, we achieved fast chemo- and regiospecific arylation of a mutant p53 protein and confirmed arylation sites by protein X-ray crystallography. Hence, we report the first example of a protein site specifically *S*-arylated with iodo-aromatic motifs. Overall, this study provides the most comprehensive structure–reactivity relationship to date on heteroaryl sulfones and highlights 2-sulfonylpyrimidine as a synthetically tractable and protein compatible covalent motif for targeting reactive cysteines, expanding the arsenal of tunable warheads for modern covalent ligand discovery.

The past two decades have seen a tremendous expansion of the range of bioconjugation strategies for preparing increasingly complex unnatural biologicals with novel properties beyond those accessible from their canonical building blocks. These strategies rely on mild and biocompatible chemical reactions, where a reactive electrophilic “warhead” creates a covalent linkage between the nucleophilic sites of the biomolecule and the designed synthetic molecule. Notable examples of such reactions include a range of condensations, ligations, nucleophilic substitution, conjugate additions and substitutions, metal/light/strain promoted “click” cycloadditions, and transition metal catalyzed couplings.^1^ These warheads have been incorporated in a myriad of chemical labeling agents such as biochemical probes for *in cellulo*/*in vivo* mechanistic studies and characterization of post-translational modifications (PTMs), tracers for bioimaging, novel biomaterials, therapeutic macromolecules with enhanced metabolic stability, and small molecule targeted covalent inhibitors (TCIs) to address biomolecular targets reputed to be intractable.^2−5^

Mild conditions for bioconjugation are paramount to retaining the structure and functionality of the biological target. Those generally require stringent conditions, including operating in aqueous buffers at a pH close to neutral and with minimal use of organic cosolvents, at temperatures ≤ 37 °C, and with minimal stirring. Critically, such reactions must be quantitative and fast, while remaining highly chemoselective, and proceed at a low substrate concentration, usually in the low micromolar range or below.^1^ Cysteine bioconjugation has, to date, received the most attention and still represents the cornerstone of most modern protein modification strategies. Cysteine is present in nearly all mammalian proteins, but represents only ca. 2% of the whole proteome^6^ and has a distinctive chemical reactivity due to the superior nucleophilicity of its thiol side chain. These two features are key advantages for the development of chemoselective bioconjugation strategies.

Balancing the reactivity of the warhead is of prime importance^3,7^ to allow covalent modification and minimize unspecific reactions at off-target sites at the protein surface, or inactivation through hydrolysis. Many cysteine reactive warheads have been reported, with maleimides, acrylamides, and related conjugated acceptors being the most popular (Figure 1). However, although they are being employed extensively, they have certain limitations. Their variable chemoselectivity,^8−10^ in addition to linker cleavage via retro-Michael,^11−14^ thiol exchange,^11,12,15−17^ hydrolysis,^16,18^ or aminolysis,^19^ are well-known historical bottlenecks,^8^ leading to variable *in vivo* efficacy and toxicity due to the formation of dynamic heterogeneous mixtures of conjugates.^20^

**Figure 1:**
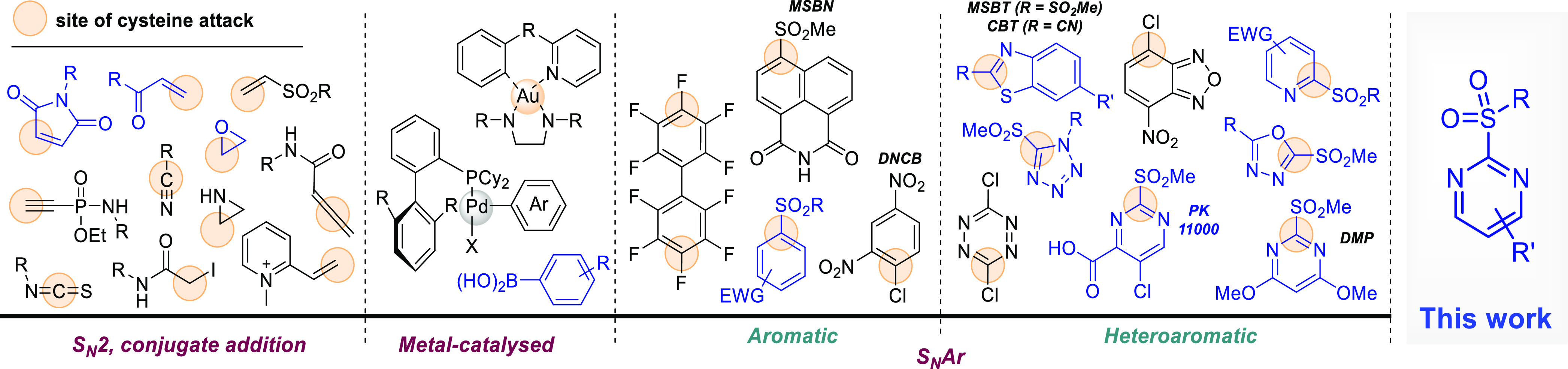
Representative classes of electrophilic warheads used for cysteine modification, extracted from the literature. The site of cysteine attack is highlighted with a beige circle. Compounds tested in our study for reactivity and stability are depicted in blue.

Heteroaryl sulfones have recently emerged as excellent reagents for the metal-free arylation of cysteine. The first example of such an agent is 4,6-dimethoxy-2-(methylsulfonyl)pyrimidine (DMP), which was reported in 2005 as a cysteine “capping” agent for proteomics studies. In 2016, prototypical lead 2-methylsulfonyl pyrimidine PK11000 (Figure 1) was reported as stabilizer of several thermolabile p53 cancer mutants *in vitro* along with thiol (e.g. GSH) depletion, accumulation of reactive oxygen species (ROS), and toxicity in p53 compromised cancer cell lines.^21^ Few such ≪thio-click≫ reagents based on benzothiazole, tetrazole, and oxadiazole scaffolds were reported by Xian et al.^22^ and Barbas et al.^16^ and others.^23−26^ These reagents show preferential selectivity for cysteine over other amino acids, and unlike maleimides, they do not react with sulfenic acids (−SOH) and *S*-nitrosothiol (−SNO).^27,28^ Importantly, the resulting thioether linked conjugates are markedly more stable than adducts of conjugate acceptors.^12,13^ Heteroaryl sulfones also display diverse reaction rates toward cysteine, modulated by the nature and electrophilicity of the heterocyclic system. This was underscored by reports from the Bollong,^29^ Martin,^30^ and Fang groups,^31^ showing that scaffolds such as 2-methylsulfonylbenzothiazole (MSBT) and naphtalimide (MSBN) carrying electron withdrawing/donating groups (EWGs/EDGs) exhibit diverse reaction rates with biological thiols.

The limited structure–reactivity relationship data for heteroarylsulfonyl make it challenging to design new synthetic reagents displaying optimal stability and reactivity profiles under physiologically relevant conditions. A better understanding of the structure–reactivity relationship of heteroarylsulfones will not only be pivotal to rationalize their bioactivity profile but will also be critical for developing tunable covalent warheads with suitable electrophilicity, aqueous stability, while maintaining chemoselectivity.

Herein, we describe the first systematic structure–reactivity relationship study of 2-sulfonylpyrimidine (2-SP) based reagents along with straightforward and scalable synthetic routes for their preparation. We show that 2-SPs and their analogues display good aqueous stability and solubility (mM, *vide infra*) compared with more hydrophobic activated benzenes and MSBT derivatives (<50 μM) requiring organic cosolvents (up to 20% MeCN).^30^ Through the systematic UV- and NMR-based determination of *in vitro* reaction rate constants with l-glutathione (GSH), we highlight the exquisite chemoselectivity of 2-SPs and show that reactivity (*k*) can be modulated over 9 orders of magnitude (i.e., a billion-fold) by precise substitution/functionalization of the heteroaromatic ring and exocyclic group. We provide general design principles for the controlled reactivity modulation of 2-SPs, supported by density functional theory (DFT) calculations. In full protein arylation experiments, we achieved fast and chemoselective cysteine arylation under benign buffered conditions. Finally, we could demonstrate regioselective arylation of the model 25 kDa DNA binding domain of p53 using mass spectrometry and X-ray crystallography and conservation of protein stability using differential scanning fluorimetry. Last but not least, 5-NO_2_-MSBT previously reported by Martin and co-workers in 2020 is the fastest reacting cysteine arylator known to date. Here, we found that 2-SP derivatives are 10^2^ to 10^3^ times more soluble than 5-NO_2_-MSBT in an aqueous buffer and identified an ester functionalized derivative which reacts 1 order of magnitude faster than 5-NO_2_-MSBT, without detriment to molecular properties and chemoselectivity.

## Results

### Structure–Reactivity Studies: Reactivity of 2-SPs Can Be Effectively Modulated beyond 9 Orders of Magnitude

**Figure 2:**
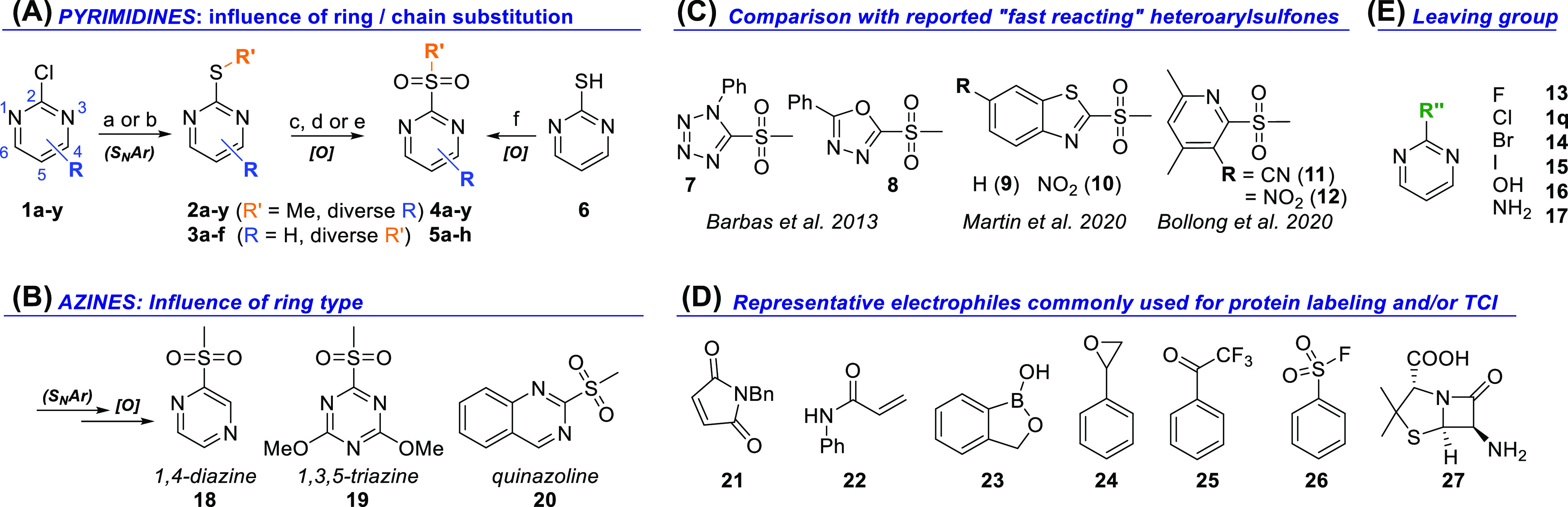
Assembly of electrophilic warhead library for structure/reactivity studies. General structures of R/R′-functionalized 2-SP (A) and azine (B) and representative synthetic routes for their preparation: (a) R′-SNa, THF, 0 °C to rt, 15–24 h; (b) R′-SH, K_2_CO_3_, THF, 0 °C to rt, 15–24 h; (c) *m*-CPBA, DCM, rt, 16 h to 4 d; (d) 30% w/w aq. H_2_O_2_, AcOH, rt, 16–24 h; (e) (NH_4_)Mo_7_O_24_·4H_2_O, 30% w/w aq. H_2_O_2_, EtOH, 0 °C to rt, 24–48 h; (f; i) aq. HCl, NaOCl, DCM, −20 to −5 °C, 30 min; (ii) BnNH_2_ or C_6_F_5_OH/Et_3_N, DCM, −20 °C to rt, 2.5 h. (C–E) Common literature electrophiles used in protein bioconjugation, for benchmarking against 2-SPs.

We assembled a library of over 40 2-SP derivatives, through both synthesis and commercial sources (Figure 2). A summary of all compound structures and associated numbering, detailed synthetic protocols, and analytical characterization data can be found in the Supporting Information. We anticipated that modulating the reactivity could be achieved by (i) introducing EWGs (e.g., −CF_3_, −NO_2_) and EDGs (e.g., −NH_2_, −OMe) on the pyrimidine ring (R) to respectively accelerate or slow down reaction rates (Figure 2A). The generation of isomeric “matched pairs” also allowed determining whether substitution at the 4- or 5-position has the strongest effect. The modulation of the reactivity could also be achieved by (ii) adjusting the sterics and electronics of the leaving group (R′; Figure 2A and E) and (iii) varying the heteroaromatic system (e.g., quinazoline), heteroatom position (e.g., pyrazine), and number of heteroatoms (e.g., triazine; Figure 2B). We also synthesized or purchased several heterocyclic sulfones recently reported (Figure 2C) and representative electrophiles (Figure 2D) from diverse classes commonly used for bioconjugation.

**Figure 3:**
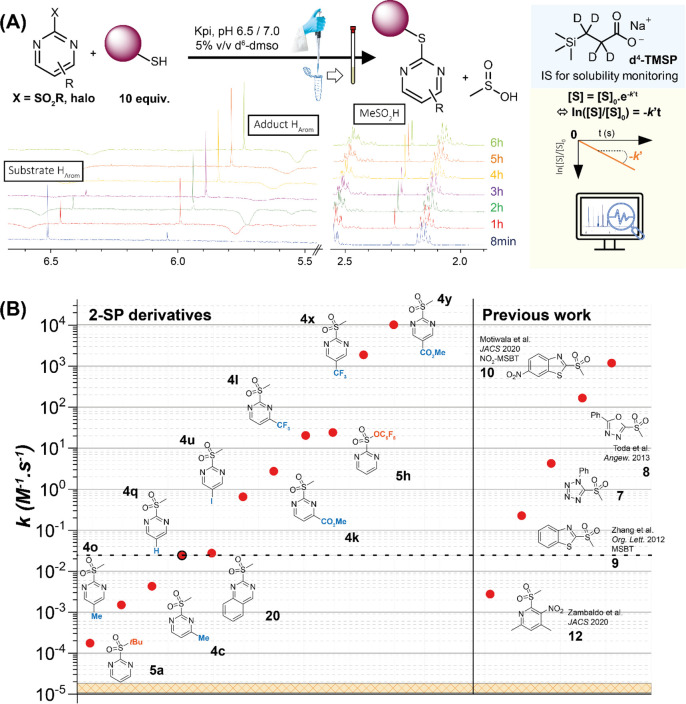
In vitro determination of electrophilic reactivity of compounds. (A) NMR assay setup for warhead/GSH reaction rate constant determination and chemoselectivity monitoring, over a 6 h time scale. Purple bead = GSH. Hydrolytic stability was determined in the same way over 36 h. d^4^-Trimethylsilylpropanoate (TMSP, blue box) was used as an internal standard for monitoring the warhead solubility. Second-order reaction rate constants (*k*) were obtained from their pseudo-first-order counterparts (*k*′), by time-dependent normalized integration of the disappearing warhead signals. An example NMR stack for the representative reaction of 4,6-dimethoxy-2-methylsulfonylpyrimidine with GSH shows time-dependent signal evolution toward quantitative formation of arylated GSH and generation of methanesulfinic acid (2.3 ppm). (B) Experimental second-order rate constants (*y* axis, log_10_ scale) for the reaction of representative 2-SP derivatives (left, 11 out of ca. 40 examples) and previously reported heteroarylsulfones (right) with GSH in a KPi buffer, pH 7.0, 20 °C. All rate constants were calculated as an average of at least two independent measurements. Numerical values and standard deviations at both pH 7.0 and 6.5, along with a full list of 2-SPs and other electrophile types tested, are presented in Tables S2–S6 and Figure S4. The horizontal dashed line marks the reaction rate of 2-methylsulfonylpyrimidine at pH 7.0, as a reference when comparing with other reagents (see main text).

We employed nuclear magnetic resonance (NMR) and UV–vis to quantify the reactivity of our 2-SP derivatives and other representative electrophiles (Figure 2) against cysteine. NMR allowed straightforward determination of reaction rate constants through dual monitoring of the consumption of the warhead and formation of the product by integration of their respective NMR signals (Figures S1–S3). It simultaneously provided a direct readout on reaction specificity and hydrolytic stability of the warhead. All measurements were carried out in KPi buffer in the presence of 5% d^6^-dmso, which is generally well-tolerated by a wide range of proteins in *in vitro* studies. *N*-acetylcysteine methylester (NACME) and l-glutathione (GSH) are useful model cysteine nucleophiles for *in vitro* studies of electrophilic agents.^32^ Mixing reference electrophile 2-methylsulfonylpyrimidine (**4q**) with NACME or GSH in a 1:10 ratio (pseudo-first-order conditions) allowed extraction of accurate and reproducible second order rate constants (*k*). At pH 7.0, quantitative arylation of NACME occurred within minutes (*k* ≈ 4.5 × 10^–2^ M^–1^ s^–1^), whereas GSH reacted approximately 3 times slower (*k* ≈ 1.6 × 10^–2^ M^–1^ s^–1^; Figures S1–S3). This was unambiguously confirmed by rapid and characteristic formation of methanesulfinic acid (δ ≈ 2.3 ppm) in each case (Figure 3A). This was also consistent with shielding of the pyrimidine aromatic protons from 9.1 (H4,6) to 7.9 (H5) ppm in **4q** to ∼8.6 (H4,6) to 7.3 (H5) ppm in the arylated NACME and GSH products, characteristic of the 2-alkylthioether motif (Figures S1B and S2B). Not unexpectedly, the reaction was approximately 5 times faster at pH 7.0 vs 6.5 with both nucleophiles, overall consistent with a higher effective equilibrium concentration of the thiolate anion. Critically, arylation was completely chemoselective and produced *S*-arylated NACME and GSH as the sole products. The p*K*_a_’s of the sulfhydryl groups in NACME and GSH are 8.3 and 9.2, respectively,^33^ explaining the greater nucleophilic reactivity of NACME compared to GSH due to higher thiolate anion concentration, translating into higher rate constants.

We selected GSH for a further structure–reactivity study as (i) the time scale on which arylation takes place is suitable for NMR studies, which is preferable for characterizing faster reacting analogues; (ii) cysteine is embedded within the GSH tripeptide, which provides a better reflection of the natural steric and electronic constraints of cysteine residues exposed at protein surfaces; (iii) the presence of the peptidic backbone and unprotected, free *N*/*C*-termini allows for a primary assessment of 2-SP reagents chemoselectivity toward thiols and potential off-target reactivity.

To establish an accurate structure–reactivity profile of 2-SP reagents, we systematically determined their reaction rate constants for the arylation of GSH by NMR, at pH 7.0 and 6.5. This was a prerequisite to (i) quantify the influence of substitution on reaction rates; (ii) accurately assess the tunability and chemoselectivity of 2-SPs for cysteine arylation; (iii) determine the influence of pH on the reaction rates to further inform on the precise reaction mechanism; and (iv) benchmark the overall performance of 2-SPs with those of previously reported fast reacting heteroarylsulfones and other common cysteine reactive warheads (Figure 2C,D). NMR allowed us to probe a dynamic range of approximately 10^4^, with rate constants (*k*) ranging from ∼5.0 × 10^–5^ M^–1^ s^–1^ to 0.5 M^–1^ s^–1^. The chemoselectivity of faster reacting warheads (*k* > 0.5 M^–1^ s^–1^, *t*_100%_ < 8 min) was also assessed by NMR, although their associated rate constants were determined by time-dependent UV absorbance, reducing the delay between mixing and data acquisition (seconds vs minutes). A representative subset of warheads was characterized in both NMR and UV-absorbance assays, and rate constants determined by both methods were generally in good agreement (Table S2). Rate constants (*k*) were determined in duplicate, at both pH 7.0 and pH 6.5. In line with our previous observation, virtually all 2-SPs reacted faster at pH 7.0, consistent with a higher equilibrium concentration of thiolate anions in solution. In comparison, the corresponding 2-chloro and 2-methylthio pyrimidines were far less reactive to completely unreactive under the same conditions. All rate constants (*k*) are summarized in Figure S4 and Tables S2–S6, and a representative set is presented in Figure 3B.

Substitution at position 5 had the most important effect on reactivity. As anticipated, strong mesomeric acceptor (−M) EWGs such as −NO_2_ and −COOMe and inductive acceptor (−I) groups such as −CF_3_ drastically increased the reaction rate by ca. 3.5 to 6 orders of magnitude compared with the unsubstituted reference warhead (**4q**, *k*_7.0/H_ ≈ 1.2 × 10^–2^ M^–1^ s^–1^) at both pH’s. In particular, the 5-COOMe derivative **4y** (*k*_7.0/COOMe_ ≈ 9900 M^–1^ s^–1^) was the most reactive warhead and was >800 000 times more reactive than **4q**. In contrast, strong + M EDGs such as -NH_2_ (**4m**) and -OMe (**4n**) completely switched off reactivity. GSH arylation could not be detected (*k* < 5.0 × 10^–5^ M^–1^ s^–1^) even after extended reaction times of 6 h. Weaker ±I/M representative groups such as −Me, −Ph, −Br, and −Cl allowed finer reactivity adjustment within approximately 1 order of magnitude. Substitution at the 4-position modulated reactivity in a less pronounced but similar manner, with strong −I/M EWG functionalized derivatives reacting faster. Trifluoromethylated derivative **4l** (*k*_7.0/CF3_ ≈ 21 M^–1^ s^–1^) was the fastest reagent of the 4- series, approximately 1750 times more reactive than **4q**. Modification of the exocyclic leaving group offered additional entry points for controlling reactivity. Fine reactivity moderation could be achieved by both increasing the steric constraint around C2 using larger alkyl chains such as *n*-Bu or *t*-Bu and reducing C2 electrophilicity by replacing the sulfone by a sulfonamide (Figure 3B, Table S3). Pleasingly, trifluoromethylation or introduction of electron-deficient aromatic systems resulted in up to 1000-fold rate acceleration while retaining complete specificity. In control experiments, 2-halo, 2-methylthio-, 2-hydroxy, and 2-amino pyrimidines (**1q** and **13**–**17**) all failed to induce observable arylation of GSH under the same conditions over 6 h, further highlighting the superior reactivity of sulfonyl based leaving groups across the pyrimidine series (Table S4). Finally, alteration of the aromatic system had a profound effect on the reactivity (Table S5). Replacement of the pyrimidine ring by a 1,4-pyrazine (**18**) completely switched off reactivity, while quinazoline analogue **20** was marginally faster reacting (*k*_7.0/quinaz_ ≈ 2.8 × 10^–2^ M^–1^ s^–1^) than reference pyrimidine **4q**. In contrast, replacement of the pyrimidine ring with a 1,3,5-triazine resulted in a drastically increased reactivity. We could access milligram quantities of purified 2,4-dimethoxy-6-(methylsulfonyl)-1,3,5-triazine **19** following anhydrous flash column chromatography. However, **19** could not be assayed due to its rapid hydrolysis in aqueous buffers or by trace/ambient moisture, even upon storage at 4 °C in the solid state. This evidences the fundamental importance of the ring type as a basis for reactivity modulation. Overall, our data highlight yet another opportunity to switch the scaffold while maintaining reactivity in a suitable range through a judicious combination of the heterocyclic system and leaving group.

### Benchmarking Experiments

We directly compared a set of diverse historical Cys reactive warheads with 2-SPs. Strikingly, none of representative acrylamide **22**, boronate **23**, epoxide **24**, electrophilic ketone **25**, sulfonyl fluoride **26**, and beta-lactam **27** showed any reactivity under our assay conditions (Table S6). *N*-benzylmaleimide **21** (*k*_7.0/NBM_ > 0.5 M^–1^ s^–1^) fully reacted within minutes but produced a heterogeneous mixture of succinimidyl products. Among reported arylating agents, electrophiles 4,6-dimethyl-2-(methylsulfonyl)nicotinonitrile **11** and 4,6-dimethyl-2-(methylsulfonyl)-3-nitropyridine **12**, recently reported by Bollong et al.,^29^ were ca. 5 times less reactive than **4q**. 2-Methysulfonylbenzothiazole **9** (MSBT, *k*_7.0/MSBT_ ≈ 0.23 M^–1^ s^–1^) was ca. 20 times more reactive than **4q** while maintaining specificity. Such selectivity was conserved across faster reacting 2-(methylsulfonyl)-6-nitrobenzo[d]thiazole **10** (*k*_7.0/NO2-MSBT_ ≈ 1200 M^–1^ s^–1^), 1-phenyl 5-methylsulfonyl tetrazole **7** (*k*_7.0/TET_ ≈ 4.3 M^–1^ s^–1^), and 2-methylsulfonyl-1,3,4-oxadiazole 5-phenyl **8** (*k*_7.0/oxdiaz_ ≈ 160 M^–1^ s^–1^), with complete reaction with GSH within minutes.

### Molecular Properties

In additional control experiments, we further underscored the chemoselectivity of the 2-SP scaffold by mixing 2 mM **4q** in a KPi buffer with 10 equiv of either lysine, tyrosine, proline, or serine. We did not observe any reaction at room temperature and a pH as high as 8.2, even after up to 6 h. In all NMR experiments, we added a fixed concentration of 3-(trimethylsilyl)propionic-2,2,3,3-d_4_ acid sodium salt (TMSP) as a water-soluble internal standard to assess both the solubility and hydrolytic stability of our reagents. With few exceptions, 2-SPs generally displayed excellent solubility at 2 mM and stability to hydrolysis in a reactivity experiment (50 mM KPi, 5% v/v d^6^-dmso) (Table S7), leading to arylated GSH as the sole product in each case. All arylated GSH conjugates remained stable and soluble at room temperature for up to 36 h. Of note, we unsurprisingly observed slow time and pH-dependent *in situ* hydrolysis of a small number of EWG-functionalized 2-SP derivatives over extended time scales. 2-SP derivatives substituted with 5-nitro (**4w**) and trifluoromethyl (**4l** and **4x**) underwent partial, slow hydrolysis toward the corresponding unreactive pyrimidin-2-ol byproducts in stability assays, i.e., in a buffer in the absence of GSH. This was unambiguously revealed by the generation of methanesulfinate and characteristic shielding (Δδ ∼ 1.0 ppm) of the pyrimidine aromatic ^1^H signals. Nonetheless, in all cases, hydrolysis generally occurred to a quantifiable extent (>5%) after several hours (Table S7) while GSH arylation was complete within seconds to a few minutes at pH 7.0 or below. Pleasingly, 2-SPs were generally soluble at millimolar concentration in KPi buffer, contrasting with fast-reacting 2-methylsulfonyl benzothiazoles **9**–**10** recently reported by the Martin lab, which required up to 20% organic cosolvent (MeCN) in PBS buffer to reach low micromolar concentrations of solubilized compounds.^30^

Density functional theory (DFT) calculations indicated that the mechanism generally proceeds in two steps and involves a stabilized Meisenheimer–Jackson complex intermediate, hence reminiscent of the “classical” model (Figure S5A). Unsurprisingly, the generally large negative Δ*G* of the overall transformation largely explains the irreversibility of the arylation. The calculated energies of activation Δ*G*_calc_^≠1^ for nucleophilic addition toward the first transition state (TS1) were significantly greater than Δ*G*_calc_^≠2^, supporting Δ*G*^≠1^ and Meisenheimer complex formation as the RDS of the reaction. Calculations also highlighted a significantly lower activation energy for the attack of 2-sulfonylated compounds compared to their 2-halo counterparts (Table S8), in line with experimental results. With a few outliers, calculated differences in activation energies (ΔΔ_calc_) of the diverse 2-SPs relative to reference 2-methylsulfonylpyrimidine (**4q**) were generally in good agreement with their experimental counterparts (ΔΔ*G*_exp_^≠1^; Figure S5B–D, Table S8). They also correlated similarly well with Hammett σ parameters (Table S8 and Figure S6). Pleasingly, our DFT model predicted the effect of side chain functionalization on reactivity quite reliably, providing a valuable tool for future reagent design (Figure S5B). We advise relying on DFT estimations for these systems in the future. It is more general and allows treating substituents not covered by Hammett parameters, such as leaving groups at the 2-position, and combinations of substituents at positions 4–6 of the pyrimidine ring.

### Application to Protein Cysteine Arylation

We characterized the covalent reactivity and chemoselectivity of representative 2-SPs in protein arylation experiments by electrospray ionization (ESI) mass spectrometry and X-ray crystallography. The cancer associated mutant p53-Y220C^34^ is a particularly well-suited test case. *1. Chemo- and regioselectivity*: In the unmodified protein, cysteine residues C182/C229/C275/C277 are solvent exposed and freely accessible, while C124/135/141/176/238/242 are sterically hindered and/or involved in structural Zn(II) coordination. The cancer specific C220 lies at the bottom of a mutationally induced hydrophobic pocket at the surface of the p53 DNA-binding domain (DBD, 25 kDa) and is also sterically hindered. *2. Mildness*: p53-Y220C also displays relatively low intrinsic stability and is prone to aggregation, hence making it a challenging model system to evaluate the protein compatibility of our reagents. C182 and C277 are known to be intrinsically more reactive than C229 and C275. However, achieving selective modification has proven challenging. For example, Michael acceptor APR-244-MQ, currently examined as a p53 stabilizer for anticancer therapy, reacts with up to nine cysteines *in vitro*, implying partial unfolding of the protein.^35^*3. Resolution*: Historically, the structural validation of covalent modifications of the p53 DBD by X-ray crystallography has proven notoriously difficult, partly because of side chain flexibility leading to diverse alternate conformations. We wondered whether arylation with 2-SPs may lead to conformational restriction and a better electron density at the arylation site. Further, we reasoned that large groups, such as iodine, should display unmistakable electronic density should arylation take place to any extent. In the same way, any off-target specificity and arylation of noncysteine side chains would be unambiguously identifiable under high concentration soaking conditions during protein crystallography experiments (*vide infra*).

**Figure 4:**
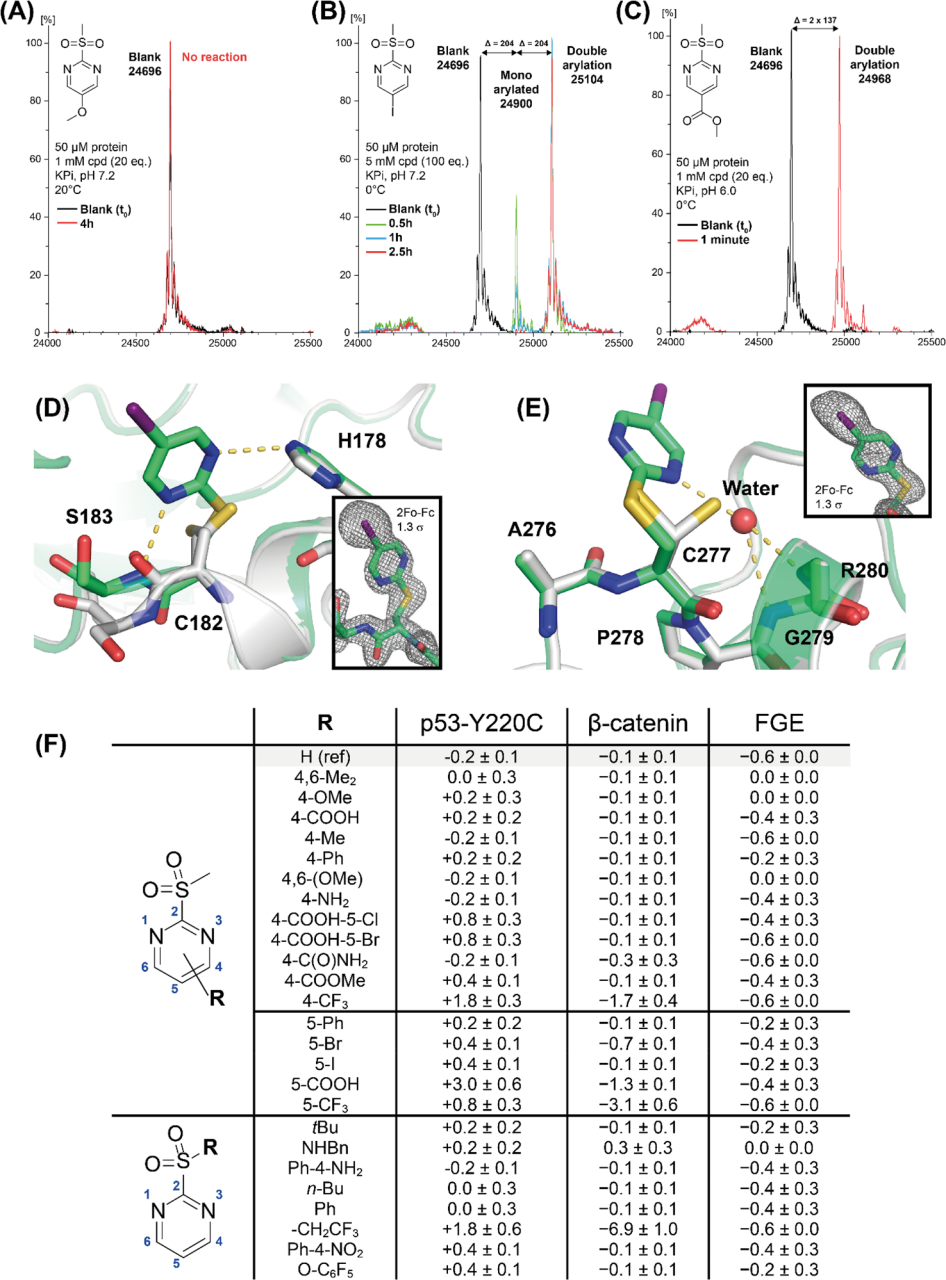
Biophysical and structural characterization of protein cysteine arylation by 2-SPs. Top: Representative deconvoluted ESI (ES+) mass spectra of 50 μM p53-Y220C incubated with arylating agents **4n** (A), **4u** (B), **4y** (C) (green/red) versus without compound (black) in KPi buffer. Stoichiometry, reaction time/temperature, and pH are indicated in each case. *X* axis: *m*/*z* (Da). *Y* axis: normalized intensities as a percentage of the most intense peak. Middle: Structure of the modified Y220C mutant (green) superimposed onto the structure of the unmodified protein (gray, PDB entry 6SHZ)^40^ showing the region around modified C182 (E) and C277 (F). Hydrogen bonds formed by the pyrimidine are highlighted as dashed yellow lines. 2*F*_o_ – *F*_c_ electron density maps are shown at a contour level of 1.3σ for segments of chain B including the modified residues C182 and C277. Bottom (F): Protein thermal stability (Δ*T*_m_, °C) of p53-Y220C, β-catenin ARD. and FGE, determined by DSF in the presence of 100 μM 2-SP derivatives.

In mass spectrometry experiments, we incubated 50 μM purified p53-Y220C DBD in KPi buffer at varying pH’s (6.0–8.0), temperatures (0 and 20 °C), and equivalents of 2-SPs (20–100). Representative 2-SPs were selected to span a broad reactivity range to determine whether trends observed in GSH *in vitro* assays translate to relative rates of modification of solvent exposed cysteines in folded proteins. Consistent with their lack of reactivity toward GSH *in vitro*, we could not detect any protein modification by representative 4,6-dimethyl- (**4a**) and 5-methoxy- (**4n**) derivatives under all conditions tested, even after 4 h at 20 °C (Figure 4A). Pleasingly, incubation with 100 equiv of 5-bromo or 5-iodo halogenated derivatives **4t** and **4u** at 0 °C resulted in completely selective double arylation of the protein in 2.5 h at pH 7.2 (Figure 4B, Figure S7). The same could also be achieved with only 20 equiv of reagent by increasing the temperature to 20 °C, or raising the pH to 8.0. This is an important result because late-stage protein functionalization with bromo- and iodo-(hetero)aromatic motifs is challenging and inaccessible by metal-catalyzed arylation methodologies due to dehalogenation and/or off-target specificity. The introduction of an iodo-(hetero)aromatic motif on the protein surface facilitates drastically the crystal structure resolution with a sharp variation of density. Finally, fast reacting 5-methylester derivative **4y** reacted at staggering speed, with only 20 equiv leading to complete and clean double protein arylation in under 30 s at pH 6.0 and 0 °C (Figure 4C). Overall, we observed good correlation between GSH and protein experiments.

To validate the arylation sites, we determined the crystal structure of the p53-Y220C DBD after soaking with iodinated warhead **4u**. The 1.53 Å resolution crystal structure revealed modification of the solvent-exposed cysteines at positions 182 and 277 (Figure 4D,E), consistent with MS data. The modifications could be unambiguously modeled in both chains of the asymmetric unit, with an unmistakable density pattern for the iodoaromatic unit (Figure S8, Table S9). The pyrimidine moiety at C182 was stabilized via hydrogen bonds with the backbone amide of S183 and the imidazole group of H178, while the iodine moiety protruded into the solvent. Upon modification of C277 in the loop preceding the *C*-terminal helix, one of the two pyrimidine nitrogen atoms formed a water-mediated hydrogen bond with the backbone amides of G279 and R280. The side chains of both cysteines adopted two alternative conformations in the unmodified structure, whereas upon modification only a single conformation was observed. Conversely and despite being surface-exposed, there were no noticeable positive densities at C229 and C275, showing that subtle differences in microenvironments and reactivity can be exploited for selective targeting. C220 at the bottom of the mutation-induced surface crevice also was not modified despite its sulfur atom being accessible, presumably because the narrowness of the pocket prevents a productive geometry for the nucleophilic attack. Further it is interesting to note that none of the cysteines in the cluster of three neighboring cysteines (C124, C135, and C141) that are known to be chemically reactive^36^ was modified, suggesting that these more sterically hindered cysteines require more reactive agents or partial unfolding for modification. Pleasingly, we also did not observe any additional density at the protein surface. Overall, this is very much in line with the MS data presented in Figure 4A–C.

### Effect of 2-SPs on Protein Stability

We also probed the general effect of 2-SPs on protein stability by using differential scanning fluorimetry (DSF). We selected the p53-Y220C cancer mutant, the folded armadillo domain (ARD) of β-catenin, and the formylglycine-generating enzyme (FGE) as representative model proteins due to their structural and functional diversity. Mutants of the 25 kDa DNA-binding domain of the tumor suppressor p53 are notorious for their reduced thermal stability and denaturation/aggregation propensity.^37^ The 56 kDa armadillo domain (ARD) of β-catenin mediates canonical Wnt signaling and plays a central role in embryogenesis and tissue homeostasis.^38^ FGE (37 kDa) is the only known activator of human sulfatases, and its stability is susceptible to modification of its cysteines.^39^ We envisaged that the challenge imposed by the structural complexity and diversity, size, and known low intrinsic stabilities would offer a convincing demonstration of the general applicability and tolerability of 2-sulfonylpyrimidine reagents. In DSF experiments, all proteins retained wild-type (WT)-like stabilities and melting temperatures (*T*_m_) following incubation with excess reagents. With few exceptions, all compound-treated proteins displayed minor changes in melting temperatures (Δ*T*_m_), usually within ca. 1 °C of that of the nontreated proteins (Figure 4F).

## Discussion

Despite their reversibility and off-target reactivity, Michael acceptors and alkylating agents still form the backbones of modern bioconjugation strategies. Comparatively, protein cysteine arylation has received less attention. Here, we disclose a library of cysteine chemoselective 2-sulfonylpyrimidines whose reactivity can be finely adjusted over (at least) 9 orders of magnitude *in vitro*, providing opportunities to match reactivity to that of specific reactive cysteines. Arylation by 2-SPs is metal-free, operates under benign aqueous conditions, at neutral pH, and forms highly stable conjugates. In full protein modification experiments, we demonstrate that 2-SPs can discriminate between many cysteine residues at a protein surface to arylate the most reactive cysteines selectively without compromising protein stability. To the best of our knowledge, prototypical ester substitute (**1y**) is the fastest known cysteine arylating agent to date, surpassing previously reported nitro-MSBT (**10**) by an order of magnitude *in vitro* and retaining exquisite selectivity.

It is striking that 2-sulfonylpyrimidines and other heteroarylsulfones are often absent from covalent compound libraries for screening and rarely identified by pan assay interference (PAIN) filters, arguably due to the gap in published knowledge on their reactivity. We anticipate wide-ranging applications of 2-SPs, from the development of improved antibody–drug conjugates for selective drug delivery to new classes of fine-tuned TCIs for therapeutic applications. The latter, in particular, holds promise. Michael acceptors are still employed extensively in covalent drug development, despite their limitations. It will be interesting to see how 2-SP warheads perform against, e.g., maleimides and acrylamides in terms of inhibitory potency but also selectivity for individual members from structurally related protein families, such as kinases. The recent advances in radiosynthetic methodologies for ^18^F-trifluoromethylation of aromatics also presents interesting opportunities for developing new classes of ^18^F-labeled fast arylating agents and their application in positron emission tomography (PET).^41,42^ The range of synthetically tractable “exit vectors” protruding from the structurally minimalist motifs described in this study, combined with good aqueous solubility and adjustable reaction rates, make 2-SPs well-positioned as an optimum molecular scaffold for general application to next-generation protein bioconjugates.

## Supporting Information Available

Methods, synthetic procedures, compound characterization data, additional references, protocols for recombinant protein production, protocols for biophysical studies, X-ray crystallographic data, extended data, acknowledgments, details of author contributions, competing interests, and statements of data accessibility (PDF)
Full wwPDB X-ray Structure Validation Report (PDF)
^1^H NMR spectra (PDF)

## Accession Codes

Atomic coordinates and structure factors for the reported crystal structure have been deposited in the Protein Data Bank (PDB) under accession number 8CG7.
