# Supplementary material for: Structure–Reactivity Studies of 2-Sulfonylpyrimidines Allow Selective Protein Arylation
Source: Bioconjug Chem. 2023 Sep 1;34(9):1679–87. doi: 10.1021/acs.bioconjchem.3c00322 (PMC10515483; doi:10.1021/acs.bioconjchem.3c00322)
Supplement: Supplementary file 1 — bc3c00322_si_001.pdf [file bc3c00322_si_001.pdf]

Supporting Information

Structure-Reactivity Studies of 2-Sulfonylpyrimidines Allow Selective Protein Arylation

Maëva M. Pichon[1], Dawid Drelinkiewicz[1], David Lozano[1], Ruxandra Moraru[1], Laura J. Hayward[1], Megan Jones[1], Michael A. Mccoy[1], Samuel Allstrum-Graves[1], Dimitrios-Ilias Balourdas[2,3], Andreas C. Joerger[2,3], Richard J. Whitby[1], Stephen M. Goldup[1], Neil Wells[1], Graham J. Langley[1], Julie M. Herniman[1], Matthias G. J. Baud[1]*

[1]School of Chemistry, University of Southampton, Highfield, SO17 1BJ Southampton, UK

[2]Institute of Pharmaceutical Chemistry, Johann Wolfgang Goethe University, Max-von-Laue-Str. 9, Frankfurt am Main, 60438, Germany

[3]Structural Genomics Consortium (SGC), Buchmann Institute for Molecular Life Sciences (BMLS), Max-von-Laue-Str. 15, 60438 Frankfurt am Main, Germany

*Correspondence: m.baud@soton.ac.uk

Table of Content

Table S1. Yields summary for the synthesis of R/R' substituted 2-sulfonyl pyrimidine derivatives via the SNAr/oxidation route. 6

Figure S1 6

Figure S2. 6

Figure S3. Determination of pseudo-first order reaction k' 7

Reactivity assays: full reactivity plot summarising all measurable rate constants for GSH arylation by 2-SPs **8**

Figure S4. All experimental second order rate constants 8

Reactivity assays: tabulated rate constants **9**

Table S2. Influence of substitution (R) at the 4- and 5- positions on the reactivity of 2-methylsulfonylpyrimidines and their derivatives 9

Table S3. Influence of exocyclic S- substitution (R) on the reactivity of 2-methylsulfonylpyrimidines and their derivatives 10

Table S4. Control experiments highlighting the superior reactivity of 2-sulfonyl pyrimidines with nucleophilic thiols compared to their corresponding 2-halo, 2-hydroxy and 2-amino counterparts. 10

Table S5. Influence of the heterocyclic system on the reactivity of 2-methylsulfonylpyrimidines and their derivatives. 11

Table S6. Control experiments: benchmarking of 2-sulfonylpyrimidine against a representative set of covalent warheads commonly used for bioconjugation 12

Stability and solubility assays **13**

Table S7. Solubility and stability of 2-SPs in 50 mM KPi aqueous buffer 13

DFT calculations **14**

Table S8. 14

Figure S5. DFT calculations highlight nucleophilic addition as the rate determining step for the S_NAr reaction of 2-sulfonylpyrimidine derivatives with thiol nucleophiles. 15

Figure S6. Correlation of Arrhenius derived experimental ΔΔG≠1exp (Y-axis, kJ/mol) and Hammett parameters. 16

Protein mass spectrometry **17**

Figure S7. 17

X-ray crystallography **18**

Figure S8. 18

Table S9. X-ray data collection and refinement statistics 19

Methods **20**

Protein production 20

NMR studies: rate constant determination and hydrolytic stability. 20

Chemoselectivity 21

UV/Vis experiments: rate constant determination of fast reacting warheads 21
Computational Methods 21
Differential scanning fluorimetry (DSF). 22
Protein mass spectrometry. 22
Protein crystallography. 23
Synthetic chemistry procedures 24
General information 24
Mass Spectrometry 24
Nuclear Magnetic Resonance 24
Fourier-transform infrared (FT-IR) 24
General Procedures 25
Compounds characterisation 26
Acknowledgements 52
Author contributions 52
Competing interests 53
References 53

Warhead library assembly and synthetic routes. Nucleophilic aromatic substitution reactions between commercially available thiols and heteroaryl chlorides **1a-y** afforded the corresponding 2-sulfanyl intermediates **2a-y** and **3a-f** generally in good to excellent isolated yields (**Figure 2A, main article**). Further oxidation with *m*-CPBA or hydrogen peroxide afforded the corresponding 2-sulfonylated products **4a-y** and **5a-f**. 2,4-diazine, 1,3,5-triazine and quinazoline derivatives **18-20** were prepared in a similar manner (**Figure 2B, main article**). Oxidation of 2-mercaptopyrimidine **6** to the corresponding sulfonyl chloride and further condensation with benzylamine or pentafluorophenol afforded 2-sulfonamido and 2-sulfonate derivatives **5g-h** in a one-pot sequence, as previously reported (**Figure 2A, main article**).[1,2] Prototypical literature heteroarylsulfones (**Figure 2C, main article**),[3,4] 2-halo/hydroxy/amino pyrimidines (**Figure 2D, main article**) and representative literature electrophilic warheads commonly used for bioconjugation (**Figure 2E, main article**) were either purchased or re-synthesised for comparison ("benchmarking"). Yields and detailed synthetic procedures are provided in **Table S1** and from page 24, respectively.

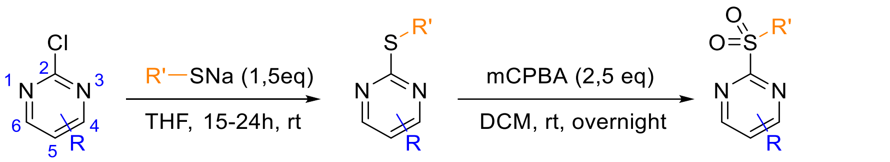

1a-y

2a-y (R' = Me, diverse R)
3a-f (R = H, diverse R')

4a-y (R' = Me, diverse R)
5a-f (R = H, diverse R')

Compound ID	R	R'	Yield S$_N$Ar (%)[a]	Yield [O] (%)[a]
a	4,6-Me$_2$	Me	n/a	n/a
b	4-OMe	Me	90	73
c	4-Me	Me	88	63
d	4-COOH	Me	87[b]	95[g]
e	4-Ph	Me	96	98
f	4,6-(OMe)$_2$	Me	n/a	n/a
g	4-NH$_2$	Me	58	29
h	4-COOH-5-Cl	Me	n/a	98[g]
i	4-COOH-5-Br	Me	n/a	n/a
j	4-C(O)NH$_2$	Me	93	99
k	4-COOMe	Me	67	85
l	4-CF$_3$	Me	75[d]	91
m	5-NH$_2$	Me	87[h]	46
n	5-OMe	Me	80	65
o	5-Me	Me	91	77
p	5-Ph	Me	98	91
q	H (***ref***)	Me	71	68
r	5-F	Me	53	86
s	5-Cl	Me	93	72
t	5-Br	Me	95	68
u	5-I	Me	61	84
v	5-COOH	Me	61	24
w	5-NO$_2$	Me	87	37
x	5-CF$_3$	Me	-	61[e]
y	5-COOMe	Me	78	78
a	H	*t*Bu	27[d]	87
b	H	*p*-NH$_2$Ph	-	80[f]
c	H	*n*Bu[c]	98	91
d	H	Ph[c]	54	67
e	H	CH$_2$CF$_3$	-	24[e]
f	H	*p*-NO$_2$Ph[c]	93[i]	77[k]

Table S1. Yields summary for the synthesis of R/R' substituted 2-sulfonyl pyrimidine derivatives via the SNAr/oxidation route.

[a]Isolated yield; [b]Reaction performed in MeOH instead of THF in the presence of 1.1 eq of K_2CO_3; [c]Corresponding thiol used instead of thiolate, in the presence of 1.5 equivalent of K_2CO_3; [d]Performed in DMF; [e]Yield over two steps; [f] Reduction from 2-SO_2(*p*-NO_2Ph) using $SnCl_2.2H_2O$ [g]Oxidised using H_2O_2, AcOH; [h]Obtained by reduction of the corresponding NO_2 using Fe, AcOH; [i]From 2-mercaptopyrimidine, S_NAr on 1-Chloro-4-nitrobenzene; [j]Obtained by reduction of the corresponding NO_2 using $SnCl_2.2H_2O$; [k]Oxidation with H_2O_2, $(NH_4)_6Mo_7O_{24}.4H_2O$; n/a commercially available.

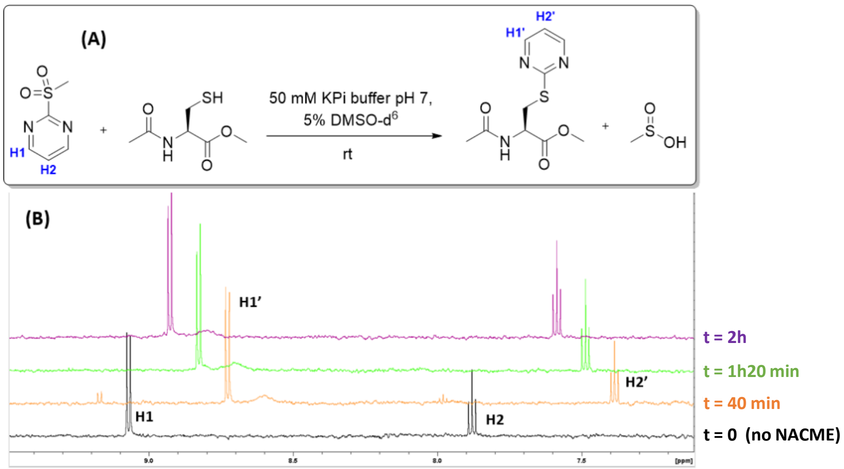

Figure S1. (A) Reaction of **4q** with NACME with pyrimidine protons assigned in both starting material and product; (B) Staggered NMR spectra of 10 equivalents NACME reacting with **4q** over 2 hours in 50 mM KPi buffer, 5% v/v DMSO-d^6, pH 7.0. Black

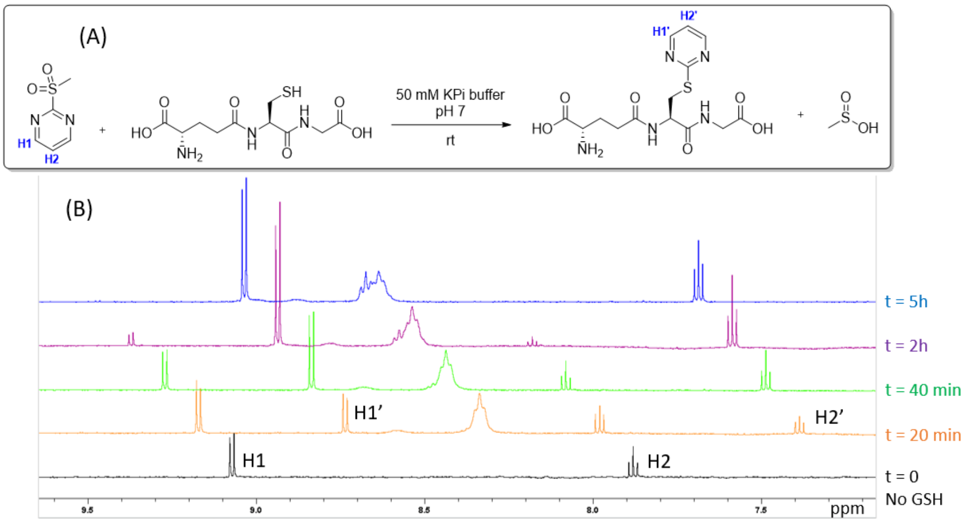

Figure S2. (A) Reaction of **4q** with GSH with pyrimidine protons assigned in both starting material and product; (B) Staggered NMR spectra of 10 equivalents GSH reacting with **4q** over 5 hours in 50 mM KPi buffer, 5% v/v DMSO-d^6, pH 7.0.

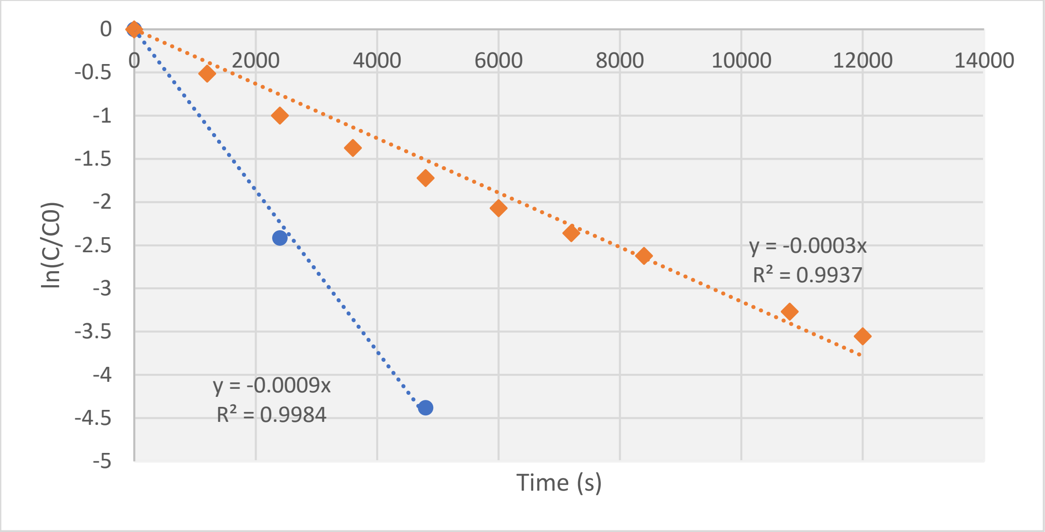

Figure S3. Determination of pseudo-first order reaction k'. ln(C/C_0) as a function of time (seconds) for the reaction of 2-methylsulfonylpyrimidine (**4q**, 2 mM) with NACME (20 mM, blue) and GSH (20 mM, orange), at pH 7.0. C: concentration of **4q** at t; C_0: concentration of **4q** at t_0.

Reactivity assays: full reactivity plot summarising all measurable rate constants for GSH arylation by 2-SPs

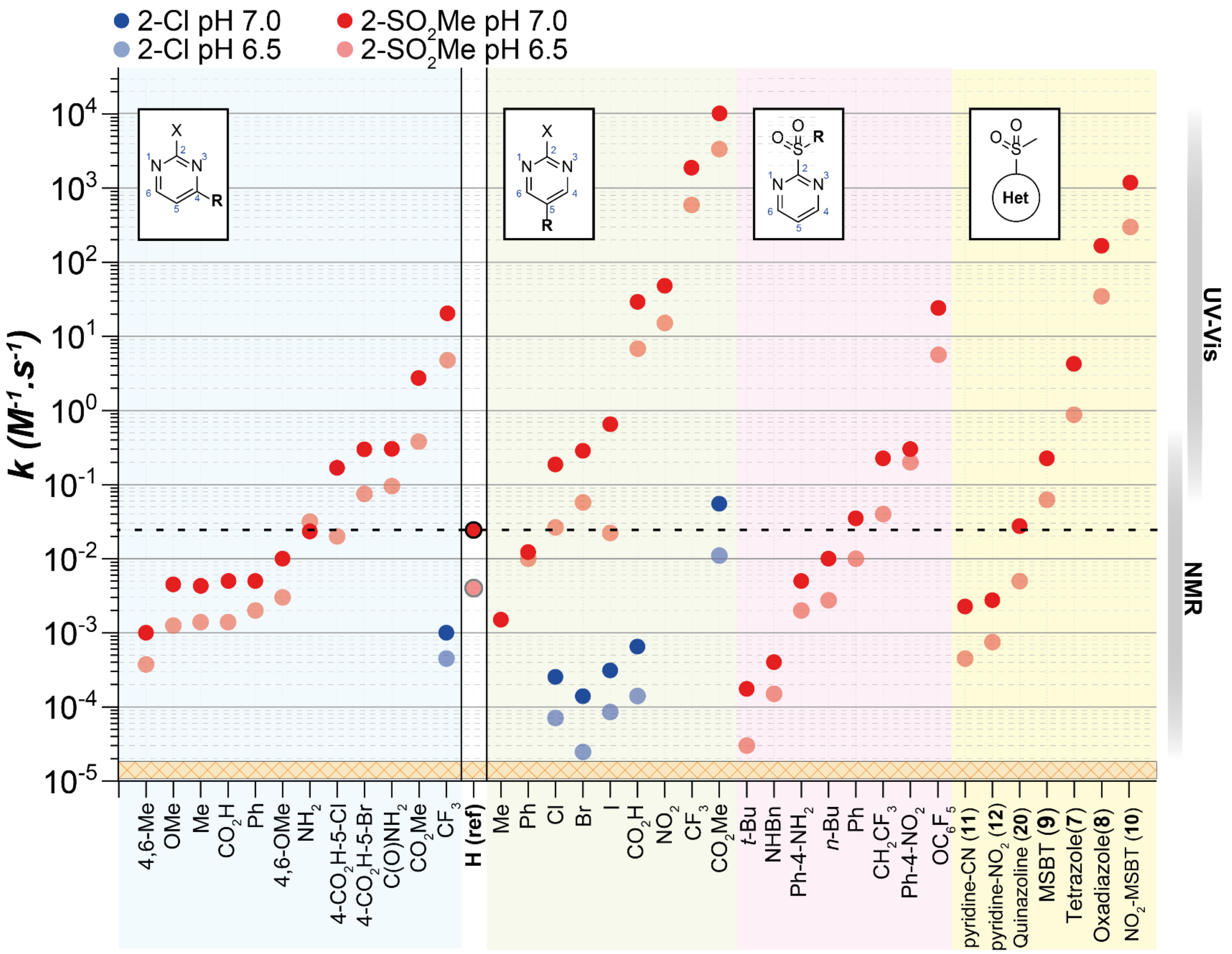

Figure S4. All experimental second order rate constants (Y-axis, $\log_{10}$ scale) for the reaction of heteroarylsulfones library (red dots) and their corresponding 2-chloro- (blue dots) derivatives (X-axis) with GSH at pH 7.0 and 6.5, determined by NMR and/or UV-Vis titrations at 20°C. 2-methythio- synthetic intermediates were all unreactive in these conditions. Dynamic ranges probed by NMR and UV-vis are shown on the right of the plot. All rate constants were calculated as an average of at least two independent measurements. Numerical values and standard deviations, along with a full list of unreactive warheads are presented in **Tables S2-6**. The horizontal dashed line marks the reaction rate of 2-methylsulfonylpyrimidine at pH 7.0, as reference when comparing with other reagents (see main text).

Reactivity assays: tabulated rate constants

**Cpd ID (1 ,2 ,4)**	**R**	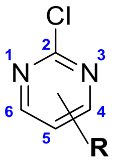		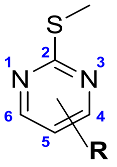		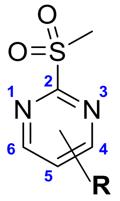		**k change (vs ref)[c]**
		pH 6.5	pH 7.0	pH 6.5	pH 7.0	pH 6.5	pH 7.0	pH 7.0
**q**	H (ref)	NR	NR	NR	NR	4.0 ± 1.6	25 ± 14	/
**a**	4,6-Me	NR	NR	NR	NR	0.38 ± 0.08	1.0 ± 0	-25
**b**	4-OMe	NR	NR	NR	NR	1.3 ± 0.8	4.5 ± 0.5	-6
**c**	4-Me	NR	NR	NR	NR	1.5 ± 0.5	4.3 ± 0.8	-6
**d**	4-COOH	NR	NR	NR	NR	1.5 ± 0	5.0 ± 0	-5
**e**	4-Ph	NR	NR	NR	NR	2.0 ± 0.5	5.0 ± 0	-5
**f**	4,6-OMe	NR	NR	NR	NR	3.0 ± 0.5	10 ± 0	-3
**g**	4-NH_2	NR	NR	NR	NR	32 ± 6	23 ± 3	≈
**h**	4-COOH-5-Cl	NR	NR	NR	NR	20 ± 0	170 ± 7.5	+7
**i**	4-COOH-5-Br	NR	NR	NR	NR	75 ± 35<br>150 ± 90[a]	300 ± 13<br>640 ± 9[a]	+12 (+26[a])
**j**	4-C(O)NH_2	NR	NR	NR	NR	95 ± 15	300 ± 2.5	+12
**k**	4-COOMe	NR	NR	NR	NR	260 ± 45<br>370 ± 63[a]	2800 ± 1100[a]	+112[a]
**l**	4-CF_3	0.45 ± 0	1.0 ± 0.5	NR	NR	4900 ± 480[a]	21000 ± 600[a]	+840[a]
**m**	5-NH_2	NR	NR	NR	NR	NR	NR	/
**n**	5-OMe	NR	NR	NR	NR	NR	NR	/
**o**	5-Me	NR	NR	NR	NR	NR	1.7 ± 1.5	-15
**p**	5-Ph	NR	NR	NR	NR	10 ± 5.0	12 ± 7.8	-2
**s**	5-Cl	Ca. 0.07	Ca. 0.26	NR	NR	27 ± 12	190 ± 110	+8
**t**	5-Br	Ca. 0.025	Ca. 0.15	NR	NR	56 ± 34	290 ± 250	+12
**u**	5-I	Ca. 0.085	Ca. 0.31	NR	NR	25 ± 10	660 ± 10	+26
**v**	5-COOH	Ca. 0.14	Ca. 0.65	NR	NR	6700 ± 3100[a]	30000 ± 3200[a]	+1,200
**w**	5-NO_2	NR	NR	0.25 ± 0.16	3.4 ± 1.6	15000 ± 400[a]	47000 ± 1000[a]	+1,880
**x**	5-CF_3	NR	NR	NR	NR	5.8 x 10^{5}<br>± 2.6 x 10^{5} [a]	1.9 x 10^{6}<br>± 8.3 x 10^{5} [a]	+76,000
**y**	5-COOMe	Ca. 11	Ca. 55	NR	Ca. 0.020	3.3 x 10^{6}<br>± 1.8 x 10^{5} [a]	9.9 x 10^{6}<br>± 2.5 x 10^{4} [a]	+396,000
**r**	5-F	NR	NR	NR	NR	Multiple[b]	Multiple[b]	/

Table S2. Influence of substitution (R) at the 4- and 5- positions on the reactivity of 2-methylsulfonylpyrimidines and their derivatives. Summary of experimental rate constants (*k*) determined by NMR for the reaction of substituted 2-chloro-, 2-methyltiho- and 2-methylsulfonyl pyrimidine derivatives with GSH at pH 6.5 and 7.0. All values are given in $mM^{-1}.s^{-1}$ and are the average of at least two repeats. Standard deviations are provided. [a] Measured by UV titrations; NR = no reaction. [b] Multiple products observed by NMR, resulting from competitive attack at the 5-position with fluoride displacement. [c] Ratio of individual rate constants to the reference rate constant associated with warhead **4q**, at pH 7.0. Given as -fold acceleration (+) or deceleration (-).

Cpd ID (3, 5)	R	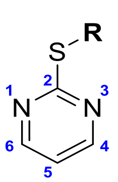	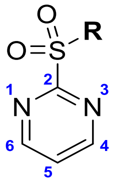		k change (vs ref)[b]
		pH 7.0	pH 6.5	pH 7.0	pH 7.0
	Me (ref)	NR	4.0 ± 1.6	25 ± 14	/
**a**	*t*Bu	NR	Ca. 0.03	0.18 ± 0.03	-139
**g**	NHBn	NR	0.15 ± 0.05	0.40 ± 0.10	-63
**b**	Ph-4-NH_2	-	2.0 ± 0	5.0 ± 0	-5
**c**	*n*-Bu	NR	2.8 ± 0.3	10 ± 0	-3
**d**	Ph	NR	10 ± 5.0	35 ± 15	≈
**e**	-CH_2CF_3	-	40 ± 5.0	230 ± 50	+9
**f**	Ph-4-NO_2	NR	200 ± 90[a]	290 ± 170[a]	+12
**h**	O-C_6F_5	NR	5400 ± 720[a]	24000 ± 1100[a]	+960

Table S3. Influence of exocyclic S- substitution (R) on the reactivity of 2-methylsulfonylpyrimidines and their derivatives. Summary of experimental rate constants (*k*) determined by NMR for the reaction of substituted 2-thio- and 2-sulfonyl pyrimidine derivatives with GSH at pH 6.5 and 7.0. All values are given in mM^{-1}.s^{-1} and are the average of at least two repeats. Standard deviations are provided. [a] Measured by UV titrations; NR = no reaction. [b] Ratio of individual rate constants to the reference rate constant associated with warhead **4q**. Given as -fold acceleration (+) or deceleration (-).

Cpd ID	R	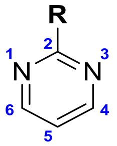
		pH 7.0
**4q**	SO_2Me (ref)	25 ± 14
**13**	F	NR
**1q**	Cl	NR
**14**	Br	NR
**15**	I	NR
**16**	OH	NR
**17**	NH_2	NR

Table S4. Control experiments highlighting the superior reactivity of 2-sulfonyl pyrimidines with nucleophilic thiols compared to their corresponding 2-halo, 2-hydroxy and 2-amino counterparts. The rate constant (*k*) for reference warhead **4q** is given in mM^{-1}.s^{-1}.

Cpd ID	Structure			k change (vs ref)[c]
		pH 6.5	pH 7.0	pH 7.0
**4q**	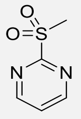 (ref)	4.0 ± 1.6	25 ± 14	/
**18**	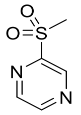	NR	NR	/
**11**	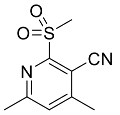	0.45 ± 0.05	2.3 ± 0.25	-11
**12**	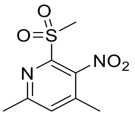	0.75 ± 0.25	2.8 ± 0.25	-9
**20**	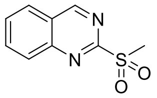	5.0 ± 0	28 ± 2.5	~
**9**	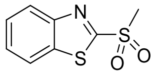	63 ± 23	230 ± 35	+9
**7**	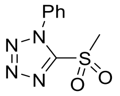	Ca. 850[a]	Ca. 4300[a]	+170
**8**	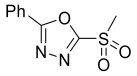	35000 ± 2000[a]	160000 ± 9200[a]	+6,400
**10**	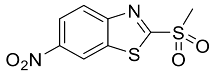	290000 ± 130000[a]	1.1×10^6 ± 540000[a]	+44,000
**19**	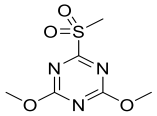	- [b]	- [b]	/

Table S5. Influence of the heterocyclic system on the reactivity of 2-methylsulfonylpyrimidines and their derivatives. Summary of experimental rate constants (*k*) determined by NMR for the reaction of diverse sulfonylated heterocyclic systems with GSH at pH 6.5 and 7.0. All values are given in $mM^{-1}.s^{-1}$ and are the average of at least two repeats. Standard deviations are provided. [a]Measured by UV titrations; NR = no reaction; [b] Not determined due to high reactivity and rapid degradation. [c] Ratio of individual rate constants to the rate constant associated with reference warhead **4q**. Given as -fold acceleration (+) or deceleration (-).

Cpd ID	Structure	
		pH 7.0
**4q**	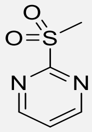 (ref)	25 ± 14
**21**	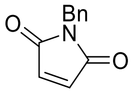	Multiple products
**22**	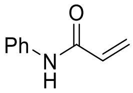	NR
**23**	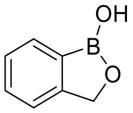	NR
**24**	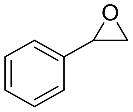	NR
**25**	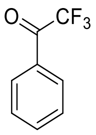	NR
**26**	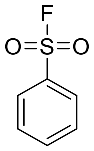	NR
**27**	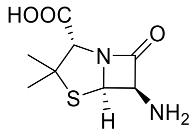	NR

Table S6. Control experiments: benchmarking of 2-sulfonylpyrimidine against a representative set of covalent warheads commonly used for bioconjugation. The rate constant (*k*) for reference warhead **4q** is given in $mM^{-1}.s^{-1}$. Standard deviation is provided. NR = no reaction.

Stability and solubility assays

R	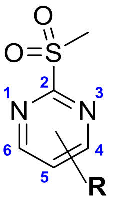		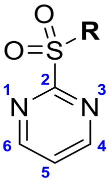		
	% degradation		% degradation		Solubility (mM) upon mixing (t_0) 50 mM KPi pH 7.0
	pH 7.0	pH 8.2	pH 7.0	pH 8.2	
Me (ref)	<5	<5			≥ 2
4,6-Me_2	<5	Nd			≥ 2
4,6-$(OMe)_2$	Nd	Nd			≥ 2
4-OMe	Nd	Nd			≥ 2
4-Me	Nd	10			≥ 2
4-NH_2	5	Nd			≥ 2
4-COOH-5-Cl	<5	Nd			≥ 2
4-COOH	<5	<5			≥ 2
4-Ph[a]	<5	50			< 2
4-COOMe[c]	77	100			≥ 2
4-CF_3[b]	17	65			≥ 2
5-Me	Nd	<5			≥ 2
5-Ph[a]	78	78			< 2
5-Br	<5	<5			≥ 2
5-Cl	Nd	7			≥ 2
5-I[b]	<5	14			≥ 2
5-NO2[b]					Ca. 0.13
5-COOMe[b]		100			≥ 2
5-COOH	18				≥ 2
5-CF3	97	100			≥ 2
-*t*Bu			<5	Nd	≥ 2
-NHBn			Nd	<5	≥ 2
-Ph-4-NH_2			Nd	Nd	≥ 2
-*n*Bu			Nd	Nd	≥ 2
-Ph			12	10	≥ 2
-CH_2CF_3[b]			100	100	≥ 2
-Ph-4-NO_2[a]			100	100	0.1
-OC_6F_5			100	100	0.1
3-CN pyridine	nd	nd			≥ 2
3-NO2 pyridine	<5	<5			≥ 2
Quinazoline	<5	<5			≥ 2
MSBT	15	<5			≥ 2
Oxadiazole	42	49			0.1
Tetrazole	<5	8			≥ 2

Table S7. Solubility and stability of 2-SPs in 50 mM KPi aqueous buffer. % degradation over 12 hours measured by NMR (sealed tube, constant volume), calculated from the difference in aromatic signal(s) integration I over time ($I_{t0} - I_{12h}$). [a]Compound crashing out of solution over time, no degradation product observed; [b]Hydrolysis to the corresponding 2-hydroxy pyrimidine; [c]Ester hydrolysis; Nd no degradation observed, remained soluble.

DFT calculations

	R	*k* ($M^{-1}.s^{-1}$)	k/k_{ref}	$\Delta\Delta G^{\neq1}_{exp}$ (kJ/mol)	$\Delta\Delta G^{\neq1}_{calc}$ (kJ/mol)	σ_m	σ_p
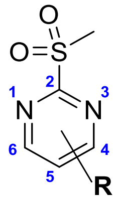	**H (ref)**	**0.0250**	**1**	**0.00**	**0.00**	**0.00**	**0.00**
	4,6-Me	0.0010	0.040	**8.0**	**4.4**		
	4-Me	0.0043	0.17	**4.4**	**0.91**	-0.07	
	4-OMe	0.0045	0.18	**4.3**	**-3.2**	0.12	
	4-COO^-	0.0050	0.20	**4.0**	**2.1**	-0.10	
	4,6-OMe	0.010	0.40	**2.3**	**-5.0**		
	4-NH_2	0.023	0.92	**0.21**	**7.5**	-0.16	
	4-COOMe	2.8	112	**-12**	**-10**	0.37	
	4-CF_3	21	840	**-17**	**-13**	0.43	
	5-Me	0.0017	0.068	**6.7**	**3.2**		-0.17
	5-Cl	0.19	7.6	**-5.0**	**-16**		0.23
	5-Br	0.29	12	**-6.1**	**-18**		0.23
	5-COO^-	30	1200	**-18**	**-3.0**		0.00
	5-CF_3	1900	76000	**-28**	**-27**		0.54
	5-COOMe	9900	396000	**-32**	**-18**		0.45
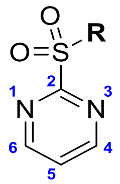	tBu	0.00020	0.0072	**12**	**9.9**		
	Ph-4-NH_2	0.0050	0.20	**4.0**	**3.8**		
	Ph	0.035	1.4	**-0.83**	**-1.2**		
	CH_2CF_3	0.23	9.2	**-5.4**	**-3.3**		
	Ph-4-NO_2	0.29	12	**-6.1**	**-10**		
	OC_6F_5	24	960	**-17**	**-18**		
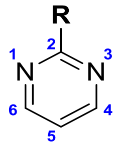	F	NR			**21**		
	Cl	NR			**32**		
	Br	NR			**31**		
	SMe	NR			**52**		
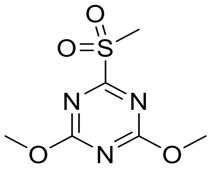		unstable			**-27**		
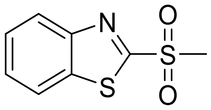		0.23	9.2	**-5.5**	**-4.8**		
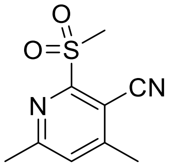		0.0028	0.11	**5.4**	**5.6**		

Table S8. Differences in activation energies relative to reference reagent 2-methylsulfonyl pyrimidine **4q**, ref). $\Delta\Delta G^{\neq1}_{exp}$ was derived from second order rate constants *k* via the Arrhenius equation ($-RT\ln(k/k_{ref})$). Sigma values taken from Hansch *et al.*[5]

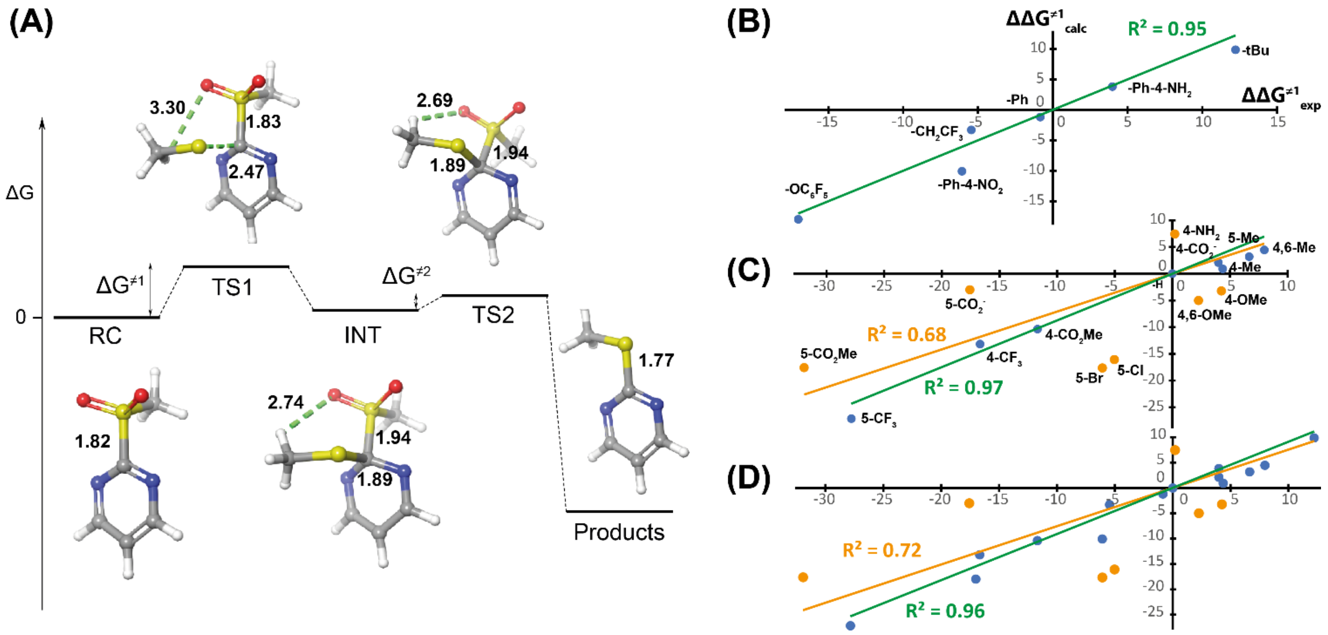

Figure S5. DFT calculations highlight nucleophilic addition as the rate determining step for the S$_N$Ar reaction of 2-sulfonylpyrimidine derivatives with thiol nucleophiles. (A) DFT calculated general Gibbs free energy profile for S$_N$Ar reactions of selected 2-halo- and 2-sulfonylpyrimidine derivatives with model methanethiolate. RC = reactants, TS1= transition state 1, INT = Meisenheimer intermediate, TS2 = transition state 2. Computed transition state structures and Meisenheimer intermediate for the reaction of 2-methylsulfonylpyrimidine with methanethiolate are shown; Correlation of Arrhenius derived experimental $\Delta\Delta G^{\neq 1}_{exp}$ (X-axis, kJ/mol) and DFT calculated $\Delta\Delta G^{\neq 1}_{calc}$ (Y-axis, kJ/mol) for varying leaving group **(B)**, C4/5/6-substitution **(C)**, and all derivatives **(D)**. Level of theory: ωB97XD/6-31+G(d,p) / SMD (water). Outliers are shown in orange. Best fit from linear regression and coefficient of determination (R^2) are shown with inclusion (orange) or exclusion (green) of outliers.

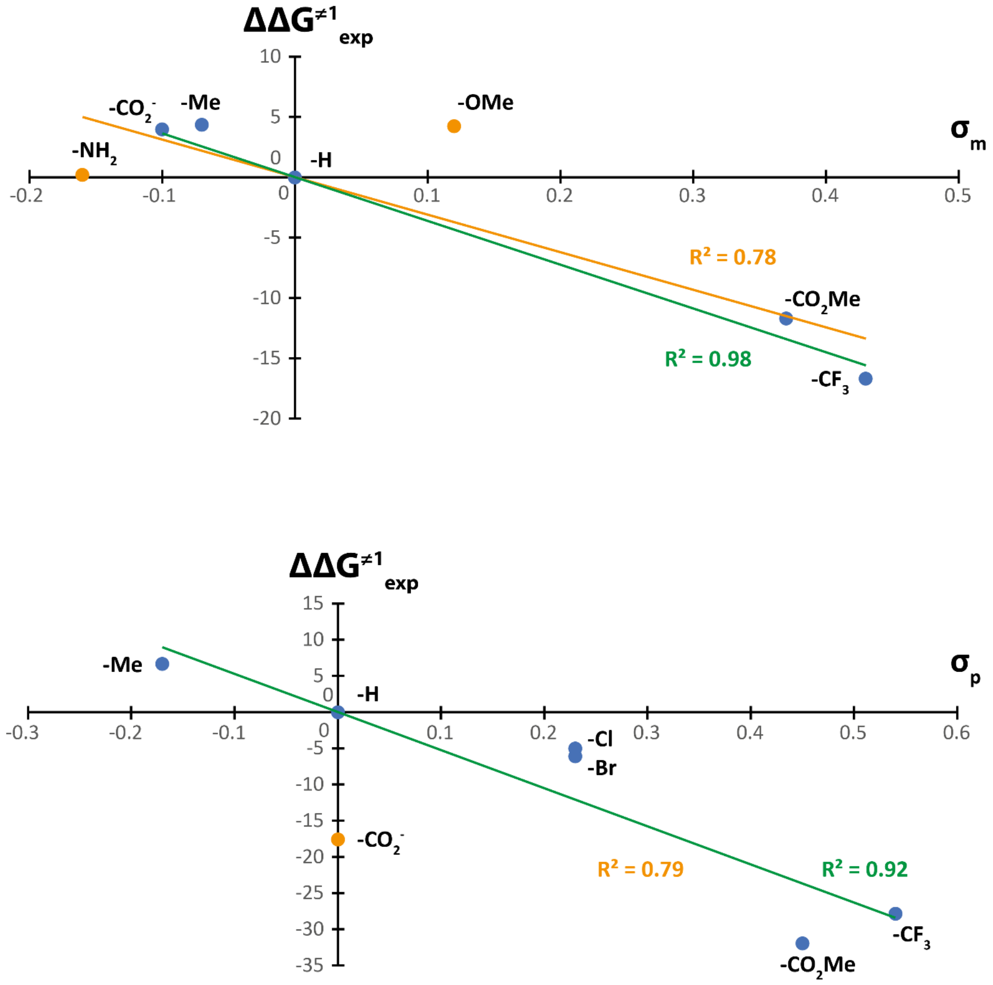

Figure S6. Correlation of Arrhenius derived experimental ΔΔG≠1exp (Y-axis, kJ/mol) and Hammett parameters. Outliers are shown in orange. Best fit from linear regression and coefficient of determination (R^2) are shown with inclusion (orange) or exclusion (green) of outliers.

Protein mass spectrometry

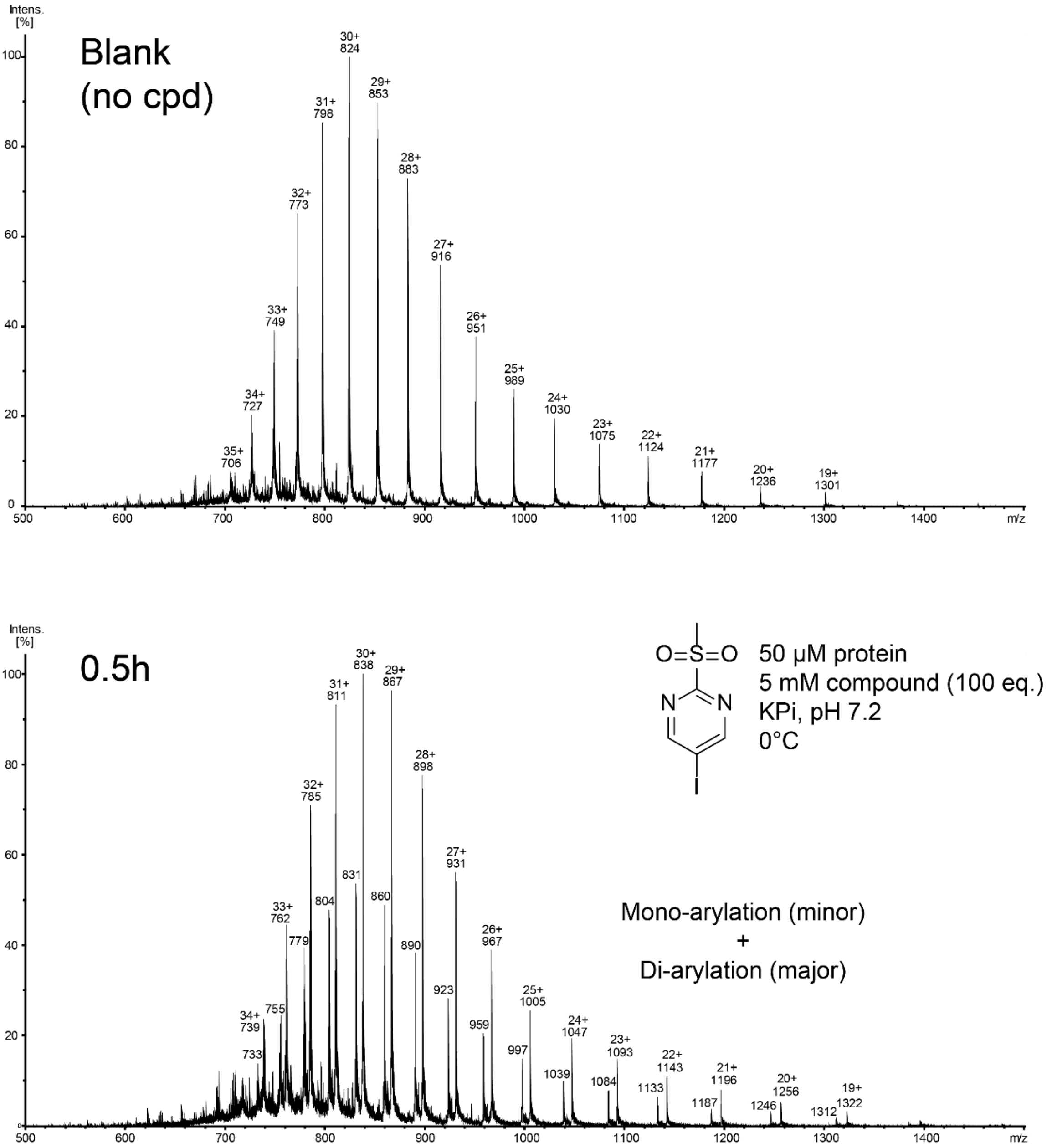

Figure S7. Representative ESI data prior to deconvolution showing the multiple charge states of unmodified p53-Y220C (top), mono- and di-arylated p53-Y220C (bottom) following treatment with 5-iodo-2-(methylsulfonyl)pyrimidine **4u**. See Figure 5A in the main text for the associated deconvoluted data.

X-ray crystallography

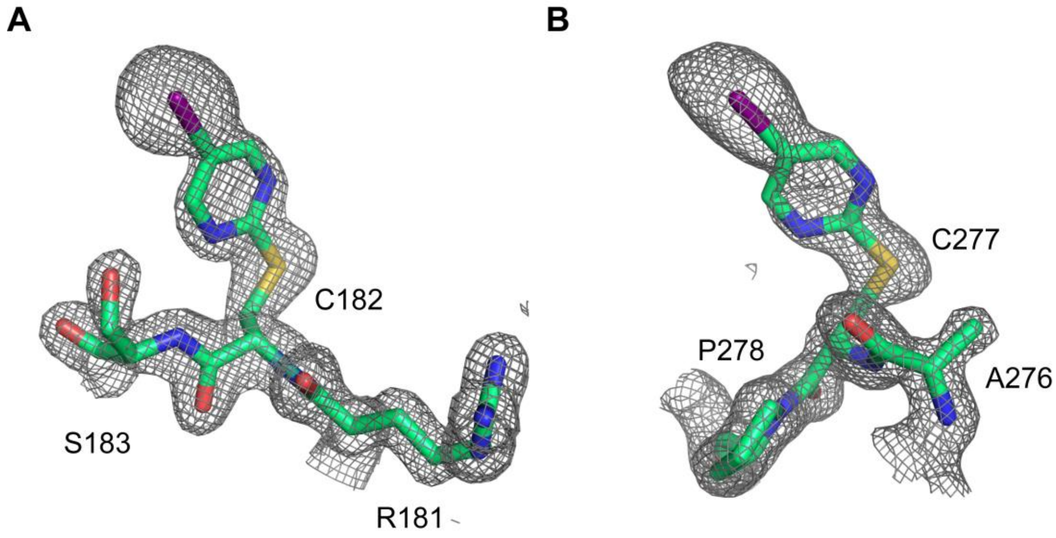

Figure S8. Electron density at modified cysteine residues in the p53-Y220C mutant structure after treatment with compound **4u**. 2Fo-Fc electron density maps are shown at a contour level of 1.3 σ for segments of chain B including the modified residues Cys182 (A) and Cys277 (B).

Table S9. X-ray data collection and refinement statistics

	p53-Y220C modified with compound **4u**
Data Collection	
Space Group	$P2_12_12_1$
a, b, c (Å)	64.88 71.05 104.89
α, β, γ (°)	90, 90, 90
Molecules per asymmetric unit	2
Resolution (Å)[a]	47.9-1.53
Unique reflections	73,231
Completeness (%)[a]	99.4 (99.4)
Multiplicity[a]	5.6 (5.7)
R_{merge} (%)[a]	4.0 (75.2)
CC(1/2)[a]	1.000 (0.892)
Mean $I/\sigma(I)$[a]	17.6 (2.3)
Refinement	
R_{work}, (%)[b]	15.8
R_{free}, (%)[b]	18.7
No. of atoms	
Protein[c]	3150
Zinc	2
Ethylene glycol	4
Water	443
RMSD bonds (Å)	0.006
RMSD angles (°)	0.78
Mean B (Å^2)	26.8
Ramachandran favored (%)[d]	99.3
Ramachandran outliers (%)[d]	0.0
PDB entry	8CG7

[a]Values in parentheses are for the highest-resolution shell.
[b]R_{work} and $R_{free} = \sum||F_{obs}| - |F_{calc}||/\sum|F_{obs}|$, where R_{free} was calculated with 5 % of the reflections chosen at random and not used in the refinement.
[c]Number includes alternative conformations and covalent modification.
[d]Calculated with MolProbity (Williams et al. 2018).[6]

Methods

Protein production. *Stabilised DNA binding domain of the human p53 cancer mutant Y220C (p53-Y220C)*. Stabilized p53-Y220C DBD (residues 94-312) was expressed and purified as previously described.[7] Briefly, the *N*-terminal fusion protein (6xHis/lipoyl domain/TEV protease cleavage site) was overexpressed using E. coli C41 cells in 2xTY medium at 20°C for 16 h and purified using standard Ni-affinity chromatography protocols. After overnight digestion with TEV protease, the p53-Y220C DBD was further purified using a Heparin column. Finally, gel filtration chromatography was performed using a Superdex 75 16/60 preparative gel filtration column (GE Healthcare) in a 25 mM KPi (pH 7.2), 150 mM NaCl, and 1 mM TCEP buffer. Molecular weight and protein purity (>95%) were confirmed via SDS gel electrophoresis and ESI-MS.

Human β-catenin armadillo domain. β-catenin sequence was amplified by PCR (KOD DNA polymerase, Merck Millipore) from plasmid templates and cloned into bacterial expression vectors by restriction-free cloning. All plasmids were verified by sequencing. For protein production, bacterial expression vector pLipK-ARD (residues 150 – 662) was used. 6xHis-Lip-tagged recombinant proteins were purified from BL21(DE3) pRARE2 E. coli bacterial strains. Bacteria were grown in LB media supplemented with appropriate antibiotic to OD_{600} 0.6, then dropped to a lower temperature (16 – 24 °C) and induced at OD_{600} 0.8 by addition of 0.4 mM isopropyl β-D-1-thiogalactopyranoside (IPTG). Proteins were expressed overnight and bacteria were subsequently harvested by centrifugation, cell pellets shock-frozen in liquid nitrogen and stored at -80°C until use. Cell pellets were re-suspended in lysis buffer (25 mM Tris-HCl pH 8, 200 mM NaCl, 20 mM imidazole, 10 µg/mL DNAse, EDTA-free protease inhibitor cocktail) and lysed with an Emulsiflex C-3 (Avestin). Lysates were cleared by ultracentrifugation (140,000x rcf, 30 minutes, 4°C) and mixed with Ni-NTA agarose. Beads were washed multiple times with lysis buffer, and 6xHisLip-tagged protein was eluted with lysis buffer supplemented with 500 mM imidazole. The protein was further purified by SEC on a HiPrep26/60 G200 (GE Healthcare) into the final buffer of 200 mM NaCl, 25 mM Tris pH 7.4, 0.06% NaN_3, 1 mM DTT, and protein purity was assessed by SDS-PAGE. Pure protein fractions were concentrated using a 10 kDa MWCO Vivaspin 20 concentrator (Sartorius) to 17 – 28 mg/mL, then shock frozen as droplets in liquid nitrogen and stored at -80°C until use.

Human formylglycine generating enzyme (FGE-C341S). The cloning of human FGE-WT cDNA into baculoviral transfer vector pAcGP67B-His_7 and the generation of the recombinant virus (in Sf9 cells) for expression of *C*-terminally His-tagged recombinant human FGE in High Five insect cells have been described earlier by Peng et al.[8]Expression constructs for FGE-active site cysteine to serine FGE-C341S were generated by site-directed PCR mutagenesis using pAcGP67B-FGE-WT-His_7 as template and the following complementary primers (only coding strand sequences shown): FGE-C341S_fwd: 5'-TGCCATAGGTCTTATAGTTACAGGTATCGCTGT-3'. All expression plasmids were verified by sequencing of the entire coding region to exclude any undesired PCR-derived errors. The purification method was essentially performed as described in Peng et al., with one modification. The size exclusion chromatography step was skipped and FGE-C341S was purified first with a metal-affinity chromatography (His-trap) step followed directly by a second step purification using the ion-exchange column chromatography (Resource-Q). Note: WT human FGE possesses C341 mediated redox catalytic activity. Its corresponding C341S mutant dis catalytically inactive but retains a virtually identical structure and stability, and is routinely used for biophysical and structural studies.

NMR studies: rate constant determination and hydrolytic stability. 850 µL of 50 mM Kpi buffer solution (pH 8.2 or 7), 20 µL of the appropriate pyrimidine stock solution (100 mM in DMSO-d^6), 20 µL of TMSP stock solution (22.2 mM in DI water), 30 µL DMSO-d^6, and 80 µL of GSH stock solution (0.25 M in DI water, reactivity only) were successively added/mixed at room temperature, final pH 7.0 or 6.5 (**Table S1**). 600 µL of this freshly prepared solution was poured into an NMR tube and immediately

transferred to the NMR instrument for acquisition. ^{1}H spectra (16 scans) were recorded every 10 minutes under water suppression conditions. Following acquisition, spectra were analysed using TopSpin. Normalised integrations of the pyrimidine aromatic ^{1}H signals in the starting material and product were plotted as a function of time (s), using the TMSP peak as standard for integral calibration/normalisation and monitoring of compound solubility, allowing extraction of pseudo first order reaction rate constants (*k'*). Second order rate constants (*k*) were back calculated from the ratio of the pseudo first counterparts and the concentration of thiol in solution. In all cases, chemoselectivity was confirmed by formation of a single *S*-arylated product (characteristic 2-alkylthioether signals), concomitant with formation of the sulfinic acid by-product (leaving group). Hydrolytic stability assessment was performed at pH 7.0 and pH 8.2 in a similar manner, in the absence of thiol.

	Solutions used	**Stability assays**		**Reactivity assays**	
DMSO-d6	100 mM pyrimidine solution	20 μL	2 mM	20 μL	2 mM
dWater	22,2 mM of TMSP	20 μL	0,44 mM	20 μL	0,44 mM
	DMSO-d6	30 μL	5 %	30 μL	5 %
dWater	50 mM KPi Buffer solution	930 μL		850 μL	
dWater	0,25M GSH	-		80 μL	20 mM

Table S1. Volumes and concentrations of solutions used for reactivity and stability NMR assays.

Chemoselectivity. Similarly, but replacing GSH by other amino acids, reference 2-methylsulfonylpyrimidine **4q** in 50 mM KPi (pH 8.2) was incubated with 10 equivalents of either Boc-Lys-OH, L-ser-OMe, N-Ac-Tyr-OH or Proline were used. No reactivity was observed over a 6 hours timescale.

UV/Vis experiments: rate constant determination of fast reacting (t100% <8 minutes) warheads. A 1 cm pathlength quartz cuvette (Hellma Analytics 111-QS 10x10 mm made of Quartz Suprasil®) was used to record UV/Vis spectra evolution over time using OceanOptics DH-2000-BAL light source connected to the USB2000+ Fiber Optic Spectrometer. SpectraSuite software was used for data acquisition. A 50 mM KPi buffer with 5% DMSO-d^{6} solution was measured as a background prior to each experiment. All experiments were recorded in duplicates both at pH 6.5 and 7.0. The appropriate warhead (20-100 μM) and GSH were successively added and rapidly mixed in a 1:10 ratio directly in the cuvette, and spectra were immediately acquired every second or 100 ms. Spectra were post-processed using MatLab® and the N-Way Toolbox.[9] Analysis was performed using PARAFAC Parallel Factor model. Pseudo-first order rate constants (*k'*) were obtained by "one phase decay" fitting of the data in GraphPad Prism, from which second order rate constants (*k*) were derived.

Computational Methods

Geometry optimizations and single-point energy calculations were carried out using Gaussian 09[10] in combination with GaussView.[11] The ωB97XD/6-31+G(d,p)[12,13] combined with the SMD[14] solvent model (water) provided the best agreement between calculated activation energy and experimental rate constants for SNAr reaction on different pyrimidine substrates.

First transition states (TS1) were found using ωb97xd/6-31+G(d,p) method by scanning the distance between the nucleophile (methanethiolate) and the electrophilic sulfone carbon atom, followed by optimisation to a transition state to reach a local maxima with a single imaginary frequency. TS1 were validated for every example by internal reaction coordinate (IRC) scans in both directions, falling to the reactant complex, the Meisenheimer intermediate, or sulfide-pyrimidine products. Second transition states (TS2) were found using the same strategy, also being validated for every example by IRC in both directions, falling to reaction products or the Meisenheimer intermediate

Energy and Vibrational Frequency calculations were run using ωb97xd/6-31+G(d,p) for every optimised structure found to determine free energy values (G) and compare the functional performance against experimental results. Corrected Gibbs energy of activation ($\Delta\Delta G^{\neq}$) with reference sulfonyl pyrimidine (**X**) was then plotted *vs* normalized experimental rate constants obtained at pH = 7.

Differential scanning fluorimetry (DSF). DSF experiments were performed on a Bio-rad CFX Connect Real-time qPCR system, using SYPRO Orange (Life Technologies) as a reporter dye to monitor protein denaturation. In brief: compounds were plated in DMSO at a stock concentration of 2 mM and stored at -20°C when not in use. A "mastermix" was prepared with assay buffer, SYPRO orange and protein. The mastermix (23.75 µL) was added to compound stocks in DMSO (1.25 µL) pre-plated on a 96-wp. Final concentrations: SYPRO Orange (10x), protein (8µM β-cat, 8µM p53-Y220C, 10µM FGE), compound (100 µM) and DMSO concentration 5% (v/v). 20 µL of the resulting samples were transferred to a Bio-Rad Hard-Shell® 96-Well PCR Plate (HSP9655), and sealed with Microseal 'B' PCR Plate Sealing Film (MSB1001). The temperature was raised (20 °C to 84 °C over 31 minutes) and the time dependent fluorescence recorded. The time dependent fluorescence data were analysed using GraphPad Prism 9 and the melting temperature (T_m) of individual sample wells was determined as the maximum of the first derivative of the data. The compound induced thermal stabilisation (ΔT_m) values were calculated as $\Delta T_m = T_m$ (protein + compound) − T_m (protein). All DSF experiments were performed in triplicate.

Protein	Assay buffer	T_m (°C, no compound)
P53-Y220C	25mM KPi (pH 7.2), 150 mM NaCl, 1mM TCEP, 5% dmso (v/v)	40.3
β-catenin	25mM Tris (pH 7.4), 200 mM NaCl, 0.06% NaN_3, 1mM DTT, 5% dmso (v/v)	54.5
FGE-C341S	25mM KPi (pH 7.6), 150 mM NaCl, 1mM TCEP, 5% dmso (v/v)	50.3

Protein mass spectrometry. Sample preparation: 50 µM of the Y220C mutant p53 DNA-binding domain in KPi buffer pH 6.0 – 8.0, temperature 0 – 20 °C, was incubated with 20-100 equivalents of the appropriate arylating agent (from concentrated dmso stocks), 5% v/v final dmso content, 50 µL final volume. For "Blank" samples, pure dmso was used instead of dmso compound stocks. Small aliquots were diluted 5-fold with DI water at regular time points, and immediately submitted for MS analysis.

Chromatography, data acquisition and processing: Separations were performed using a Dionex Ultimate 3000 UHPLC system (ThermoFisher, Hemel Hempstead, UK) with a Waters (Wilmslow, UK) Acquity UPLC BEH C18 packed column 1.7 µm particle size, 50 mm x 2.1 mm. The column was maintained at 50 °C and 2.0 uL of sample was injected. Solvent A, water 0.2% formic acid and solvent B, acetonitrile 0.2% formic acid were used for separation at a flow rate of 0.6 mL/min. A five-minute gradient elution was

performed as follows: Solvent B held at 5% and then increased to 100% until 2.8 min, held for one min and then returned to 5%. Following injection, the flow for the first minute of each acquisition was diverted to waste. A variable wavelength detector was set at 254 nm.

Full scan positive ion electrospray ionisation mass spectra were recorded over the m/z range 150-1500 using a maXis TOF mass spectrometer (Bruker Daltonics, Bremen, Germany) with the following conditions: Capillary 4500 V, end plate offset -500 V, nebuliser gas 3.0 bar at a flow rate of 6 L/min, dry heater 230OC.

The mass spectra were deconvoluted with ESI Compass 1.3 Maximum Entropy using Bruker DataAnalysis Version 4.0 SP 4 (Build 281) software.

Protein crystallography. Crystals of a stabilized variant of the Y220C mutant p53 DNA-binding domain[15] were grown at 20 °C using the vapor diffusion technique. Protein solution: 6 mg/ml in 25 mM phosphate buffer, pH 7.2, 0.5 mM TCEP. Crystallization buffer: 19% polyethylene glycol 4000, 100 mM Hepes, pH 7.0. For covalent modifications, crystals were soaked for 4 hours in crystallization buffer complemented with 20% glycerol and 30 mM compound and subsequently flash frozen in liquid nitrogen. X-ray data sets were collected at 100 K at beamline X06SA of the Swiss Light Source, Villigen, Switzerland. The diffraction data were integrated with XDS[16] and scaled with AIMLESS,[17] which is part of the CCP4 program suite.[17] The structure was solved with the program PHENIX[18] using PDB entry 6SHZ[19] as a starting model and initial rigid body refinement. The structure was then refined using iterative cycles of manual model building in COOT [20] and refinement in PHENIX. Data collection and refinement statistics are shown in **Table S9**. Structural figures were prepared using PyMOL (www.pymol.org).

Synthetic chemistry procedures

General information

All reagents and solvents were obtained from commercial sources and were used without further purification unless otherwise indicated. Reaction monitoring was performed by analytical thin layer chromatography (TLC) on aluminium sheets coated with silica gel 60 F254 from Merck KGaA. Eluted TLCs were visualised under UV light (254 nm) and/or by staining with vanilin or potassium permanganate upon heating. Crude mixtures were purified by flash column chromatography on silica gel 60 (230-400 mesh, 0.040-0.063 mm) purchased from Merck KGaA. Solvents were removed by rotary evaporator below 40˚C and the compounds further dried using high vacuum pumps.

Mass Spectrometry

Low resolution mass spectrometry (LRMS) was carried on a Waters TQD mass spectrometer equipped with a triple quadrupole analyser with UHPLC injection [BEH C18 column; H_2O-MeCN gradient {0.2% formic acid}]. High resolution mass spectrometry (HRMS) was carried out on a MaXis, Bruker Daltonics, with a Time of Flight (TOF) analyser. Samples were introduced to the mass spectrometer via a Dionex Ultimate 3000 autosampler and uHPLC pump. Ultrahigh performance liquid chromatography was performed using a Waters, Acquity UPLC BEH C18 (50 mm x 2.1 mm 1.7 um) column. Gradient elution from 5% acetonitrile (0.2% formic acid) to 100% acetonitrile (0.2% formic acid) was performed in five minutes at 0.6 mL/min.

Nuclear Magnetic Resonance

Proton (^{1}H), carbon (^{13}C) and fluorine (^{19}F) nuclear Magnetic Resonance (NMR) spectra were recorded on Bruker AV400 and AV3-400 spectrometers in the indicated deuterated solvent at a constant temperature of 298 K. Chemical shifts for ^{1}H and ^{13}C spectra are reported on the delta (δ) scale in parts per million (ppm) from low to high field and referenced to residual solvent reference: ^{1}H δ = 7.26 ($CDCl_3$), 2.50 (d^6-DMSO), 3.31 (CD_3OD), ^{13}C δ = 77.16 ($CDCl_3$), 39.52 (d^6-DMSO), 49.05 (CD_3OD). Data are presented as follows: chemical shift, multiplicity (s = singlet, br. s = broad singlet, d = doublet, dd = doublet of doublets, ddd = doublet of doublets of doublets, dt = doublet of triplets, t = triplet, td = triplet of doublets, tdd = triplet of doublets of doublets, q = quadruplet, m = multiplet), coupling constants (*J)* expressed in Hz, integration value and assignment. The subscript Ar means aromatic. The superscript III means tertiary carbon and the superscript IV means quaternary carbon. Carbon multiplicities were assigned by Distortionless Enhancement by Polarization Transfer (DEPT) experiments. Where required, ^{1}H and ^{13}C signals were assigned by correlation spectroscopy (COSY), Heteronuclear Single Quantum Correlation (HSQC), Heteronuclear Multiple-Bond Correlation spectroscopy (HMBC) and Nuclear Overhauser Effect Spectroscopy (NOESY).

Fourier-transform infrared (FT-IR)

Spectra are reported in wavenumbers (cm^{-1}) and were recorded as neat films on a Thermo Scientific Nicolet iS5 spectrometer using neat samples (solid or liquid).

General Procedures

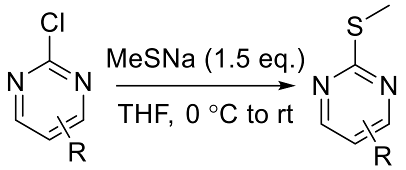

<u>Procedure A:</u> **S_NAr of diverse 2-chloropyrimidines with methanethiolate – Variation of the pyrimidine substitution pattern (R).** The appropriate 2-chloropyrimidine (1.0 eq.) and sodium methanethiolate (1.1-1.5 eq) were dissolved in anhydrous THF (c = 0.3 mol/L) at 0 °C under argon atmosphere. The resulting solution was allowed to warm to room temperature and stirred overnight until TLC and/or LCMS analysis showed completion of the reaction. In case of slow conversion the solution was further heated at 50 °C until full consumption of the pyrimidine. The resulting mixture was concentrated to dryness. The residue was partitioned between ethyl acetate (EA) and deionised water, and the aqueous phase was extracted with EA (x3). The combined organic layers were washed with deionised water twice, and finally brine. The organic phase was then dried over $MgSO_4$, filtered and concentrated under reduced pressure. The product was purified by flash column chromatography on silica gel.

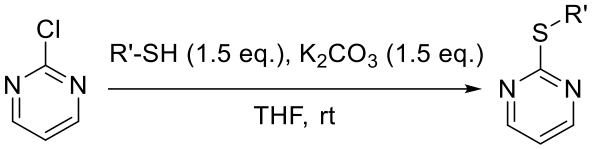

<u>Procedure B:</u> **S_NAr of 2-chloropyrimidine with diverse thiols – Variation of sulfur substitution (R').** 2-Chloropyrimidine **1q** (1 eq.), the appropriate thiol (1.5 eq.) and K_2CO_3 (1.5 eq.) were dissolved in THF (c = 0.3 mol/L). The resulting mixture was stirred at room temperature under argon atmosphere. After completion, the reaction was evaporated to dryness, the remaining residue was partitioned between EA and water. The aqueous phase was extracted with EA (x3). The combined organic layers were washed with water (x2), brine once (x1), dried over $MgSO_4$, filtered and concentrated under reduced pressure. The product was purified by flash column chromatography on silica gel.

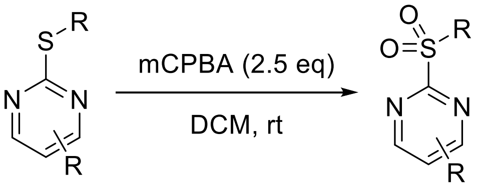

<u>Procedure C:</u> **Oxidation of thioethers to the corresponding sulfones.** The appropriate substrate (1 eq.) was dissolved in DCM (c = 0.1 mol/L) at room temperature. mCPBA (>77% purity) (2.5 eq.) was added portion wise to the solution under argon atmosphere. The resulting solution was stirred at room temperature until TLC indicated completion of the reaction. Generally, the solution turned progressively turbid with mCBA formation. The reaction was quenched by addition of a few drops of concentrated aq. Sodiul thiosulfate, followed by sat. aq. $NaHCO_3$. After a further 5 minutes of stirring and solubilisation of mCBA, the aqueous and organic phase was separated. The aqueous phase was further extracted with DCM (x3). The combined organic layers were washed with water (x2), dried over $MgSO_4$, filtered and concentrated under reduced pressure. The product was purified by flash chromatography on silica gel.

Compounds characterisation

2-Chloropyrimidine-4-carboxylic acid methyl ester (1k)

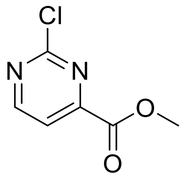

Chemical Formula: $C_6H_5ClN_2O_2$
Molecular Weight: 172,57

2-Chloro-4-pyrimidinecarboxylic acid **1d** (250 mg, 1.58 mmol, 1eq.) was dissolved in DCM (4 mL) and few drops of DMF. Oxalyl chloride 2M (0.15 mL, 1.73 mmol, 1.1 eq.) was added dropwise at 0 °C. The reaction was stirred at room temperature under argon atmosphere for 2h. MeOH (0.5 mL) was added and the reaction stirred for further 1h10min. The solution was then evaporated to dryness. The residue was partitioned between EA and water. The aqueous phase was extracted with EA (x5). The combined organic layers were washed successively with H_2O (x1), $NaHCO_3$ (x1), H_2O (x1), brine (x1), dried over $MgSO_4$, filtered and evaporated to dryness. The crude was purified by flash column chromatography on silica gel (8:2 DCM/hexane) to afford product **1k** (189 mg, 0.49 mmol, 69%) as a white solid. **R_f (product)** = 0.2 (7:3 DCM/hexane); **Mp** = 100 °C; **^{1}H-NMR (400MHz, DMSO-d^6):** δ_H 9.06 (d, *J* = 5.0 Hz, 1H), 8.06 (d, *J* = 5.0 Hz, 1H), 3.93 (s, 3H); **^{13}C-NMR (100MHz, DMSO-d^6):** δ_C 163.2, 162.9, 160.4, 157.0, 120.1, 53.3; **IR:** 3135, 3089, 3059, 2964, 1743, 1559, 1546, 1443, 1350, 1309, 1287, 1203, 1178, 1158, 973, 878, 858, 764, 748, 672 cm^{-1}; **HRMS (ESI)** *m/z* $[M+H]^+$ calcd for $C_6H_6ClN_2O_2$ 173.0112; Found 173.0112

2-Chloro-5-phenylpyrimidine (1p)

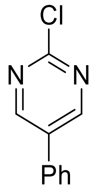

Chemical Formula: $C_{10}H_7ClN_2$
Molecular Weight: 190,6

2-Chloro-5-phenylpyrimidine **1p** was prepared following procedure from Chatzopoulou et al.[21] starting from commercially available 5-bromo-2-chloropyrimidine **1t** (202 mg, 1.04 mmol, 1 eq.). The crude was purified by column chromatography on silica gel (eluent 100% DCM) affording the 2-Chloro-5-phenylpyrimidine **1p** (155 mg, 0.81 mmol, 78%) as a white solid. Spectral data were in accordance with those reported in literature.[21] **^{1}H-NMR (400MHz, $CDCl_3$):** δ_H 8.83 (s, 2H), 7.58-7.46 (m, 5H)

4-Methoxy-2-(methylthio)pyrimidine (2b)

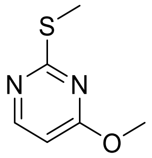

Chemical Formula: $C_6H_8N_2OS$
Molecular Weight: 156,20

Procedure A was followed using 2-Chloro-4-methoxypyrimidine **1b** (405 mg, 2.80 mmol) and 1.5 eq. of sodium methanethiolate (315 mg, 4.27 mmol). The reaction was stirred for 16h. The crude was purified by column chromatography on silica gel (4:6 DCM/hexane) to afford product **2b** (393 mg, 2.52 mmol, 90 %) as a white solid. Analytical data were in accordance with those reported in literature.[22] **R_f (product)** = 0.2 (1:1 DCM/hexane); **Mp** = 38°C; **^{1}H-NMR (400MHz, DMSO-d^6):** δ_H 8.33 (d, *J* = 5.7 Hz, 1H), 6.62 (d, *J* = 5.7 Hz, 1H), 3.90 (s, 3H), 2.50 (s, 3H)

4-Methyl-2-(methylthio)pyrimidine (2c)

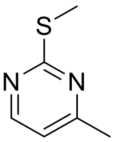

Chemical Formula: $C_6H_8N_2S$
Molecular Weight: 140,20

Procedure A was followed using 2-chloro-4-methylpyrimidine **1c** (393 mg, 3.06 mmol) and 1.5 eq. of sodium methanethiolate (339 mg, 4.59 mmol). The reaction was stirred for 21h. The crude was purified by column chromatography on silica gel (1:9 EA/hexane) to afford product **2c** (380 mg, 2.71 mmol, 88 %) as an oil. Analytical data are in accordance with those reported in literature.[23] **R_f (product)** = 0.4 (1:9 EA/hexane); **^{1}H-NMR (400MHz, DMSO-d^6):** δ_H 8.47 (d, *J* = 5.1 Hz, 1H), 7.07 (d, *J* = 5.1 Hz, 1H), 2.48 (s, 3H), 2.40 (s, 3H)

2-Methylsulfanyl-4-carboxylic acid pyrimidine (2d)

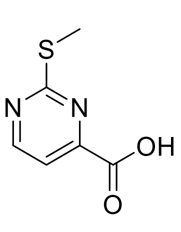

Chemical Formula: $C_6H_6N_2O_2S$
Molecular Weight: 170,19

2-Chloro-4-pyrimidinecarboxylic acid **1d** (2.01 g, 12.7 mmol, 1 eq.), K_2CO_3 (1.93 g, 14.0 mmol, 1.1 eq.) and sodium methane thiolate (1.40 g, 19.0 mmol, 1.5 eq.) were dissolved in MeOH (42 mL) under an argon atmosphere. The reaction was stirred for 18h. The reaction formed a paste which was dissolved with MeOH and EA and then evaporated. The crude was solubilised in deionised water. The pH of the aqueous phase was adjusted to ca. 3-4 by dropwise addition of HCl 1M until a white solid crashed out.

The solid was filtered and rinsed with cold water to afford XX013B as a pure product (1.70 g, 10.0 mmol). The filtrate was extracted with EA (x2), $CHCl_3$/iPrOH (8:2) (x3). The combined organic layers were dried over MgSO4, filtered and evaporated to dryness, to afford product **2d** (192 mg, 1.13 mmol) as a white solid without further purification. The two combined fractions of product gave an overall yield of 87% (1.89 g, 11.1 mmol). Analytical data are in accordance with those reported in literature.[24] **R_f (product)** = 0.5 (95:5 DCM/MeOH + 2 % AcOH); **Mp** = 213 °C; **^{1}H-NMR (400MHz, DMSO-d^6):** δ_H 13.92 (br. s, 1H), 8.86 (d, *J* = 4.9 Hz, 1H), 7.64 (d, *J* = 4.9 Hz, 1H), 2.56 (s, 3H)

2-(Methylthio)-4-phenylpyrimidine (2e)

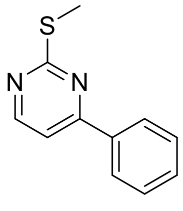

Chemical Formula: $C_{11}H_{10}N_2S$
Molecular Weight: 202,28

Procedure A was followed using 2-chloro-4-phenylpyrimidine **1e** (404 mg, 2.12 mmol) as substrate and 1.5 eq. of sodium methanethiolate (241 mg, 3.27 mmol). The reaction was stirred for 23h. The crude was purified by column chromatography on silica gel (1:1 DCM/hexane) to afford product **2e** (410 mg, 2.03 mmol, 96 %) as a white solid. Analytical data are in accordance with those reported in literature.[25] **Mp** = 89 °C; **^{1}H-NMR (400MHz, DMSO-d^6):** δ_H 8.68 (d, *J* = 5.3 Hz, 1H), 8.22-8.17 (m, 2H), 7.77 (d, *J* = 5.3 Hz, 1H), 7.60-7.52 (m, 3H), 2.59 (s, 3H)

2-(Methylthio)-4-pyrimidinamine (2g)

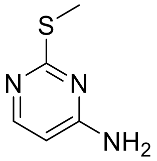

Chemical Formula: $C_5H_7N_3S$
Molecular Weight: 141,19

Procedure A was followed using 2-chloro-4-pyrimidinamine **1g** (502 mg, 3.87 mmol) as substrate, 1.5 eq. of sodium methanethiolate (429 mg, 5.81 mmol) and replacing THF by DMF. The reaction was stirred for 19h. The crude was purified by column chromatography on silica gel (4:6 DCM/hexane) to afford product **2g** (318 mg, 2.25 mmol, 58 %) as a white solid. Analytical data were in accordance with those reported in the literature.[26] **R_f** = 0.4 (1:1 DCM/hexane); **Mp** = 128°C; **^{1}H-NMR (400MHz, DMSO-d^6):** δ_H 7.89 (d, *J* = 5.8 Hz, 1H), 6.88 (br. s, 2H), 6.13 (d, *J* = 5.8 Hz, 1H), 2.38 (s, 3H)

2-Methylsulfanyl-4-carboxamide pyrimidine (2j)

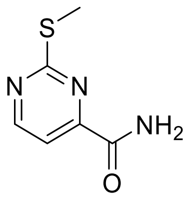

Chemical Formula: $C_6H_7N_3OS$
Molecular Weight: 169,20

Procedure A was followed using 2-chloro-4-pyrimidinecarboxamide **1j** (153 mg, 0.97 mmol) and 1.5 eq. of sodium methanethiolate (109 mg, 1.48 mmol). The reaction was stirred for 27h. The crude was purified by column chromatography on silica gel (35:55 EA/hexane) to afford product **2j** (154 mg, 0.91 mmol, 93 %) as a white solid. **R_f (product)** = 0.25 (4:6 EA/PE); **Mp** = 193 °C; **^{1}H-NMR (400MHz, DMSO-d^6):** δ_H 8.84 (d, *J* = 4.9 Hz, 1H), 8.20 (br s, 1H), 7.95 (br s, 1H), 7.64 (d, *J* = 4.9 Hz, 1H), 2.59 (s, 3H); **^{13}C-NMR (100MHz, DMSO-d^6):** δ_C 171.4, 164.3, 159.8, 157.4, 113.6, 13.6; **IR:** 3429, 3166, 2921, 2851, 1694, 1546, 1388, 1328, 1214, 1178, 1076, 860, 852, 799, 776, 715, 638, 560; **HRMS (ESI)** *m/z* $[M+Na]^+$ calcd for $C_6H_7N_3NaOS$ 192.0202; Found 192.0201.

2-Methylsulfanyl-4-methylester pyrimidine (2k)

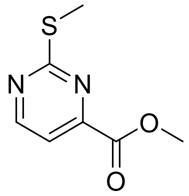

Chemical Formula: $C_7H_8N_2O_2S$
Molecular Weight: 184,21

Procedure A was followed using 2-Chloropyrimidine-4-carboxylic acid methyl ester **1k** (49 mg, 0.28 mmol) as substrate, 1.5 eq. of sodium methanethiolate (32 mg, 0.43 mmol) and with MeOH as solvent. The reaction was stirred for 66h. Pure product **2k** was obtained (35 mg, 0.19 mmol, 67%) after work-up without further purification. **R_f (product)** = 0.25 (9:1 DCM/hexane); **Mp** = 71 °C; **^{1}H-NMR (400MHz, DMSO-d^6):** δ_H 8.90 (d, *J* = 5.0 Hz, 1H), 7.68 (d, *J* = 5.0 Hz, 1H), 3.91 (s, 3H), 2.56 (s, 3H); **^{13}C-NMR (100MHz, DMSO-d^6):** δ_C 172.4, 163.9, 160.2, 154.7, 116.1, 53.1, 13.6; **IR:** 2952, 2923, 1722, 1553, 1441, 1420, 1334, 1320, 1309, 1208, 1148, 970, 861, 747, 673 cm^{-1}; **HRMS (ESI)** *m/z* $[M+H]^+$ calcd for $C_7H_9N_2O_2S$ 185.0379; Found 185.0379.

2-(Methylthio)-4-(trifluoromethyl)pyrimidine (2l)

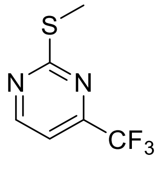

Chemical Formula: $C_6H_5F_3N_2S$
Molecular Weight: 194,18

Procedure A was followed using 2-Chloro-4-(trifluoromethyl)pyrimidine **1l** (100 mg, 0.55 mmol) and 1.4 eq. of sodium methanethiolate (58 mg, 0.78 mmol). The reaction was performed in DMF. The reaction was stirred for 18h. The crude was purified by column chromatography on silica gel (3:97 EA/hexane) to afford product **2l** (80 mg, 0.41 mmol, 75 %) as an oil. Analytical data were in accordance with those reported in the literature.[27] **R_f (product)** = 0.5 (1:9 EA/PE); **^{1}H-NMR (400MHz, DMSO-d^6):** δ_H 8.99 (d, *J* = 5.0 Hz, 1H), 7.69 (d, *J* = 5.0 Hz, 1H), 2.57 (s, 3H); **^{19}F-NMR (376MHZ, DMSO-d^6):** δ_F - 68.8

2-(methylthio)-5-nitropyrimidine (2m)

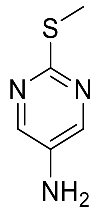

Chemical Formula: $C_5H_7N_3S$
Molecular Weight: 141,19

2-(methylthio)-5-nitropyrimidine **2w** (500 mg, 2.92 mmol, 1 eq.) was dissolved in EtOH (20 mL) and AcOH (12 mL) under Argon atmosphere. Iron powder was added (1.62 g, 29.2 mmol, 10 eq.) portionwise to the mixture. The reaction was stirred and heated at 80 °C for 2.5h. The reaction was then concentrated. The residue was partitioned between EA and $NaCO_3$ sat. solution. The aqueous phase was extracted with EA (x3). The combined organic layers were washed with brine, dried over $MgSO_4$, filtered and concentrated. The crude was purified by flash column chromatography on silica gel (65:35 DCM/EA) to afford product **2m** (360 mg, 2.55 mmol, 87 %) as a beige solid. **R_f (product) =** 0.25 (7:3 DCM/hexane); **^{1}H-NMR (400MHz, DMSO-d^6):** δ_H 8.03 (s, 2H), 5.30 (br. s, 2H), 2.41 (s, 3H); **^{13}C-NMR (100MHz, DMSO-d^6):** δ_C 156.3, 143.1, 139.5, 13.6; **IR:** 3329, 3210, 1624, 1543, 1400, 1304, 1205, 1183, 759, 643 cm^{-1}; **HRMS (ESI)** *m/z* $[M+H]^+$ calcd for $C_5H_8N_3S$ 142.0433; Found 142.0438.

2- (methylthio)-5-methoxypyrimidine (2n)

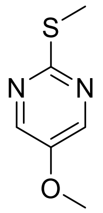

Chemical Formula: $C_6H_8N_2OS$
Molecular Weight: 156,20

Procedure A was followed using 2-Chloro-5-methoxypyrimidine **1n** (70 mg, 4.84 mmol) and 1.5 eq. of sodium methanethiolate (520 mg, 7.26 mmol). The crude was purified by column chromatography on silica gel (85:15 PE/DCM) to afford product **2n** (605 mg, 3.87 mmol, 80 %) as a white solid. Data were in accordance with those reported in literature.[28] **Mp** = 63 °C; **^{1}H-NMR (400MHz, DMSO-d^6):** δ_H 8.45 (s, 2H), 3.86 (s, 3H), 2.49 (s, 3H)

2-(methylthio)-5-methylpyrimidine (2o)

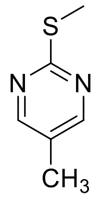

Chemical Formula: $C_6H_8N_2S$
Molecular Weight: 140,20

Procedure A was followed using 2-Chloro-5-methylpyrimidine **1o** (1.50 g, 11.7 mmol) and 1.1 eq. of sodium methanethiolate (903 mg, 12.9 mmol). The crude was purified by column chromatography on silica gel (85:15 hexane/EA) to afford product **2o** (1.49 g, 10.6 mmol, 91 %) as an oil. **R_f (product)** = 0.35 (8:2 hexane/EA); **^{1}H-NMR (400MHz, DMSO-d^6):** δ_H 8.48 (s, 2H), 2.48 (s, 3H), 2.19 (s, 3H); **^{13}C-NMR (100MHz, DMSO-d^6):** δ_C 168.2, 157.6, 125.9, 14.4, 13.5; **IR:** 3016, 2926, 1585, 1538, 1394, 1248, 1187, 1169, 1159, 1039, 993, 966, 911, 831, 769, 645 cm^{-1}; **HRMS (ESI)** *m/z* [M+H]$^+$ calcd for $C_6H_9N_2S$ 141.0481; Found 141.0484.

2-(Methylthio)-5-phenylpyrimidine (2p)

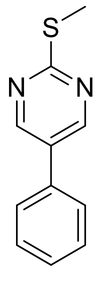

Chemical Formula: $C_{11}H_{10}N_2S$
Molecular Weight: 202,28

Procedure A was followed using 2-Chloro 5-phenylpyrimidine **1p** (113 mg, 0.59 mmol, 1 eq.) as substrate and 1.5 equivalent of sodium methanethionate (69 mg, 0.89 mmol). The reaction was stirred for 72h. The crude was purified by column chromatography on silica gel (1:1 DCM/hexane) to afford 2-(Methylthio)-5-phenylpyrimidine **2p** (117 mg, 0.58 mmol, 98%) as a white solid. **R_f (product) =** 0.20 (6:4 DCM/hexane); **Mp** = 98 °C; **^{1}H-NMR (400MHz, DMSO-d^6):** δ_H 8.97 (s, 2H), 7.78-7.73 (m, 2H), 7.54-7.48 (m, 2H), 7.47-7.41 (m, 1H), 2.56 (s, 3H); **^{13}C-NMR (100MHz, DMSO-d^6):** δ_C 170.2, 155.3, 133.7, 129.3, 128.6, 128.5, 126.5, 13.7; **IR:** 3059, 2924, 1582, 1530, 1403, 1372, 1197, 1184, 922, 773, 758, 699 cm^{-1}; **HRMS (ESI)** *m/z* [M+H]$^+$ calcd for $C_{11}H_{11}N_2S$ 203.0637; Found 203.0640.

2-(Methylthio)pyrimidine (2q)

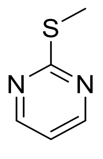

Chemical Formula: $C_5H_6N_2S$
Molecular Weight: 126,18

Procedure A was followed using 2-chloropyrimidine **1q** (1.50 g, 13.1 mmol) and 1.1 eq of sodium methanethiolate (1.01 g, 14.4 mmol). The crude was purified by column chromatography on silica gel (8:2 hexane/EA) to afford product **2q** (1.18 g, 9.35 mmol, 71 %) as an oil. Analytical data are in accordance with those reported in literature.[29] **R_f (product)** = 0.35 (8:2 hexane/EA); **^{1}H-NMR (400MHz, DMSO-d^6):** δ_H 8.64 (d, *J* = 4.8 Hz, 2H), 7.21 (t, *J* = 4.8 Hz, 1H), 2.50 (s, 3H)

5-Fluoro-2-(methylthio)pyrimidine (2r)

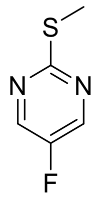

Chemical Formula: $C_5H_5FN_2S$
Molecular Weight: 144,17

Procedure A was followed using 2-chloro-5-fluoropyrimidine **1r** (1.50 g, 11.3 mmol) and 1.1 eq of sodium methanethiolate (872 mg, 12.5 mmol). The crude was purified by column chromatography on silica gel (95:5 hexane/EA) to afford product **2r** (867 mg, 6.01 mmol, 53 %) as an oil. **R_f (product)** = 0.4 (9:1 hexane/EA); **^{1}H-NMR (400MHz, DMSO-d^6):** δ_H 8.78 (d, *J* = 1 Hz, 2H), 2.52 (s, 3H); **^{13}C-NMR (100MHz, DMSO-d^6):** δ_C 166.7 (d, *J* = 4 Hz, 155.5 (d, *J* = 257 Hz), 145.9 (d, *J* = 21 Hz), 14.6; **^{19}F-NMR (376MHz, DMSO-d^6):** δ_F -145.8 (s, 1F); **IR:** 3041, 2930, 1553, 1388, 1239, 1191, 1176, 1156, 968, 924, 761, 642, 629 cm^{-1}; **HRMS (ESI)** *m/z* $[M+H]^+$ calcd for $C_5H_6FN_2S$ 145.0230; Found 145.0233.

5-Chloro-2-(methylthio)pyrimidine (2s)

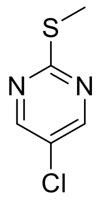

Chemical Formula: $C_5H_5ClN_2S$
Molecular Weight: 160,62

Procedure A was followed using 2,5-dichloropyrimidine **1s** (1.50 g, 10.1 mmol) and 1.1 eq of sodium methanethiolate (776 mg, 11.1 mmol). The crude was purified by column chromatography on silica gel (9:1 hexane/EA) to afford product **2s** (1.51 g, 9.40 mmol, 93 %) as a white solid. Analytical data are in accordance with those reported in literature.[30] **R_f (product) =** 0.5 (9:1 hexane/EA); **Mp** = 56 °C; **^{1}H-NMR (400MHz, DMSO-d^6):** δ_H 8.77 (s, 2H), 2.52 (s, 3H)

2-(methylthio)-5-bromopyrimidine (2t)

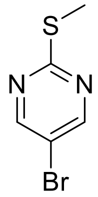

Chemical Formula: $C_5H_5BrN_2S$
Molecular Weight: 205,07

Procedure A was followed using 5-Bromo-2-chloropyrimidine **1t** (1.50 g, 7.76 mmol) and 1.1 eq. of sodium methane thiolate (598 mg, 8.53 mmol). The crude was purified by column chromatography on silica gel (9:1 hexane/EA) to afford product **2t** (1.51 g, 7.36 mmol, 95 %) as a white solid. **R_f (product)** = 0.75 (8:2 hexane/EA); **Mp** = 70-71 °C; **^{1}H-NMR (400MHz, DMSO-d^6):** δ_H 8.81 (s, 2H), 2.50 (s, 3H); **^{13}C-NMR (100MHz, DMSO-d^6):** δ_C 170.1, 158.0, 115.0, 13.9; **IR:** 3022, 2927, 1547, 1524, 1399, 1364, 1242, 1199, 1171, 1150, 969, 943, 762, 631 cm^{-1}; **HRMS (ESI)** *m/z* [M+H]$^+$ calcd for $C_5H_6BrN_2S$ 204.9430; Found 204.9434.

2-(methylthio)-5-iodopyrimidine (2u)

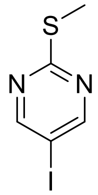

Chemical Formula: $C_5H_5IN_2S$
Molecular Weight: 252,07

Procedure A was followed using 2-chloro 5-iodopyrimidine **1u** (1.00 g, 4.16 mmol) and 1.1 eq. of sodium methanethiolate (0.66 g, 4.58 mmol). The crude was purified by column chromatography on silica gel (95:5 PE/EA) to afford product **2u** (0.645 g, 2.56 mmol, 61 %) as a white solid. **Mp** = 104 °C; **^{1}H-NMR (400MHz, DMSO-d^6):** δ_H 8.84 (s, 2H), 2.46 (s, 3H); **^{13}C-NMR (100MHz, DMSO-d^6):** δ_C 170.0, 162.5, 88.0, 13.7; **IR:** 2921, 1541, 1516, 1395, 1361, 1243, 1194, 1172, 999, 932, 762, 636 cm^{-1}; **HRMS (ESI)** *m/z* [M+H]$^+$ calcd for $C_5H_6IN_2S$ 252.9291; Found 252.9293.

2-(methylthio)pyrimidine-5-carboxylic acid (2v)

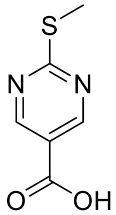

Chemical Formula: $C_6H_6N_2O_2S$
Molecular Weight: 170,19

Procedure A was followed using 2-chloro-5-pyrimidinecarboxylic acid **1v** (2.0 g, 12.6 mmol) and 1.1 equivalent of sodium methane thiolate (0.97 g, 13.9 mmol) and K_2CO_3 (2.61 g, 18.9 mmol, 1.5 eq.). Instead of partitioning with DCM/$NaHCO_3$ sat. solution, the crude residue after evaporation was

dissolved in distilled water. The resulting aqueous phase was acidified with 2M HCl until pH = 3. The white solid precipitate was washed with 10 mL of cold deionised water and filtered through Buchner. The crude was purified by flash chromatography on silica gel (DCM/MeOH 9:1) to afford product **2v** (1.30 g, 7.64 mmol, 61 %) as a white solid. **Mp** = 251 °C; **^{1}H-NMR (400MHz, DMSO-d^6):** δ_H 13.60 (s, 1H), 9.01 (s, 2H), 2.58 (s, 3H); **^{13}C-NMR (100MHz, DMSO-d^6):** δ_C 175.8, 164.9, 158.1, 120.0, 13.8; **IR:** 2521, 1717, 1583, 1538, 1385, 1285, 1243, 1205, 1189, 1168, 938, 834, 779, 654 cm^{-1}; **HRMS (ESI)** *m/z* [M+H]$^+$ calcd for $C_6H_7N_2O_2S$ 171.0223; Found 171.0224.

2-(methylthio)-5-nitropyrimidine (2w)

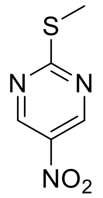

Chemical Formula: $C_5H_5N_3O_2S$
Molecular Weight: 171,17

Procedure A was followed using 2-chloro-5-nitropyrimidine **1w** (1.50 g, 9.40 mmol) and 1.1 equivalent of sodium methane thiolate (725 mg, 10.3 mmol). The reaction was stirred or 24h at room temperature. The resulting solution was diluted with Et_2O (100 mL) until a solid crashed out. The solid was collected by filtration giving a crude orange solid. The crude was purified by column chromatography on silica gel (8:2 hexane/EA) to afford product **2w** (1.40 g, 8.18 mmol, 87 %) as an off-white solid. **R_f (product) =** 0.55 (8:2 hexane/EA); **Mp** = 84 °C; **^{1}H-NMR (400MHz, DMSO-d^6):** δ_H 9.36 (s, 2H), 2.64 (s, 3H); **^{13}C-NMR (100MHz, DMSO-d^6):** δ_C 177.7, 153.0, 139.4, 14.0; **IR:** 3027, 1566, 1507, 1400, 1338, 1243, 1211, 1192, 1139, 859, 770, 635 cm^{-1}; **HRMS (ESI)** *m/z* [M+H]$^+$ calcd for $C_5H_6N_3O_2S$ 172.0175; Found 172.0174.

2-Methylsulfanylpyrimidine-5-carboxylic acid methyl ester (2y)

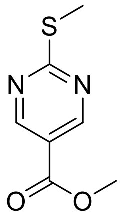

Chemical Formula: $C_7H_8N_2O_2S$
Molecular Weight: 184,21

Procedure A was followed using 2-chloropyrimidine-5-carboxylic acid methyl ester **1y** (0.5 g, 2.90 mmol) and 1.1 equivalent of sodium methanethiolate (235 mg, 3.19 mmol). The crude was purified by column chromatography on silica gel (85:15 DCM/PE) to afford product **2y** (416.3 mg, 2.26 mmol, 78 %) as a white solid. Analytical data are in accordance with those reported in literature.[31] **Mp** = 95 °C; **^{1}H-NMR (400MHz, DMSO-d^6):** δ_H 9.03 (s, 2H), 3.88 (s, 3H), 2.58 (s, 3H); **^{13}C-NMR (100MHz, DMSO-d^6):** δ_C 176.2, 164.0, 157.9, 119.2, 52.4, 13.8; **IR:** 3062, 1717, 1583, 1538, 1385, 1285, 1243, 1205, 1189, 938, 834, 779, 654 cm^{-1}; **HRMS (ESI)** *m/z* [M+H]$^+$ calcd for $C_7H_9N_2O_2S$ 185.0379; Found 185.0381.

2-(*tert*-Butylthio)pyrimidine (3a)

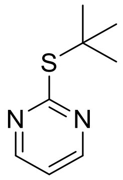

Chemical Formula: $C_8H_{12}N_2S$
Molecular Weight: 168,26

Procedure B was followed using 2-chloropyrimidine (109 mg, 0.95 mmol, 1 eq.), *t*-butyl sodium thiolate (175.5 mg, 1.31 mmol, 1.5 eq.), and DMF (3.2 mL, c = 0.3 mol/L) as solvent, without K_2CO_3. The reaction was stirred for 3 days at room temperature. The crude was purified using column chromatography on silica gel (5:95 EA/Hexane) to afford 2-(*t*-butylthio)pyrimidine **3a** (44 mg, 0.26 mmol, 27 %) as an oil. Analytical data are in accordance with those reported in literature.[32,33] **R_f (product)** = 0.35 (1:9 EA/hexane); **^{1}H-NMR (400MHz, DMSO-d^6):** δ_H 8.61 (d, *J* = 4.9 Hz, 2H), 7.17 (t, *J* = 4.9 Hz, 1H), 1.57 (s, 9H)

2-(*n*-butylthio)pyrimidine (3c)

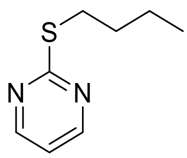

Chemical Formula: $C_8H_{12}N_2S$
Molecular Weight: 168,26

Procedure B was followed using *n*-butylthiol (0.14 mL, 1.31 mmol, 1.5 eq.) as nucleophile. The reaction was stirred for 18 hours. The crude was purified using column chromatography on silica gel (4:6 DCM/Hexane) to afford 2-(*n*-butylthio)pyrimidine **3c** (126 mg, 0.75 mmol, 98 %) as an oil. **R_f (product)** = 0.3 (4:6 DCM/hexane); **^{1}H-NMR (400MHz, DMSO-d^6):** δ_H 8.62 (d, *J* = 4.8 Hz, 2H), 7.19 (t, *J* = 4.9 Hz, 1H), 3.10 (t, *J* = 7.3 Hz, 2H), 1.68-1.59 (m, 2H), 1.41 (m, 2H), 0.90 (t, *J* = 7.4 Hz, 3H); **^{13}C-NMR (100MHz, DMSO-d^6):** δ_C 171.2, 157.7, 117.1, 30.9, 29.6, 21.4, 13.5; **IR:** 1182, 1375, 1544, 2360, 2929 cm^{-1}; **HRMS (ESI)** *m/z* $[M+H]^+$ calcd for $C_8H_{13}N_2S$ 169.0794; Found 169.0798.

2-(phenylthio)pyrimidine (3d)

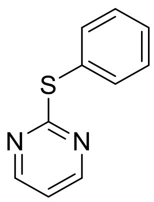

Chemical Formula: $C_{10}H_8N_2S$
Molecular Weight: 188,25

Procedure B was followed using thiophenol (144 mg, 1.31 mmol, 1.5 eq.) as the nucleophile. After 48 hours, additional thiophenol (97 mg, 1.1 eq.) and K_2CO_3 (121 mg, 1 eq.) were added to the mixture. The reaction was complete 24 hours after the second addition. The crude was purified using column chromatography on silica gel (15:85 EA/hexane) to afford 2-(phenylthio)pyrimidine **3d** (88 mg, 0.47 mmol, 54 %) as a white solid. Analytical data are in accordance with those reported in literature.[34] **Mp**

= 90 °C; **R_f (product)** = 0.4 (15:85 EA/hexane); **^{1}H-NMR (400MHz, DMSO-d^{6}):** δ_H 8.59 (d, *J* = 4.9 Hz, 2H), 7.63-7.58 (m, 2H), 7.49-7.45 (m, 3H), 7.23 (t, *J* = 4.9 Hz, 1H)

2-((4-nitrophenyl)thio)pyrimidine (3f)

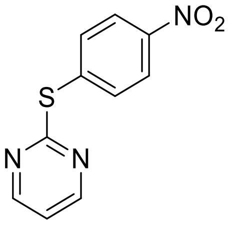

Chemical Formula: $C_{10}H_7N_3O_2S$
Molecular Weight: 233,25

1-Chloro 4-nitrobenzene (382 mg, 2.4 mmol, 1.2 eq.), 2-mercaptopyrimidine (229 mg, 2.0 mmol, 1 eq.) and then K_2CO_3 (553 mg, 4.0 mmol, 2 eq.) were successively mixed in DMF (10 mL, c = 0.2 M) at room temperature. The reaction mixture was then heated at 110 °C under nitrogen atmosphere for 20h. After completion, EA and H_2O were added to the mixture and the organic and aqueous phases were separated. The aqueous phase was extracted with EA (x3). The combined organic layers were washed with H_2O (5 x 20 mL) and then Brine, dried over $MgSO_4$, filtered and evaporated to dryness. The residue was purified by column chromatography on silica gel (Eluent 6:4 to 8:2 DCM/hexane) to afford **3f** (432 mg, 1.85 mmol, 93%) as a beige solid. **Mp** = 118 - 120 °C; **R_f (product)** = 0.3 (6:4 DCM/hex); **^{1}H-NMR (400MHz, DMSO-d^{6}):** δ_H 8.66 (d, *J* = 4.9 Hz, 2H), 8.30-8.25 (m, 2H), 7.94-7.89 (m, 2H), 7.33 (t, *J* = 4.9 Hz, 1H); **^{13}C-NMR (100MHz, DMSO-d^{6}):** δ_C 169.3, 158.4, 147.5, 138.1, 134.9, 124.0, 118.8; **IR:** 3095, 2918, 2848, 1548, 1505, 1472, 1380, 1339, 1306, 1201, 1187, 1011, 853, 810, 768, 742, 727, 682, 630 cm^{-1}; **HRMS (ESI)** *m/z* [M+H]$^{+}$ calcd for $C_{10}H_8N_3O_2S$ 234.0332; Found 234.0327.

2-Methylsulfonyl-4-methoxypyrimidine (4b)

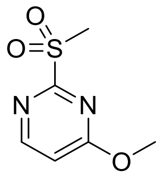

Chemical Formula: $C_6H_8N_2O_3S$
Molecular Weight: 188,20

Procedure C was followed using **2b** (70 mg, 0.45 mmol, 1 eq.) as substrate. The reaction was stirred for 20h. The crude was purified by column chromatography on silica gel (100% DCM to 94:6 DCM/EA) to afford product **4b** (62 mg, 0.33 mmol, 73 %) as a white solid. **Mp** = 60 °C; **Rf =** 0.3 (96:4 DCM/EA); **^{1}H-NMR (400MHz, DMSO-d^{6}):** δ_H 8.74 (d, *J* = 5.8 Hz, 1H), 7.25 (d, *J* = 5.8 Hz, 1H), 4.03 (s, 3H), 3.40 (s, 3H); **^{13}C-NMR (100MHz, $CDCl_3$):** δ_C 170.1, 165.0, 158.6, 111.2, 54.9, 38.9; **IR:** 3040, 2939, 1717, 1578, 1540, 1474, 1407, 1355, 1324, 1298, 1135, 1011, 983, 973, 958, 855, 757, 539; **HRMS (ESI)** *m/z* [M+Na]$^{+}$ calcd for $C_6H_8N_2NaO_3S$ 211.0148; Found 211.0147.

2-Methylsulfonyl-4-methyl pyrimidine (4c)

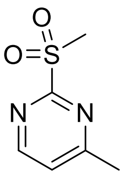

Chemical Formula: $C_6H_8N_2O_2S$
Molecular Weight: 172,20

Procedure C was followed using **2c** (106 mg, 0.76 mmol) as substrate. The reaction was stirred for 44h. The crude was purified by column chromatography on silica gel (100% DCM) to afford product **4c** (83 mg, 0.48 mmol, 63 %) as an oil. Analytical date are in accordance with those reported in literature.[35] **R_f (product)** = 0.1 (100% DCM); **^{1}H-NMR (400MHz, DMSO-d^6):** δ_H 8.90 (d, *J* = 5.1 Hz, 1H), 7.72 (d, *J* = 5.1 Hz, 1H), 3.38 (s, 3H), 2.61 (s, 3H)

2-(methylsulfonyl)pyrimidine-4-carboxylic acid (4d)

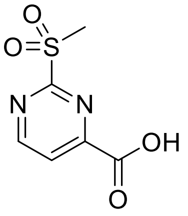

Chemical Formula: $C_6H_6N_2O_4S$
Molecular Weight: 202,18

2-Methylsulfanyl-4-carboxylic acid pyrimidine **2d** (70 mg, 0.41 mmol, 1 eq.) was dissolved in AcOH (1 mL, c = 0.4 mol/L). H_2O_2 35% w/w (0.40 mL, 8 eq) was added dropwise by syringe pump over a period of 5 min. After 44h, the reaction was diluted with distilled water and freeze-dried to obtain product **4d** (79 mg, 0.39 mmol, 95%) as a white solid without further purification. **R_f (product)** = 0.4 (1:1 DCM/hexane); **Mp** = 115-118 °C; **^{1}H-NMR (400MHz, DMSO-d^6):** δ_H 9.31 (d, *J* = 5.0 Hz, 1H), 8.26 (d, *J* = 5.0 Hz, 1H), 3.45 (s, 3H); **^{13}C-NMR (100MHz, DMSO-d^6):** δ_C 165.8, 164.0, 161.7, 157.1, 123.6, 39.6; **IR:** 2936, 1712, 1573, 1353, 1310, 1132, 1042, 952, 780, 717, 656, 537 cm^{-1}; **HRMS (ESI)** *m/z* $[M-H]^-$ calcd for $C_6H_5N_2O_4S$ 200.9976; Found 200.9979

2-Methylsulfonyl-4-phenylypyrimidine (4e)

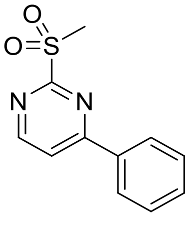

Chemical Formula: $C_{11}H_{10}N_2O_2S$
Molecular Weight: 234,27

Procedure C was followed using **2e** (95 mg, 0.47 mmol) as substrate. The reaction was stirred for 16h. The crude was purified by column chromatography on silica gel (8:2 DCM/hexane to 100% DCM) to afford product **4e** (109 mg, 0.46 mmol, 98 %) as a white solid. Analytical data are in accordance with

those reported in literature.[36] **R_f (product)** = 0.4 (1:1 DCM/hexane); **Mp** = 139 °C; **^{1}H-NMR (400MHz, DMSO-d^6):** δ_H 9.12 (d, *J* = 5.4 Hz, 1H), 8.40 (d, *J* = 5.4 Hz, 1H), 8.33-8.29 (m, 2H), 7.68-7.60 (m, 3H, H10), 3.50 (s, 3H)

2-Methylsulfonyl-4-aminopyrimidine (4g)

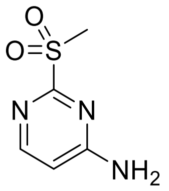

Chemical Formula: $C_5H_7N_3O_2S$
Molecular Weight: 173,19

Procedure C was followed using **2g** (101 mg, 0.71 mmol, 1 eq.) as substrate. The reaction was stirred for 16h. The crude was purified by column chromatography on silica gel (7:3 EA/hexane) to afford product **4g** (36 mg, 0.21 mmol, 29 %) as a white solid. **R_f (product)** = 0.2 (7:3 EA/hexane); **Mp** = 166 °C; **^{1}H-NMR (400MHz, DMSO-d^6):** δ_H 8.19 (d, *J* = 5.9 Hz, 1H), 7.64 (br. s, 2H), 6.59 (d, *J* = 5.9 Hz, 1H), 3.24 (s, 3H); **^{13}C-NMR (100MHz, DMSO-d^6):** δ_C 165.6, 164.2, 155.0, 107.5, 38.7; **IR:** 3417, 3308, 3203, 2926, 1630, 1588, 1528, 1500, 1283, 1213, 1130, 963, 839, 764, 660, 536 cm^{-1}; **HRMS (ESI)** *m/z* [M+Na]$^+$ calcd for $C_5H_7N_3NaO_2S$ 196.0151; Found 196.0150.

5-Chloro-2-(methylsulfonyl)-4-pyrimidinecarboxylic acid (4h)

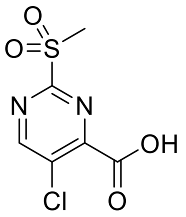

Chemical Formula: $C_6H_5ClN_2O_4S$
Molecular Weight: 236,63

Commercially available 5-Chloro-2-(methylthio)-4-pyrimidinecarboxylic acid **2h** (203 mg, 0.99 mmol, 1 eq.) was dissolved in AcOH (1.5 mL). H_2O_2 30 % w/w (0.44 mL, 3.91 mmol, 4 eq.) was added dropwise over a period of 30 min with a syringe pump at room temperature under argon atmosphere. After 48h, the reaction was diluted with water and freeze dried. Product **4h** (230 mg, 0.97 mmol, 98 %) was obtained as a white powder and used without further purification. **Mp** = 172-173°C; **^{1}H-NMR (400MHz, DMSO-d^6):** δ_H 9.36 (s, 1H), 3.43 (s, 3H); **^{13}C-NMR (100MHz, DMSO-d^6):** δ_C 163.5, 162.9, 160.5, 156.8, 129.7, 39.4; **IR:** 2884, 1730, 1570, 1434, 1396, 1307, 1280, 1196, 1159, 1144, 1066, 974, 894, 777, 660, 645, 573 cm^{-1}; **HRMS (ESI)** *m/z* [M+Na]$^+$ calcd for $C_6H_5ClN_2NaO_4S$ 258.9551; Found 258.9542.

2-(methylsulfonyl)pyrimidine-4-carboxamide (4j)

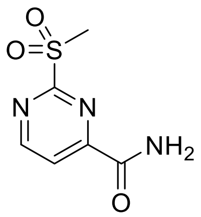

Chemical Formula: $C_6H_7N_3O_3S$
Molecular Weight: 201,20

2-Methylsulfanyl-4-carboxamide pyrimidine **2j** (83 mg, 0.49 mmol, 1 eq.) was dissolved in AcOH (1 mL, c = 0.5 mol/L). H_2O_2 30% w/w (0.22 mL, 4 eq) was added dropwise by syringe pump over a period of 20 min. After 48h, the reaction was diluted with distilled water and freeze-dried to obtain product **4j** (98 mg, 0.487 mmol, 99%) as a white solid without further purification. **R_f (product)** = 0.3 (8:2 EA/DCM); **Temperature degradation** = 208 °C; **^{1}H-NMR (400MHz, DMSO-d^6):** δ_H 9.30 (d, *J* = 5.0 Hz, 1H), 8.52 (br. s, 1H), 8.25 (d, *J* = 5.0 Hz, 1H), 8.18 (br s., 1H), 3.56 (s, 3H); **^{13}C-NMR (100MHz, DMSO-d^6):** δ_C 165.0, 163.3, 161.7, 158.3, 121.3, 38.9; **IR:** 3392, 3192, 3009, 2928, 1695, 1575, 1533, 1456, 1395, 1298, 1206, 1127, 1043, 974, 880, 773, 623, 549, 532 cm^{-1}; **HRMS (ESI)** *m/z* [M+Na]$^+$ calcd for $C_6H_7N_3NaO_3S$ 224.0100; Found 224.0103.

2-Methylsulfonyl-4-methylester pyrimidine (4k)

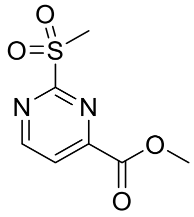

Chemical Formula: $C_7H_8N_2O_4S$
Molecular Weight: 216,21

Procedure C was followed using **2k** (50 mg, 0.27 mmol, 1 eq.) as substrate. The reaction was stirred for 22h. The crude was purified by column chromatography on silica gel (95:5 DCM/EA) to afford product **4k** (50 mg, 0.23 mmol, 85 %) as a white solid. **R_f (product)** = 0.55 (7:3 DCM/EA); **Mp** = 80 °C; **^{1}H-NMR (400MHz, DMSO-d^6):** δ_H 9.35 (d, *J* = 5.0 Hz, 1H), 8.30 (d, *J* = 5.0 Hz, 1H), 3.97 (s, 3H), 3.45 (s, 3H); **^{13}C-NMR (100MHz, DMSO-d^6):** δ_C 165.7, 163.0, 161.8, 155.8, 123.7, 53.4, 39.2; **IR:** 2924, 2851, 1734, 1573, 1542, 1311, 1203, 1133, 957, 737, 670, 541; **HRMS (ESI)** *m/z* [M+Na]$^+$ calcd for $C_7H_8N_2NaO_4S$ 239.0097; Found 239.0095.

2-(methylsulfonyl)-4-(trifluoromethyl)pyrimidine (4l)

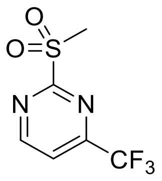

Chemical Formula: $C_6H_5F_3N_2O_2S$
Molecular Weight: 226,17

Procedure C was followed using **2l** (104 mg, 0.54 mmol) as substrate. The reaction was stirred for 21h. The crude was purified by column chromatography on silica gel (7:3 DCM/hexane to 100% DCM) to afford product **4l** (111 mg, 0.49 mmol, 91 %) as a white solid. **R_f (product)** = 0.3 (9:1 DCM/EA); **Mp** = 82 °C; **^{1}H-NMR (400MHz, DMSO-d^6):** δ_H 9.48 (d, *J* = 5.0 Hz, 1H), 8.40 (d, *J* = 5.0 Hz, 1H), 3.48 (s, 3H); **^{13}C-NMR (100MHz, DMSO-d^6):** δ_C 162.7, 163.0, 154.9 (q, *J* = 34 Hz), 120.9 (q, *J* = 3 Hz), 120.0 (q, *J* = 275 Hz), 39.1; **^{19}F-NMR (376MHz, DMSO-d^6):** δ_F -68.1; **IR:** 3016, 2936, 2362, 1339, 1316, 1139, 1119, 955, 867, 774, 661, 538 cm^{-1}; **HRMS (ESI)** *m/z* $[M+H]^+$ calcd for $C_6H_6F_3N_2O_2S$ 227.0097; Found 227.0095.

2-Methylsulfonyl-5-aminopyrimidine (4m)

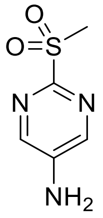

Chemical Formula: $C_5H_7N_3O_2S$
Molecular Weight: 173,19

Procedure C was followed using **2m** (182 mg, 1.29 mmol, 1 eq.) as substrate. The reaction was stirred for 16h. The crude was purified by column chromatography on silica gel (100% DCM) to afford product **4m** (105 mg, 0.60 mmol, 46 %) as a white solid. **Mp** = 138 °C; **^{1}H-NMR (400MHz, DMSO-d^6):** δ_H 8.19 (s, 2H), 6.46 (br. s, 2H), 3.22 (s, 3H); **^{13}C-NMR (100MHz, DMSO-d^6):** δ_C 152.8, 145.0, 140.8, 39.9; **IR:** 3433, 3352, 3235, 2920, 2850, 1635, 1571, 1420, 1297, 1207, 1119, 961, 779, 748 cm^{-1}; **HRMS (ESI)** *m/z* $[M+Na]^+$ calcd for $C_5H_7N_3NaO_2S$ 192.0151; Found 192.0152.

2-Methylsulfonyl-5-methoxypyrimidine (4n)

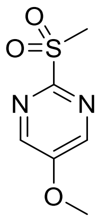

Chemical Formula: $C_6H_8N_2O_3S$
Molecular Weight: 188,20

Procedure C was followed using **2n** (4.54 g, 29.1 mmol, 1 eq.) as reported in literature from Jacobsen et al.[28] The reaction was stirred for 16h. The crude was purified by column chromatography on silica gel

(9:1 DCM/EA) to afford product **4n** (3.51 g, 18.7 mmol, 65 %) as a white solid. Data were in accordance with those reported in literature.[28]

2-Methylsulfonyl-5-methylpyrimidine (4o)

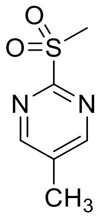

Chemical Formula: $C_6H_8N_2O_2S$
Molecular Weight: 172,20

Procedure C was followed using **2o** (210 mg, 1.50 mmol, 1 eq.) as substrate. The reaction was stirred for 16h. The crude was purified by column chromatography on silica gel (100% DCM) to afford product **4o** (199 mg, 1.16 mmol, 77 %) as a colourless oil. **^{1}H-NMR (400MHz, CDCL3):** δ_H 8.75 (s, 2H), 3.34 (s, 3H), 2.46 (s, 3H); **^{13}C-NMR (100MHz, CDCl3):** δ_C 164.0, 158.6, 134.5, 39.6, 16.0; **IR:** 3040, 3015, 2934, 1559, 1399, 1308, 1195, 1124, 969, 785, 758, 640 cm^{-1}; **HRMS (ESI)** *m/z* [M+H]$^+$ calcd for $C_6H_9N_2O_2S$ 173.0379; Found 173.0379.

2-Methyl sulfonyl 5-phenyl pyrimidine (4p)

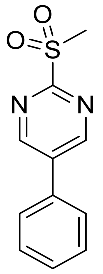

Chemical Formula: $C_{11}H_{10}N_2O_2S$
Molecular Weight: 234,27

Procedure C was followed using **2p** as substrate (70 mg, 0.35 mmol, 1 eq.). The reaction was stirred for 21h. The crude was purified by column chromatography on silica gel (100% DCM) to afford product **4p** (75 mg, 0.32 mmol, 91 %) as a white solid. **Mp =** 180 °C; **^{1}H-NMR (400MHz, DMSO-d^6):** δ_H 9.41 (s, 2H), 7.94-7.89 (m, 2H), 7.63-7.53 (m, 3H, H10), 3.45 (s, 3H); **^{13}C-NMR (100MHz, DMSO-d^6):** δ_C 164.2, 156.4, 135.5, 132.5, 129.8, 129.4, 127.6, 39.2; **IR:** 3059, 2924, 1317, 1217, 1208, 1193, 1117, 962, 760, 743, 693, 642, 549 cm^{-1}; **HRMS (ESI)** *m/z* [M+Na]$^+$ calcd for $C_{11}H_{10}N_2NaO_2S$ 257.0355; Found 257.0359.

2-Methylsulfonylpyrimidine (4q)

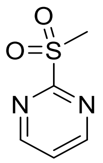

Chemical Formula: $C_5H_6N_2O_2S$
Molecular Weight: 158,18

Procedure C was followed using **2q** (210 mg, 1.66 mmol) as reported in literature by Kamijo et al. [37]. The reaction was stirred for 16h. The crude was purified by column chromatography on silica gel (100% DCM) to afford product **4q** (178 mg, 1.13 mmol, 68 %) as a white solid. Analytical data are in accordance with those reported in literature.[37] **Mp** = 68-70 °C; **^{1}H-NMR (400MHz, DMSO-d^6):** δ_H 9.10 (d, *J* = 4.9 Hz, 2H), 7.86 (t, *J* = 4.9 Hz, 1H), 3.42 (s, 3H); **^{13}C-NMR (100MHz, DMSO-d^6):** δ_C 165.6, 159.1, 124.7, 39.0; **HRMS (ESI)** *m/z* $[M+H]^+$ calcd for $C_5H_7N_2O_2S$ 159.0223; Found 159.0222.

2-Methylsulfonyl-5-fluoropyrimidine (4r)

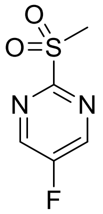

Chemical Formula: $C_5H_5FN_2O_2S$
Molecular Weight: 176,17

Procedure C was followed using **2r** (374 mg, 2.59 mmol, 1 eq.) as substrate. The reaction was stirred for 16h. The crude was purified by column chromatography on silica gel (100% DCM) to afford product **4r** (395 mg, 2.22 mmol, 86 %) as a colourless oil. **R_f (product)** = 0.30 (7:3 hexane/acetone); **^{1}H-NMR (400MHz, DMSO-d^6):** δ_H 9.19 (d, *J* = 0.9 Hz, 2H), 3.43 (s, 3H); **^{13}C-NMR (100MHz, DMSO-d^6):** δ_C 161.1 (d, *J* = 5 Hz, C2), 158.3 (d, *J* = 268 Hz, C5), 147.1 (d, *J* = 22 Hz, C6 and C4), 39.7 (C7); **^{19}F-NMR (376MHz, DMSO-d^6):** δ_F -129.9; **IR:** (neat) 1720, 1598, 1384, 1298, 1124, 959, 763, 549 cm^{-1}; **HRMS (ESI)** *m/z* $[M+H]^+$ calcd for $C_5H_6FN_2O_2S$ 177.0129; Found 177.0130.

2-Methylsulfonyl-5-chloropyrimidine (4s)

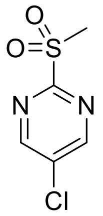

Chemical Formula: $C_5H_5ClN_2O_2S$
Molecular Weight: 192,62

Procedure C was followed using **2s** (501 mg, 3.13 mmol, 1 eq.) as substrate. The reaction was stirred for 16h. The crude was purified by column chromatography on silica gel (99:1 DCM/EA) to afford product **4s** (434 mg, 2.26 mmol, 72 %) as a white solid. Data are in accordance with those reported in literature.[38]

Rf (product) = 0.40 (99:1 DCM/EA); **Mp** = 125 °C; **^{1}H-NMR (400MHz, DMSO-d^6):** δ_H 9.24 (s, 2H), 3.43 (s, 3H)

5-Bromo-2-(methylsulfonyl)pyrimidine (4t)

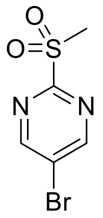

Chemical Formula: $C_5H_5BrN_2O_2S$
Molecular Weight: 237,07

Procedure C was followed using **2t** (516 mg, 2.50 mmol, 1 eq.) as substrate. The reaction was stirred for 16h. The crude was purified by column chromatography on silica gel (100% DCM) to afford product **4t** (405 mg, 1.71 mmol, 68 %) as a white solid. Analytical date are in accordance with those reported in literature.[35] **Mp** = 133 °C; **^{1}H-NMR (400MHz, DMSO-d^6):** δ_H 9.31 (s, 2H), 3.42 (s, 3H)

2-Methylsulfonyl-5-iodopyrimidine (4u)

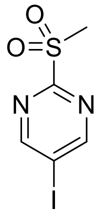

Chemical Formula: $C_5H_5IN_2O_2S$
Molecular Weight: 284,07

Procedure C was followed using **2u** (62 mg, 0.25 mmol, 1 eq.) as substrate. The reaction was stirred for 14h. The crude was purified by column chromatography on silica gel (9:1 DCM/hexane) to afford product **4u** (61 mg, 0.21 mmol, 84 %) as a yellow powder. **Mp** = 56 °C; **^{1}H-NMR (400MHz, DMSO-d^6):** δ_H 9.37 (s, 2H), 3.95 (s, 3H); **^{13}C-NMR (100MHz, DMSO-d^6):** δ_C 164.2, 164.0, 98.6, 39.2; **IR:** 3031, 2927, 1540, 1394, 1312, 1215, 1127, 1008, 958, 793, 628, 549 cm^{-1}; **HRMS (ESI)** *m/z* $[M+H]^+$ calcd for $C_5H_6IN_2O_2S$ 284.9189; Found 284.9194.

2-Methylsulfonylpyrimidine-5-carboxylic acid (4v)

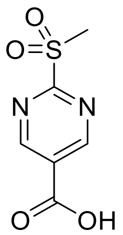

Chemical Formula: $C_6H_6N_2O_4S$
Molecular Weight: 202,18

Procedure C was followed using **2v** (83 mg, 0.49 mmol) as substrate. The reaction was stirred for 16h. The reaction was concentrated and the crude was dissolved in 2 mL of MeCN and water, injected on a Biotage Sfar C18 Duo 12 g cartridge running a 0 to 20 % MeCN gradient over 20 min to afford product **4v** (24 mg, 0.12 mmol, 24 %) as a white solid, after freeze drying. **Mp** = 138 °C; **^{1}H-NMR (400MHz, DMSO-d^6):** δ_H 9.43 (s, 2H), 3.46 (s, 3H); **^{13}C-NMR (100MHz, DMSO-d^6):** δ_C 167.2, 163.7, 159.6, 159.2, 39.1; **IR:** 3042, 3016, 2934, 1703, 1576, 1557, 1395, 1383, 1301, 1264, 1147, 1125, 1034, 966, 924, 771, 635 cm^{-1}; **HRMS (ESI)** *m/z* [M+H]$^+$ calcd for $C_6H_7N_2O_4S$ 203.0121; Found 203.0119

2-Methylsulfonyl-5-nitropyrimidine (4w)

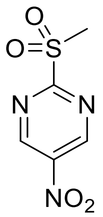

Chemical Formula: $C_5H_5N_3O_4S$
Molecular Weight: 203,17

Procedure C was followed using **2w** (69 mg, 0.40 mmol, 1 eq.) as substrate and mCPBA (558 mg, 2.43 mmol, 6 eq.). The reaction was stirred for 5 days. The crude was purified by column chromatography on silica gel (100% DCM) to afford product **4w** (30 mg, 0.15 mmol, 37 %) as a pale yellow solid. **Mp** = 113 °C; **^{1}H-NMR (400MHz, DMSO-d^6):** δ_H 9.98 (s, 2H), 3.80 (s, 3H); **^{13}C-NMR (100MHz, DMSO-d^6):** δ_C 170.2, 156.4, 126.6, 40.6; **IR:** 3077, 3040, 2940, 1596, 1574, 1530, 1414, 1352, 1281, 1178, 972, 903, 780, 734, 544 cm^{-1}; **HRMS (GC-EIMS)** *m/z* [M] calcd for $C_5H_6N_3O_4S$ 202.9995; Found 202.9997.

2-(Methylsulfonyl)-5-(trifluoromethyl)pyrimidine (4x)

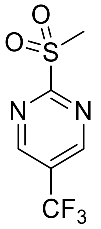

Chemical Formula: $C_6H_5F_3N_2O_2S$
Molecular Weight: 226,17

Procedure A was followed using 2-chloro-5-(trifluoromethyl)pyrimidine **2x** (108 mg, 0.59 mmol) as substrate and 1.5 eq. of sodium methanethiolate (66 mg, 0.89 mmol). The reaction was stirred for 19h. The crude solution of intermediate **2x** was evaporated on rotary evaporator under 10°C and used in the next step without further purification following procedure C. The reaction was stirred for 45h. The reaction was quenched by addition of $Na_2S_2O_3$ 10% solution. The aqueous phase was extracted with DCM (x3). The combined organic layers were washed with brine (x1), dried over $MgSO_4$, filtered and concentrated under reduced pressure. The crude was purified by column chromatography on silica gel (6:4 DCM/hexane to 100% DCM) to afford product **4x** (81 mg, 0.36 mmol, 61 %) as an oil. **R_f (XX045B)** = 0.65 (7:3 DCM/hexane), **R_f (XX045C)** = 0.40 (9:1 DCM/hexane); **^{1}H-NMR (400MHz, DMSO-d^6):** δ_H 9.59 (d, *J* = 0.8 Hz, 2H), 3.48 (s, 3H); **^{13}C-NMR (100MHz, DMSO-d^6):** δ_C 167.9, 157.1 (q, *J* = 3 Hz), 125.7 (q, *J* = 34 Hz), 122.2 (q, *J* = 273Hz), 39.1; **^{19}F-NMR (376MHz, DMSO-d^6):** δ_F -60.1; **IR:** 3060, 2939, 1699, 1594, 1563, 0321, 1141, 1121, 1087, 1020, 965, 793, 748, 718, 640, 554 cm^{-1}; **HRMS (ESI)** *m/z* [M+H]$^+$ calcd for $C_6H_6F_3N_2O_2S$ 227.0097; Found 227.0095

Methyl 2-methylsulfonylpyrimidine-5-carboxylate (4y)

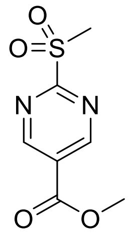

Chemical Formula: $C_7H_8N_2O_4S$
Molecular Weight: 216,21

Procedure C was followed using **2y** (277 mg, 1.50 mmol, 1 eq.) as substrate. The reaction was stirred for 16h. The crude was purified by column chromatography on silica gel (95:5 DCM/EA) to afford product **4y** (253 mg, 1.17 mmol, 78 %) as a white solid. **Mp** = 128 °C; **^{1}H-NMR (400MHz, DMSO-d^6):** δ_H 9.48 (s, 2H), 3.96 (s, 3H), 3.47 (s, 3H); **^{13}C-NMR (100MHz, DMSO-d^6):** δ_C 167.5, 162.8, 159.6, 126.1, 53.1, 39.0; **IR:** 3050, 3019, 2967, 2933, 1733, 1577, 1559, 1403, 1380, 1311, 1260, 1133, 1118, 797, 772, 638, 563 cm^{-1}; **HRMS (ESI)** *m/z* [M+H]$^+$ calcd for $C_7H_9N_2O_4S$ 217.0278; Found 217.0280.

2-(*t*-butylsulfonyl)pyrimidine (5a)

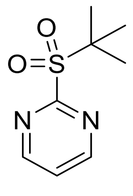

Chemical Formula: $C_8H_{12}N_2O_2S$
Molecular Weight: 200,26

Procedure C was followed using 2-(*t*-butylthio)pyrimidine **3a** (52 mg, 0.31 mmol, 1 eq.) as substrate. The reaction was stirred for 16h. The crude was purified by column chromatography on silica gel (5:95 to 1:9 EA/DCM) to afford 2-(*t*-butylsulfonyl)pyrimidine **5a** (54 mg, 0.27 mmol, 87 %) as a white solid. **R_f (product)** = 0.4 (1:9 EA/DCM); **Mp =** 58 °C; **^{1}H-NMR (400MHz, DMSO-d^6):** δ_H 9.12 (d, *J* = 4.9 Hz, 2H), 7.87 (t, *J* = 4.9 Hz, 1H), 1.35 (s, 9H); **^{13}C-NMR (100MHz, DMSO-d^6):** δ_C 163.3, 159.0, 124.6, 59.8, 23.1; **IR:** 2982,

2933, 1561, 1385, 1307, 1206, 1109, 990, 755, 650, 625, 578 cm^{-1}; **HRMS (ESI)** *m/z* $[M+Na]^+$ calcd for $C_8H_{12}N_2NaO_2S$ 223.0512; Found 223.0507.

4-(2-Pyrimidinylsulfonyl)benzenamine (5b)

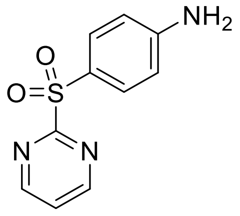

Chemical Formula: $C_{10}H_9N_3O_2S$
Molecular Weight: 235,26

2-(4-Nitro-benzenesulfonyl)-pyrimidine **3b** (70 mg, 0.26 mmol, 1 eq.) was dissolved in 4 mL of a 1:1 DCM/EA mixture under argon atmosphere. The reaction was cooled to 0 °C. $SnCl_2.2H_2O$ (298 mg, 1.32 mmol, 5 eq.) was added portionwise over a period of 10 min. The reaction was stirred at room temperature for 23h under argon atmosphere. The reaction was then concentrated and partitioned between EA and $NaHCO_3$ sat. solution. The aqueous phase was extracted with EA (x4). The combined organic layers were washed with H_2O (x1), brine (x1), dried over $MgSO_4$, filtered and concentrated under reduced pressure. The crude was purified by column chromatography on silica gel (Eluent 1:1 DCM/EA) affording **5b** (50 mg ,0.21 mmol, 81%) as a white solid. **Mp** = 200-201 °C; **R_f (product)** = 0.30 (1:1 DCM/EA); **^{1}H-NMR (400MHz, DMSO-d^6):** δ_H 8.98 (d, *J* = 4.8 Hz, 2H), 7.71 (t, *J* = 4.8 Hz, 1H), 7.60-7.55 (m, 2H), 6.67-6.63 (m, 2H), 6.31 (br s, 2H); **^{13}C-NMR (100MHz, DMSO-d^6):** δ_C 167.0, 159.0, 154.5, 131.2, 123.7, 120.9, 112.8; **IR:** 3454, 3348, 3245, 3224, 1636, 1593, 1564, 1382, 1314, 1207, 1139, 1081, 828, 732, 679, 588, 546 cm^{-1}; **HRMS (ESI)** *m/z* $[M+Na]^+$ calcd for ; Found $C_{10}H_9N_3NaO_2S$ 258.0308; Found 258.0305.

2-(*n*-butylsulfonyl)pyrimidine (5c)

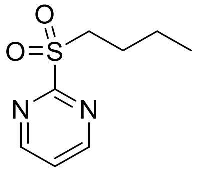

Chemical Formula: $C_8H_{12}N_2O_2S$
Molecular Weight: 200,26

Procedure C was followed using 2-(*n*butylthio)pyrimidine **3c** (81 mg, 0.48 mmol, 1 eq.) as substrate. The reaction was stirred for 18 hours. The crude was purified by column chromatography on silica gel (15:85 EA:Hexane) to afford 2-(*n*butylsulfonyl)pyrimidine **5c** (86 mg, 0.43 mmol, 91%) as an oil. **R_f (product) =** 0.3 (15:85 EA/hexane); **^{1}H-NMR (400MHz, DMSO-d^6):** δ_H 9.10 (d, *J* = 4.9 Hz, 2H), 7.86 (t, *J* = 4.9 Hz, 1H), 3.61-3.56 (m, 2H), 1.70-1.61 (m, 2H), 1.41 (m, 2H), 0.88 (t, *J* = 7.4 Hz, 3H); **^{13}C-NMR (100MHz, DMSO-d^6):** δ_C 165.0, 159.2, 124.7, 50.1, 23.8, 20.9, 13.4; **IR:** 2960, 2874, 1563, 1315, 1121 cm^{-1}; **HRMS (ESI)** *m/z* $[M+Na]^+$ calcd for $C_8H_{12}N_2NaO_2S$ 223.0512; Found 223.0514.

2- (phenylsulfonyl) pyrimidine (5d)

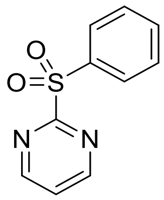

Chemical Formula: $C_{10}H_8N_2O_2S$
Molecular Weight: 220,25

Procedure C was followed using 2-(phenylthio)pyrimidine **3d** (70 mg, 0.37 mmol, 1 eq.) as reported in literature from Guilbaud et al.[39] The reaction was stirred for 18 hours. The crude was purified by column chromatography on silica gel (1:1 EA: Hexane) to afford 2-(phenylsulfonyl)pyrimidine **5d** (55 mg, 0.25 mmol, 67%) as a white solid. Analytical data are in accordance with those reported in literature.[39]

2-((2,2,2-trifluoroethyl)sulfonyl)pyrimidine (5e)

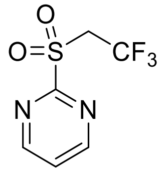

Chemical Formula: $C_6H_5F_3N_2O_2S$
Molecular Weight: 226,17

To a solution of commercially available 2-mercaptopyrimidine (100 mg, 0.89 mmol, 1 eq.) in anhydrous DMF (2 mL) was added K_2CO_3 (246 mg, 1.78 mmol, 2 eq.). The solution was stirred for 10 min at room temperature under argon atmosphere. Then 1,1,1-trifluoro-2-iodoethane (0.18 mL, 1.78 mmol, 2 eq.) was added dropwise. The reaction was heated at 50 °C for 26h. The reaction was then diluted with H2O and EA. The aqueous phase was extracted x2 with EA. The combined organic layer was washed with H_2O (x2), brine (x1), dried over $MgSO_4$, filtered and concentrated under reduced pressure. The residue was dissolved in DCM (5 mL). mCPBA (1.18 g, 5.25 mmol, 6 eq.) was dissolved in DCM (5mL). The solution of intermediate XX056B was added dropwise to the mCPBA solution. The reaction was stirred at room temperature under argon atmosphere for 5 days. The mixture was filtered through a cotton pad and rinsed with DCM. The filtrate was washed with $NaHCO_3$ sat. solution (x2), H_2O (x1), dried over $MgSO_4$, filtered and evaporated to dryness. The crude was purified by flash column chromatography on silica gel (eluent 100% DCM) to afford product **5e** (47 mg, 0.21 mmol, 24 %) as a white solid. **Mp** = 91-92 °C; **R_f (product)** = 0.25 (100% DCM); **^{1}H-NMR (400MHz, DMSO-d^6):** δ_H 9.16 (d, *J* = 4.9 Hz, 2H), 7.94 (t, *J* = 4.9 Hz, 1H), 5.21 (q, *J* = 9.9 Hz, 2H); **^{13}C-NMR (100MHz, DMSO-d^6):** δ_C 164.2, 159.5, 125.3, 123.4, 52.0 (q, *J* = 30 Hz); **^{19}F-NMR (376MHz, DMSO-d^6):** δ_F -58.8; **IR:** 3007, 2949, 1568, 1552, 1386, 1345, 1315, 1246, 1208, 1124, 1076, 991, 819, 667, 625, 575, 544 cm^{-1}; **HRMS (ESI)** *m/z* [M+Na]$^+$ calcd for ; Found $C_6H_5F_3N_2NaO_2S$ 248.9916; Found 248.9921.

2-(4-Nitro-benzenesulfonyl)-pyrimidine (5f)

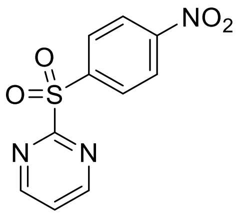

Chemical Formula: $C_{10}H_7N_3O_4S$
Molecular Weight: 265,24

2-((4-nitrophenyl)thio)pyrimidine **3f** (211 mg, 0.90 mmol, 1 eq.) was solubilised in EtOH (4.5 mL) and DCM (1 mL). $(NH_4)_6Mo_7O_{24}.6H_2O$ (446 mg, 0.36 mmol, 0.4 eq.) was added. Then H_2O_2 35% w/w (0.15 mL, 4 eq.) was added dropwise over a period of 5 min under argon atmosphere. Additional H_2O_2 and $(NH_4)_6Mo_7O_{24}.6H_2O$ were further added in portions every 12-24 h until full consumption of the starting material. The reaction was quenched after 6 days by dropwise addition of 10% aq. $Na_2S_2O_3$ until a persistent blue colour was observed. The aqueous phase was extracted with EA (x3). The combined organic layers were washed with H_2O (x2), brine (x1), dried over $MgSO_4$, filtered and concentrated under reduced pressure. The crude was purified by column chromatography on silica gel (eluent 100 % DCM) to afford 2-(4-nitro-benzenesulfonyl)-pyrimidine **5f** (182 mg, 0.69 mmol, 77 %) as a white solid. **Mp** = 225 °C; **R_f (product)** = 0.5 (95:5 DCM/EA); **^{1}H-NMR (400MHz, DMSO-d^6):** δ_H 9.06 (d, *J* = 4.9 Hz, 2H), 8.49-8.45 (m, 2H), 8.30-8.26 (m, 2H), 7.84 (t, *J* = 4.9 Hz, 1H); **^{13}C-NMR (100MHz, DMSO-d^6):** δ_C 165.1, 159.5, 151.0, 142.6, 130.9, 124.9, 124.7; **IR:** 3108, 3062, 2921, 2848, 1569, 1524, 1386, 1351, 1337, 1304, 1147, 763, 732, 605, 574 cm^{-1}; **HRMS (ESI)** *m/z* $[M+Na]^+$ calcd for ; Found $C_{10}H_7N_3NaO_4S$ 288.0049; Found 288.0045.

N-benzylpyrimidine-2-sulfonamide (5g)

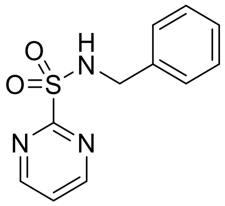

Chemical Formula: $C_{11}H_{11}N_3O_2S$
Molecular Weight: 249.3

N-benzylpyrimidine-2-sulfonamide **5g** was prepared following the procedure from Wright et al.[1] starting from 2-mercaptopyrimidine **6** (200 mg, 1.78 mmol, 1 eq.). The crude was purified by column chromatography on silica gel (9:1 to 8:2 DCM/EA) to afford product **5g** (26 mg, 0.104 mmol, 6 %) as a white solid. Data are in accordance with those reported in literature.[1] **^{1}H-NMR (400MHz, $CDCl_3$)** δ_H 8.88 (d, *J* = 4.9 Hz, 2H), 7.47 (t, *J* = 4.9 Hz, 1H), 7.31-7.25 (m, 5H), 5.20 (br s, 1H), 4.43 (d, *J* = 6.2 Hz, 2H)

Perfluorophenyl pyrimidine-2-sulfonate (5h)

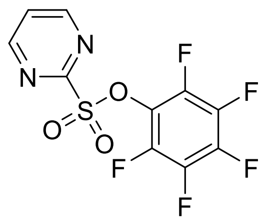

Chemical Formula: $C_{10}H_3F_5N_2O_3S$
Molecular Weight: 326,2

Perfluorophenyl pyrimidine-2-sulfonate **5h** was prepared following the procedure from Bornholdt et al.[2] starting from 2-mercaptopyrimidine **6** (200 mg, 1.78 mmol, 1 eq.). The crude was purified by column chromatography on silica gel (1:1 to 6:4 DCM/hex) to afford product **5h** (257 mg, 0.788 mmol, 44 %) as a white solid. Data are in accordance with those reported in literature.[2] **^{1}H-NMR (400MHz, CDCl₃)** δ_H 9.04 (d, *J* = 4.8 Hz, 2H), 7.71 (t, *J* = 4.8 Hz, 1H); **^{19}F-NMR (376MHz, CDCl₃)** δ_F -150.8 (d, *J* = 18 Hz, 2F), -154.8 (t, *J* = 22 Hz, 1F), -160.9 (dd, *J* = 21, 17 Hz, 2F)

5-(methylsulfonyl)-1-phenyl-1H-tetrazole (7)

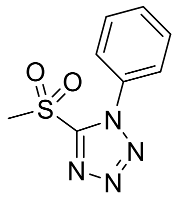

Chemical Formula: $C_8H_8N_4O_2S$
Molecular Weight: 224.2

5-(methylsulfonyl)-1-phenyl-1H-tetrazole **7** was prepared in two steps following procedures from Toda et al.,[3] purified by column chromatography on silica gel (9:1 DCM/hexane to 9:1 DCM/EA) and obtained as a white solid (115 mg, 0.515 mmol). Spectral data were in accordance with those reported in literature.[3] **^{1}H-NMR (400MHz, DMSO-d^6):** δ_H 7.76-7.63 (m, 5H), 3.67 (s, 3H)

2-(methylsulfonyl)-5-phenyl-1,3,4-oxadiazole (8)

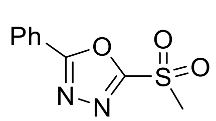

Chemical Formula: $C_9H_8N_2O_3S$
Molecular Weight: 224,2

2-(methylsulfonyl)-5-phenyl-1,3,4-oxadiazole **8** was prepared in two steps following procedures from Toda et al.,[3] purified by column chromatography on silica gel (2:8 EA/hexane), and obtained as a white solid (50 mg, 0.223 mmol). Spectral data were in accordance with those reported in literature.[3] **^{1}H-NMR (400MHz, CDCl₃):** δ_H 8.16-8.12 (m, 2H), 7.67-7.61 (m, 1H), 7.59-7.54 (m, 2H), 3.53 (s, 3H, CH_3)

2-(methylsulfonyl)-6-nitrobenzo[d]thiazole (9)

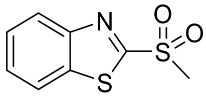

Chemical Formula: $C_8H_7NO_2S_2$
Molecular Weight: 213,27

Procedure C was followed using the commercially available 2-(methylsulfanyl)-6-nitrobenzo[d]thiazole as substrate (200 mg, 1.10 mmol). The reaction was stirred for 20h. The crude was purified by column chromatography on silica gel (6:4 DCM/Hexane) to afford product **9** (66 mg, 0.31 mmol, 28 %) as a beige crystalline solid. Data were in accordance with those reported in literature.[40] **^{1}H-NMR (400MHz, $CDCl_3$)** δ_H 8.25-8.19 (m, 1H), 8.05-8.00 (m, 1H), 7.68-7.57 (m, 2H), 3.42 (s, 3H)

2-(methylsulfonyl)-6-nitrobenzo[d]thiazole (10)

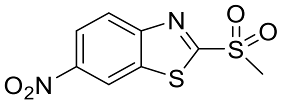

Chemical Formula: $C_8H_6N_2O_4S_2$
Molecular Weight: 258,27

2-(methylsulfonyl)-6-nitrobenzo[d]thiazole **10** was prepared in two steps following procedures from Tang et al.[41] and Motiwala et al.,[4] purified by flash chromatography on silica gel (100% DCM), and obtained as as a white solid (93 mg, 0.36 mmol). Spectral data were in accordance with those reported in literature.[4] **^{1}H-NMR (400MHz, $CDCl_3$)** δ_H 8.98 (dd, *J* = 2.2, 0.4 Hz, 1H), 8.51 (dd, *J* = 9.1, 2.3 Hz, 1H), 8.34 (dd, *J* = 9.1, 0.4 Hz, 1H), 3.47 (s, 3H)

4,6-dimethyl-2-(methylsulfonyl)-3-nitropyridine (12)

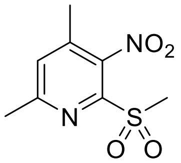

Chemical Formula: $C_8H_{10}N_2O_4S$
Molecular Weight: 230,24

4,6-dimethyl-2-(methylsulfonyl)-3-nitropyridine **12** was prepared in two steps following procedures from Zambaldo et al.,[42] purified by flash chromatography on silica gel (100% DCM), and obtained as a white solid (72 mg, 0.31 mmol). Spectral data were in accordance with those reported in literature.[42] **^{1}H-NMR (400MHz, $CDCl_3$)** δ_H 7.37 (br s, 1H), 3.31 (s, 3H), 2.66 (s, 3H), 2.40 (d, *J* = 0.7 Hz, 3H)

4,6-dimethyl-2-(methylsulfonyl)-3-nitropyridine (12)

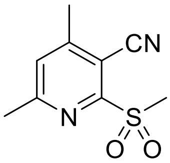

Chemical Formula: $C_9H_{10}N_2O_2S$
Molecular Weight: 210,25

4,6-dimethyl-2-(methylsulfonyl)-3-cyanopyridine **11** was prepared in two steps following procedures from Zambaldo et al.,[42] purified by flash chromatography on silica gel (100 to 95:5 DCM/EA), and obtained as a white solid (98 mg, 0.47 mmol, 96%). Spectral data were in accordance with those reported in literature.[42] **^{1}H-NMR (400MHz, $CDCl_3$)** δ_H 7.37-7.36 (m, 1H), 3.36 (s, 3H), 2.65 (d, *J* = 0.4Hz, 3H), 2.64 (d, *J* = 0.7 Hz, 3H)

2-(Methylsulfonyl)pyrazine (18)

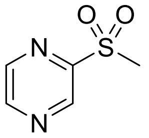

Chemical Formula: $C_5H_6N_2O_2S$
Molecular Weight: 158,18

Procedure C was followed using the commercially available 2-(Methylthio)pyrazine as substrate (102 mg, 0.81 mmol). The reaction was stirred for 41h. The crude was purified by column chromatography on silica gel (100% DCM to 9:1 DCM/EA) to afford product **18** (91 mg, 0.58 mmol, 72 %) as a white solid. Data are in accordance with literature.[43] **R_f (product) =** 0.55 (9:1 DCM/EA); **Mp** = 54 °C; **^{1}H-NMR (400MHz, DMSO-d^6):** δ_H 9.25 (d, *J* = 1.5 Hz, 1H), 9.05 (d, *J* = 2.4 Hz, 1H), 8.91 (dd, *J* = 2.4, 1.5 Hz, 1H), 3.36 (s, 3H)

2,4-dimethoxy-6-(methylsulfonyl)-1,3,5-triazine (19)

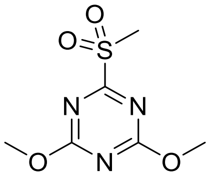

Chemical Formula: $C_6H_9N_3O_4S$
Molecular Weight: 219,22

Procedure A was followed using commercially available 2-Chloro-4,6-dimethoxy-1,3,5-triazine (210 mg, 1.20 mmol, 1 eq.) as a substrate, and following procedures described in literature.[44,45] The intermediate methylthioether was purified by column chromatography on silica gel (100% DCM) and obtained as a white solid (184 mg, 0.98 mmol, 82 %). Spectral date were in accordance with literature.[44,45] Attempts to oxidise the intermediate thioether to the corresponding sulfone using diverse oxidants led to degradation and complex mixtures during work-up and/or purification.

2-(methylsulfonyl)quinazoline (20)

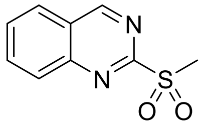

Chemical Formula: $C_9H_8N_2O_2S$
Molecular Weight: 208,24

Procedure A was followed using 2-chloroquinazoline (102 mg, 0.61 mmol, 1eq.) as substrate. Reaction was stirred for 15 h at room temperature under argon atmosphere. The crude residue was purified by flash chromatography on silica gel (8:2 DCM/Hexane) affording the intermediate 2-(methylthio)quinazoline (81 mg, 0.46 mmol, 75%). **R_f (product) =** 0.38 (9:1 DCM/hexane); **Mp** = 58°C; **^{1}H-NMR (400MHz, DMSO-d^6):** δ_H 9.40 (d, *J* = 0.7 Hz, 1H), 8.07 (ddd, *J* = 8.1, 1.5, 0.7 Hz, 1H), 7.96 (ddd, *J* = 8.6, 6.9, 1.5 Hz, 1H, H7), 7.84 (ddd, *J* = 8.6, 1.5, 0.71 Hz, 1H, H5), 7.63 (ddd, *J* = 8.1, 7.0, 1.1 Hz, 1H, H6), 2.62 (s, 1H, SMe); **^{13}C-NMR (100MHz, DMSO-d^6):** δ_C 168.4 (C2), 161.4 (C4), 150.5 (C9), 135.6 (C7), 128.6 (C8), 127.0 (C6), 126.7 (C5), 122.5 (C10), 14.2 (SMe); **HRMS (ESI)** *m/z* $[M+H]^+$ calcd for $C_9H_9N_2S$ 177.0481; Found 177.0484.

Procedure C was followed using 2-(methylthio)quinazoline (65 mg, 0.37 mmol, 1eq.) as substrate. Reaction was stirred for 16 h at room temperature under argon atmosphere. The crude residue was purified by flash chromatography on silica gel (100 to 98:2 DCM/EA) affording product **20** (69 mg, 0.33 mmol, 89%). Spectral data were in accordance with those reported in literature.[4] **^{1}H-NMR (400MHz, $CDCl_3$)** δ_H 9.58 (d, *J* = 0.8 Hz, 1H), 8.28-8.24 (m, 1H), 8.14-8.08 (m, 2H), 7.91-7.85 (m, 1H), 3.47 (s, 3H).

Acknowledgements

This work was supported by the Engineering and Physical Sciences Research Council (EP/S020799/1 New Investigator Award to MB). DD and RWW were supported by a Southampton University Vice-Chancellors Award. A.C.J. was supported by the German Research Foundation (DFG) grant JO 1473/1-3. D.-I.B. was supported by a Buchmann fellowship. The Structural Genomics Consortium is a registered charity (no. 1097737) that receives funds from Bayer AG, Boehringer Ingelheim, Bristol Myers Squibb, Genentech, Genome Canada through Ontario Genomics Institute [OGI-196], EU/EFPIA/OICR/McGill/KTH/Diamond Innovative Medicines Initiative 2 Joint Undertaking [EUbOPEN grant 875510], Janssen, Merck KGaA (a.k.a. EMD in Canada and US), Pfizer, and Takeda. We thank the staff at beamline X06SA of the Swiss Light Source for assistance during data collection. We also thank Dr Karthikeyan Radhakrishnan and Dr Alam Sarfaraz at the University of Bielefeld, Dr Marc Fiedler at the MRC-LMB Cambridge, Dr Patrick Duriez and Dr Leon Douglas at the University of Southampton for assistance in protein production.

Author contributions

MB directed the project. MB and MP designed the study. MP, SAG, MJ, RM, LH, NW synthesised the compounds and performed kinetics studies by NMR. MP, DD, RWW performed UV titrations. MP and MM performed DSF experiments. DL and SG performed DFT calculations. JH, MP, RM, LH and GJL performed protein MS experiments. DIB and ACJ performed protein crystallography experiments and solved the crystal structure of arylated p53. MP and MB wrote the manuscript with input from all authors.

Competing interests

The authors declare no competing interests.

References

(1) Wright, S. W.; Hallstrom, K. N. A Convenient Preparation of Heteroaryl Sulfonamides and Sulfonyl Fluorides from Heteroaryl Thiols. *J. Org. Chem.* **2006**, *71* (3), 1080–1084. https://doi.org/10.1021/jo052164+.

(2) Bornholdt, J.; Fjære, K. W.; Felding, J.; Kristensen, J. L. Heterocyclic Pentafluorophenyl Sulfonate Esters as Shelf Stable Alternatives to Sulfonyl Chlorides. *Tetrahedron* **2009**, *65* (45), 9280–9284. https://doi.org/10.1016/j.tet.2009.09.015.

(3) Toda, N.; Asano, S.; Barbas, C. F. Rapid, Stable, Chemoselective Labeling of Thiols with Julia-Kocieński-like Reagents: A Serum-Stable Alternative to Maleimide-Based Protein Conjugation. *Angew. Chem.* **2013**, *125* (48), 12824–12828. https://doi.org/10.1002/ange.201306241.

(4) Motiwala, H. F.; Kuo, Y.-H.; Stinger, B. L.; Palfey, B. A.; Martin, B. R. Tunable Heteroaromatic Sulfones Enhance In-Cell Cysteine Profiling. *J. Am. Chem. Soc.* **2020**, *142* (4), 1801–1810. https://doi.org/10.1021/jacs.9b08831.

(5) Hansch, Corwin.; Leo, A.; Taft, R. W. A Survey of Hammett Substituent Constants and Resonance and Field Parameters. *Chem. Rev.* **1991**, *91* (2), 165–195. https://doi.org/10.1021/cr00002a004.

(6) Williams, C. J.; Headd, J. J.; Moriarty, N. W.; Prisant, M. G.; Videau, L. L.; Deis, L. N.; Verma, V.; Keedy, D. A.; Hintze, B. J.; Chen, V. B.; Jain, S.; Lewis, S. M.; Arendall, W. B.; Snoeyink, J.; Adams, P. D.; Lovell, S. C.; Richardson, J. S.; Richardson, D. C. MolProbity: More and Better Reference Data for Improved All-Atom Structure Validation: PROTEIN SCIENCE.ORG. *Protein Sci.* **2018**, *27* (1), 293–315. https://doi.org/10.1002/pro.3330.

(7) Baud, M. G. J.; Bauer, M. R.; Verduci, L.; Dingler, F. A.; Patel, K. J.; Horil Roy, D.; Joerger, A. C.; Fersht, A. R. Aminobenzothiazole Derivatives Stabilize the Thermolabile P53 Cancer Mutant Y220C and Show Anticancer Activity in P53-Y220C Cell Lines. *Eur. J. Med. Chem.* **2018**, *152*, 101–114. https://doi.org/10.1016/j.ejmech.2018.04.035.

(8) Peng, J.; Alam, S.; Radhakrishnan, K.; Mariappan, M.; Rudolph, M. G.; May, C.; Dierks, T.; von Figura, K.; Schmidt, B. Eukaryotic Formylglycine-Generating Enzyme Catalyses a Monooxygenase Type of Reaction. *FEBS J.* **2015**, *282* (17), 3262–3274. https://doi.org/10.1111/febs.13347.

(9) The N-Way Toolbox The N-Way Toolbox (Https://Www.Mathworks.Com/Matlabcentral/Fileexchange/1088-the-n-Way-Toolbox), MATLAB Central File Exchange. Retrieved November 7, 2022., 2022.

(10) Frisch, M. J.; Trucks, G. W.; Schlegel, H. B.; Scuseria, G. E.; Robb, M. A.; Cheeseman, J. R.; Scalmani, G.; Barone, V.; Petersson, G. A.; Nakatsuii, H.; Li, X.; Caricato, M.; Marenich, A.; Bloino, J.; Janesko, B. G.; Gomperts, R.; Mennucci, B.; Hratchian, H. P.; Ortiz, J. V.; Izmaylov, A. F.; Sonnenberg, J. L.; Williams-Young, D.; Ding, F.; Lipparini, F.; Egidi, F.; Goings, J.; Peng, B.; Petrone, A.; Henderson, T.; Ranasinghe, D.; Zakrzewski, V. G.; Gao, J.; Rega, N.; Zheng, G.; Liang, W.; Hada, M.; Ehara, M.; Toyota, K.; Fukuda, R.; Hasegawa, J.; Ishida, M.; Nakajima, T.; Honda, Y.; Kitao, O.; Nakai, H.; Vreven, T.; Throssell, K.; Montgomery, J. A.; Peralta, J. J. E.; Ogliaro, F.; Bearpark, M.; Heyd, J. J.; Brothers, E.; Kudin, K. N.; Staroverov, V. N.; Keith, T.; Kobayashi, R.; Normand, J.; Raghavachari, K.; Rendell, A.; Burant, J. C.; Iyengar, S. S.; Tomasi, J.; Cossi, M.; Millam, J. M.; Klene, M.; Adamo, C.; Cammi, R.; Ochterski, J. W.; Martin, R. L.; Morokuma, K.; Farkas, O.; Foresman, J. B.; Fox, D. J. Gaussian 09, (Revisions A.02 and D.01), 2016.

(11) Dennington, R.; Keith, T. A.; Millam, J. M. GaussView, Version 6, 2016.

(12) Chai, J.-D.; Head-Gordon, M. Long-Range Corrected Hybrid Density Functionals with Damped Atom–Atom Dispersion Corrections. *Phys. Chem. Chem. Phys.* **2008**, *10* (44), 6615. https://doi.org/10.1039/b810189b.

(13) Rohrbach, S.; Murphy, J. A.; Tuttle, T. Computational Study on the Boundary Between the Concerted and Stepwise Mechanism of Bimolecular S $_N$ Ar Reactions. *J. Am. Chem. Soc.* **2020**, *142* (35), 14871–14876. https://doi.org/10.1021/jacs.0c01975.

(14) Marenich, A. V.; Cramer, C. J.; Truhlar, D. G. Universal Solvation Model Based on Solute Electron Density and on a Continuum Model of the Solvent Defined by the Bulk Dielectric Constant and Atomic Surface Tensions. *J. Phys. Chem. B* **2009**, *113* (18), 6378–6396. https://doi.org/10.1021/jp810292n.

(15) Joerger, A. C.; Ang, H. C.; Fersht, A. R. Structural Basis for Understanding Oncogenic P53 Mutations and Designing Rescue Drugs. *Proc. Natl. Acad. Sci.* **2006**, *103* (41), 15056–15061. https://doi.org/10.1073/pnas.0607286103.

(16) Kabsch, W. *XDS*. *Acta Crystallogr. D Biol. Crystallogr.* **2010**, *66* (2), 125–132. https://doi.org/10.1107/S0907444909047337.

(17) Winn, M. D.; Ballard, C. C.; Cowtan, K. D.; Dodson, E. J.; Emsley, P.; Evans, P. R.; Keegan, R. M.; Krissinel, E. B.; Leslie, A. G. W.; McCoy, A.; McNicholas, S. J.; Murshudov, G. N.; Pannu, N. S.; Potterton, E. A.; Powell, H. R.; Read, R. J.; Vagin, A.; Wilson, K. S. Overview of the *CCP* 4 Suite and Current Developments. *Acta Crystallogr. D Biol. Crystallogr.* **2011**, *67* (4), 235–242. https://doi.org/10.1107/S0907444910045749.

(18) Adams, P. D.; Afonine, P. V.; Bunkóczi, G.; Chen, V. B.; Davis, I. W.; Echols, N.; Headd, J. J.; Hung, L.-W.; Kapral, G. J.; Grosse-Kunstleve, R. W.; McCoy, A. J.; Moriarty, N. W.; Oeffner, R.; Read, R. J.; Richardson, D. C.; Richardson, J. S.; Terwilliger, T. C.; Zwart, P. H. *PHENIX* : A Comprehensive Python-Based System for Macromolecular Structure Solution. *Acta Crystallogr. D Biol. Crystallogr.* **2010**, *66* (2), 213–221. https://doi.org/10.1107/S0907444909052925.

(19) Bauer, M. R.; Krämer, A.; Settanni, G.; Jones, R. N.; Ni, X.; Khan Tareque, R.; Fersht, A. R.; Spencer, J.; Joerger, A. C. Targeting Cavity-Creating P53 Cancer Mutations with Small-Molecule Stabilizers: The Y220X Paradigm. *ACS Chem. Biol.* **2020**, *15* (3), 657–668. https://doi.org/10.1021/acschembio.9b00748.

(20) Emsley, P.; Lohkamp, B.; Scott, W. G.; Cowtan, K. Features and Development of *Coot*. *Acta Crystallogr. D Biol. Crystallogr.* **2010**, *66* (4), 486–501. https://doi.org/10.1107/S0907444910007493.

(21) Chatzopoulou, M.; Martínez, R. F.; Willis, N. J.; Claridge, T. D. W.; Wilson, F. X.; Wynne, G. M.; Davies, S. G.; Russell, A. J. The Dimroth Rearrangement as a Probable Cause for Structural Misassignments in Imidazo[1,2-a]Pyrimidines: A N-Labelling Study and an Easy Method for the Determination of Regiochemistry. *Tetrahedron* **2018**, *74* (38), 5280–5288. https://doi.org/10.1016/j.tet.2018.06.033.

(22) Janin, Y. L.; Huel, C.; Flad, G.; Thirot, S. Methyl Orthocarboxylates as Methylating Agents of Heterocycles. *Eur. J. Org. Chem.* **2002**, *11*, 1763–1769. https://doi.org/10.1002/1099-0690(200206)2002:11<1763::AID-EJOC1763>3.0.CO;2-Q.

(23) Scribner, A.; Dennis, R.; Hong, J.; Lee, S.; McIntyre, D.; Perrey, D.; Feng, D.; Fisher, M.; Wyvratt, M.; Leavitt, P.; Liberator, P.; Gurnett, A.; Brown, C.; Mathew, J.; Thompson, D.; Schmatz, D.; Biftu, T. Synthesis and Biological Activity of Imidazopyridine Anticoccidial Agents: Part I. *Eur. J. Med. Chem.* **2007**, *42* (11–12), 1334–1357. https://doi.org/10.1016/j.ejmech.2007.02.006.

(24) Stepaniuk, O. O.; Rudenko, T. V.; Vashchenko, B. V.; Matvienko, V. O.; Kondratov, I. S.; Tolmachev, A. A.; Grygorenko, O. O. Reaction of β-Alkoxyvinyl α-Ketoesters with Acyclic NCN Binucleophiles – Scalable Approach to Novel Functionalized Pyrimidines. *Tetrahedron* **2019**, *75* (25), 3472–3478. https://doi.org/10.1016/j.tet.2019.05.005.

(25) Fürstner, A.; Leitner, A.; Méndez, M.; Krause, H. Iron-Catalyzed Cross-Coupling Reactions. *J. Am. Chem. Soc.* **2002**, *124* (46), 13856–13863. https://doi.org/10.1021/ja027190t.

(26) Zhang, Q.; Xia, Z.; Mitten, M. J.; Lasko, L. M.; Klinghofer, V.; Bouska, J.; Johnson, E. F.; Penning, T. D.; Luo, Y.; Giranda, V. L.; Shoemaker, A. R.; Stewart, K. D.; Djuric, S. W.; Vasudevan, A. Hit to Lead Optimization of a Novel Class of Squarate-Containing Polo-like Kinases Inhibitors. *Bioorg. Med. Chem. Lett.* **2012**, *22* (24), 7615–7622. https://doi.org/10.1016/j.bmcl.2012.10.009.

(27) Plé, N.; Turck, A.; Heynderickx, A.; Quéguiner, G. Synthesis and Metalation of Trifluoromethylpyrimidines. Metalation of Diazines. XVI. *J. Heterocycl. Chem.* **1997**, *34* (2), 551–556. https://doi.org/10.1002/jhet.5570340234.
(28) Jacobsen, S. A.; Rødbotten, S.; Benneche, T. Phenylation of Pyrimidinones Using Diphenyliodonium Salts. *J. Chem. Soc. Perkin 1* **1999**, No. 22, 3265–3268. https://doi.org/10.1039/a905519c.
(29) Itami, K.; Yamazaki, D.; Yoshida, J. Pyrimidine-Core Extended π-Systems: General Synthesis and Interesting Fluorescent Properties. *J. Am. Chem. Soc.* **2004**, *126* (47), 15396–15397. https://doi.org/10.1021/ja044923w.
(30) Herstad, G.; Benneche, T. Decarboxylation in the Synthesis of 4-Alkyl-, 4-Alkenyl- and 4-Acylpyrimidines. *J. Heterocycl. Chem.* **2003**, *40* (2), 219–224. https://doi.org/10.1002/jhet.5570400204.
(31) Zhichkin, P.; Fairfax, D. J.; Eisenbeis, S. A. A General Procedure for the Synthesis of 2-Substituted Pyrimidine-5-Carboxylic Esters. *Synthesis* **2002**, *2002* (06), 720–722. https://doi.org/10.1055/s-2002-25767.
(32) Mo, J.; Eom, D.; Kim, S. H.; Lee, P. H. Palladium-Catalyzed Carbon–Sulfur Cross-Coupling Reactions of Aryl Chlorides with Indium Tris(Organothiolates). *Chem. Lett.* **2011**, *40* (9), 980–982. https://doi.org/10.1246/cl.2011.980.
(33) Bcherr, J.; Lundsgaard, J. INTRODUCTION OF SULFUR IN COMPOUNDS WITH REACTIVE HALOGEN ATOMS VIA THE *t* -BUTYLTHIOLATE ANION. *Phosphorus Sulfur Relat. Elem.* **1983**, *14* (2), 131–138. https://doi.org/10.1080/03086648308075933.
(34) Jiang, M.; Li, H.; Yang, H.; Fu, H. Room-Temperature Arylation of Thiols: Breakthrough with Aryl Chlorides. *Angew. Chem. Int. Ed.* **2017**, *56* (3), 874–879. https://doi.org/10.1002/anie.201610414.
(35) Yang, J.; Wang, L.; Lv, Y.; Li, N.; An, Y.; Gao, S. Oxidative Kinetic Resolution of Heterocyclic Sulfoxides with a Porphyrin-Inspired Manganese Complex by Hydrogen Peroxide. *Tetrahedron Lett.* **2018**, *59* (2), 156–159. https://doi.org/10.1016/j.tetlet.2017.12.012.
(36) Solberg, J.; Undheim, K.; Pettersson, L.; Öhman, L.-O.; Ruiz, J.; Colacio, E.; Mulichak, A. M.; Alminger, T.; Erickson, M.; Grundevik, I.; Hagin, I.; Hoffman, K.-J.; Johansson, S.; Larsson, S.; Löfberg, I.; Ohlson, K.; Persson, B.; Skånberg, I.; Tekenbergs-Hjelte, L. Regiochemistry in Pd-Catalysed Organotin Reactions with Halopyrimidines. *Acta Chem. Scand.* **1989**, *43*, 62–68. https://doi.org/10.3891/acta.chem.scand.43-0062.
(37) Kamijo, S.; Kamijo, K.; Murafuji, T. Synthesis of Alkylated Pyrimidines via Photoinduced Coupling Using Benzophenone as a Mediator. *J. Org. Chem.* **2017**, *82* (5), 2664–2671. https://doi.org/10.1021/acs.joc.6b03058.
(38) Liang, Y.; Luo, S.; Zhang, Z.; Ma, Y. EFFICIENT SYNTHESIS OF A NEW PYRIMIDINE DERIVATIVE. *Synth. Commun.* **2002**, *32* (1), 153–157. https://doi.org/10.1081/SCC-120001523.
(39) Guilbaud, J.; Labonde, M.; Selmi, A.; Kammoun, M.; Cattey, H.; Pirio, N.; Roger, J.; Hierso, J.-C. Palladium-Catalyzed Heteroaryl Thioethers Synthesis Overcoming Palladium Dithiolate Resting States Inertness: Practical Road to Sulfones and NH-Sulfoximines. *Catal. Commun.* **2018**, *111*, 52–58. https://doi.org/10.1016/j.catcom.2018.03.025.
(40) Pospíšil, J.; Sato, H. Practical Synthesis of β-Acyl and β-Alkoxycarbonyl Heterocyclic Sulfones. *J. Org. Chem.* **2011**, *76* (7), 2269–2272. https://doi.org/10.1021/jo102326p.
(41) Tang, K.; Cao, J.; Boatner, L. M.; Li, L.; Farhi, J.; Houk, K. N.; Spangle, J.; Backus, K. M.; Raj, M. Tunable Amine-Reactive Electrophiles for Selective Profiling of Lysine. *Angew. Chem. Int. Ed.* **2022**, *61* (5). https://doi.org/10.1002/anie.202112107.
(42) Zambaldo, C.; Vinogradova, E. V.; Qi, X.; Iaconelli, J.; Suciu, R. M.; Koh, M.; Senkane, K.; Chadwick, S. R.; Sanchez, B. B.; Chen, J. S.; Chatterjee, A. K.; Liu, P.; Schultz, P. G.; Cravatt, B. F.; Bollong, M. J. 2-Sulfonylpyridines as Tunable, Cysteine-Reactive Electrophiles. *J. Am. Chem. Soc.* **2020**, *142* (19), 8972–8979. https://doi.org/10.1021/jacs.0c02721.

(43) Thierry, T.; Pfund, E.; Lequeux, T. Metal-Free Aminomethylation of Aromatic Sulfones Promoted by Eosin Y. *Chem. – Eur. J.* **2021**, *27* (60), 14826–14830. https://doi.org/10.1002/chem.202102124.
(44) Tosato, M. L.; Soccorsi, L. Regioselective Reactions in Heteroaromatic Systems. Rules for Methyl Migration and Nucleophilic Substitution in Methyl Cyanurates and Thiocyanurates. *J. Chem. Soc. Perkin Trans. 2* **1982**, No. 11, 1321. https://doi.org/10.1039/p29820001321.
(45) Melzig, L.; Metzger, A.; Knochel, P. Room Temperature Cross-Coupling of Highly Functionalized Organozinc Reagents with Thiomethylated *N* -Heterocycles by Nickel Catalysis. *J. Org. Chem.* **2010**, *75* (6), 2131–2133. https://doi.org/10.1021/jo1001615.
