# Supplementary material for: Structure–Reactivity Studies of 2-Sulfonylpyrimidines Allow Selective Protein Arylation
Source: Bioconjug Chem. 2023 Sep 1;34(9):1679–87. doi: 10.1021/acs.bioconjchem.3c00322 (PMC10515483; doi:10.1021/acs.bioconjchem.3c00322)
Supplement: Supplementary file 2 — bc3c00322_si_002.pdf [file bc3c00322_si_002.pdf]

Full wwPDB X-ray Structure Validation Report (i)

Feb 7, 2023 – 01:07 am GMT

PDB ID	:	8CG7
Title	:	Structure of p53 cancer mutant Y220C with arylation at Cys182 and Cys277
Deposited on	:	2023-02-03
Resolution	:	1.53 Å(reported)

This wwPDB validation report is for manuscript review

This is a Full wwPDB X-ray Structure Validation Report.

This report is produced by the wwPDB biocuration pipeline after annotation of the structure.

We welcome your comments at *validation@mail.wwpdb.org*
A user guide is available at
https://www.wwpdb.org/validation/2017/XrayValidationReportHelp
with specific help available everywhere you see the (i) symbol.

The types of validation reports are described at
http://www.wwpdb.org/validation/2017/FAQs#types.

The following versions of software and data (see references (i)) were used in the production of this report:

MolProbity	:	4.02b-467
Mogul	:	1.8.4, CSD as541be (2020)
Xtriage (Phenix)	:	1.13
EDS	:	2.32.1
Percentile statistics	:	20191225.v01 (using entries in the PDB archive December 25th 2019)
Refmac	:	5.8.0158
CCP4	:	7.0.044 (Gargrove)
Ideal geometry (proteins)	:	Engh & Huber (2001)
Ideal geometry (DNA, RNA)	:	Parkinson et al. (1996)
Validation Pipeline (wwPDB-VP)	:	2.32.1

For Manuscript Review

1 Overall quality at a glance (i)

The following experimental techniques were used to determine the structure:
X-RAY DIFFRACTION

The reported resolution of this entry is 1.53 Å.

Percentile scores (ranging between 0-100) for global validation metrics of the entry are shown in the following graphic. The table shows the number of entries on which the scores are based.

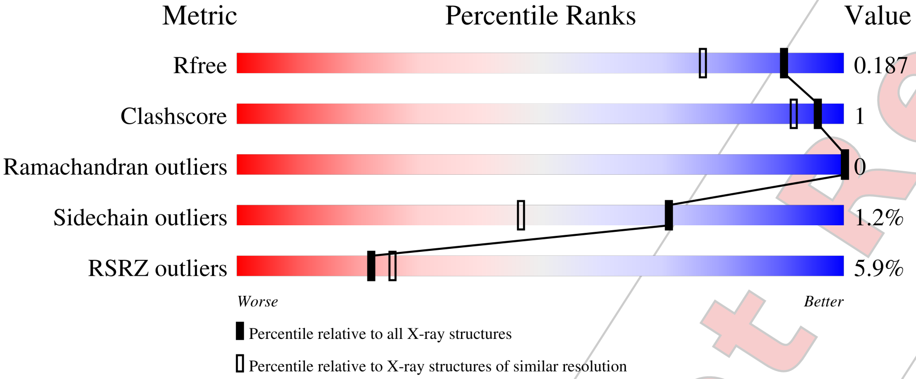

Metric	Whole archive (#Entries)	Similar resolution (#Entries, resolution range(Å))
R_{free}	130704	2556 (1.56-1.52)
Clashscore	141614	2634 (1.56-1.52)
Ramachandran outliers	138981	2580 (1.56-1.52)
Sidechain outliers	138945	2577 (1.56-1.52)
RSRZ outliers	127900	2524 (1.56-1.52)

The table below summarises the geometric issues observed across the polymeric chains and their fit to the electron density. The red, orange, yellow and green segments of the lower bar indicate the fraction of residues that contain outliers for >=3, 2, 1 and 0 types of geometric quality criteria respectively. A grey segment represents the fraction of residues that are not modelled. The numeric value for each fraction is indicated below the corresponding segment, with a dot representing fractions <=5% The upper red bar (where present) indicates the fraction of residues that have poor fit to the electron density. The numeric value is given above the bar.

Mol	Chain	Length	Quality of chain
1	A	219	6% / 88% • 10%
1	B	219	4% / 85% 5% 10%

For Manuscript Review

2 Entry composition (i)

There are 4 unique types of molecules in this entry. The entry contains 3602 atoms, of which 0 are hydrogens and 0 are deuteriums.

In the tables below, the ZeroOcc column contains the number of atoms modelled with zero occupancy, the AltConf column contains the number of residues with at least one atom in alternate conformation and the Trace column contains the number of residues modelled with at most 2 atoms.

- Molecule 1 is a protein called Cellular tumor antigen p53.

Mol	Chain	Residues	Atoms						ZeroOcc	AltConf	Trace
1	A	197	Total 1572	C 970	I 2	N 285	O 298	S 17	0	7	0
1	B	198	Total 1578	C 977	I 2	N 287	O 296	S 16	0	10	0

There are 10 discrepancies between the modelled and reference sequences:

Chain	Residue	Modelled	Actual	Comment	Reference
A	133	LEU	MET	variant	UNP P04637
A	203	ALA	VAL	variant	UNP P04637
A	220	CYS	TYR	variant	UNP P04637
A	239	TYR	ASN	variant	UNP P04637
A	268	ASP	ASN	conflict	UNP P04637
B	133	LEU	MET	variant	UNP P04637
B	203	ALA	VAL	variant	UNP P04637
B	220	CYS	TYR	variant	UNP P04637
B	239	TYR	ASN	variant	UNP P04637
B	268	ASP	ASN	conflict	UNP P04637

- Molecule 2 is ZINC ION (three-letter code: ZN) (formula: Zn).

Mol	Chain	Residues	Atoms		ZeroOcc	AltConf
2	A	1	Total 1	Zn 1	0	0
2	B	1	Total 1	Zn 1	0	0

- Molecule 3 is 1,2-ETHANEDIOL (three-letter code: EDO) (formula: $C_2H_6O_2$).

For Manuscript Review

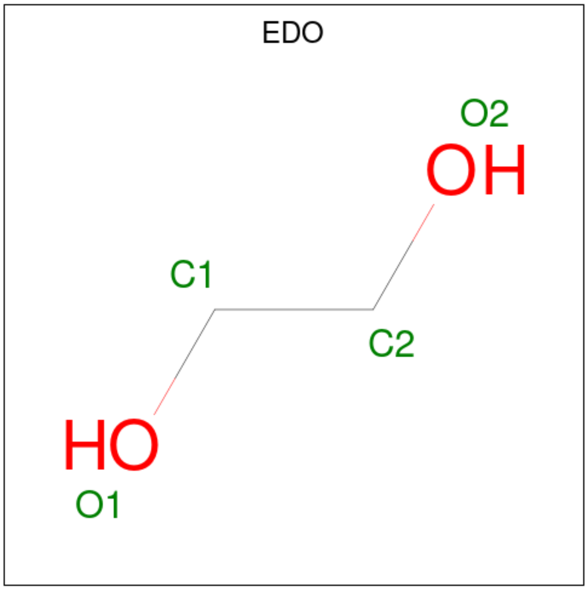

Mol	Chain	Residues	Atoms	ZeroOcc	AltConf
3	B	1	Total C O<br>4 2 2	0	0

- Molecule 4 is water.

Mol	Chain	Residues	Atoms	ZeroOcc	AltConf
4	A	225	Total O<br>225 225	0	0
4	B	221	Total O<br>221 221	0	0

For Manuscript Review

3 Residue-property plots (i)

These plots are drawn for all protein, RNA, DNA and oligosaccharide chains in the entry. The first graphic for a chain summarises the proportions of the various outlier classes displayed in the second graphic. The second graphic shows the sequence view annotated by issues in geometry and electron density. Residues are color-coded according to the number of geometric quality criteria for which they contain at least one outlier: green = 0, yellow = 1, orange = 2 and red = 3 or more. A red dot above a residue indicates a poor fit to the electron density (RSRZ > 2). Stretches of 2 or more consecutive residues without any outlier are shown as a green connector. Residues present in the sample, but not in the model, are shown in grey.

• Molecule 1: Cellular tumor antigen p53

Chain A:

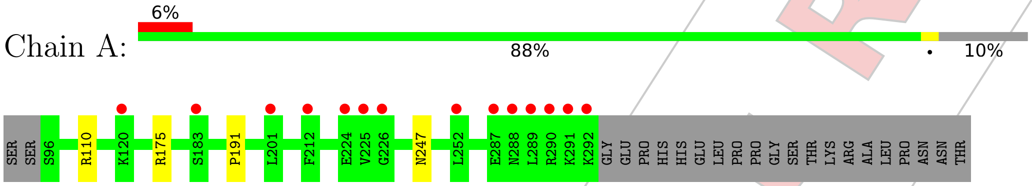

• Molecule 1: Cellular tumor antigen p53

Chain B:

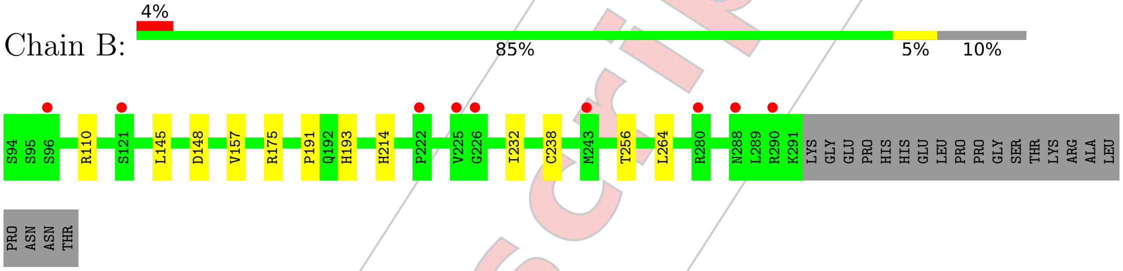

For Manuscript Review

4 Data and refinement statistics ⓘ

Property	Value	Source
Space group	P 21 21 21	Depositor
Cell constants<br>a, b, c, α, β, γ	64.88Å 71.05Å 104.89Å<br>90.00° 90.00° 90.00°	Depositor
Resolution (Å)	47.90 – 1.53<br>47.91 – 1.53	Depositor<br>EDS
% Data completeness<br>(in resolution range)	99.0 (47.90-1.53)<br>99.2 (47.91-1.53)	Depositor<br>EDS
R_{merge}	0.05	Depositor
R_{sym}	(Not available)	Depositor
$< I/\sigma(I) >$ [1]	2.59 (at 1.53Å)	Xtriage
Refinement program	PHENIX 1.10.1_2155	Depositor
R, R_{free}	0.158 , 0.187<br>0.158 , 0.187	Depositor<br>DCC
R_{free} test set	3698 reflections (5.05%)	wwPDB-VP
Wilson B-factor (Å^2)	19.3	Xtriage
Anisotropy	0.584	Xtriage
Bulk solvent k_{sol}(e/Å^3), B_{sol}(Å^2)	0.36 , 47.5	EDS
L-test for twinning[2]	$< \|L\| > = 0.53$, $< L^2 > = 0.37$	Xtriage
Estimated twinning fraction	No twinning to report.	Xtriage
F_o,F_c correlation	0.97	EDS
Total number of atoms	3602	wwPDB-VP
Average B, all atoms (Å^2)	26.0	wwPDB-VP

Xtriage's analysis on translational NCS is as follows: *The analyses of the Patterson function reveals a significant off-origin peak that is 46.57 % of the origin peak, indicating pseudo-translational symmetry. The chance of finding a peak of this or larger height randomly in a structure without pseudo-translational symmetry is equal to 1.1229e-04. The detected translational NCS is most likely also responsible for the elevated intensity ratio.*

[1] Intensities estimated from amplitudes.

[2] Theoretical values of $< |L| >$, $< L^2 >$ for acentric reflections are 0.5, 0.333 respectively for untwinned datasets, and 0.375, 0.2 for perfectly twinned datasets.

For Manuscript Review

5 Model quality (i)

5.1 Standard geometry (i)

Bond lengths and bond angles in the following residue types are not validated in this section: UJR, EDO, ZN

The Z score for a bond length (or angle) is the number of standard deviations the observed value is removed from the expected value. A bond length (or angle) with $|Z| > 5$ is considered an outlier worth inspection. RMSZ is the root-mean-square of all Z scores of the bond lengths (or angles).

Mol	Chain	Bond lengths		Bond angles	
		RMSZ	$\#\|Z\| > 5$	RMSZ	$\#\|Z\| > 5$
1	A	0.37	0/1594	0.55	0/2162
1	B	0.37	0/1609	0.55	0/2184
All	All	0.37	0/3203	0.55	0/4346

There are no bond length outliers.

There are no bond angle outliers.

There are no chirality outliers.

There are no planarity outliers.

5.2 Too-close contacts (i)

In the following table, the Non-H and H(model) columns list the number of non-hydrogen atoms and hydrogen atoms in the chain respectively. The H(added) column lists the number of hydrogen atoms added and optimized by MolProbity. The Clashes column lists the number of clashes within the asymmetric unit, whereas Symm-Clashes lists symmetry-related clashes.

Mol	Chain	Non-H	H(model)	H(added)	Clashes	Symm-Clashes
1	A	1572	0	1478	1	0
1	B	1578	0	1492	6	0
2	A	1	0	0	0	0
2	B	1	0	0	0	0
3	B	4	0	6	1	0
4	A	225	0	0	0	0
4	B	221	0	0	0	0
All	All	3602	0	2976	7	0

The all-atom clashscore is defined as the number of clashes found per 1000 atoms (including hydrogen atoms). The all-atom clashscore for this structure is 1.

For Manuscript Review

All (7) close contacts within the same asymmetric unit are listed below, sorted by their clash magnitude.

Atom-1	Atom-2	Interatomic distance (Å)	Clash overlap (Å)
1:B:148:ASP:HA	3:B:401:EDO:H22	1.78	0.64
1:B:157[B]:VAL:HG11	1:B:232:ILE:HD11	1.94	0.50
1:B:193:HIS:CE1	1:B:214:HIS:HB3	2.49	0.47
1:A:175:ARG:HD3	1:A:191:PRO:O	2.14	0.47
1:B:175:ARG:HD3	1:B:191:PRO:O	2.15	0.46
1:B:256[B]:THR:HG23	1:B:264:LEU:HD12	1.98	0.43
1:B:145:LEU:HD13	1:B:157[A]:VAL:HG21	2.01	0.43

There are no symmetry-related clashes.

5.3 Torsion angles (i)

5.3.1 Protein backbone (i)

In the following table, the Percentiles column shows the percent Ramachandran outliers of the chain as a percentile score with respect to all X-ray entries followed by that with respect to entries of similar resolution.

The Analysed column shows the number of residues for which the backbone conformation was analysed, and the total number of residues.

Mol	Chain	Analysed	Favoured	Allowed	Outliers	Percentiles	
1	A	200/219 (91%)	197 (98%)	3 (2%)	0	100	100
1	B	204/219 (93%)	204 (100%)	0	0	100	100
All	All	404/438 (92%)	401 (99%)	3 (1%)	0	100	100

There are no Ramachandran outliers to report.

5.3.2 Protein sidechains (i)

In the following table, the Percentiles column shows the percent sidechain outliers of the chain as a percentile score with respect to all X-ray entries followed by that with respect to entries of similar resolution.

The Analysed column shows the number of residues for which the sidechain conformation was analysed, and the total number of residues.

For Manuscript Review

Mol	Chain	Analysed	Rotameric	Outliers	Percentiles	
1	A	175/193 (91%)	173 (99%)	2 (1%)	73	51
1	B	175/193 (91%)	173 (99%)	2 (1%)	73	51
All	All	350/386 (91%)	346 (99%)	4 (1%)	71	51

All (4) residues with a non-rotameric sidechain are listed below:

Mol	Chain	Res	Type
1	A	110	ARG
1	A	247	ASN
1	B	110	ARG
1	B	238	CYS

Sometimes sidechains can be flipped to improve hydrogen bonding and reduce clashes. There are no such sidechains identified.

5.3.3 RNA ⓘ

There are no RNA molecules in this entry.

5.4 Non-standard residues in protein, DNA, RNA chains ⓘ

4 non-standard protein/DNA/RNA residues are modelled in this entry.

In the following table, the Counts columns list the number of bonds (or angles) for which Mogul statistics could be retrieved, the number of bonds (or angles) that are observed in the model and the number of bonds (or angles) that are defined in the Chemical Component Dictionary. The Link column lists molecule types, if any, to which the group is linked. The Z score for a bond length (or angle) is the number of standard deviations the observed value is removed from the expected value. A bond length (or angle) with $|Z| > 2$ is considered an outlier worth inspection. RMSZ is the root-mean-square of all Z scores of the bond lengths (or angles).

Mol	Type	Chain	Res	Link	Bond lengths			Bond angles		
					Counts	RMSZ	$\#\|Z\|>2$	Counts	RMSZ	$\#\|Z\|>2$
1	UJR	B	277	1	12,13,13	0.20	0	7,16,16	0.69	0
1	UJR	B	182	1	12,13,13	0.28	0	7,16,16	0.59	0
1	UJR	A	277	1	12,13,13	0.20	0	7,16,16	0.66	0
1	UJR	A	182	1	12,13,13	0.18	0	7,16,16	0.59	0

In the following table, the Chirals column lists the number of chiral outliers, the number of chiral centers analysed, the number of these observed in the model and the number defined in the Chemical Component Dictionary. Similar counts are reported in the Torsion and Rings columns. '-' means no outliers of that kind were identified.

For Manuscript Review

Mol	Type	Chain	Res	Link	Chirals	Torsions	Rings
1	UJR	B	277	1	-	0/5/7/7	0/1/1/1
1	UJR	B	182	1	-	1/5/7/7	0/1/1/1
1	UJR	A	277	1	-	0/5/7/7	0/1/1/1
1	UJR	A	182	1	-	0/5/7/7	0/1/1/1

There are no bond length outliers.

There are no bond angle outliers.

There are no chirality outliers.

All (1) torsion outliers are listed below:

Mol	Chain	Res	Type	Atoms
1	B	182	UJR	N-CA-CB-S

There are no ring outliers.

No monomer is involved in short contacts.

5.5 Carbohydrates (i)

There are no monosaccharides in this entry.

5.6 Ligand geometry (i)

Of 3 ligands modelled in this entry, 2 are monoatomic - leaving 1 for Mogul analysis.

In the following table, the Counts columns list the number of bonds (or angles) for which Mogul statistics could be retrieved, the number of bonds (or angles) that are observed in the model and the number of bonds (or angles) that are defined in the Chemical Component Dictionary. The Link column lists molecule types, if any, to which the group is linked. The Z score for a bond length (or angle) is the number of standard deviations the observed value is removed from the expected value. A bond length (or angle) with $|Z| > 2$ is considered an outlier worth inspection. RMSZ is the root-mean-square of all Z scores of the bond lengths (or angles).

Mol	Type	Chain	Res	Link	Bond lengths			Bond angles		
					Counts	RMSZ	$\#\|Z\| > 2$	Counts	RMSZ	$\#\|Z\| > 2$
3	EDO	B	401	-	3,3,3	0.44	0	2,2,2	0.43	0

In the following table, the Chirals column lists the number of chiral outliers, the number of chiral centers analysed, the number of these observed in the model and the number defined in the Chemical Component Dictionary. Similar counts are reported in the Torsion and Rings columns. '-' means no outliers of that kind were identified.

For Manuscript Review

Mol	Type	Chain	Res	Link	Chirals	Torsions	Rings
3	EDO	B	401	-	-	0/1/1/1	-

There are no bond length outliers.

There are no bond angle outliers.

There are no chirality outliers.

There are no torsion outliers.

There are no ring outliers.

1 monomer is involved in 1 short contact:

Mol	Chain	Res	Type	Clashes	Symm-Clashes
3	B	401	EDO	1	0

5.7 Other polymers (i)

There are no such residues in this entry.

5.8 Polymer linkage issues (i)

There are no chain breaks in this entry.

For Manuscript Review

6 Fit of model and data (i)

6.1 Protein, DNA and RNA chains (i)

In the following table, the column labelled '#RSRZ> 2' contains the number (and percentage) of RSRZ outliers, followed by percent RSRZ outliers for the chain as percentile scores relative to all X-ray entries and entries of similar resolution. The OWAB column contains the minimum, median, 95th percentile and maximum values of the occupancy-weighted average B-factor per residue. The column labelled 'Q< 0.9' lists the number of (and percentage) of residues with an average occupancy less than 0.9.

Mol	Chain	Analysed	<RSRZ>	#RSRZ>2			OWAB($Å^2$)	Q<0.9
1	A	195/219 (89%)	0.12	14 (7%)	15	17	16, 23, 43, 57	0
1	B	196/219 (89%)	0.12	9 (4%)	32	36	15, 23, 42, 62	0
All	All	391/438 (89%)	0.12	23 (5%)	22	25	15, 23, 43, 62	0

All (23) RSRZ outliers are listed below:

Mol	Chain	Res	Type	RSRZ
1	A	183	SER	4.4
1	A	288	ASN	3.7
1	A	287	GLU	3.5
1	B	225	VAL	3.4
1	B	226	GLY	3.2
1	A	292	LYS	3.2
1	A	201	LEU	3.0
1	A	226	GLY	2.9
1	B	96[A]	SER	2.9
1	B	290	ARG	2.8
1	B	243	MET	2.6
1	A	289	LEU	2.5
1	A	212	PHE	2.5
1	B	288	ASN	2.4
1	A	291	LYS	2.4
1	A	120	LYS	2.3
1	A	224	GLU	2.3
1	B	121	SER	2.3
1	B	222	PRO	2.2
1	B	280	ARG	2.1
1	A	225	VAL	2.1
1	A	252	LEU	2.1
1	A	290	ARG	2.0

For Manuscript Review

6.2 Non-standard residues in protein, DNA, RNA chains (i)

In the following table, the Atoms column lists the number of modelled atoms in the group and the number defined in the chemical component dictionary. The B-factors column lists the minimum, median, 95th percentile and maximum values of B factors of atoms in the group. The column labelled 'Q< 0.9' lists the number of atoms with occupancy less than 0.9.

Mol	Type	Chain	Res	Atoms	RSCC	RSR	B-factors(Å^2)	Q<0.9
1	UJR	A	277	13/13	0.79	0.17	25,36,75,91	0
1	UJR	B	277	13/13	0.83	0.13	29,42,76,91	0
1	UJR	A	182	13/13	0.94	0.10	27,43,74,85	0
1	UJR	B	182	13/13	0.96	0.12	25,33,46,54	0

6.3 Carbohydrates (i)

There are no monosaccharides in this entry.

6.4 Ligands (i)

In the following table, the Atoms column lists the number of modelled atoms in the group and the number defined in the chemical component dictionary. The B-factors column lists the minimum, median, 95th percentile and maximum values of B factors of atoms in the group. The column labelled 'Q< 0.9' lists the number of atoms with occupancy less than 0.9.

Mol	Type	Chain	Res	Atoms	RSCC	RSR	B-factors(Å^2)	Q<0.9
3	EDO	B	401	4/4	0.81	0.24	45,46,47,48	0
2	ZN	B	402	1/1	1.00	0.07	21,21,21,21	0
2	ZN	A	401	1/1	1.00	0.04	22,22,22,22	0

6.5 Other polymers (i)

There are no such residues in this entry.

For Manuscript Review
