# Supplementary material for: Structure–Reactivity Studies of 2-Sulfonylpyrimidines Allow Selective Protein Arylation
Source: Bioconjug Chem. 2023 Sep 1;34(9):1679–87. doi: 10.1021/acs.bioconjchem.3c00322 (PMC10515483; doi:10.1021/acs.bioconjchem.3c00322)
Supplement: Supplementary file 3 — bc3c00322_si_003.pdf [file bc3c00322_si_003.pdf]

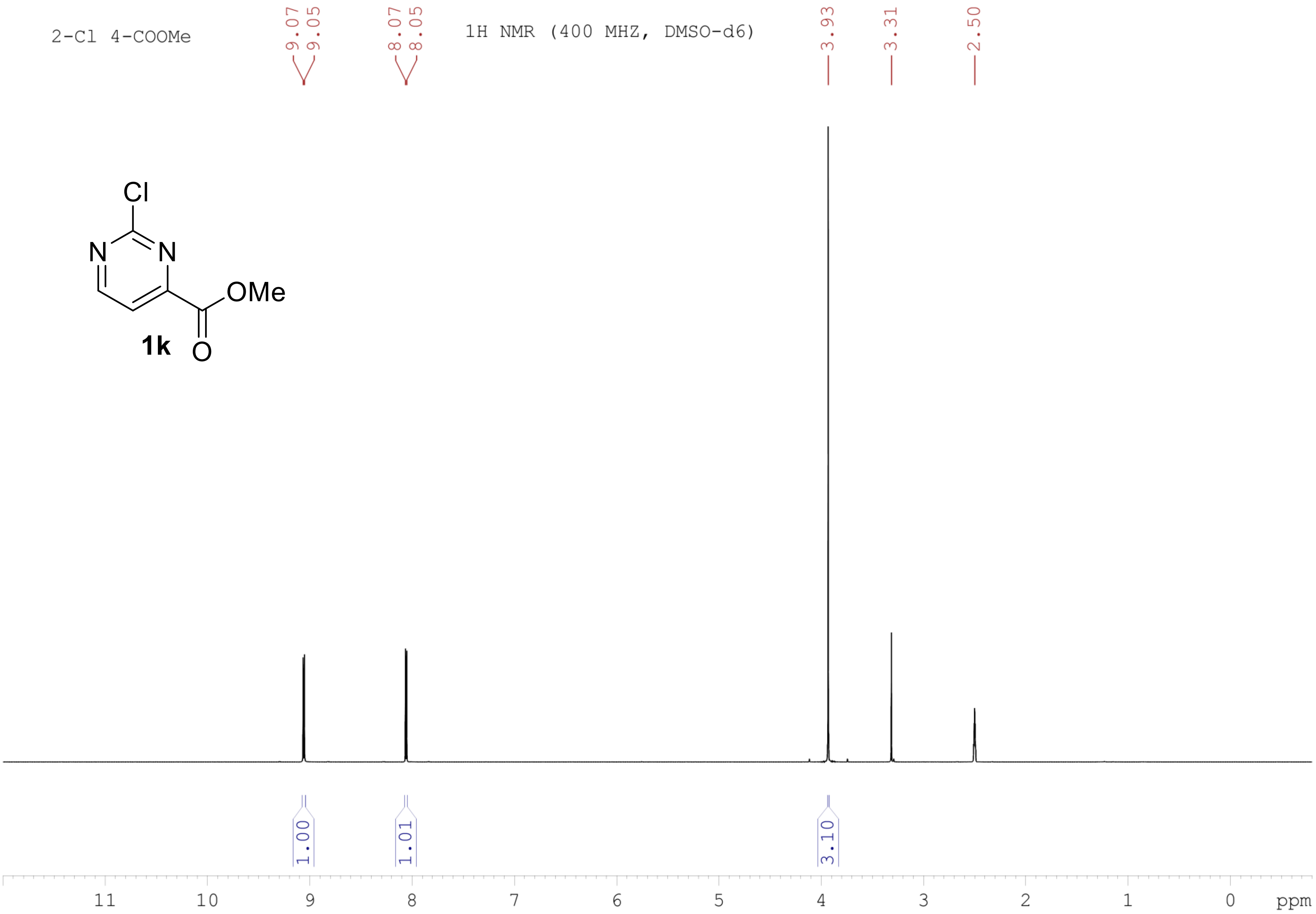
2-Cl 4-COOMe
9.07
9.05
8.07
8.05
1H NMR (400 MHZ, DMSO-d6)
3.93
3.31
2.50
Cl
N
N
OMe
O
1k
1.00
1.01
3.10
11
10
9
8
7
6
5
4
3
2
1
0
ppm

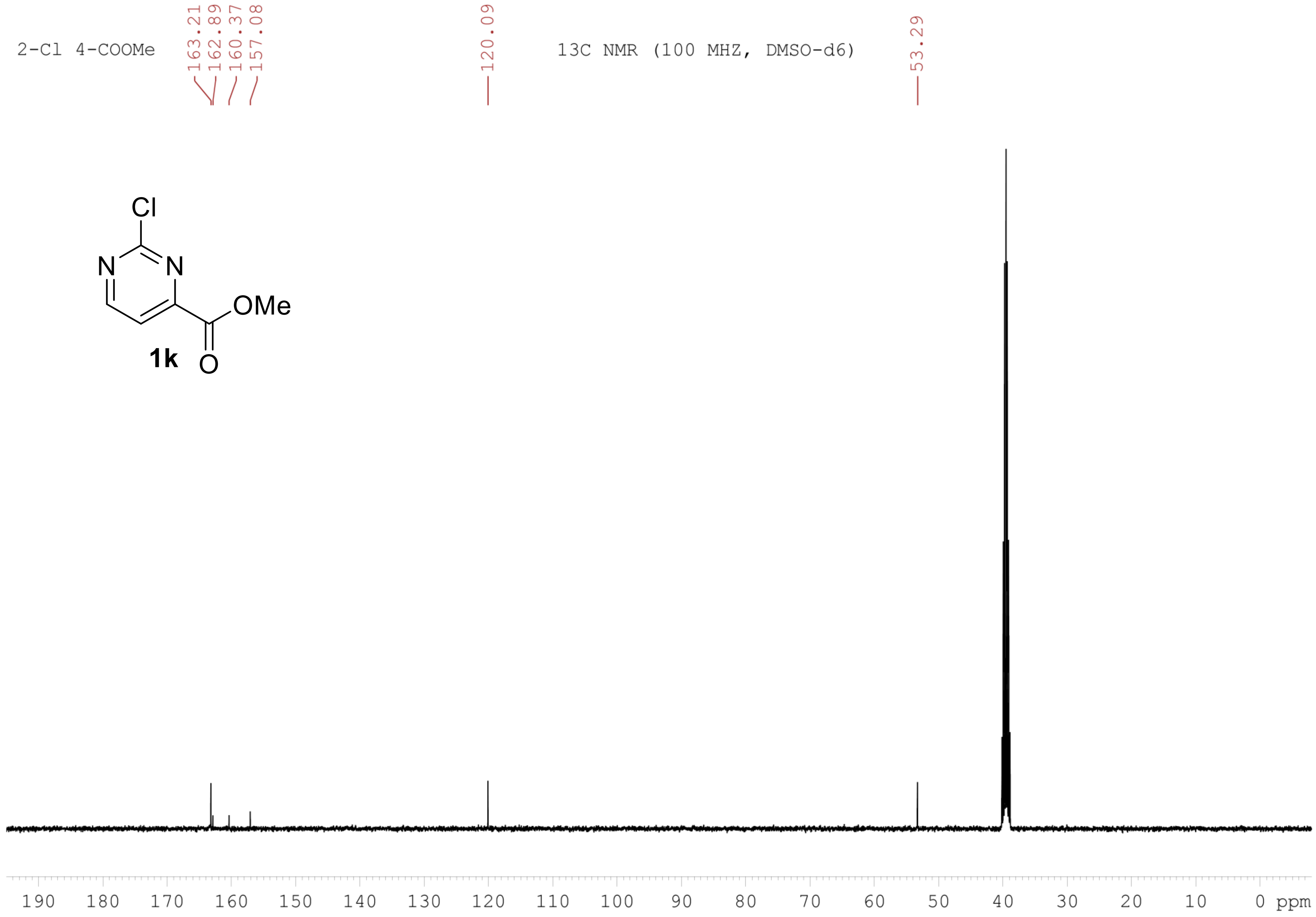
2-Cl 4-COOMe
163.21
162.89
160.37
157.08
120.09
13C NMR (100 MHZ, DMSO-d6)
53.29
Cl
N
N
OMe
O
1k
190 180 170 160 150 140 130 120 110 100 90 80 70 60 50 40 30 20 10 0 ppm

2-Cl 5-COOMe

1H NMR (400 MHZ, DMSO-d6)

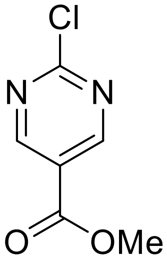

1y

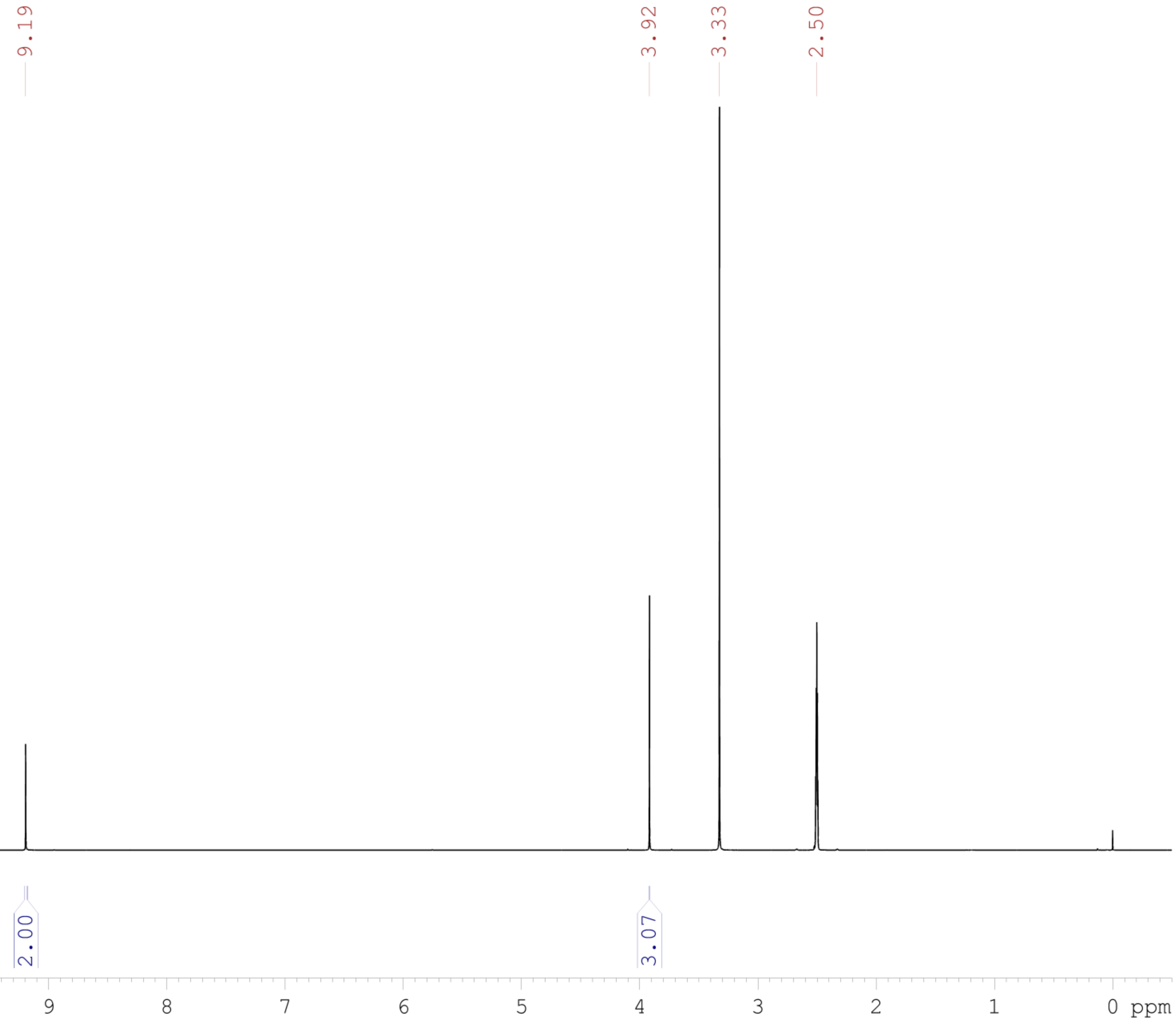

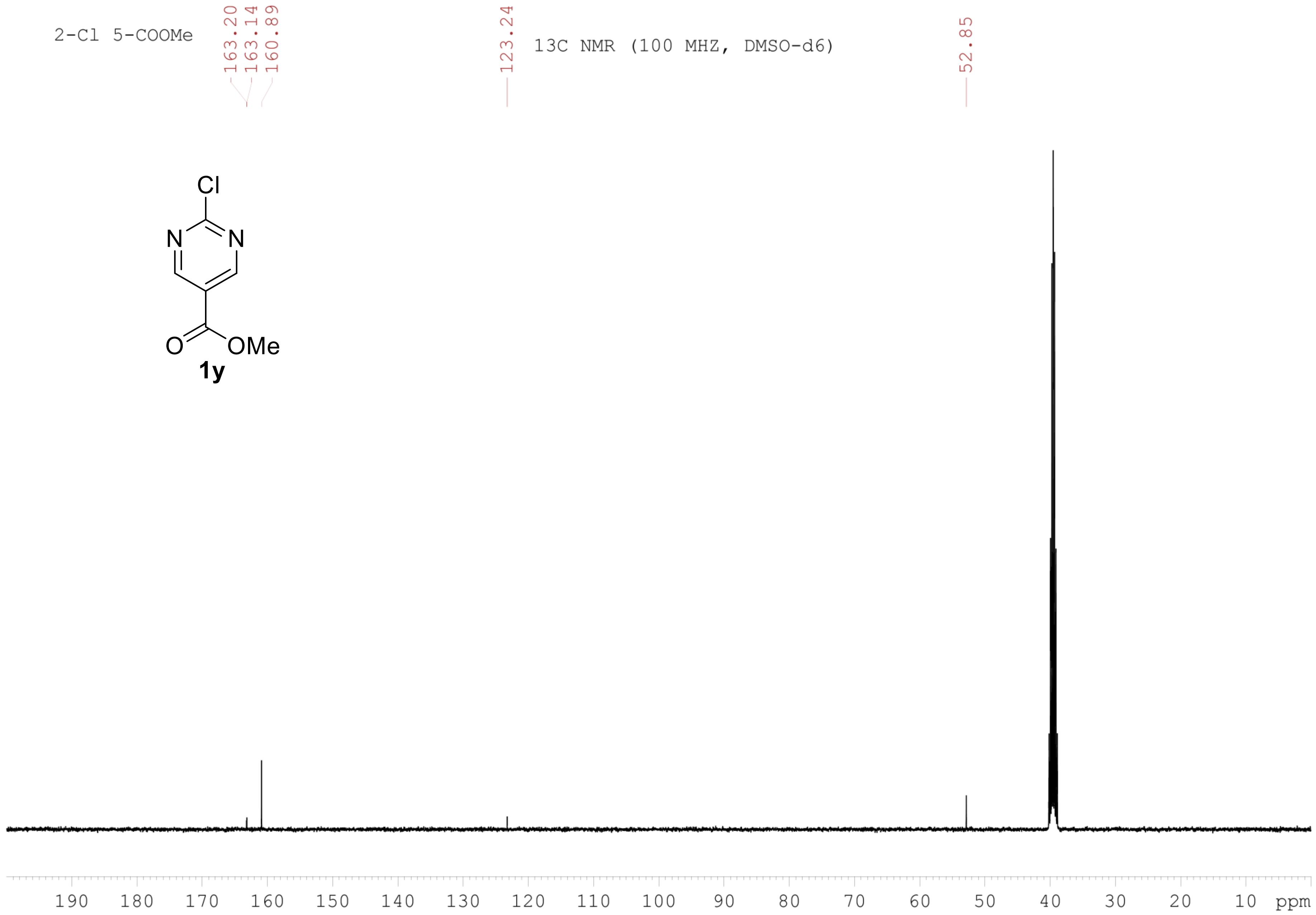
2-Cl 5-COOMe
163.20
163.14
160.89
123.24
13C NMR (100 MHZ, DMSO-d6)
52.85
Cl
N
N
O
OMe
1y
190 180 170 160 150 140 130 120 110 100 90 80 70 60 50 40 30 20 10 ppm

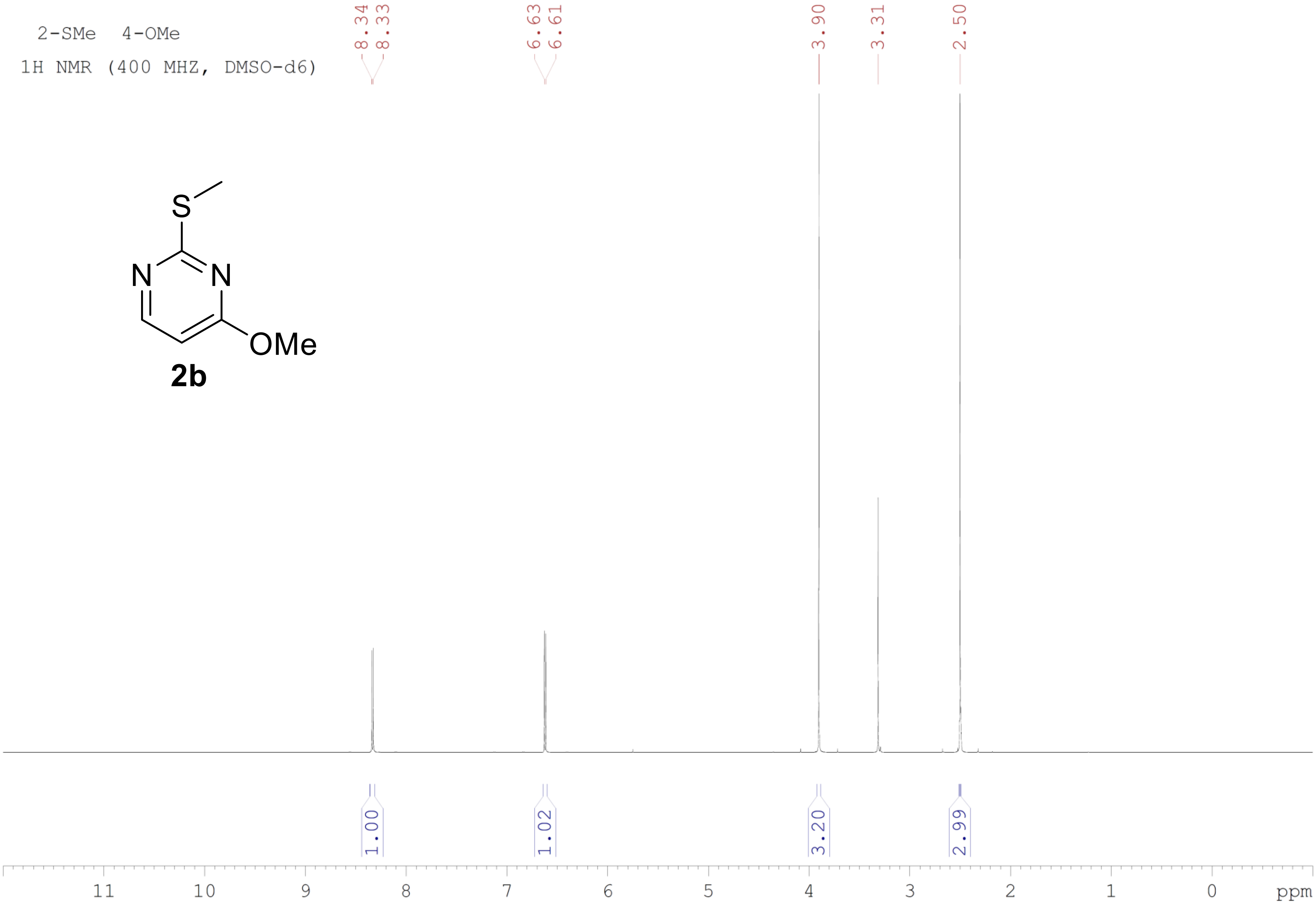

2-SMe 4-OMe
1H NMR (400 MHZ, DMSO-d6)
8.34
8.33
6.63
6.61
3.90
3.31
2.50
OMe
2b
1.00
1.02
3.20
2.99
11
10
9
8
7
6
5
4
3
2
1
0
ppm

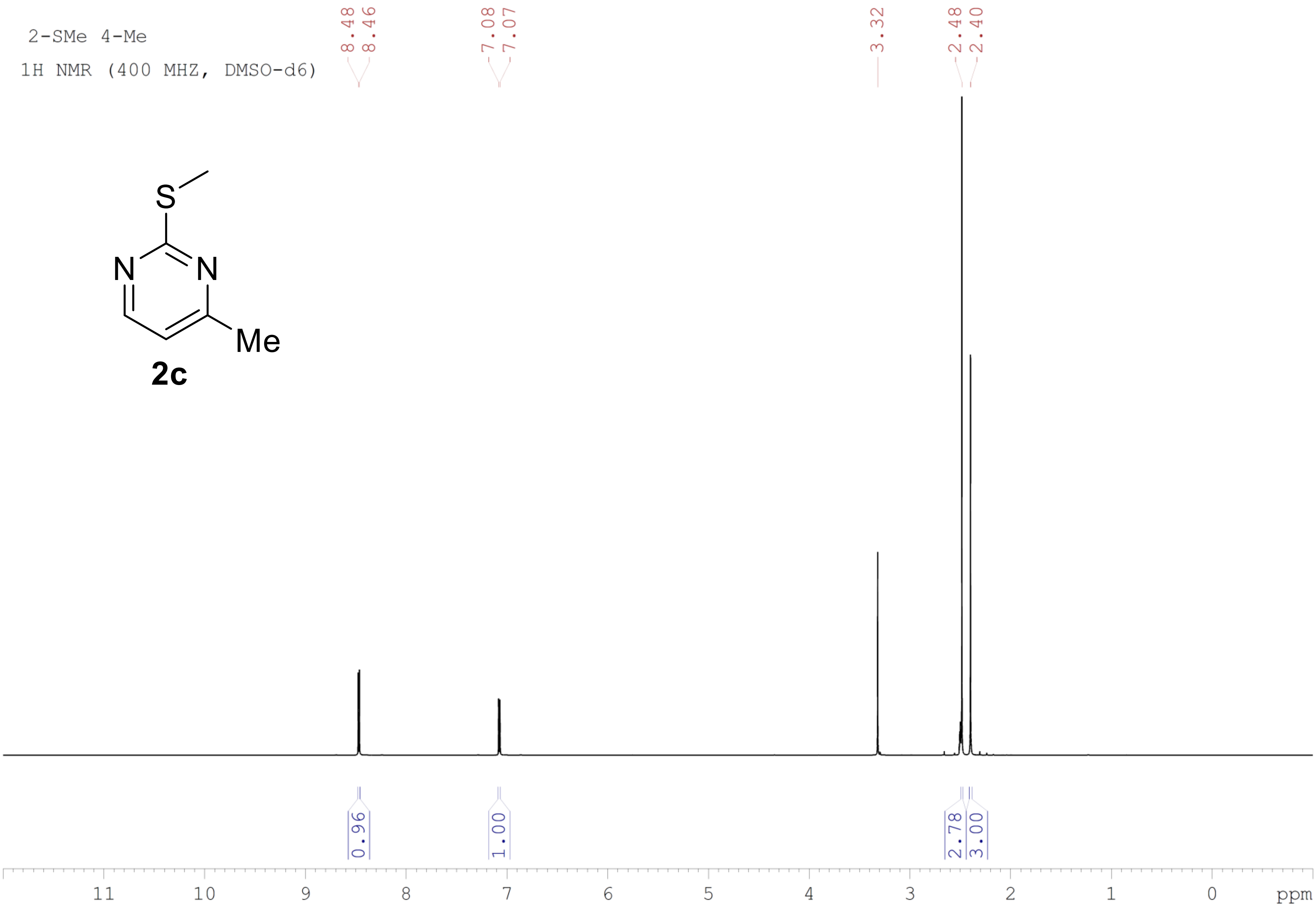
2-SMe 4-Me
1H NMR (400 MHZ, DMSO-d6)
8.48
8.46
7.08
7.07
3.32
2.48
2.40
S
N
N
Me
2c
0.96
1.00
2.78
3.00
11
10
9
8
7
6
5
4
3
2
1
0
ppm

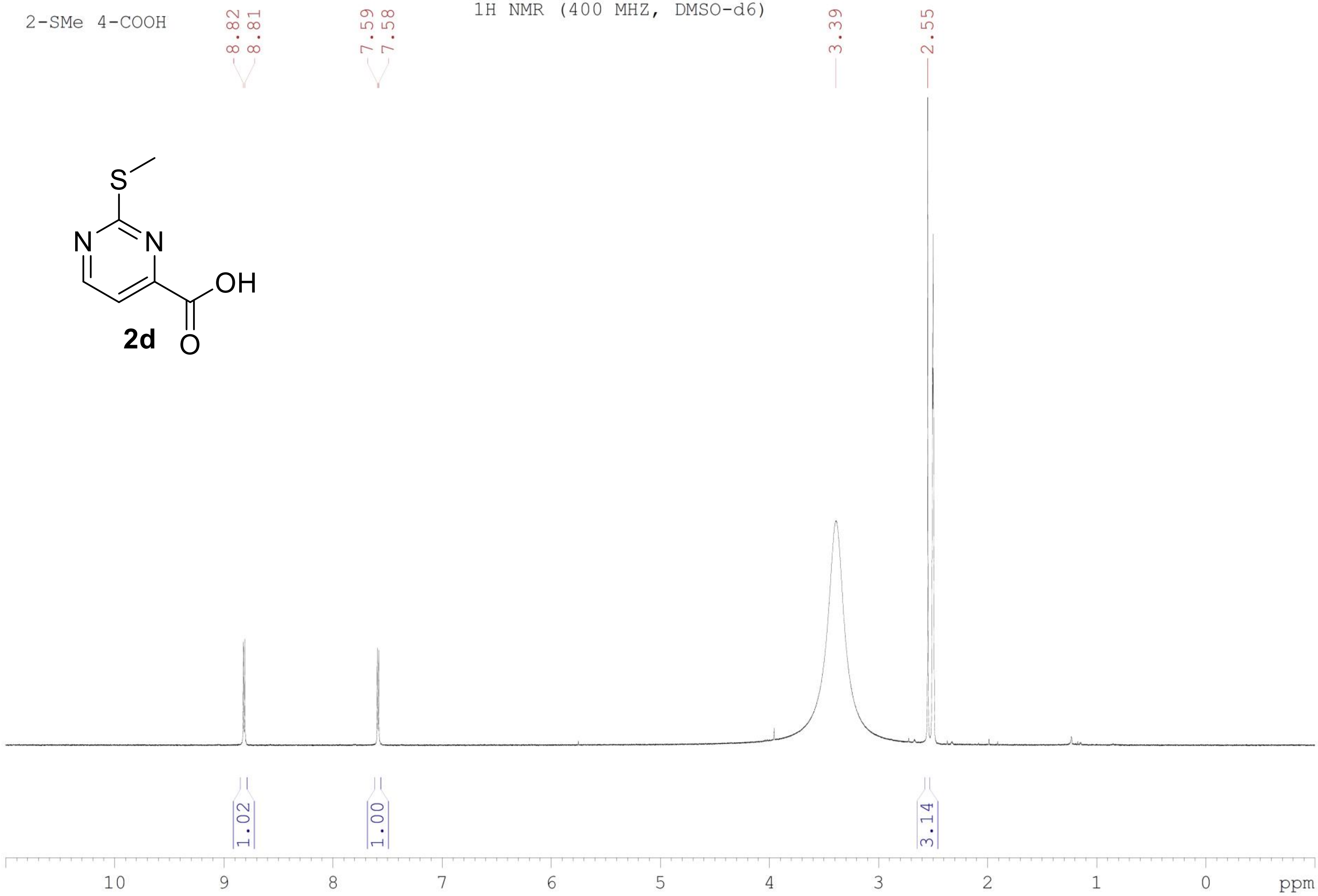
2-SMe 4-COOH
1H NMR (400 MHZ, DMSO-d6)
8.82
8.81
7.59
7.58
3.39
2.55
2d
S
N
N
OH
O
1.02
1.00
3.14
10
9
8
7
6
5
4
3
2
1
0
ppm

2-SMe 4-Ph

1H NMR (400 MHZ, DMSO-d6)

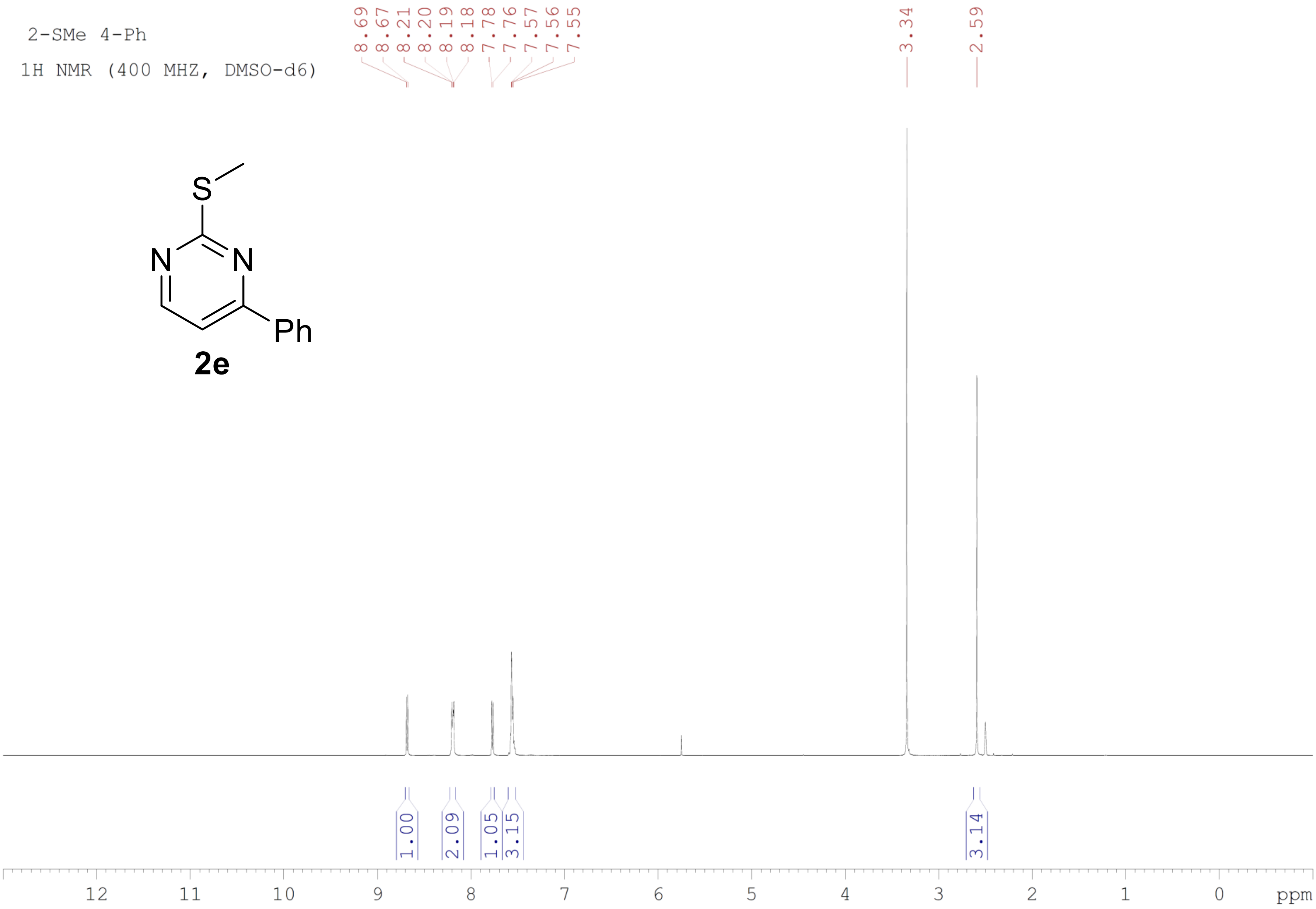

2-SMe 4-NH2
1H NMR (400 MHZ, DMSO-d6)

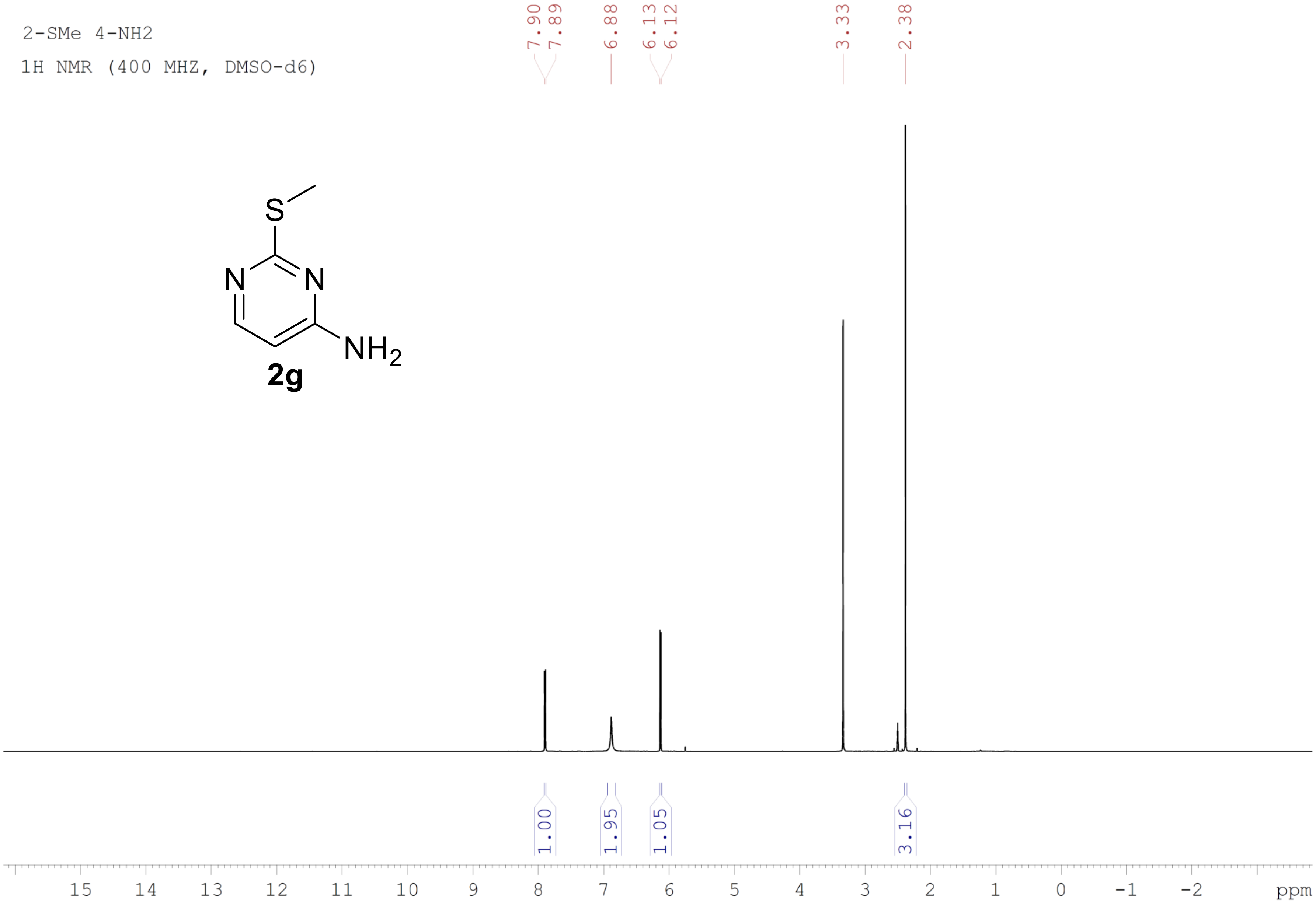

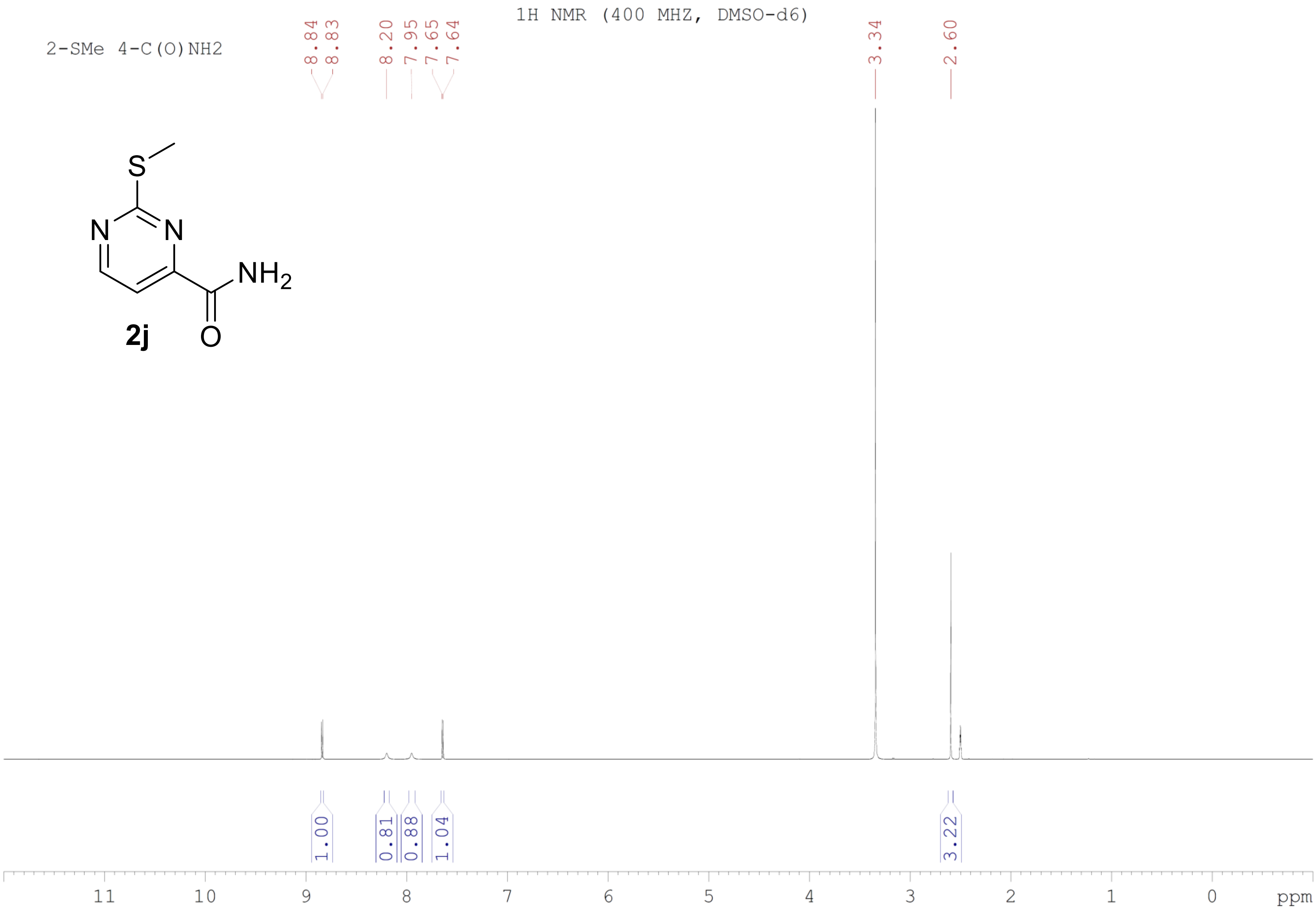
2-SMe 4-C(O)NH2
1H NMR (400 MHZ, DMSO-d6)
8.84
8.83
8.20
7.95
7.65
7.64
3.34
2.60
S
N
N
NH2
O
2j
1.00
0.81
0.88
1.04
3.22
11
10
9
8
7
6
5
4
3
2
1
0
ppm

2-SMe 4-C(O)NH2

13C NMR (100 MHZ, DMSO-d6)

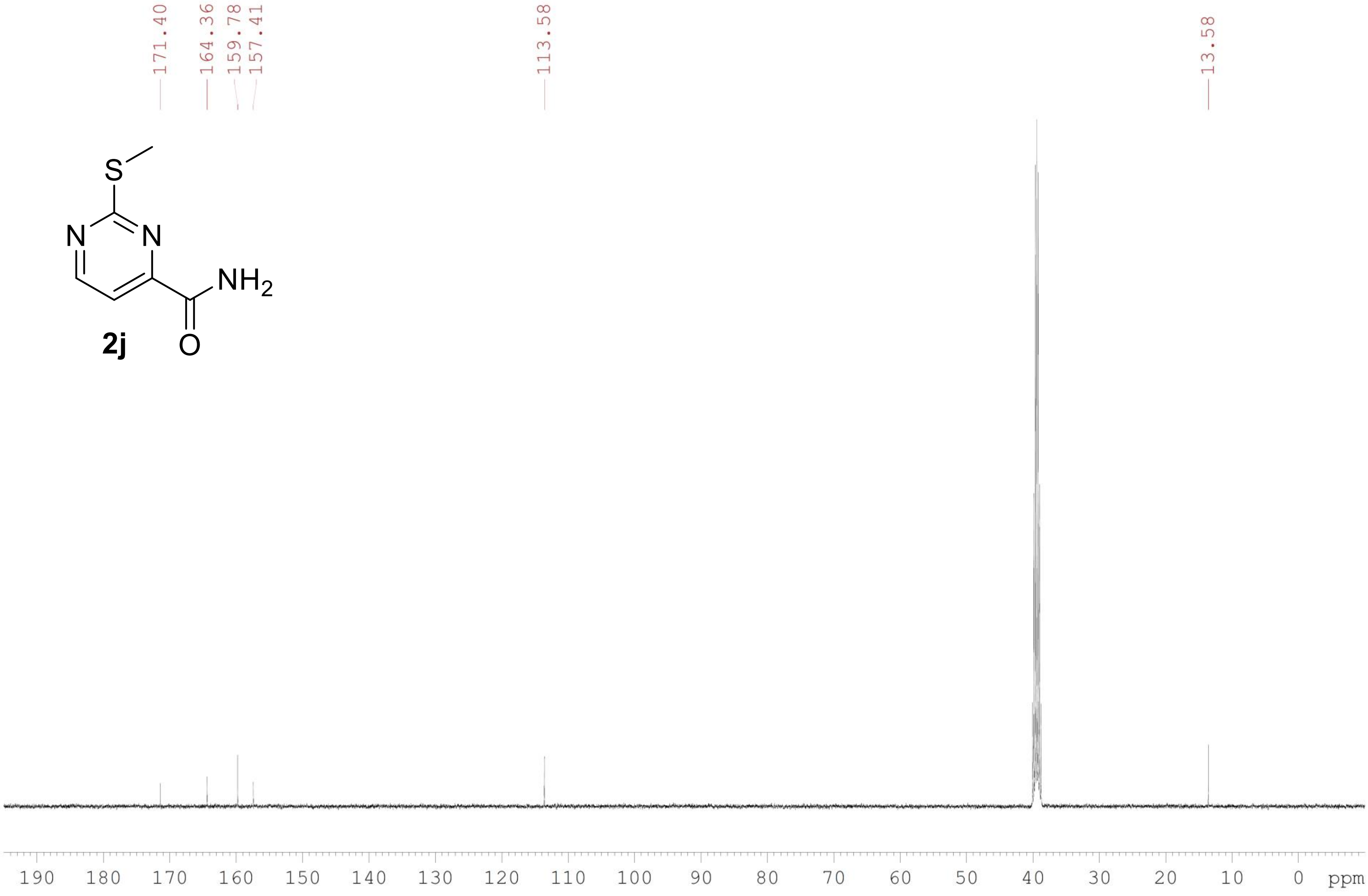

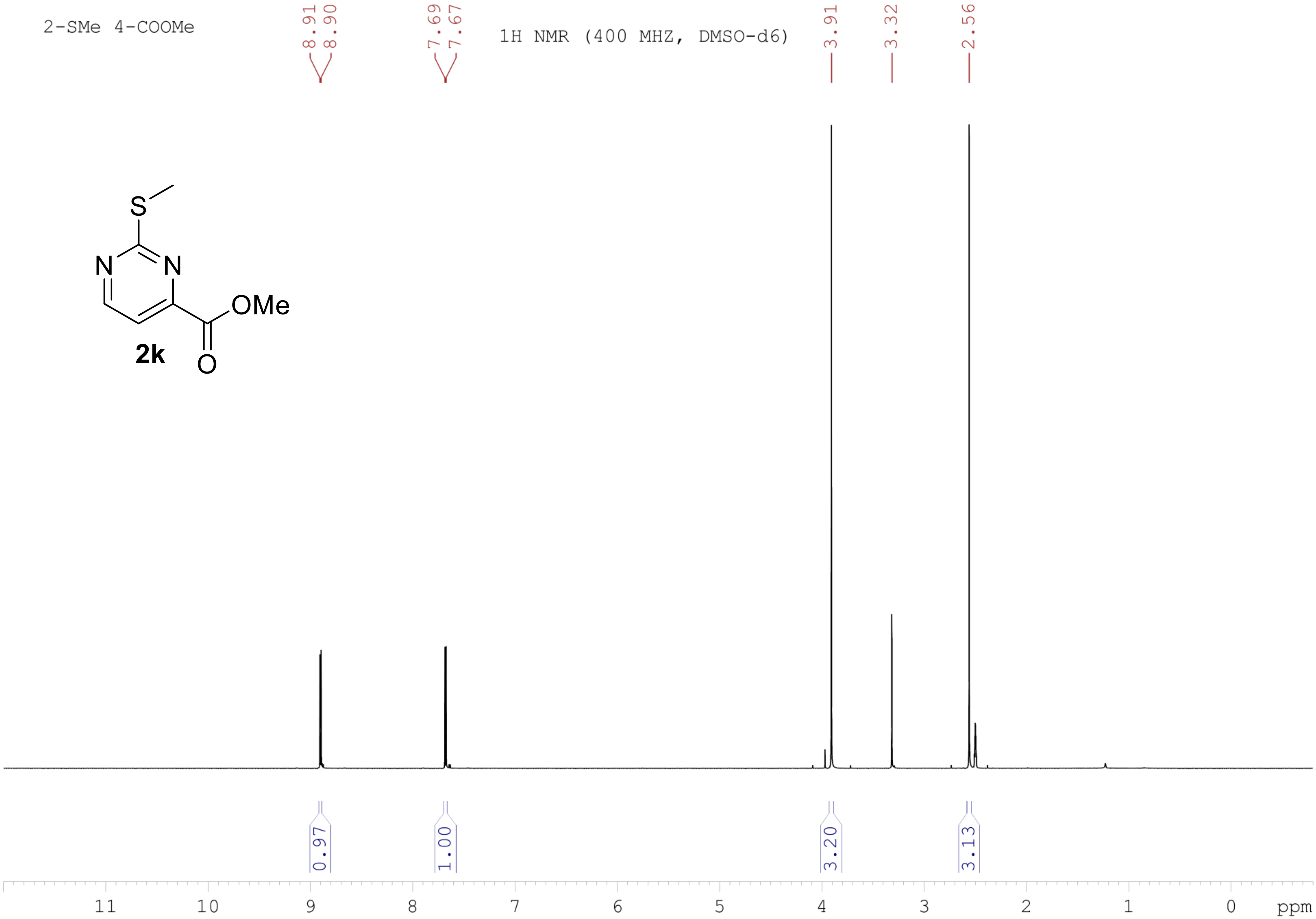
2-SMe 4-COOMe
1H NMR (400 MHZ, DMSO-d6)
8.91
8.90
7.69
7.67
3.91
3.32
2.56
S
N
N
OMe
O
2k
0.97
1.00
3.20
3.13
11
10
9
8
7
6
5
4
3
2
1
0
ppm

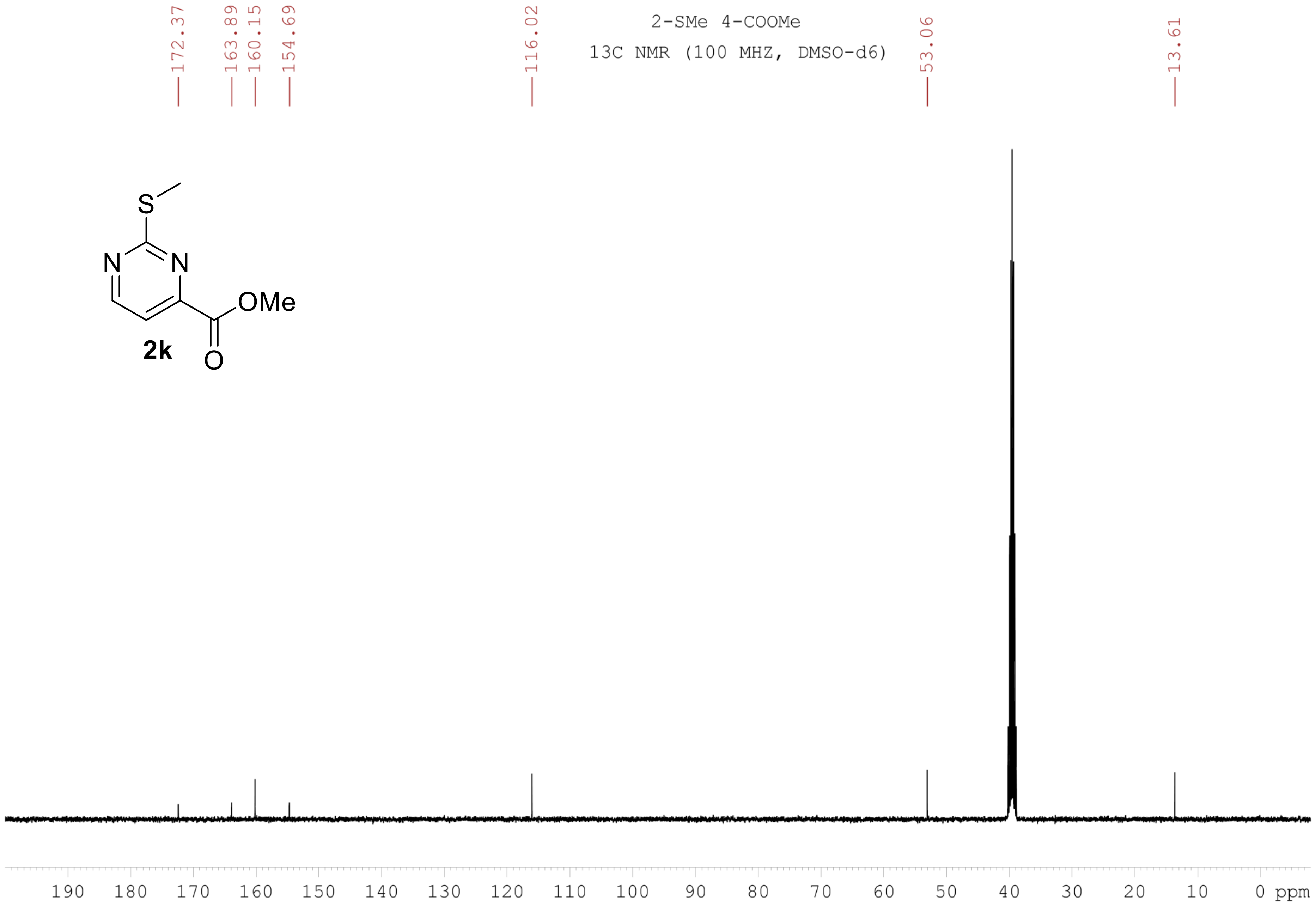

2-SMe 4-COOMe
13C NMR (100 MHZ, DMSO-d6)
172.37
163.89
160.15
154.69
116.02
53.06
13.61
S
N
N
OMe
O
2k
190
180
170
160
150
140
130
120
110
100
90
80
70
60
50
40
30
20
10
0 ppm

2-SMe 4-CF3

1H NMR (400 MHZ, DMSO-d6)

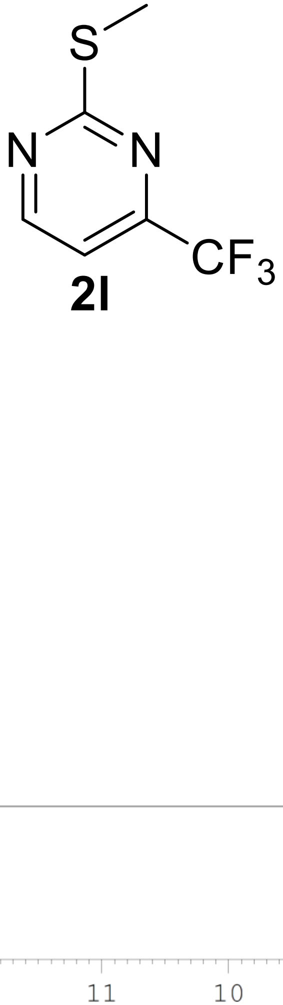

2l

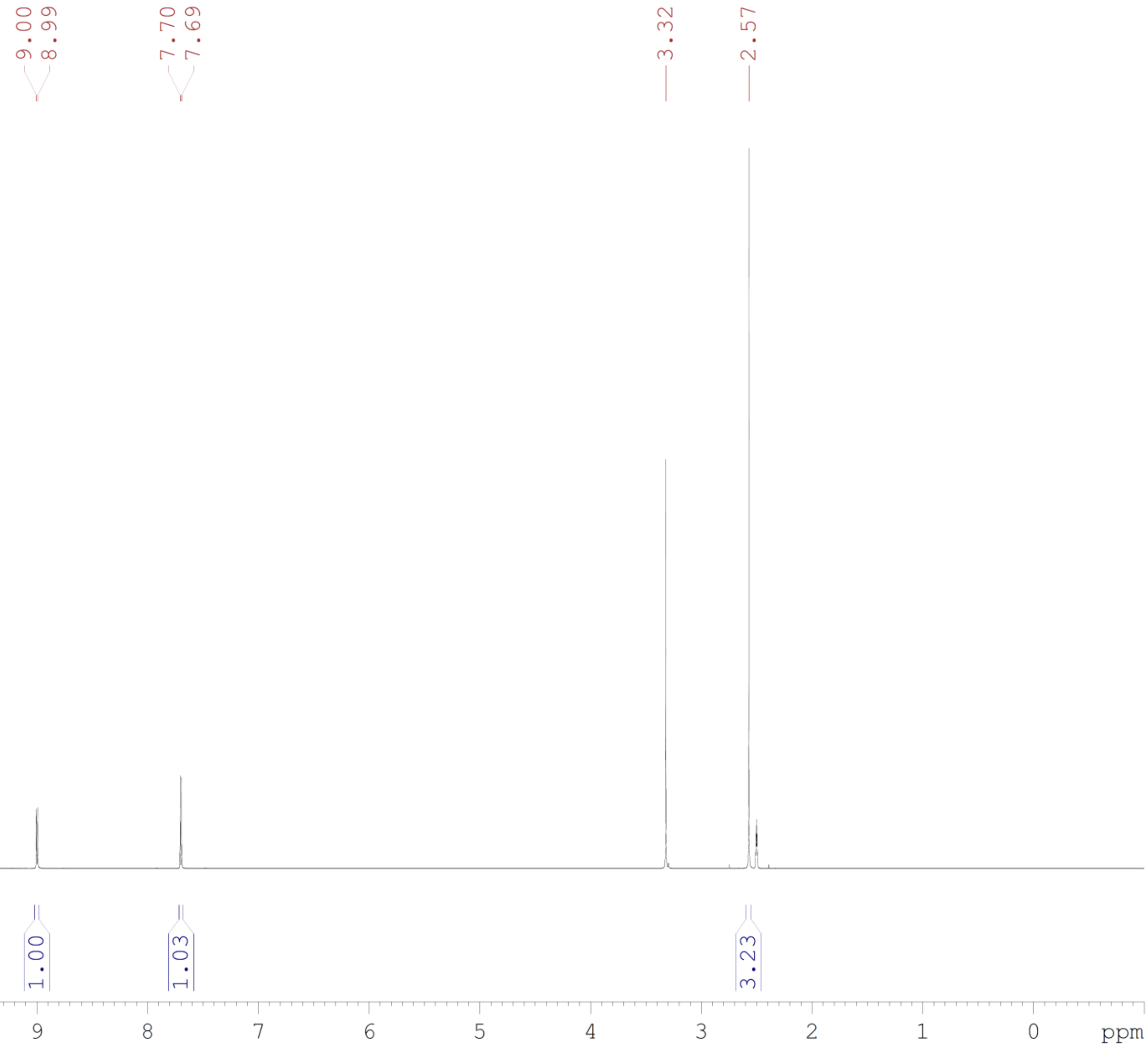

2-SMe 4-CF3

19F NMR (376 MHZ, DMSO-d6)

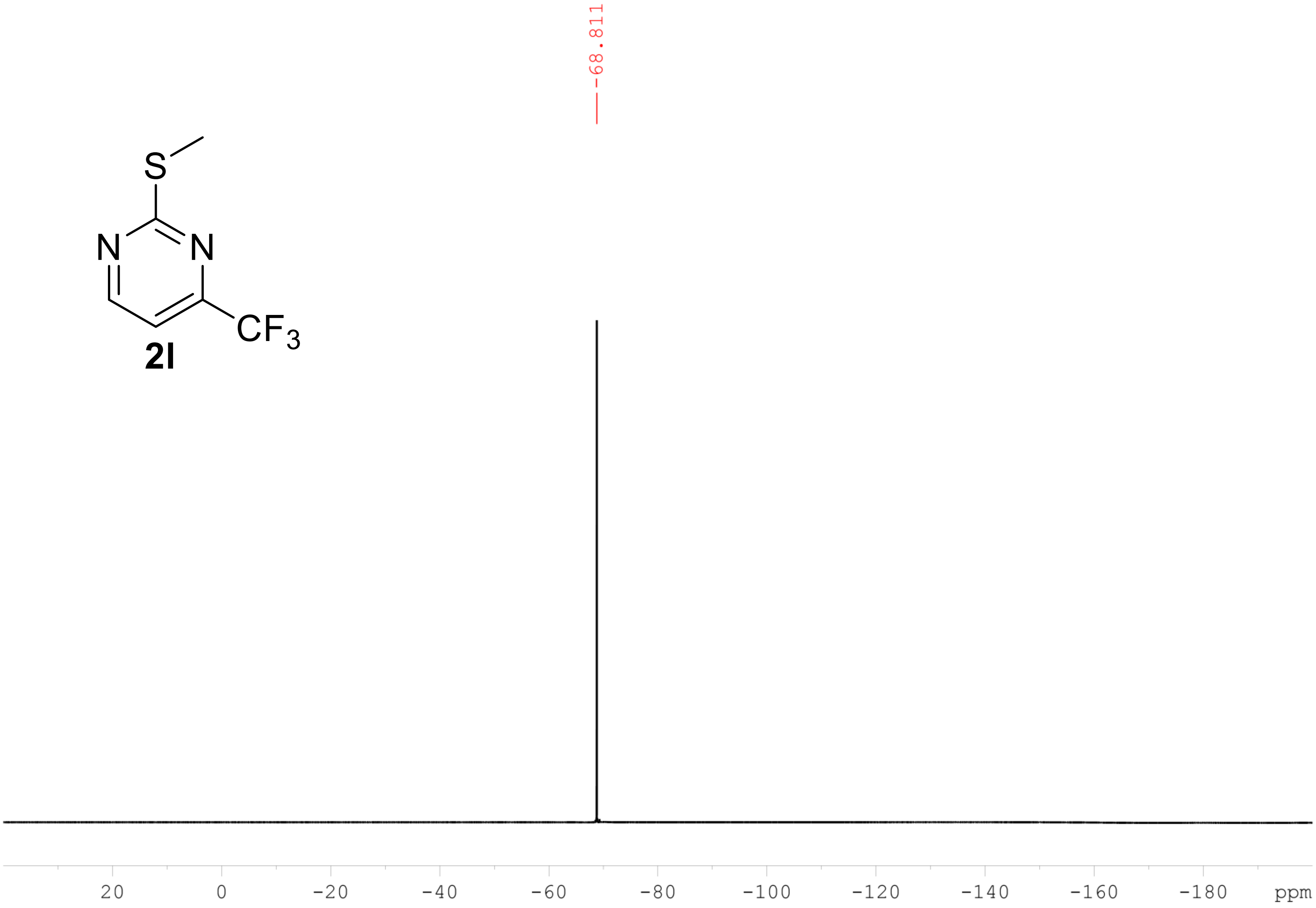

2-SMe 5-NH2

1H NMR (400 MHZ, DMSO-d6)

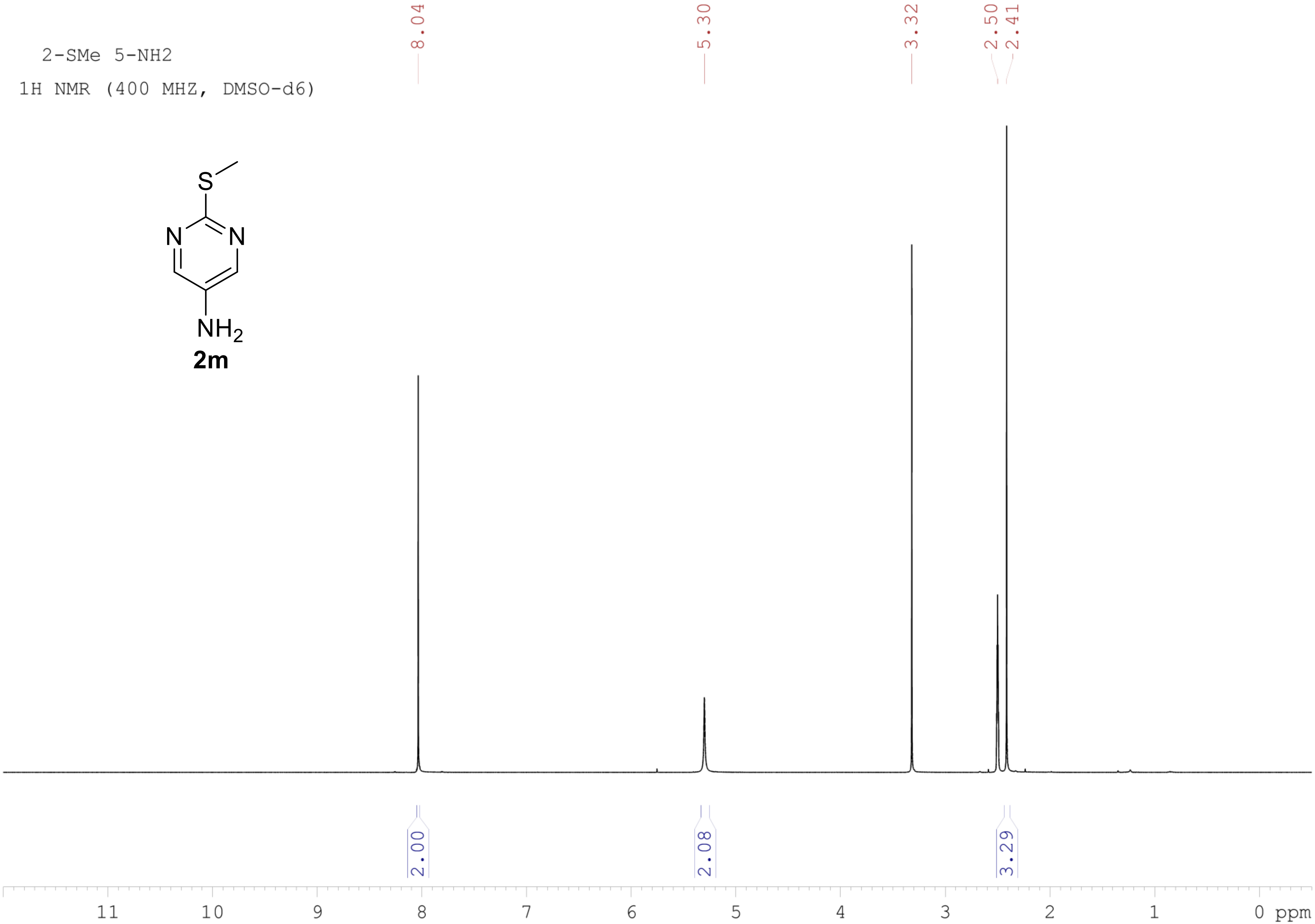

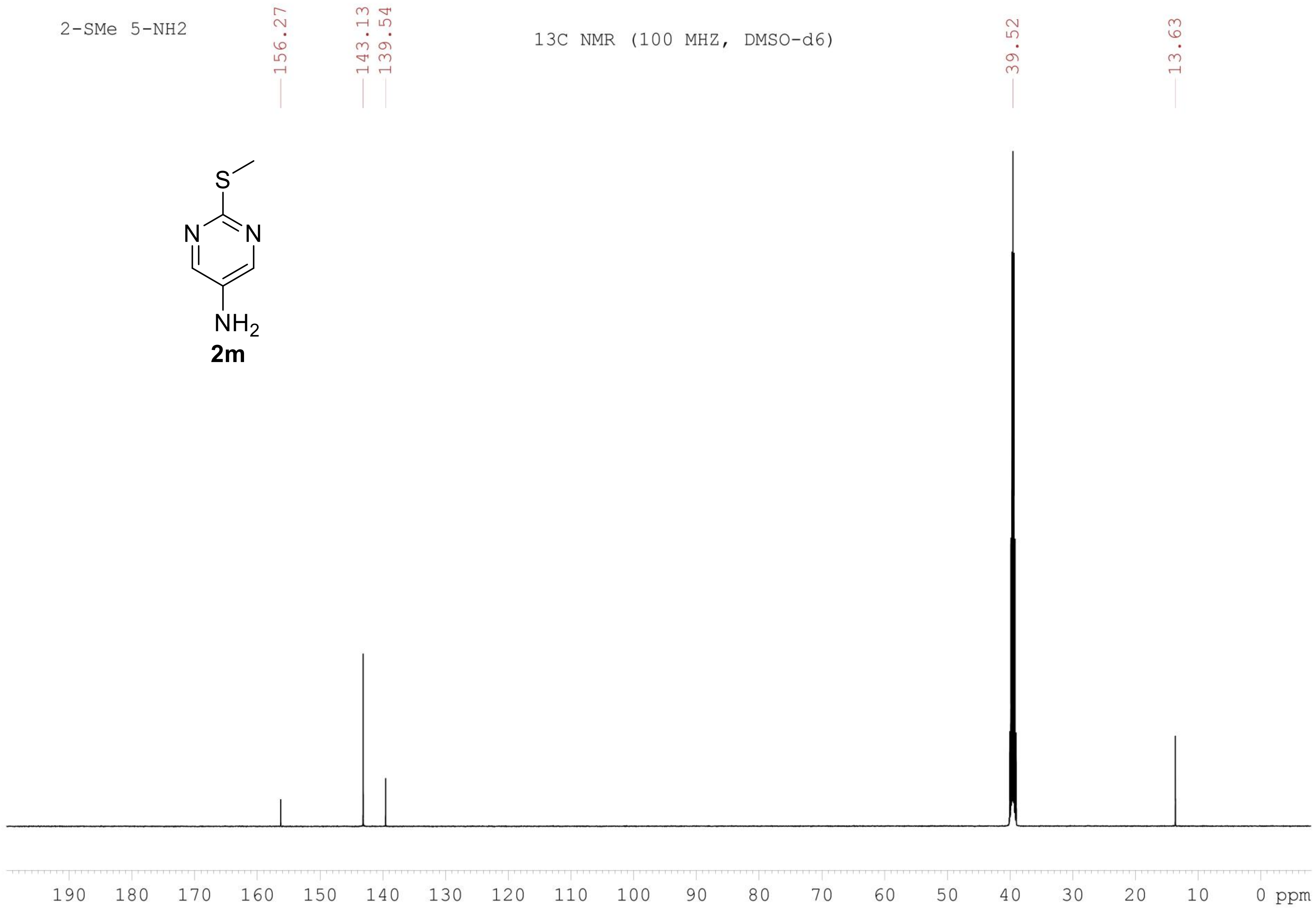
2-SMe 5-NH2
13C NMR (100 MHZ, DMSO-d6)
156.27
143.13
139.54
39.52
13.63
NH_2
2m
190 180 170 160 150 140 130 120 110 100 90 80 70 60 50 40 30 20 10 0 ppm

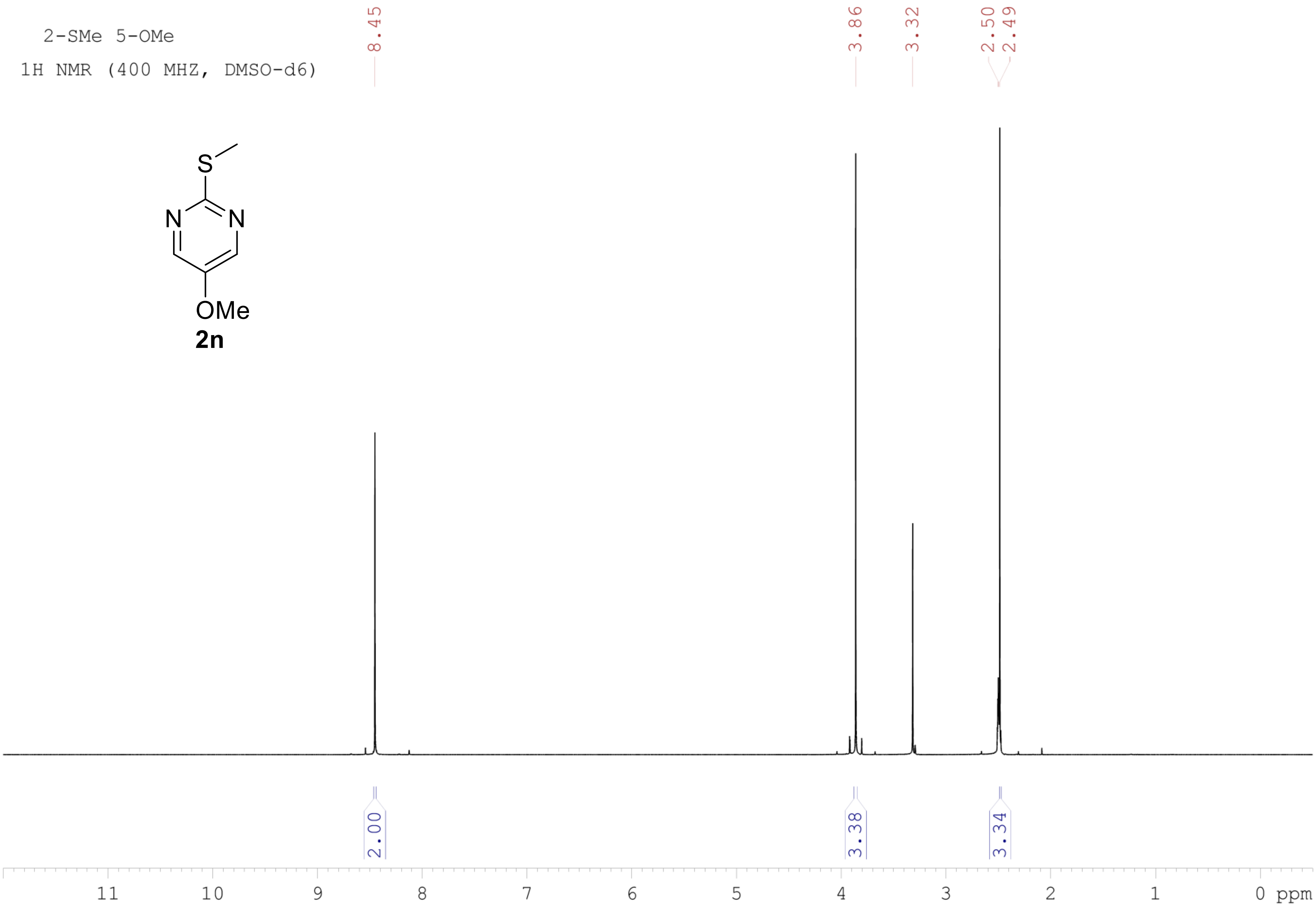
2-SMe 5-OMe
1H NMR (400 MHZ, DMSO-d6)
8.45
3.86
3.32
2.50
2.49
S
N
N
OMe
2n
2.00
3.38
3.34
11
10
9
8
7
6
5
4
3
2
1
0 ppm

2-SMe 5-Me

1H NMR (400 MHZ, DMSO-d6)

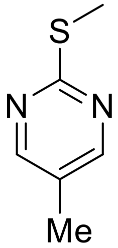

2o

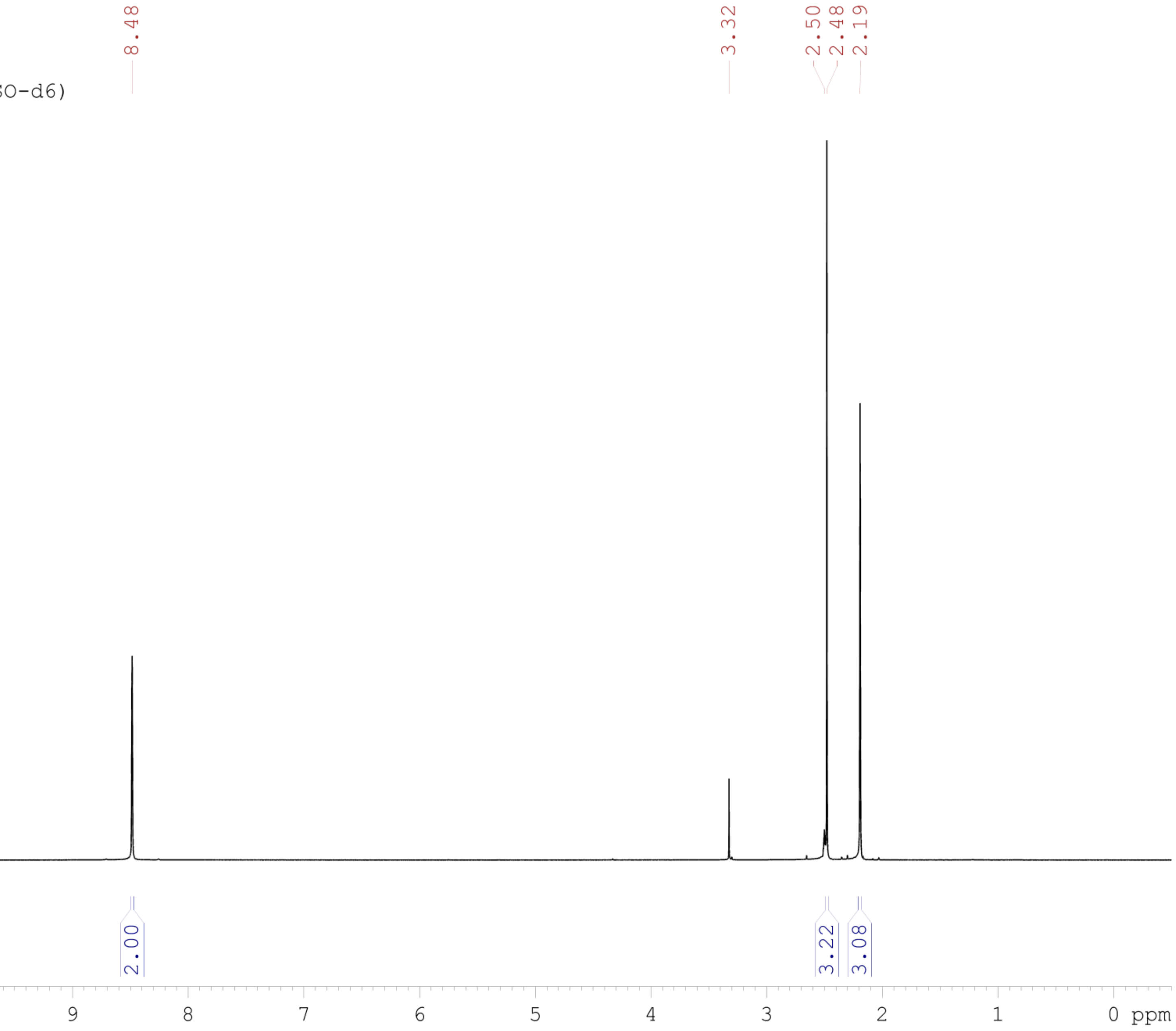

2-SMe 5-Me

13C NMR (100 MHZ, DMSO-d6)

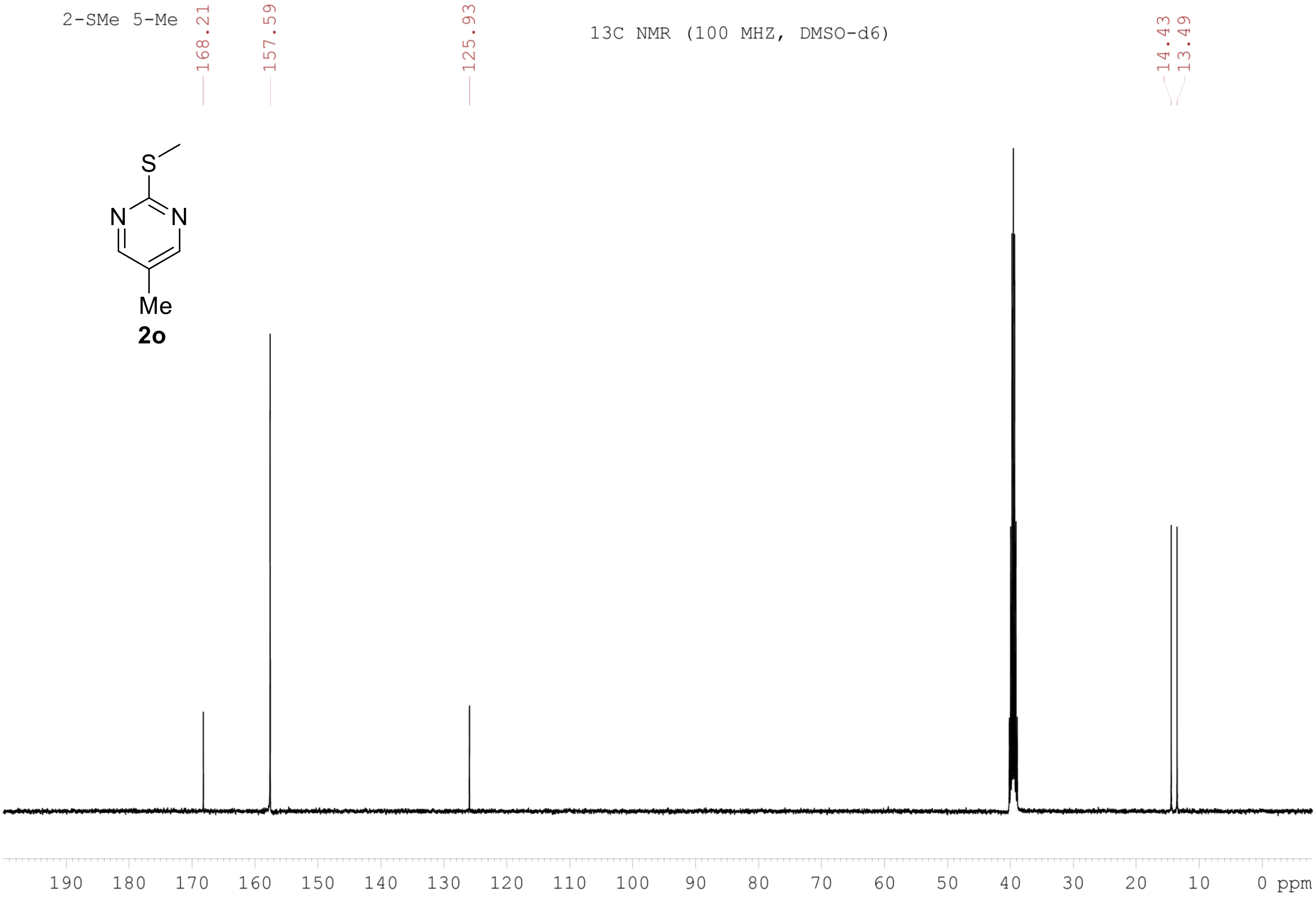

2o

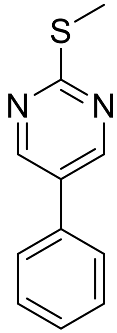
S
N
N

2p

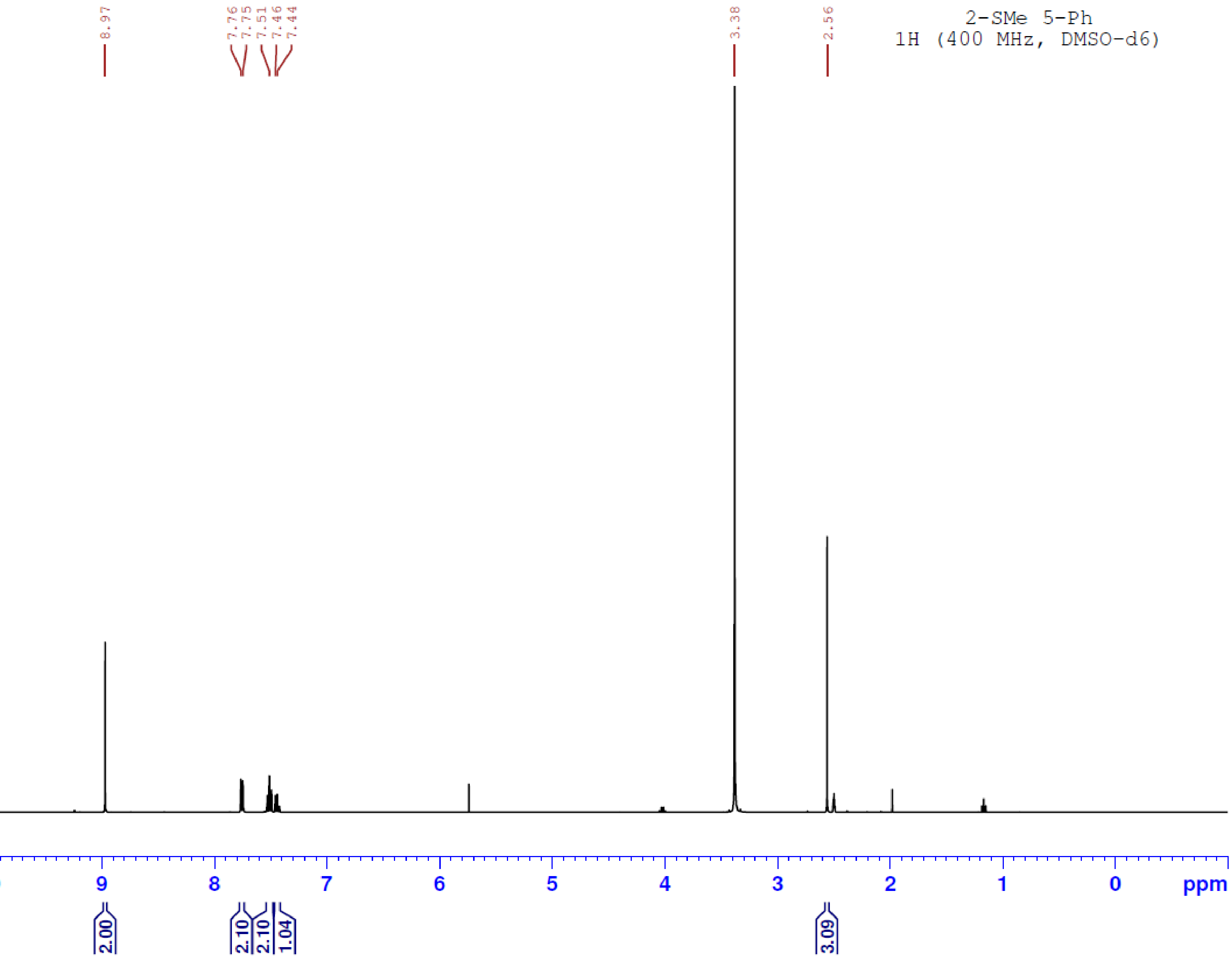
2-SMe 5-Ph
1H (400 MHz, DMSO-d6)
8.97
7.76
7.75
7.51
7.46
7.44
3.38
2.56
10
9
8
7
6
5
4
3
2
1
0
ppm
2.00
2.10
2.10
1.04
3.09

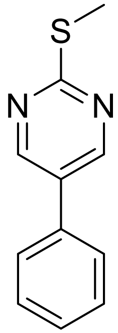
S
N
N

2p

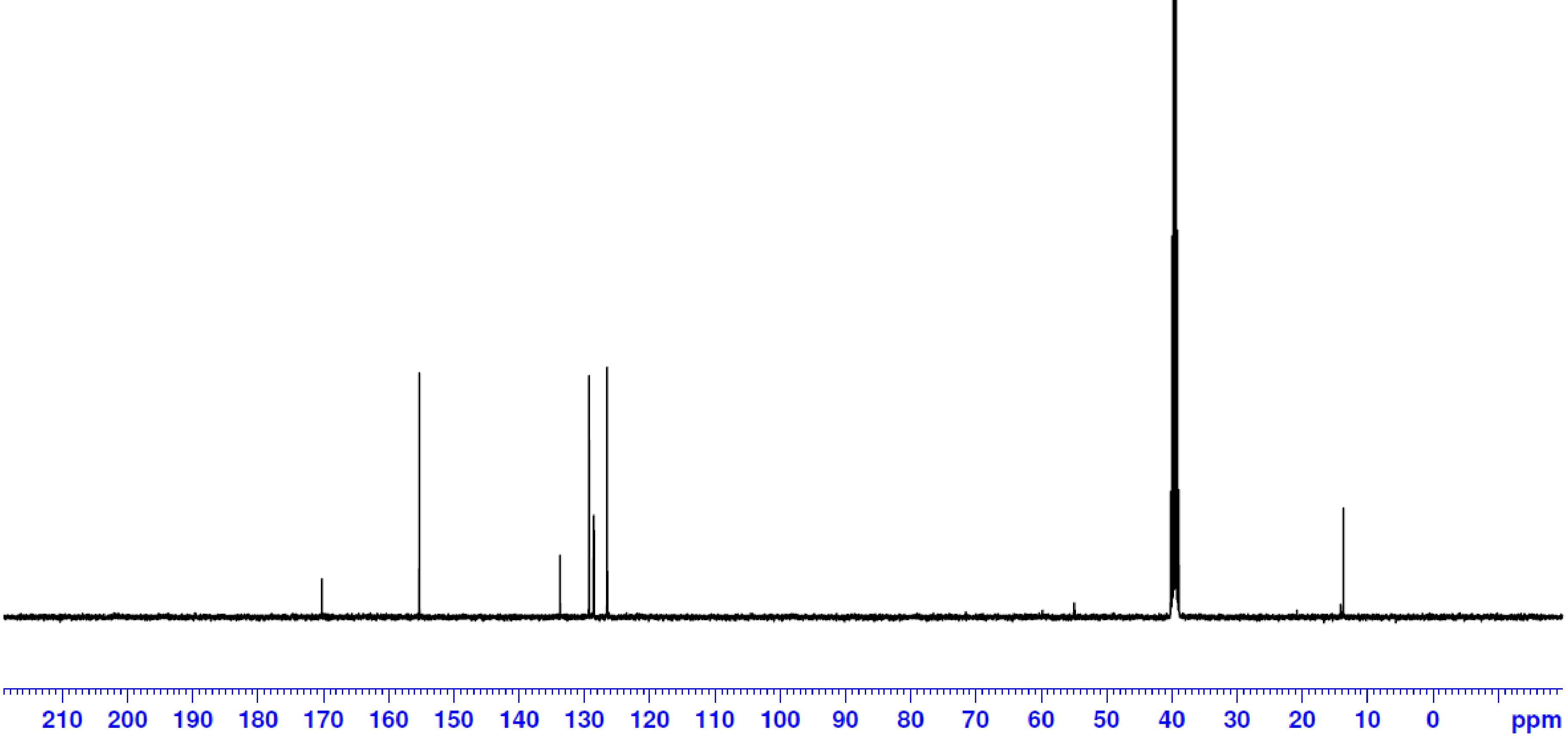
170.24
155.31
133.72
129.29
128.62
128.55
126.53
13.69
2-SMe 5-Ph
13C (100 MHz, DMSO-d6)
210
200
190
180
170
160
150
140
130
120
110
100
90
80
70
60
50
40
30
20
10
0
ppm

2-SMe pyrimidine ref

1H NMR (400 MHZ, DMSO-d6)

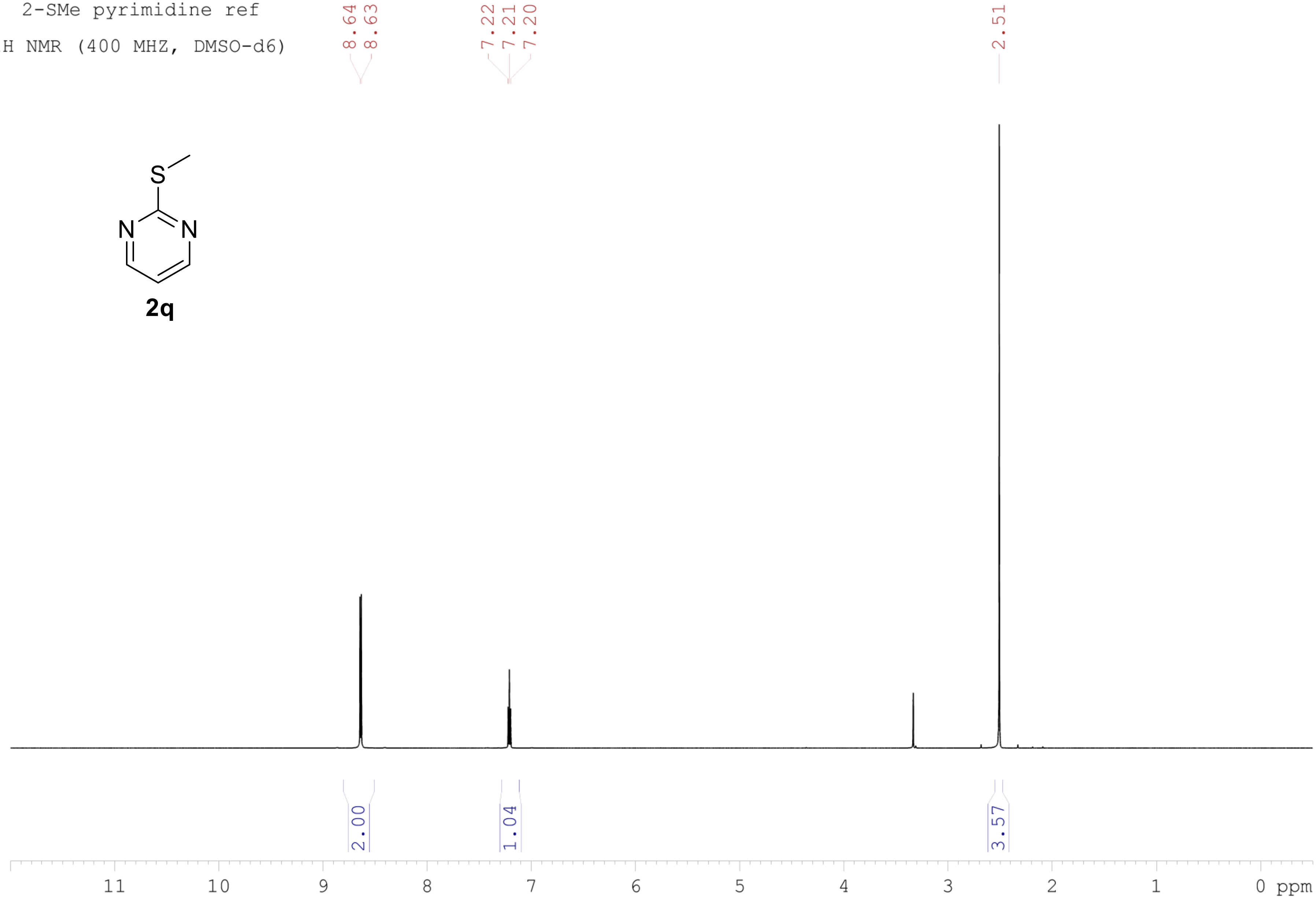

2q

2-SMe 5-F

1H NMR (400 MHZ, DMSO-d6)

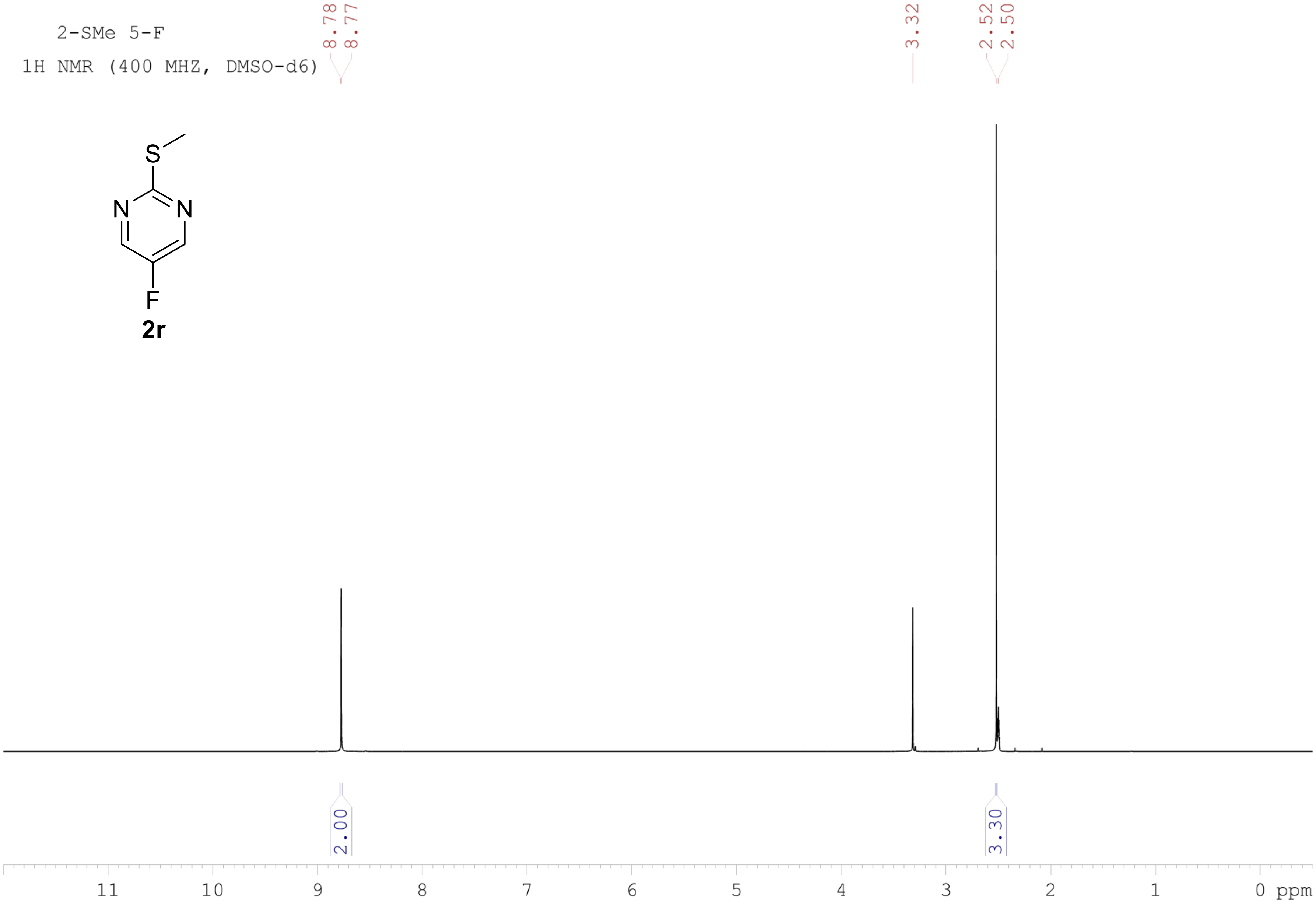

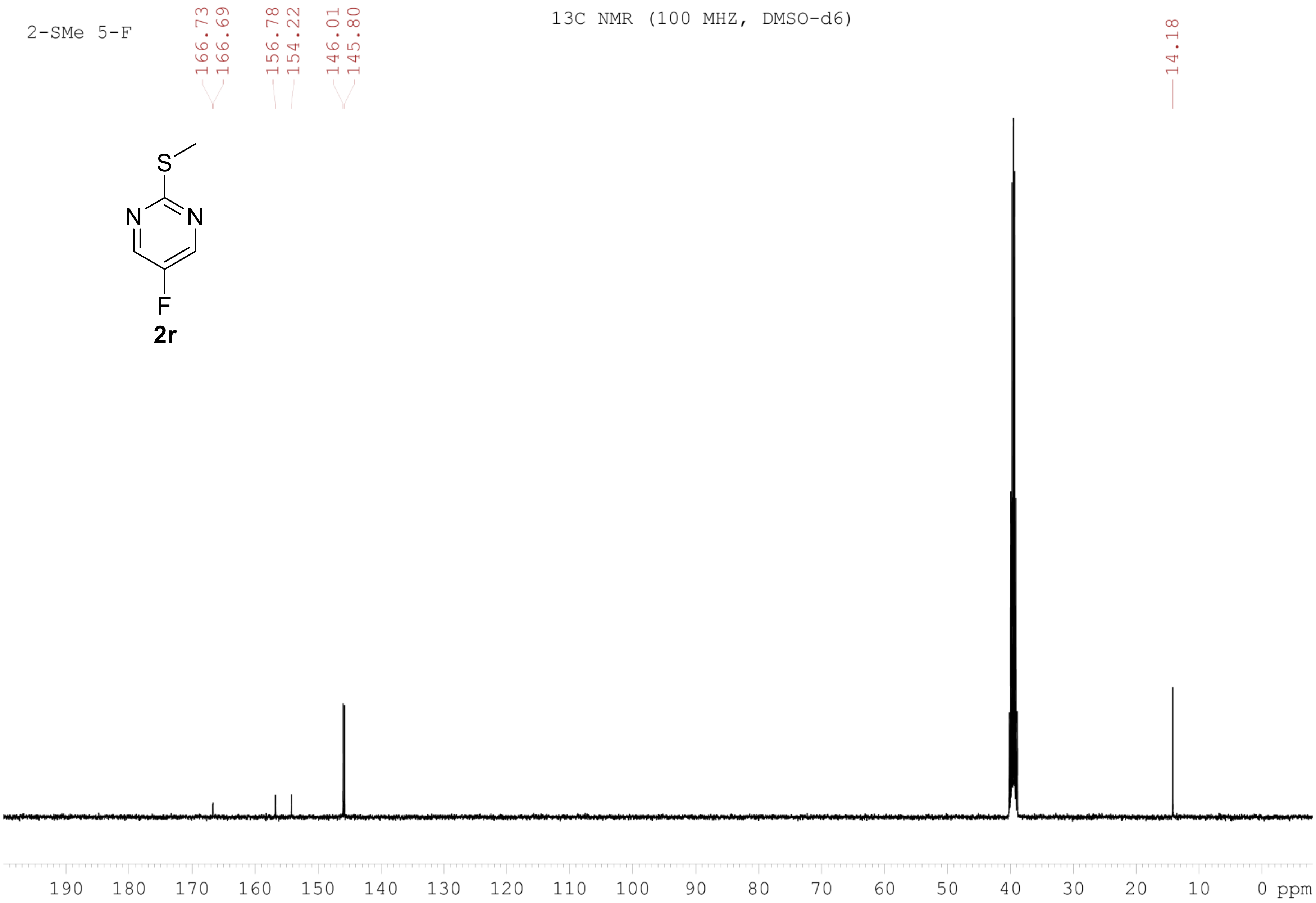
2-SMe 5-F
13C NMR (100 MHZ, DMSO-d6)
166.73
166.69
156.78
154.22
146.01
145.80
14.18
S
N
N
F
2r
190 180 170 160 150 140 130 120 110 100 90 80 70 60 50 40 30 20 10 0 ppm

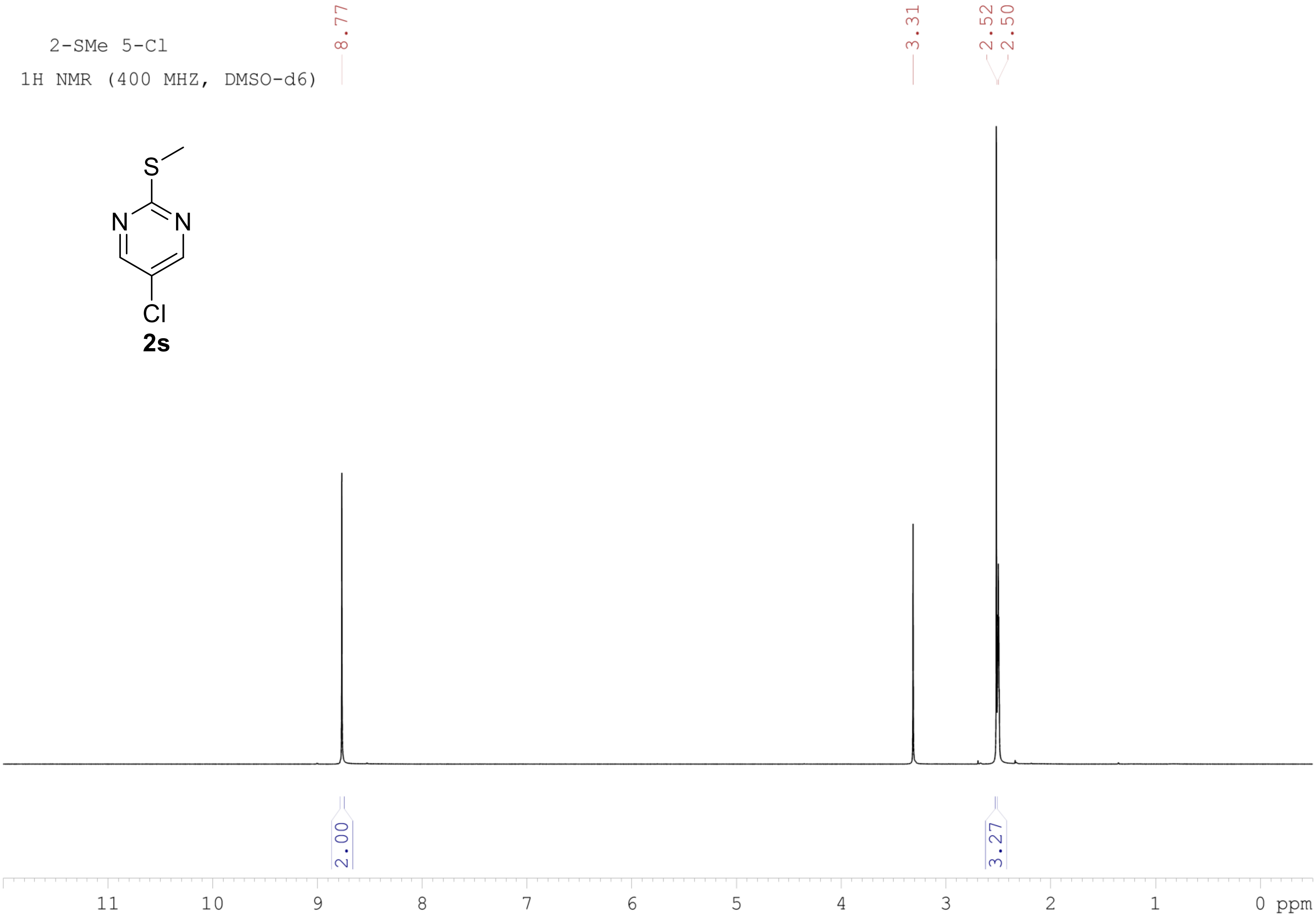
2-SMe 5-Cl
1H NMR (400 MHZ, DMSO-d6)
8.77
3.31
2.52
2.50
2s
2.00
3.27
11
10
9
8
7
6
5
4
3
2
1
0 ppm

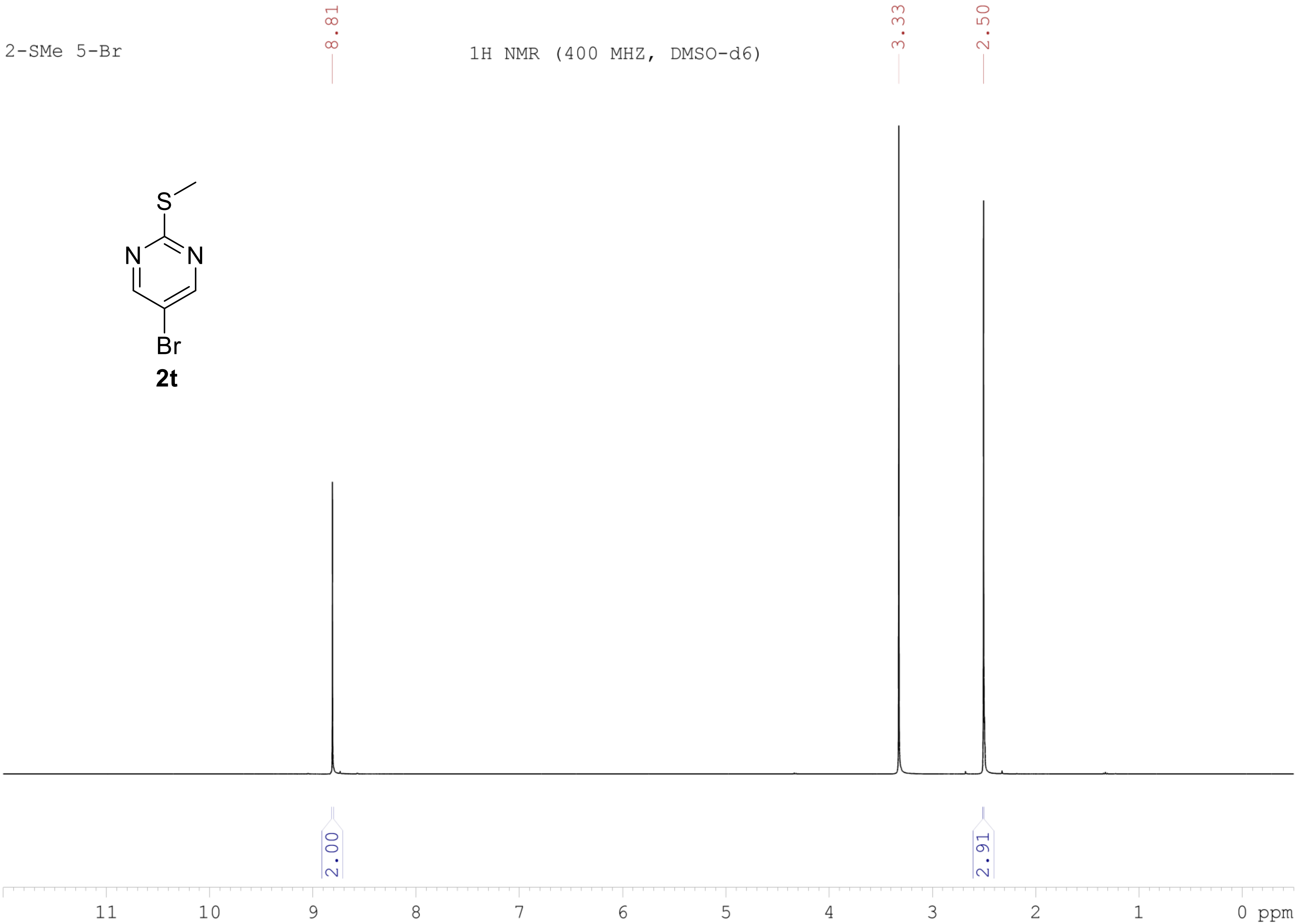
2-SMe 5-Br
1H NMR (400 MHZ, DMSO-d6)
8.81
3.33
2.50
S
N
N
Br
2t
2.00
2.91
11
10
9
8
7
6
5
4
3
2
1
0 ppm

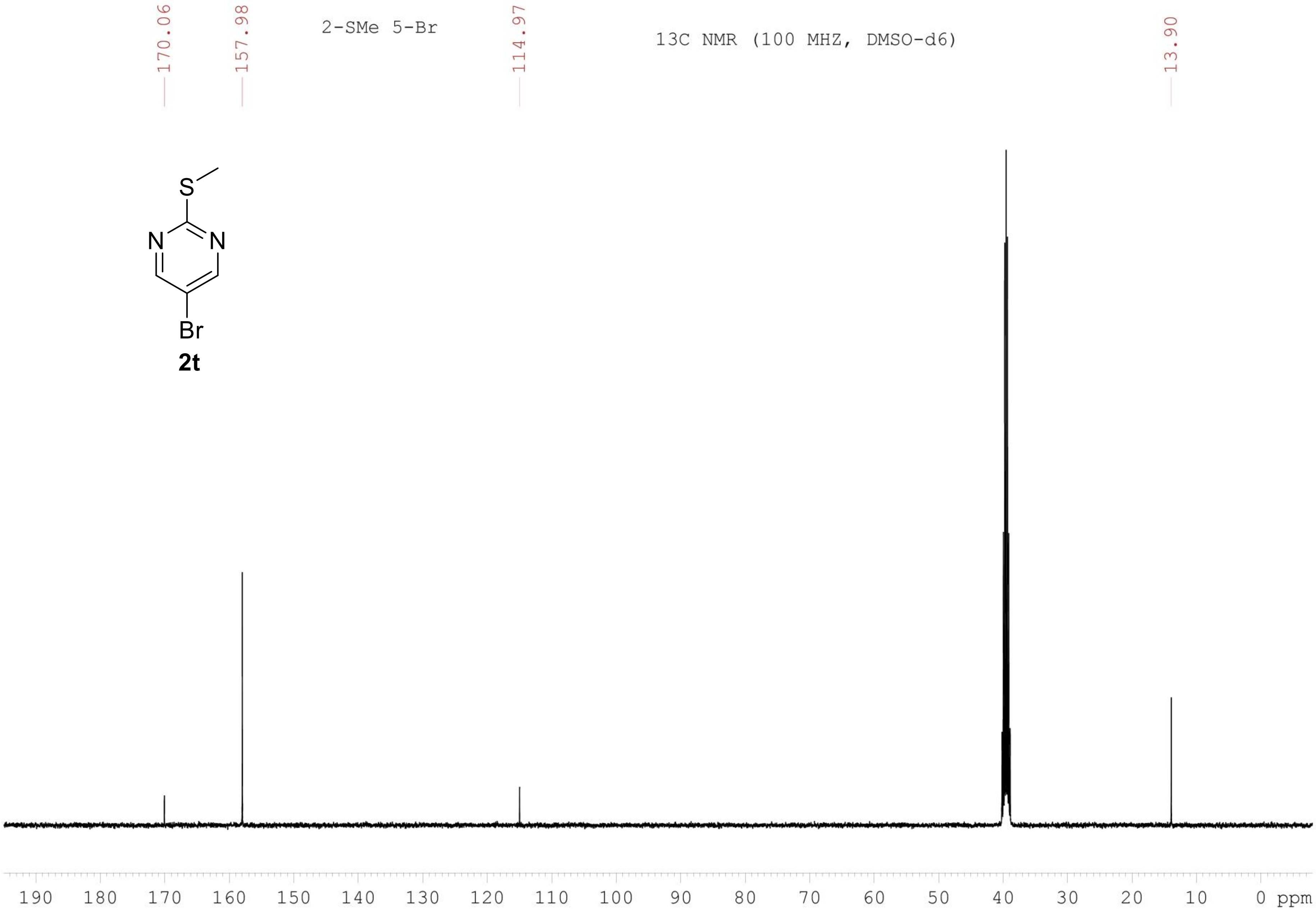
170.06
157.98
2-SMe 5-Br
114.97
13C NMR (100 MHZ, DMSO-d6)
13.90
S
N
N
Br
2t
190
180
170
160
150
140
130
120
110
100
90
80
70
60
50
40
30
20
10
0 ppm

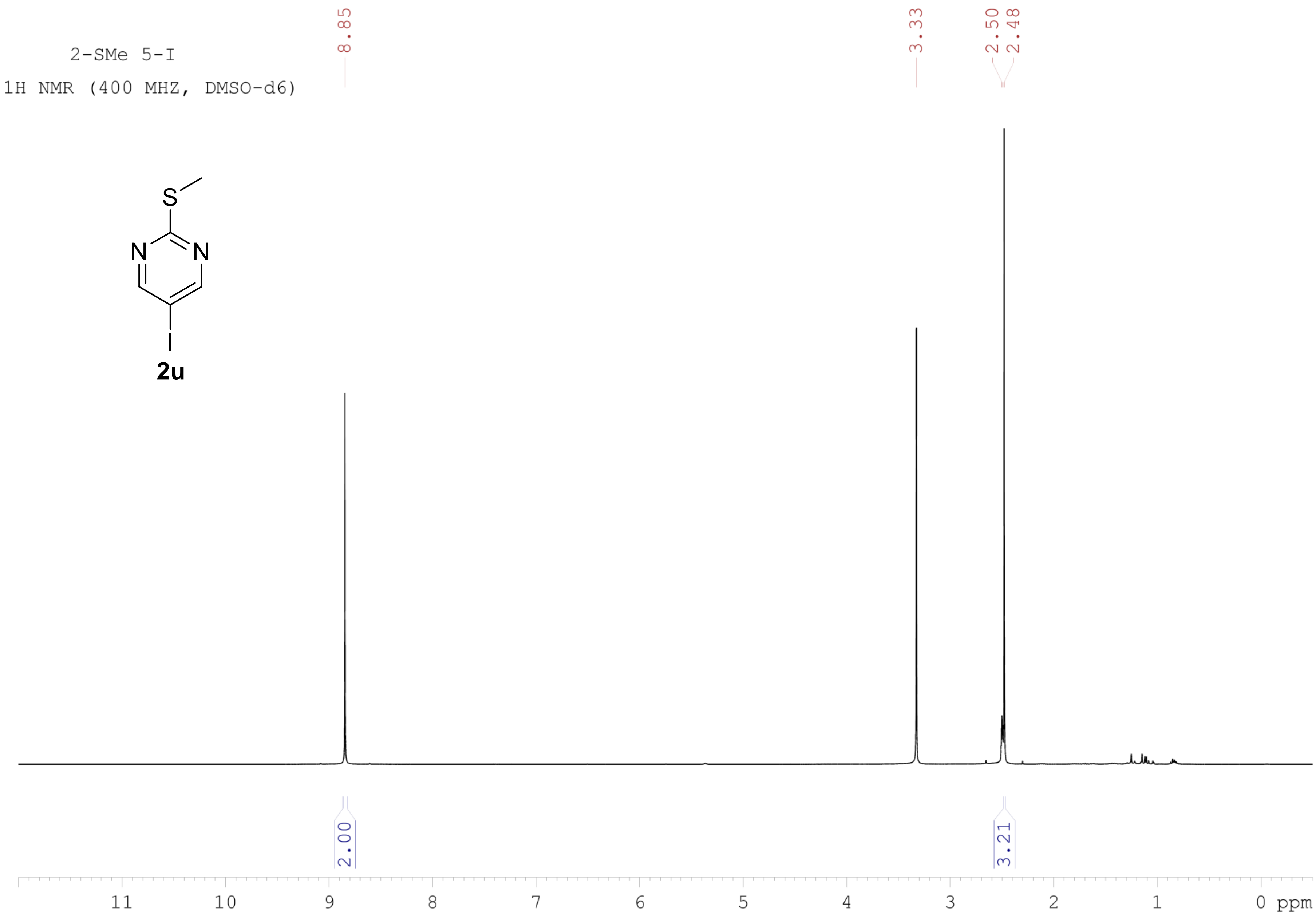
2-SMe 5-I
1H NMR (400 MHZ, DMSO-d6)
8.85
3.33
2.50
2.48
S
N
N
I
2u
2.00
3.21
11
10
9
8
7
6
5
4
3
2
1
0 ppm

2-SMe 5-I

13C NMR (100 MHZ, DMSO-d6)

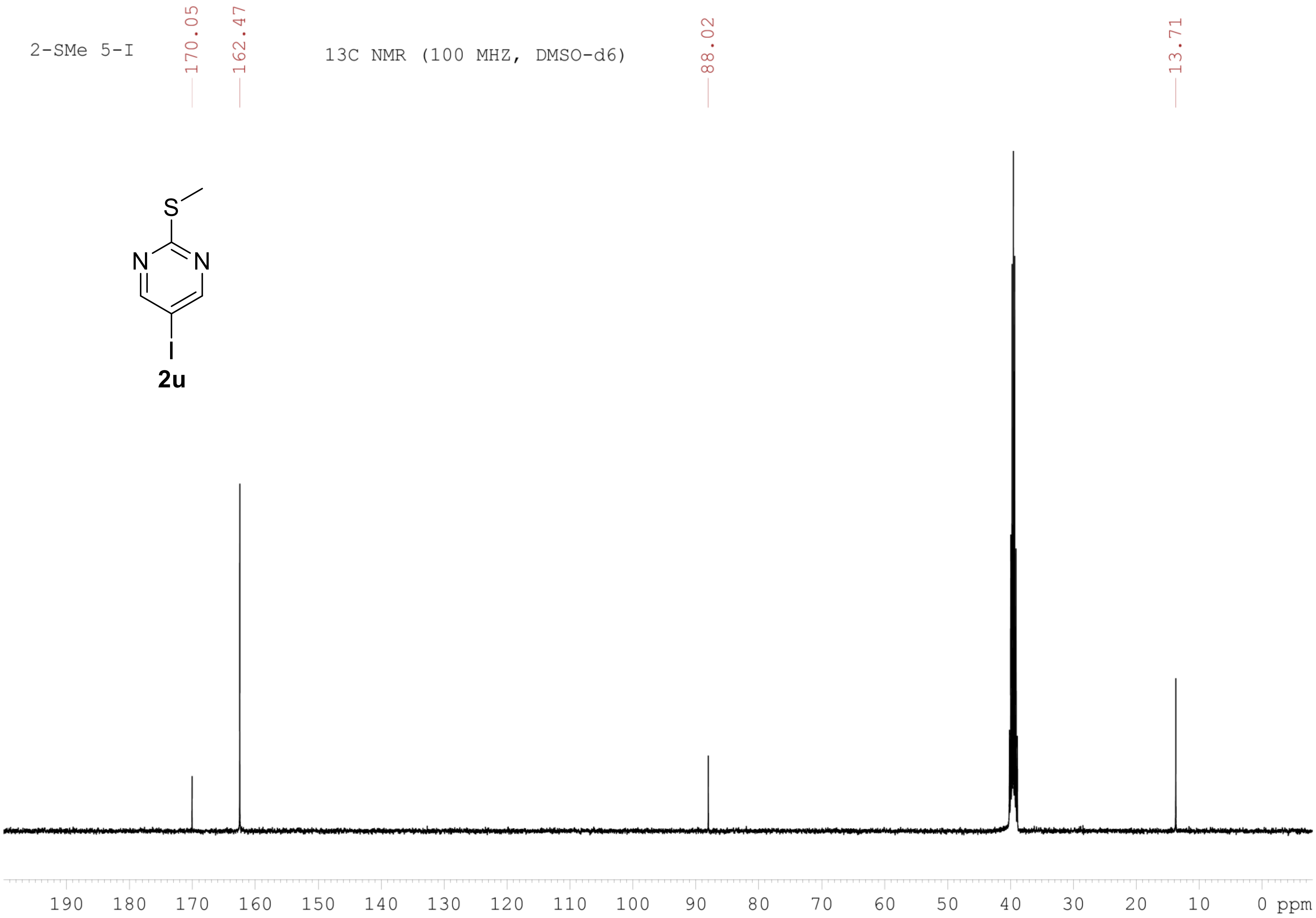

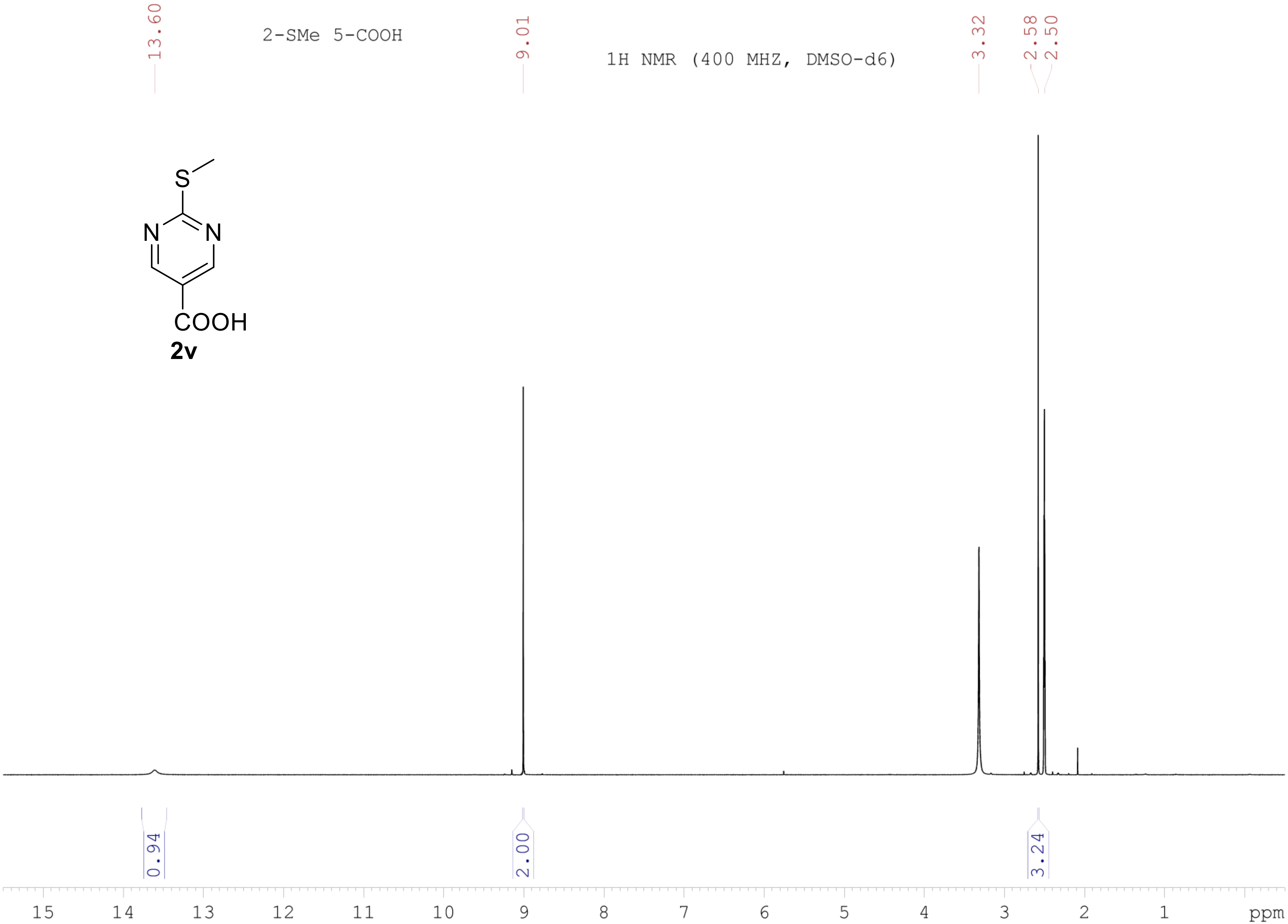

2-SMe 5-COOH
1H NMR (400 MHZ, DMSO-d6)
13.60
9.01
3.32
2.58
2.50
S
N
N
COOH
2v
0.94
2.00
3.24
15 14 13 12 11 10 9 8 7 6 5 4 3 2 1 ppm

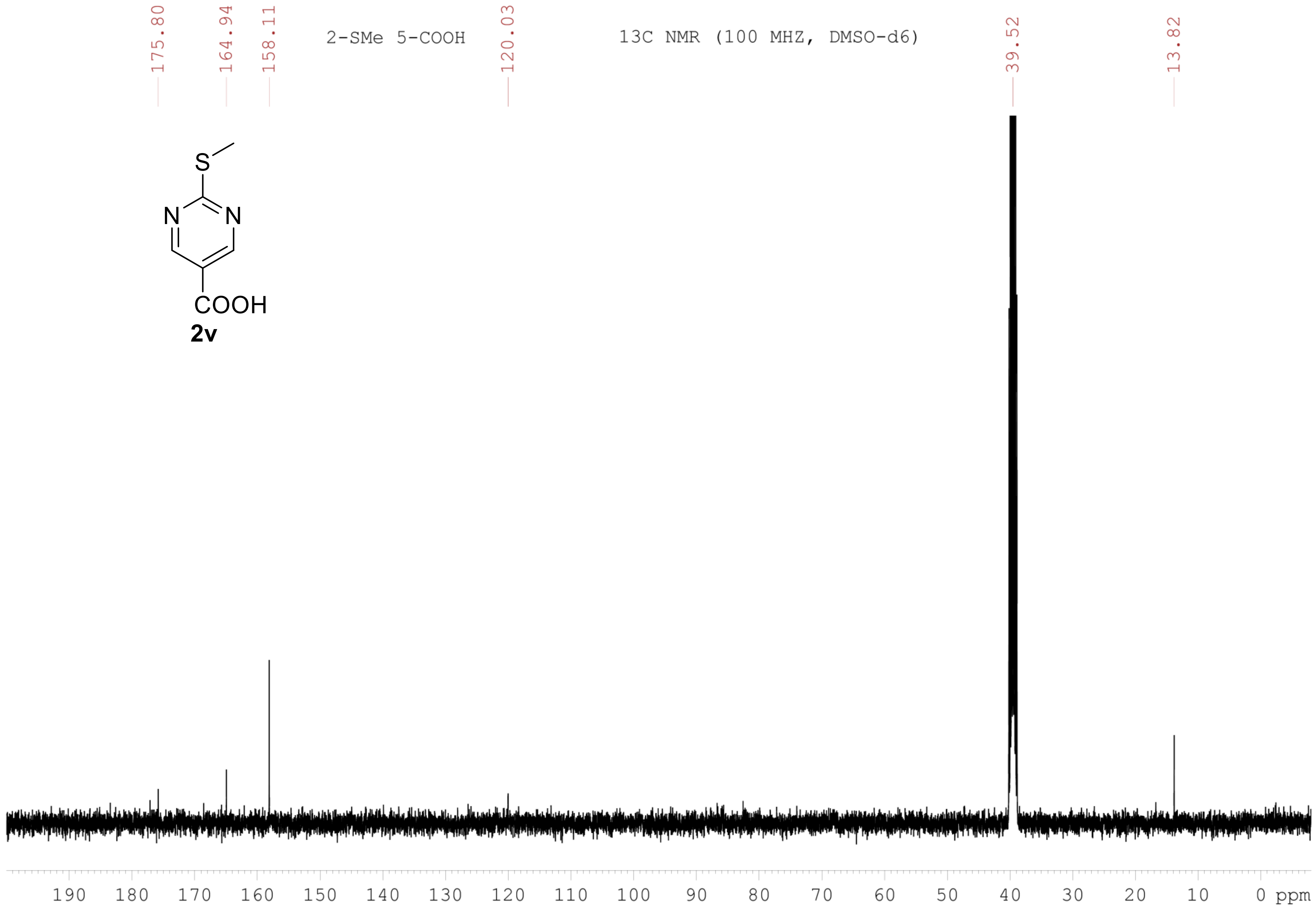
175.80
164.94
158.11
2-SMe 5-COOH
120.03
13C NMR (100 MHZ, DMSO-d6)
39.52
13.82
S
N
N
COOH
2v
190 180 170 160 150 140 130 120 110 100 90 80 70 60 50 40 30 20 10 0 ppm

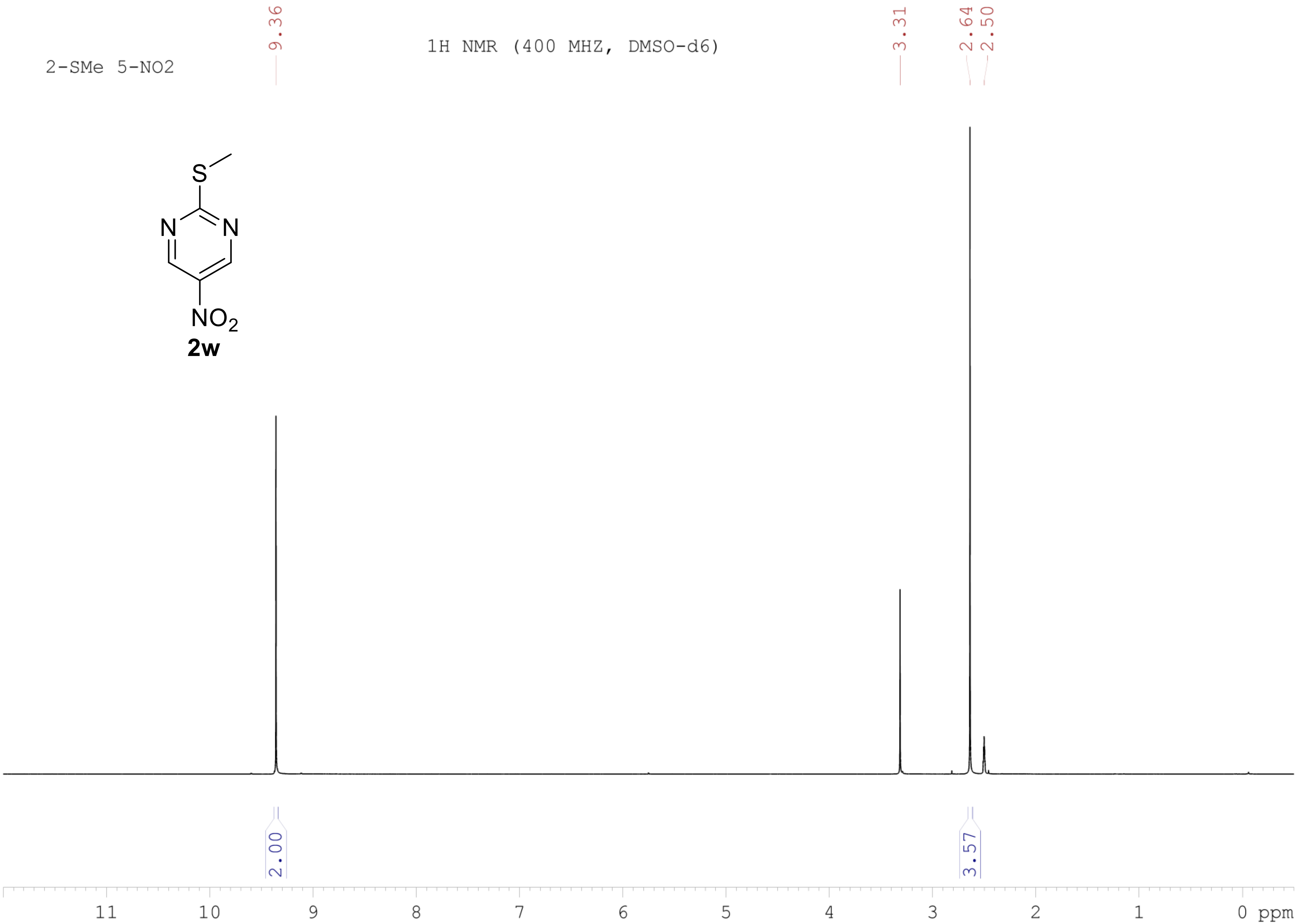
2-SMe 5-NO2
1H NMR (400 MHZ, DMSO-d6)
9.36
3.31
2.64
2.50
2w
2.00
3.57
11
10
9
8
7
6
5
4
3
2
1
0 ppm

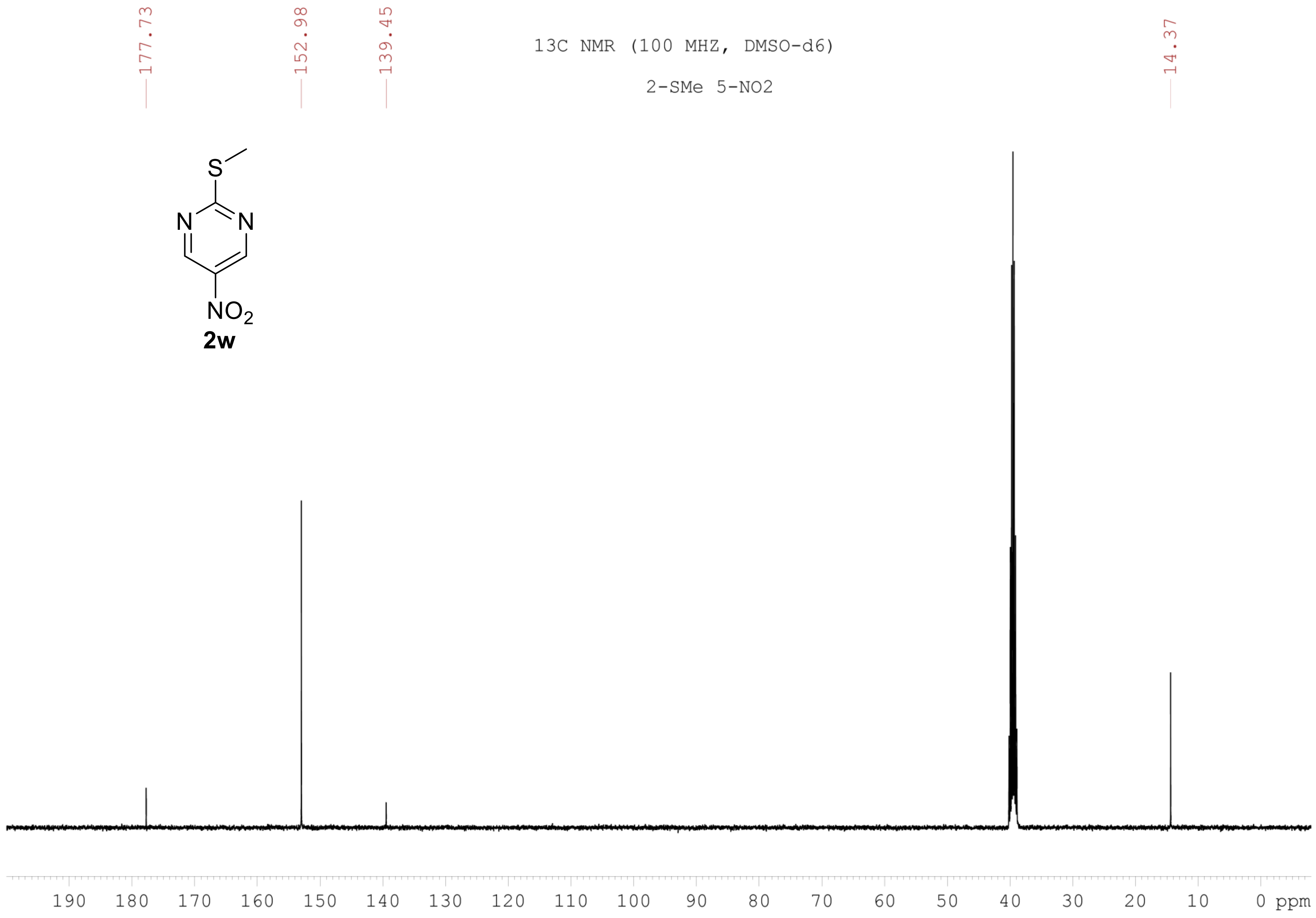

177.73
152.98
139.45
13C NMR (100 MHZ, DMSO-d6)
2-SMe 5-NO2
14.37
S
N
N
NO2
2w
190
180
170
160
150
140
130
120
110
100
90
80
70
60
50
40
30
20
10
0 ppm

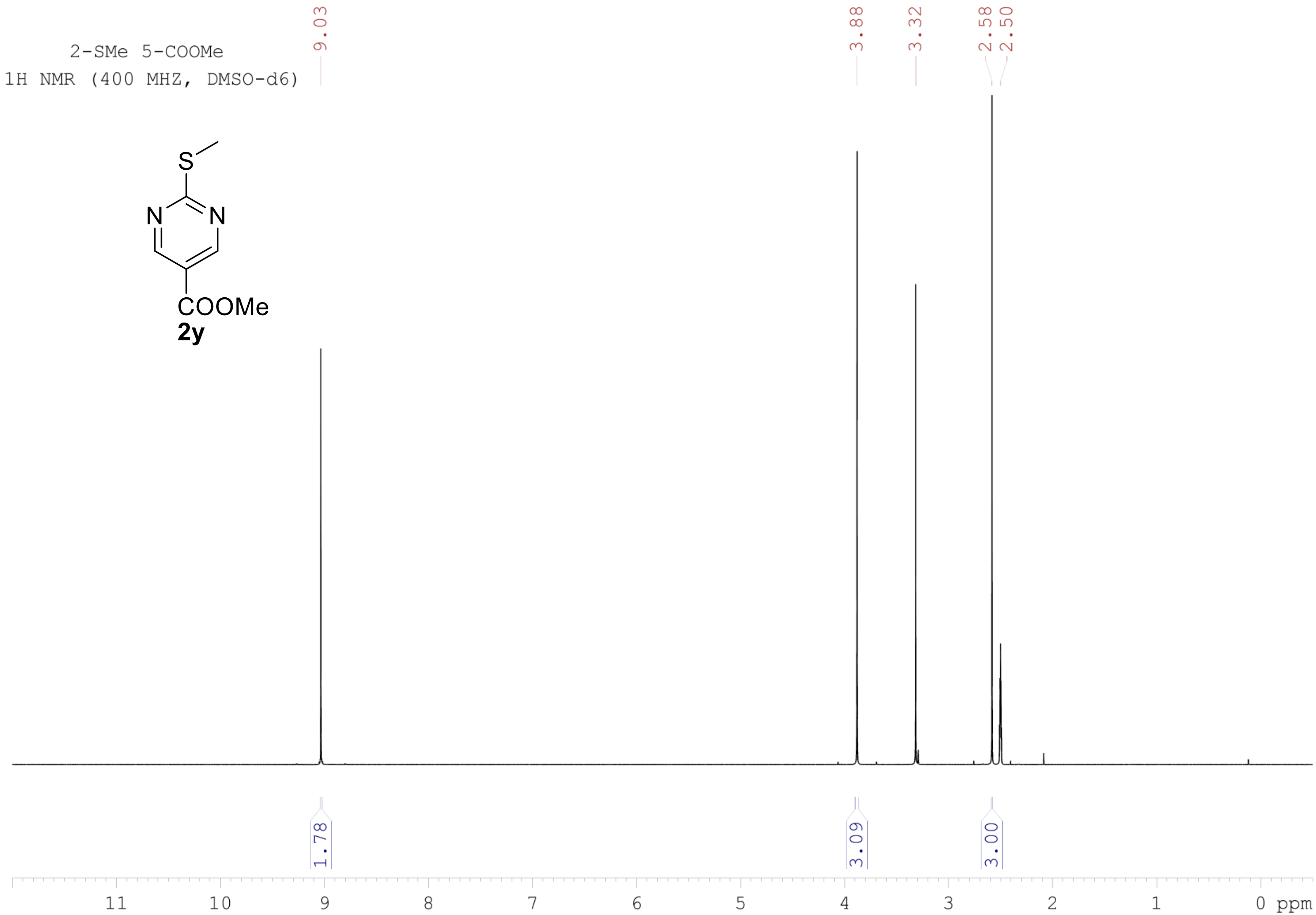
2-SMe 5-COOMe
1H NMR (400 MHZ, DMSO-d6)
9.03
3.88
3.32
2.58
2.50
S
N
N
COOMe
2y
1.78
3.09
3.00
11
10
9
8
7
6
5
4
3
2
1
0 ppm

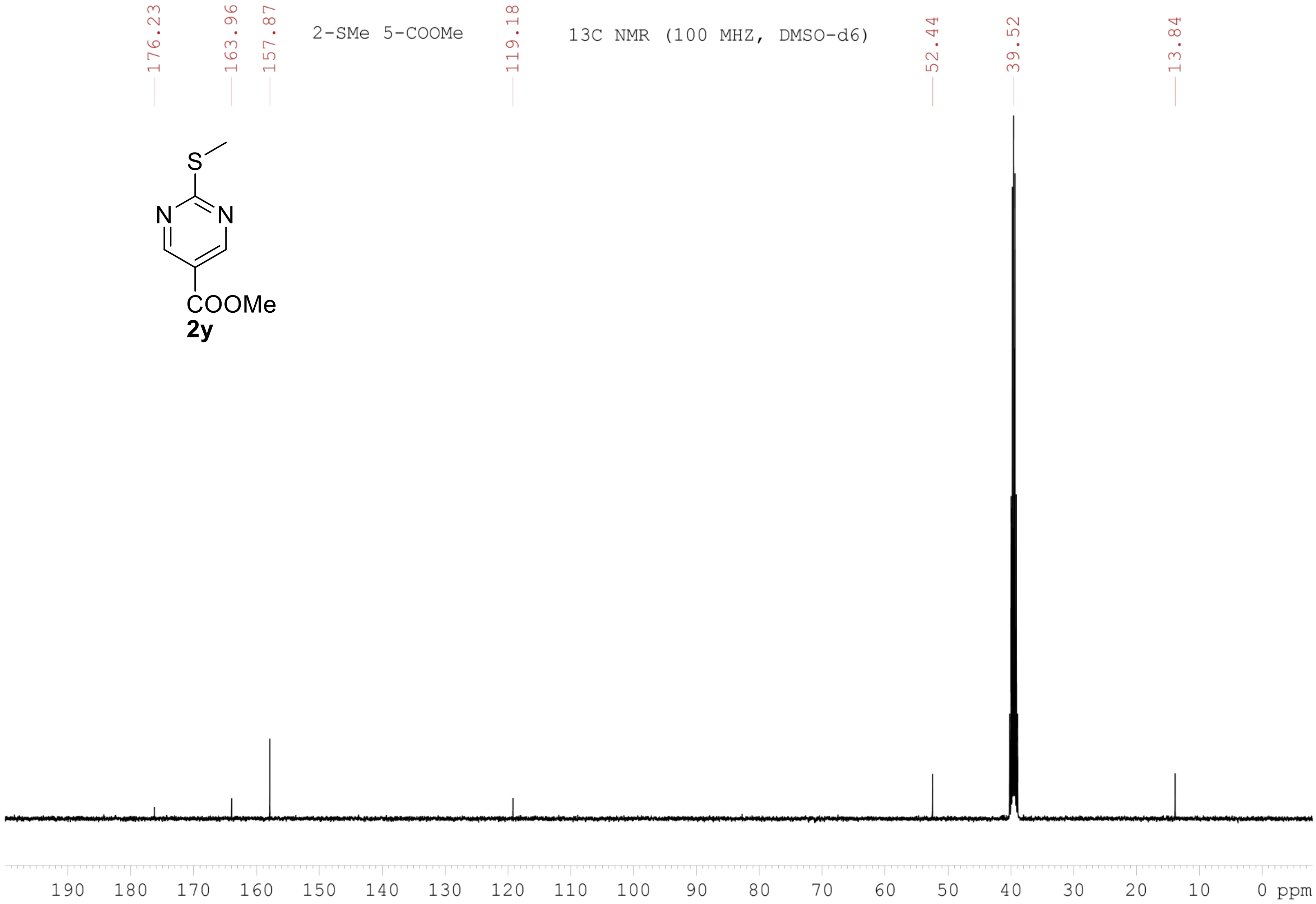
176.23
163.96
157.87
2-SMe 5-COOMe
119.18
13C NMR (100 MHZ, DMSO-d6)
52.44
39.52
13.84
S
N
N
COOMe
2y
190 180 170 160 150 140 130 120 110 100 90 80 70 60 50 40 30 20 10 0 ppm

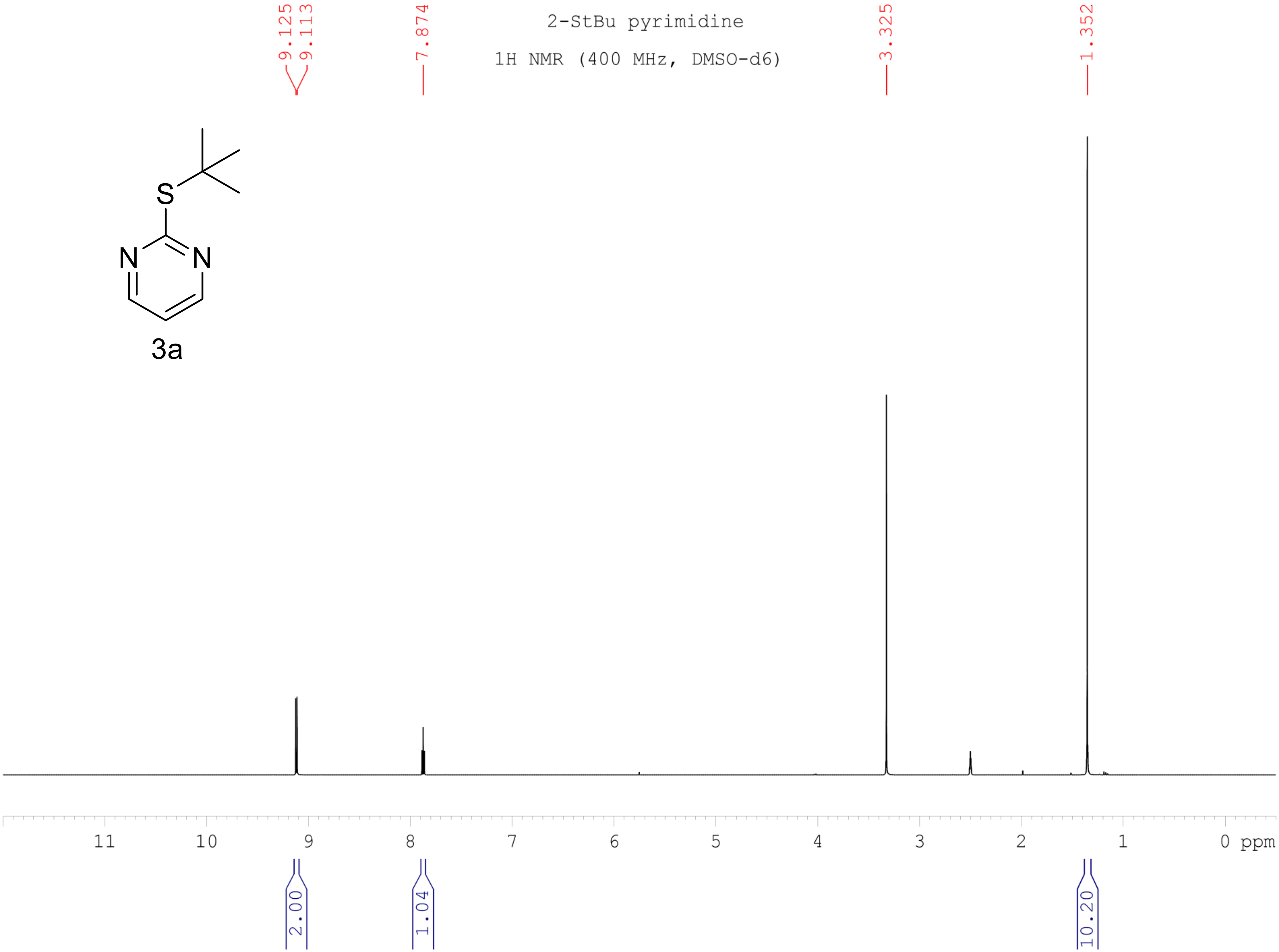
2-StBu pyrimidine
1H NMR (400 MHz, DMSO-d6)
9.125
9.113
7.874
3.325
1.352
S
N
N
3a
11 10 9 8 7 6 5 4 3 2 1 0 ppm
2.00
1.04
10.20

2-SnBu pyrimidine

1H NMR (400 MHZ, DMSO-d6)

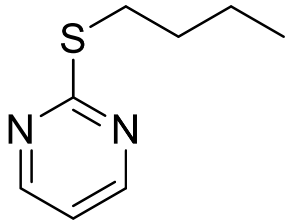

3c

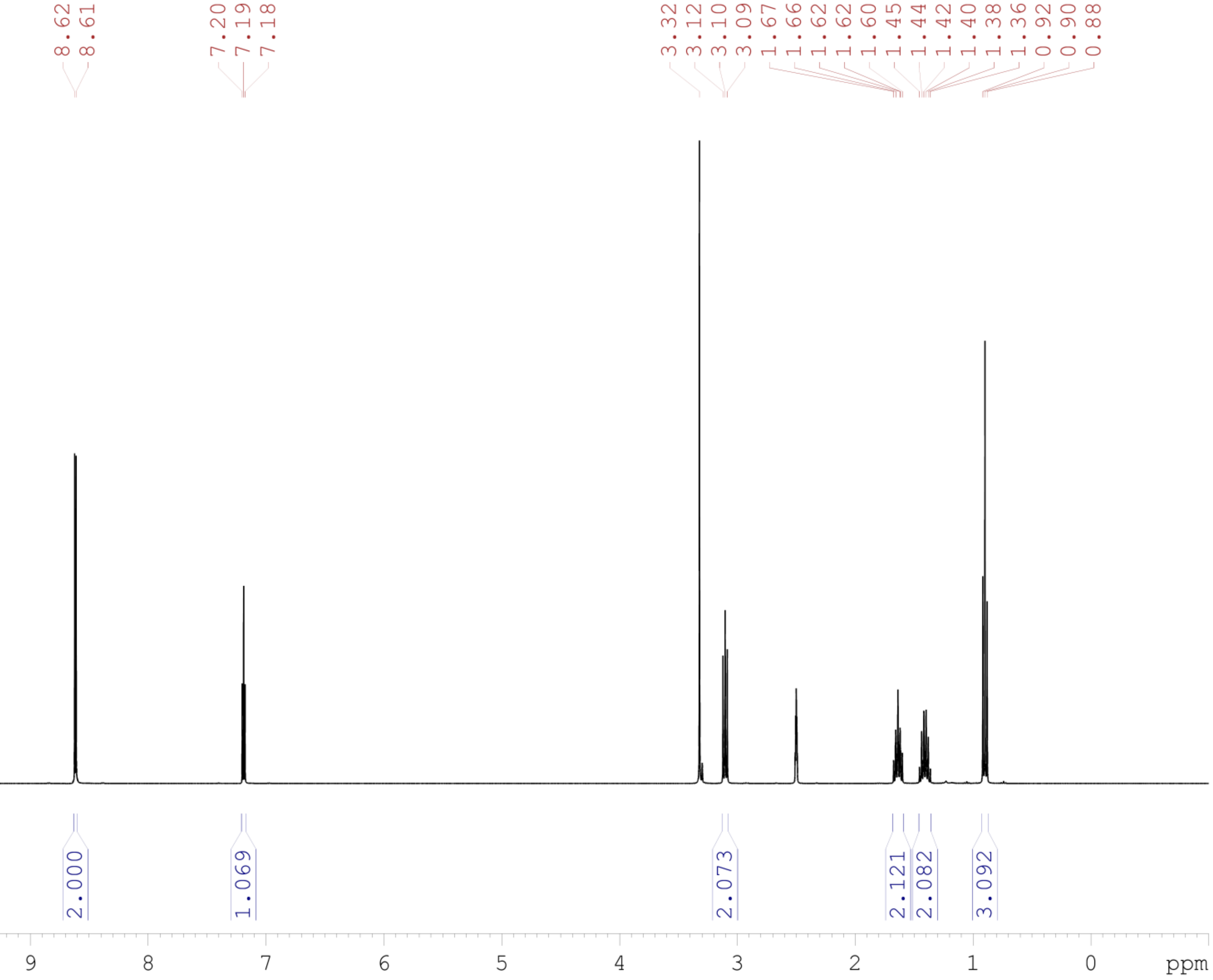

2-SnBu pyrimidine

13C NMR (100 MHZ, DMSO-d6)

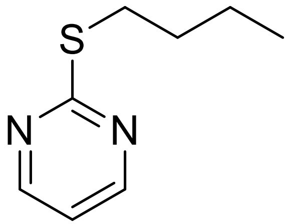

3c

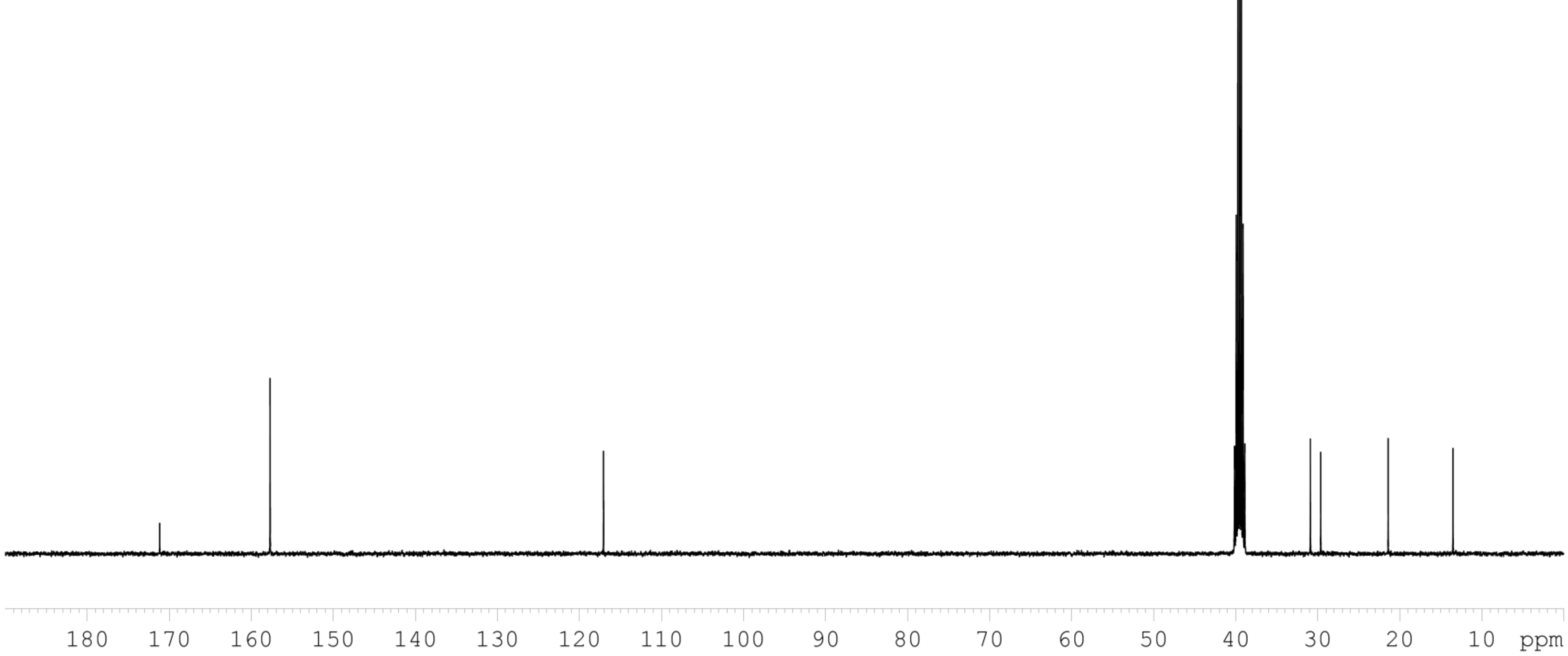

2-SPh pyrimidine
1H NMR (400 MHZ, DMSO-d6)

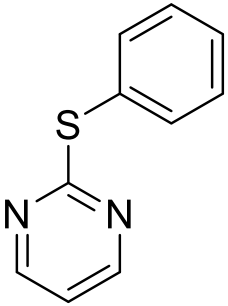

3d

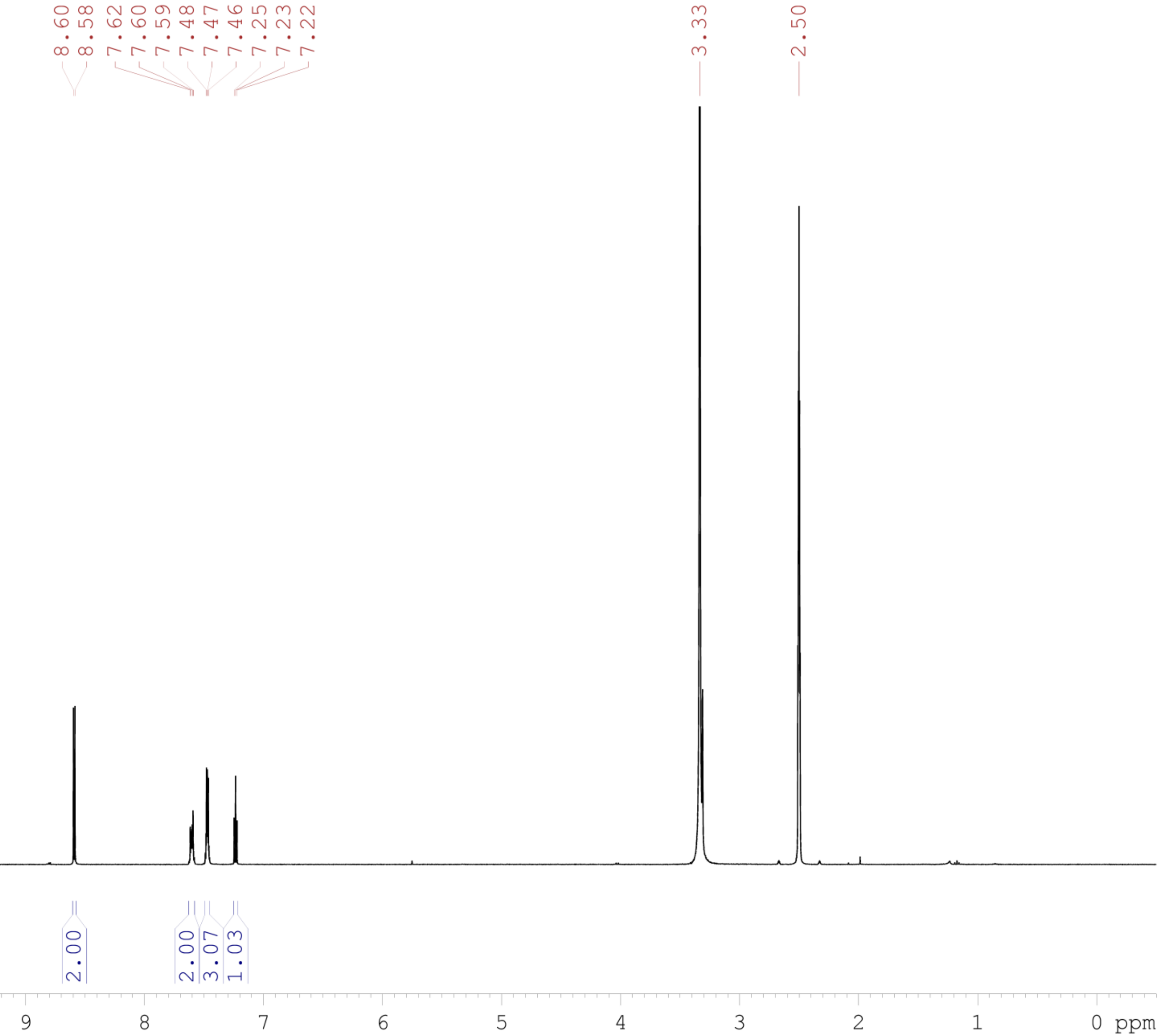

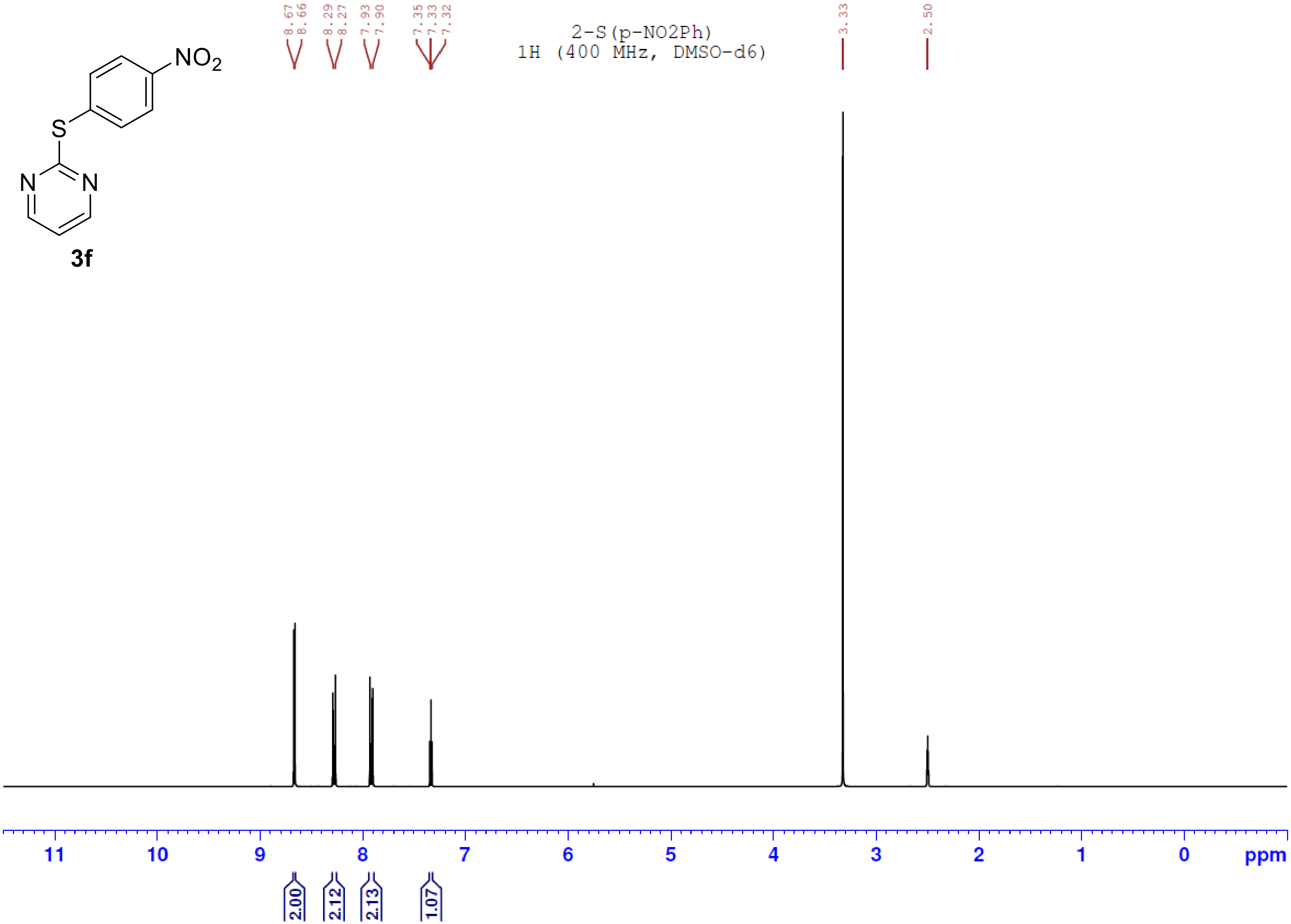

NO2
S
N
N
3f
8.67
8.66
8.29
8.27
7.93
7.90
7.35
7.33
7.32
2-S(p-NO2Ph)
1H (400 MHz, DMSO-d6)
3.33
2.50
11
10
9
8
7
6
5
4
3
2
1
0
ppm
2.00
2.12
2.13
1.07

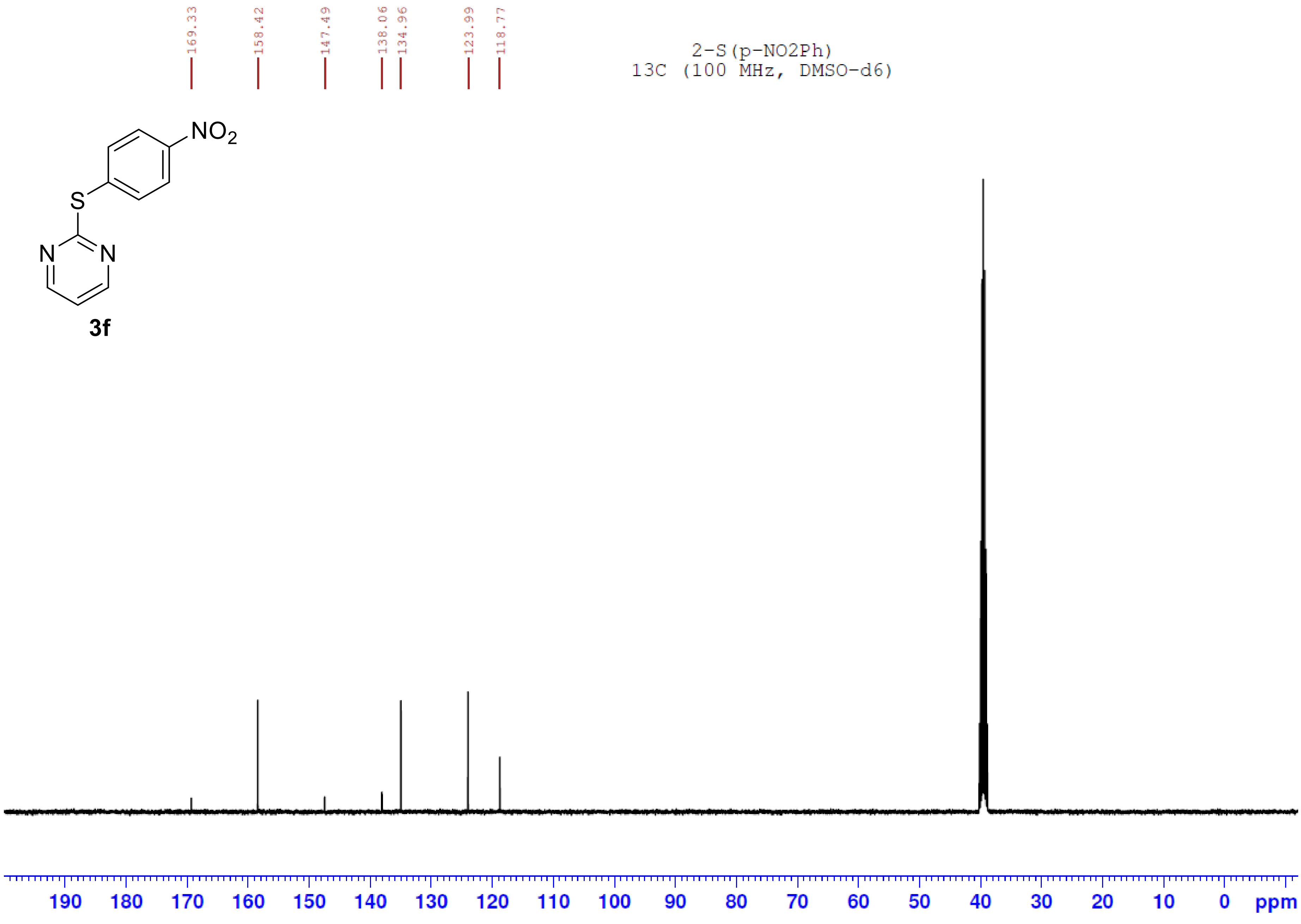
169.33
158.42
147.49
138.06
134.96
123.99
118.77
2-S(p-NO2Ph)
13C (100 MHz, DMSO-d6)
NO2
S
N
N
3f
190 180 170 160 150 140 130 120 110 100 90 80 70 60 50 40 30 20 10 0 ppm

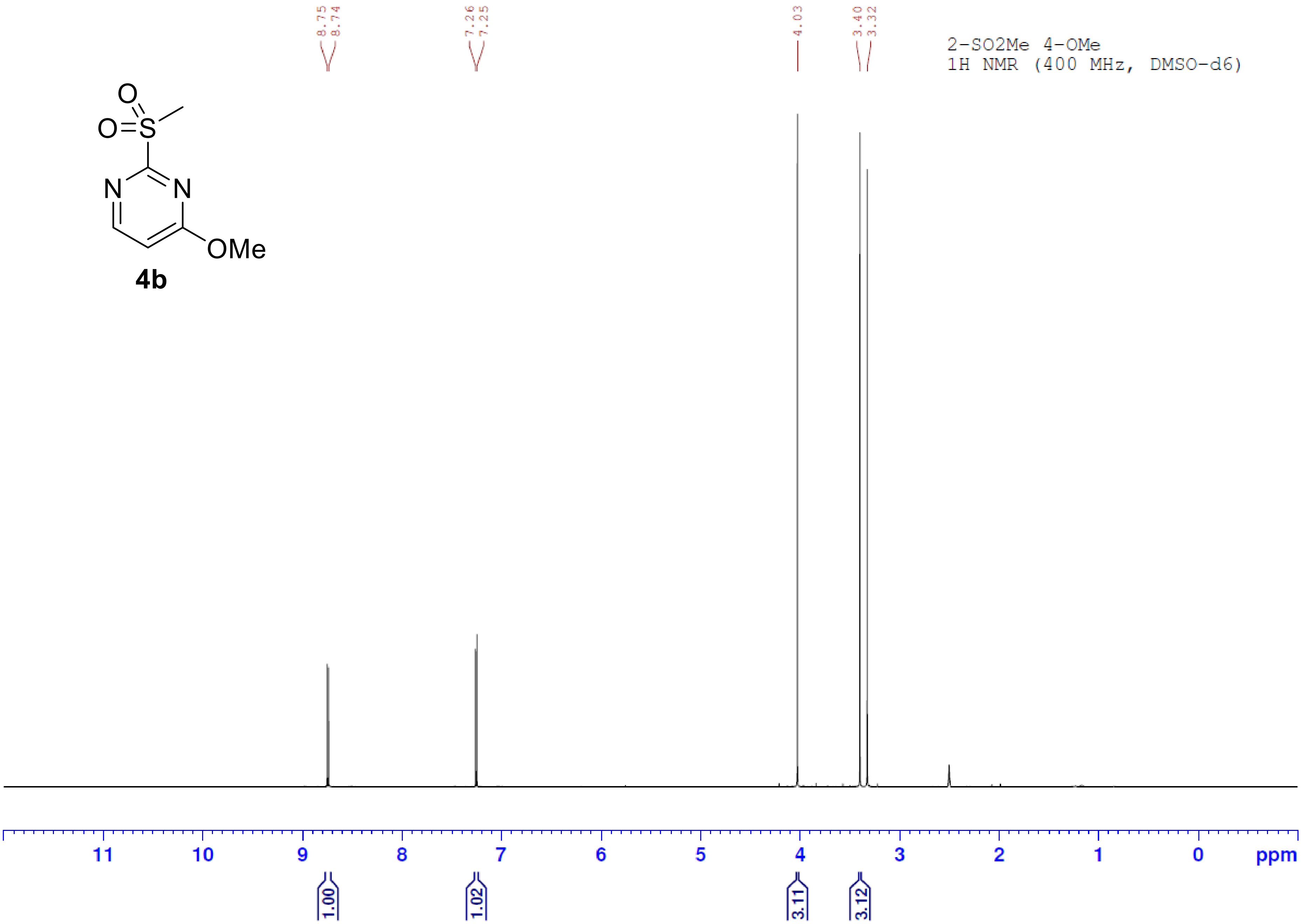
8.75
8.74
7.26
7.25
4.03
3.40
3.32
2-SO2Me 4-OMe
1H NMR (400 MHz, DMSO-d6)
OMe
11
10
9
8
7
6
5
4
3
2
1
0
ppm
1.00
1.02
3.11
3.12

4b

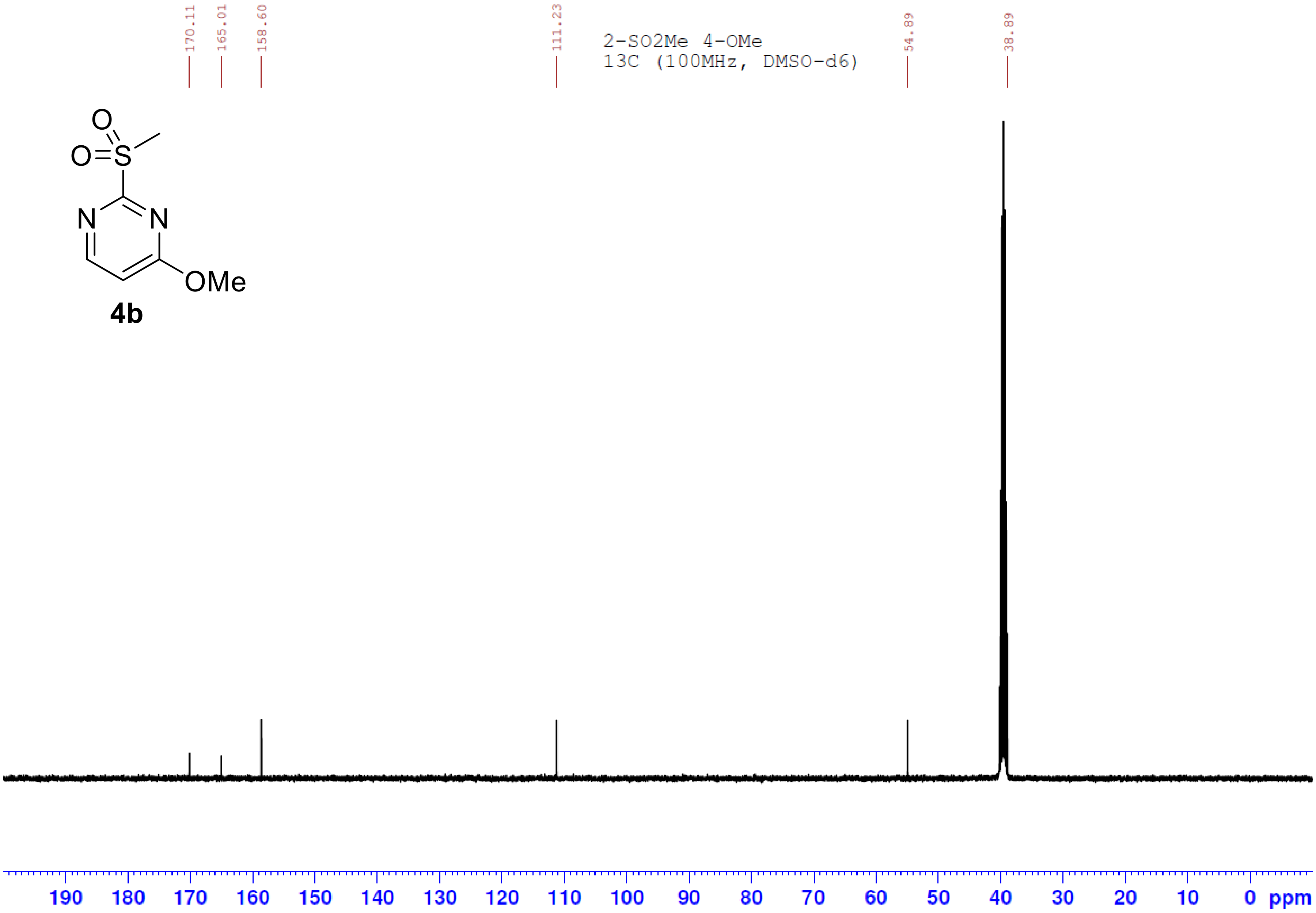
170.11
165.01
158.60
111.23
2-SO2Me 4-OMe
13C (100MHz, DMSO-d6)
54.89
38.89
O
O=S
N
N
OMe
4b
190 180 170 160 150 140 130 120 110 100 90 80 70 60 50 40 30 20 10 0 ppm

2-SO2Me 4-Me pyrimidine

1H NMR (400 MHz, DMSO-d6)

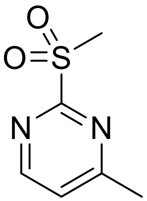

4c

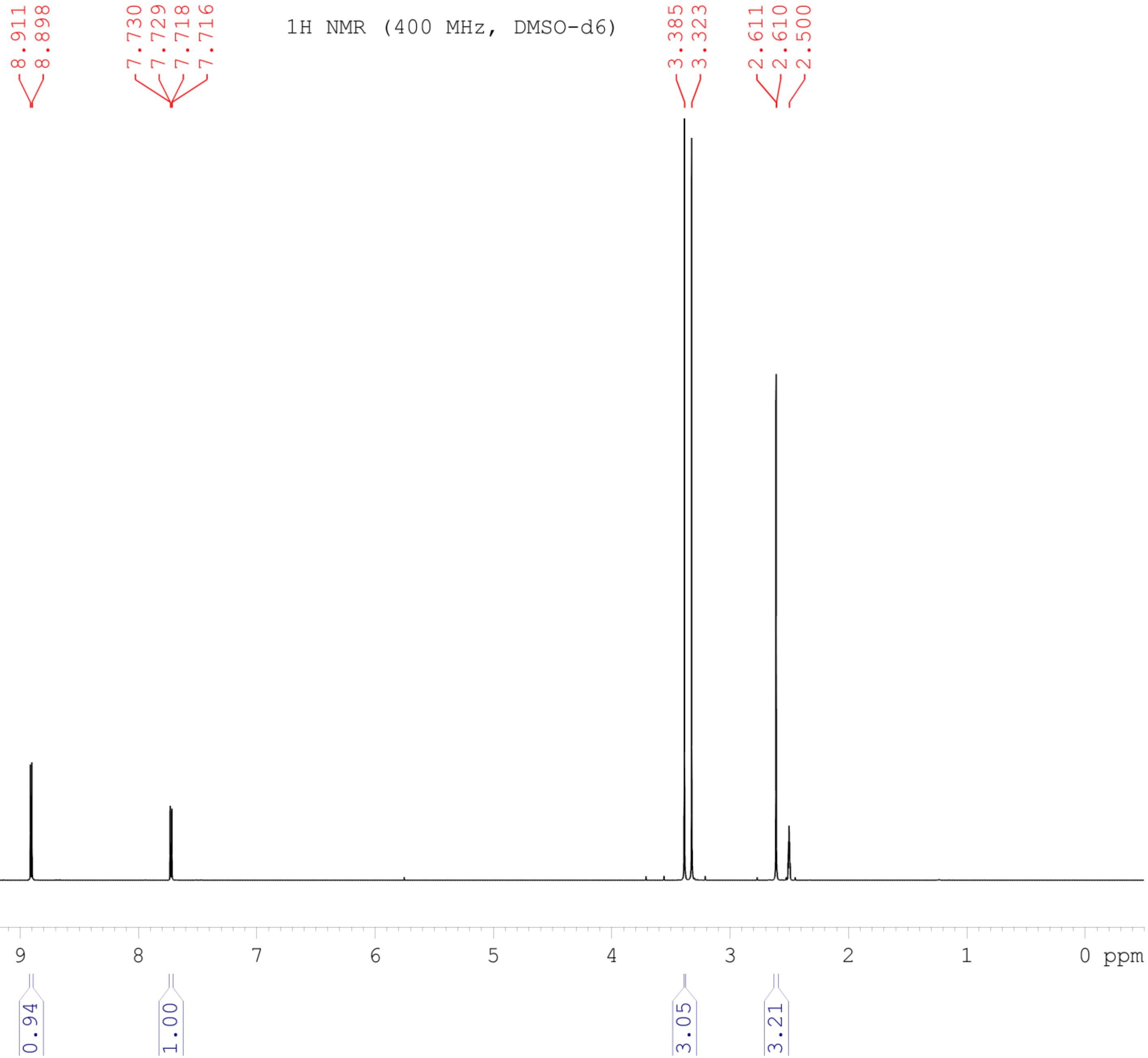

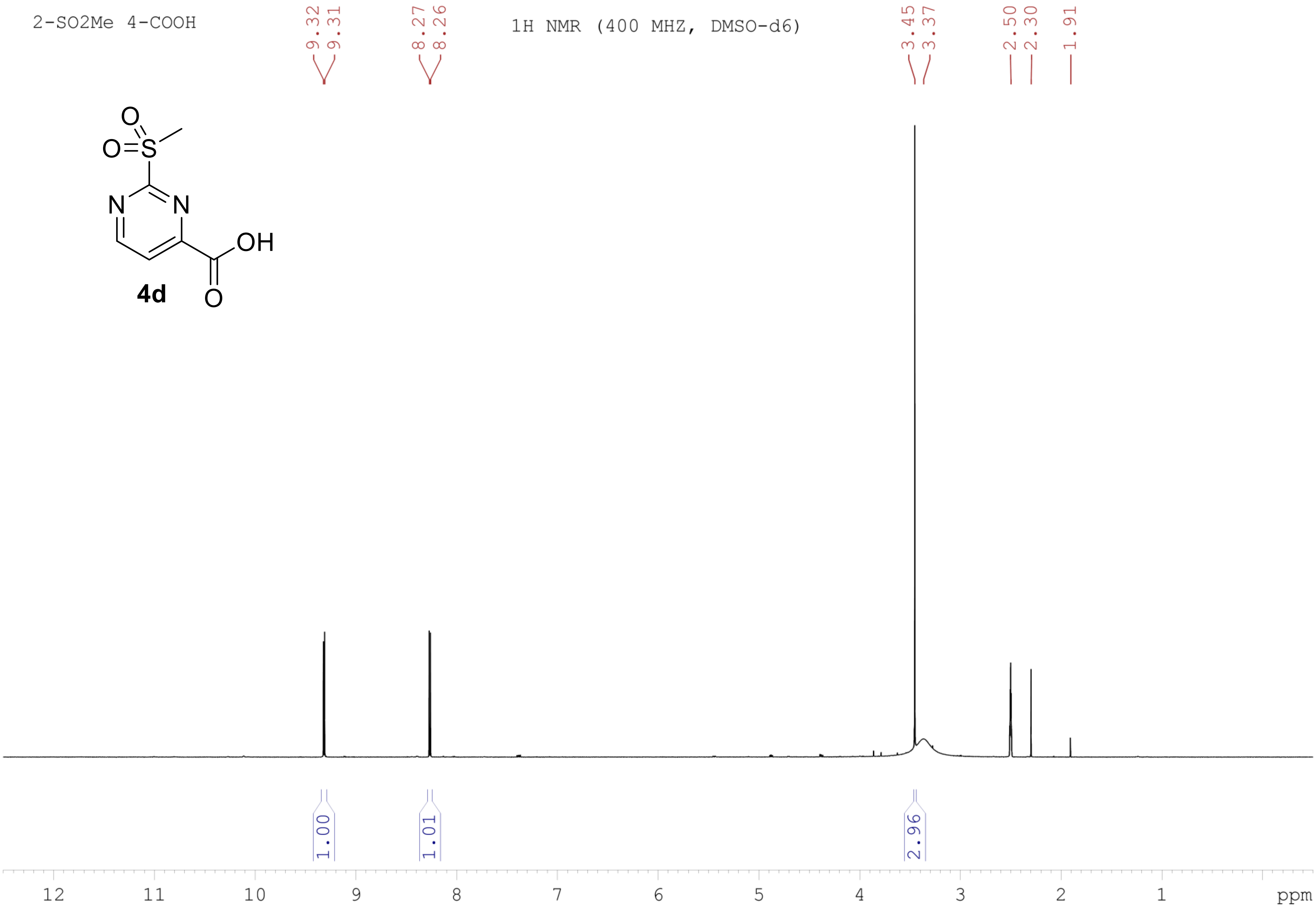
2-SO2Me 4-COOH
1H NMR (400 MHZ, DMSO-d6)
9.32
9.31
8.27
8.26
3.45
3.37
2.50
2.30
1.91
4d
1.00
1.01
2.96
12
11
10
9
8
7
6
5
4
3
2
1
ppm

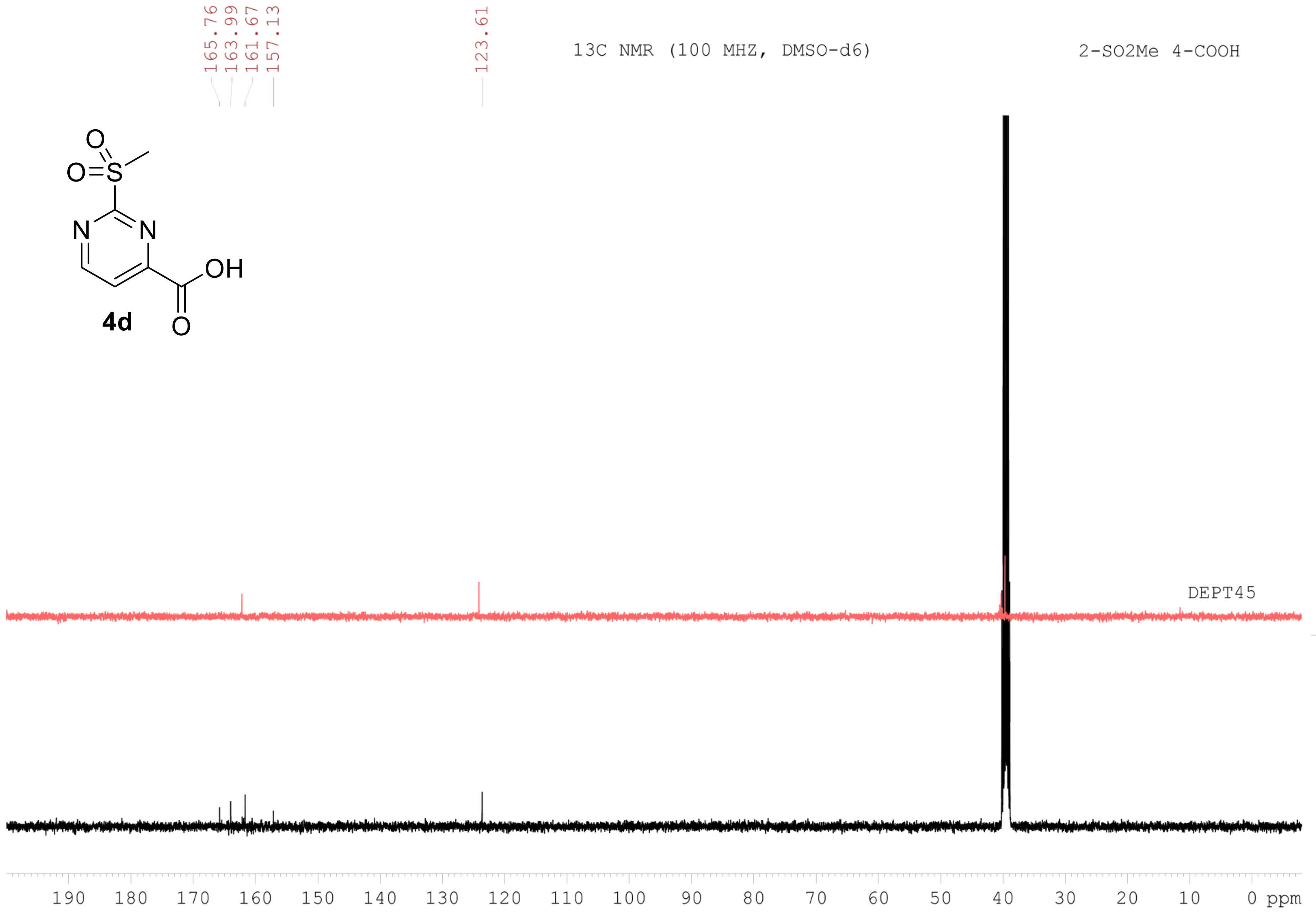

165.76
163.99
161.67
157.13
123.61
13C NMR (100 MHZ, DMSO-d6)
2-SO2Me 4-COOH
DEPT45
190 180 170 160 150 140 130 120 110 100 90 80 70 60 50 40 30 20 10 0 ppm

4d

1H NMR (400 MHZ, DMSO-d6)

2-SO2Me 4-Ph

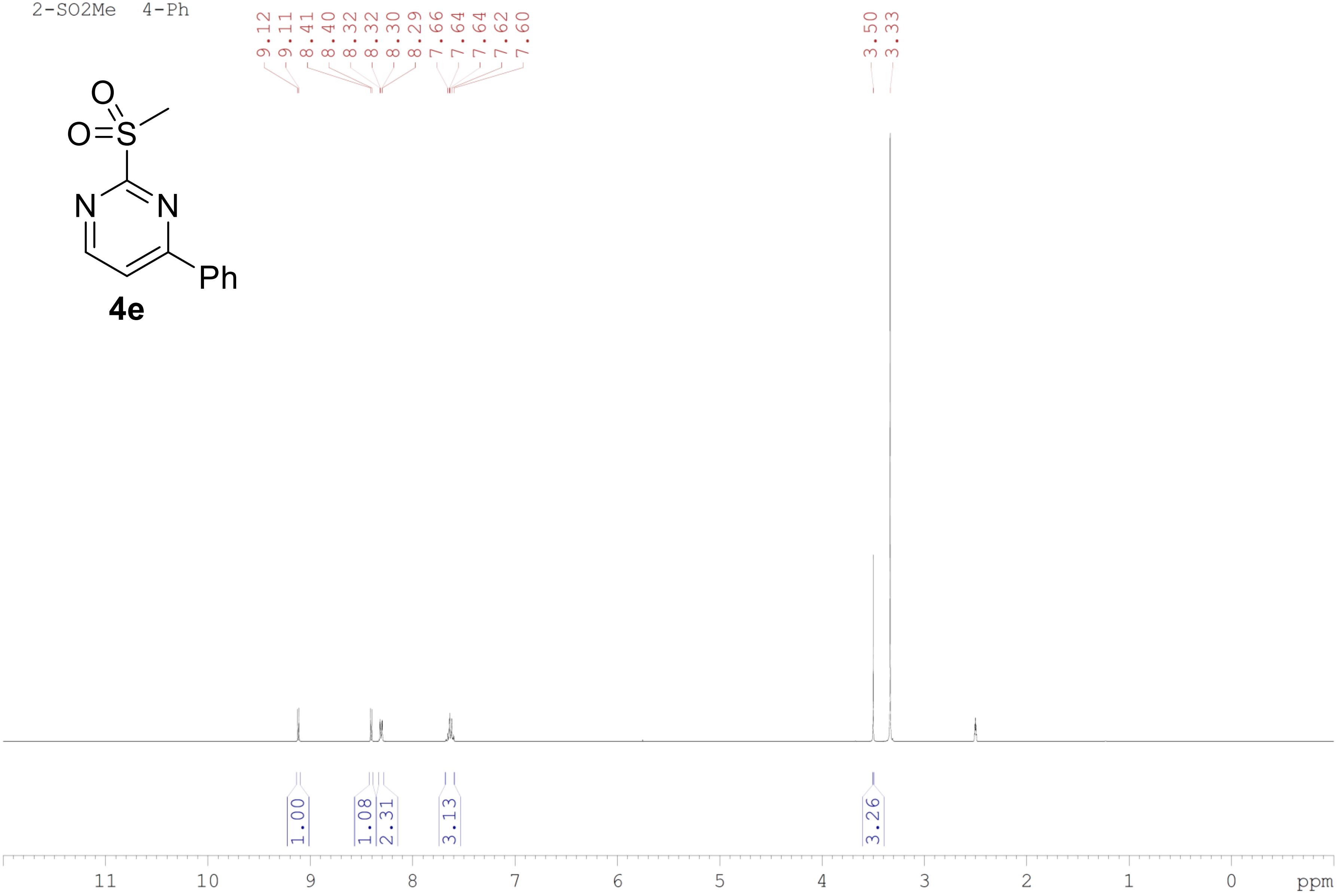

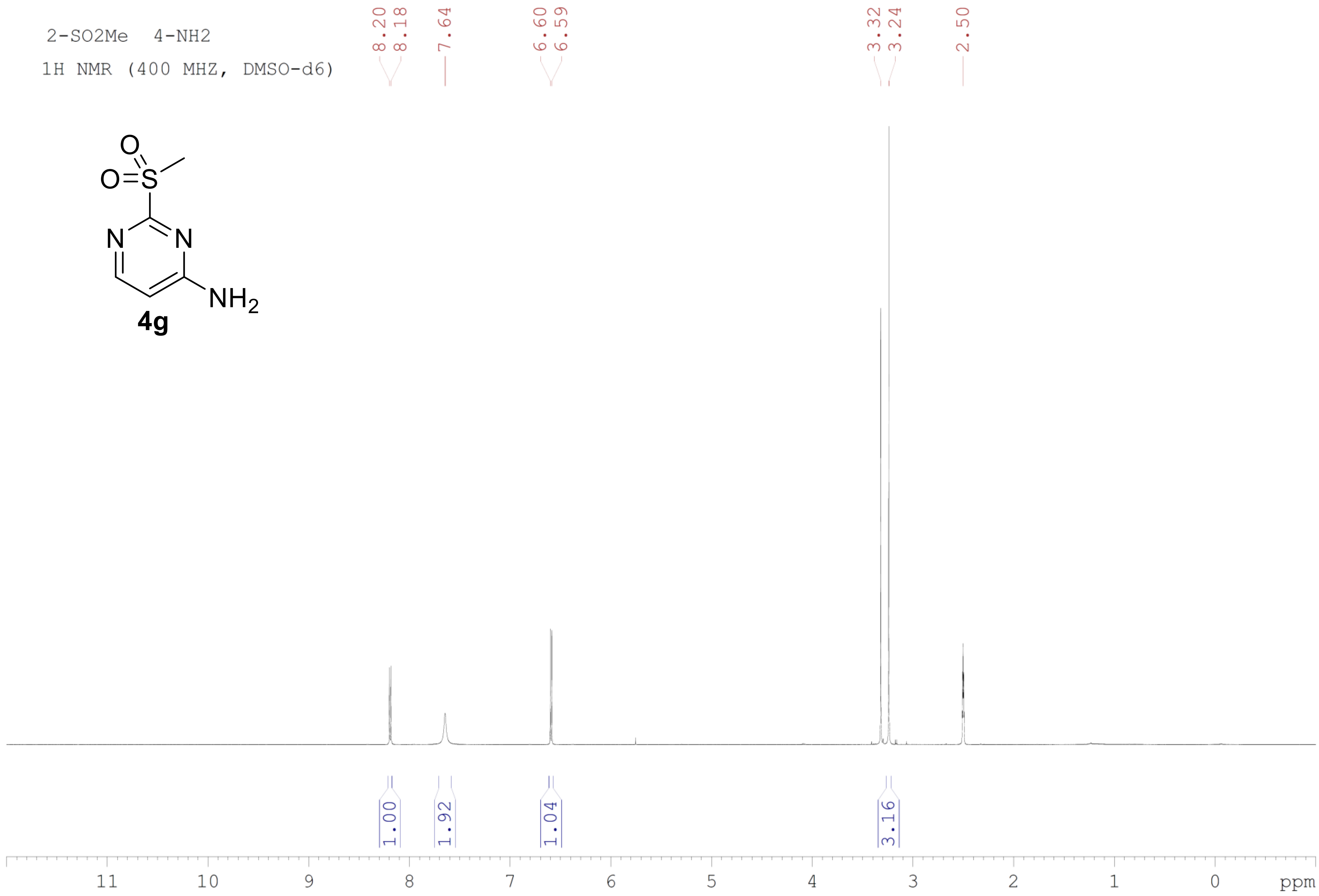
2-SO2Me 4-NH2
1H NMR (400 MHZ, DMSO-d6)
8.20
8.18
7.64
6.60
6.59
3.32
3.24
2.50
O
O=S
N
N
NH2
4g
1.00
1.92
1.04
3.16
11
10
9
8
7
6
5
4
3
2
1
0
ppm

2-SO2Me 4-NH2

13C NMR (100 MHZ, DMSO-d6)

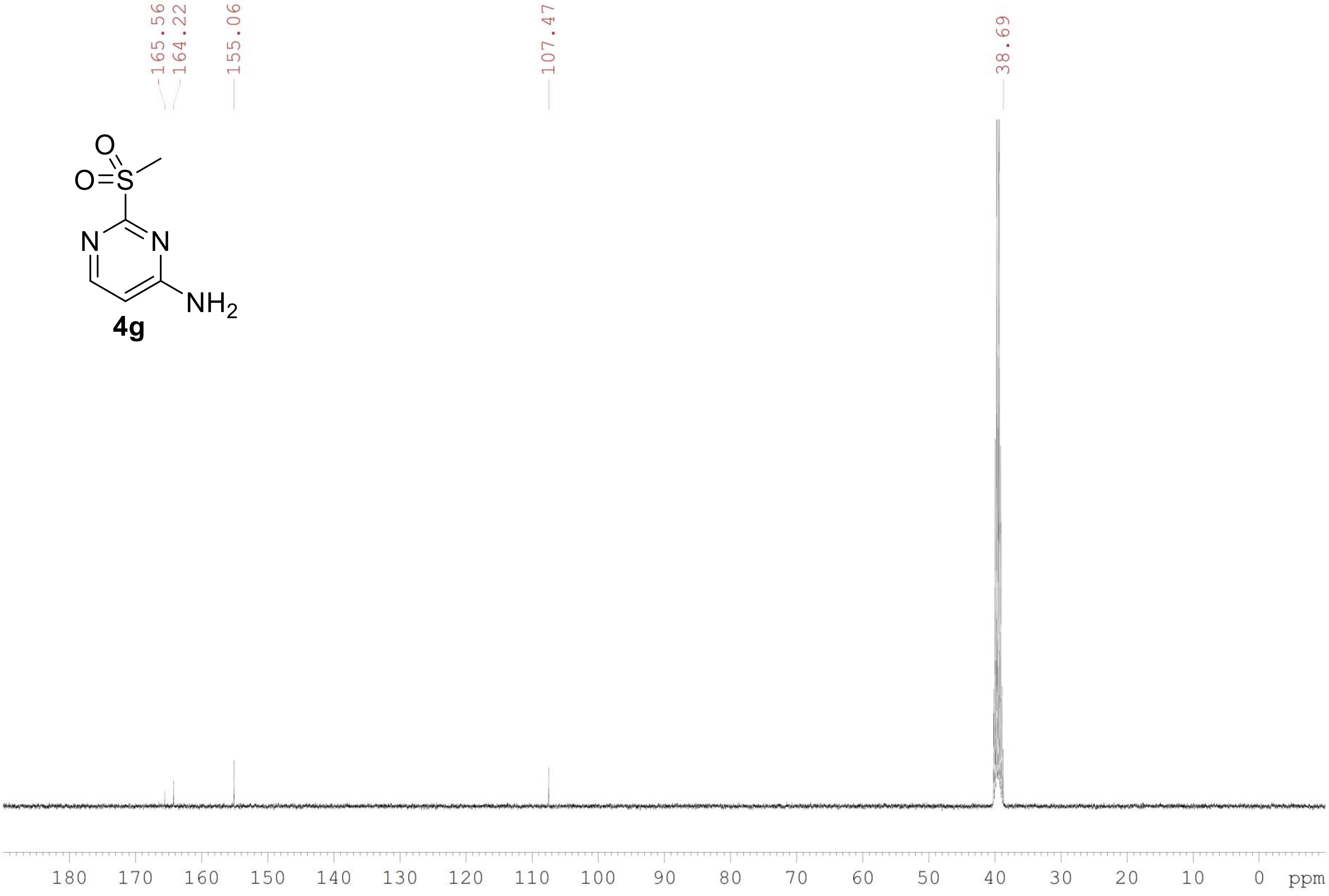

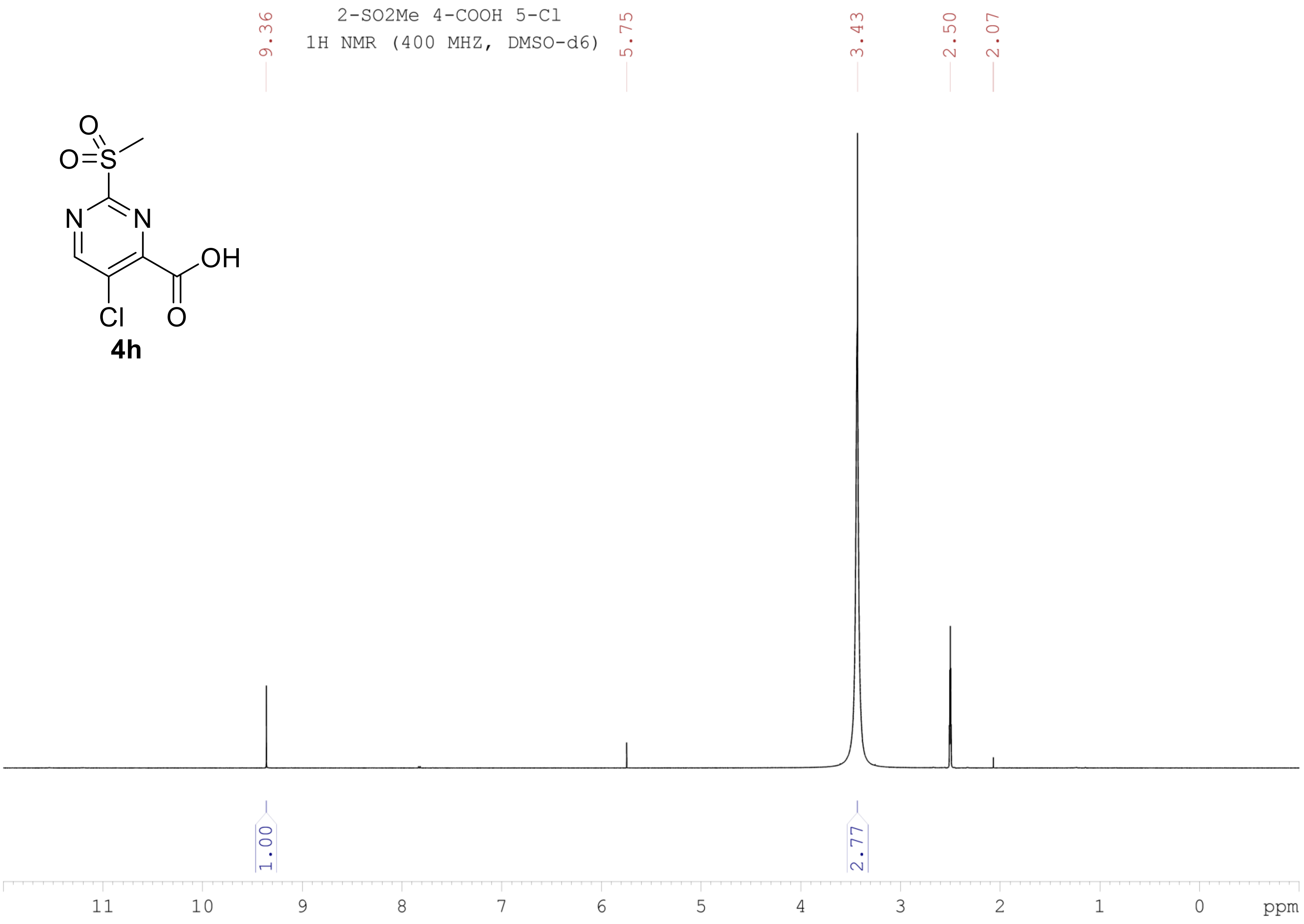
2-SO2Me 4-COOH 5-Cl
1H NMR (400 MHZ, DMSO-d6)
9.36
5.75
3.43
2.50
2.07
4h
1.00
2.77
11
10
9
8
7
6
5
4
3
2
1
0
ppm

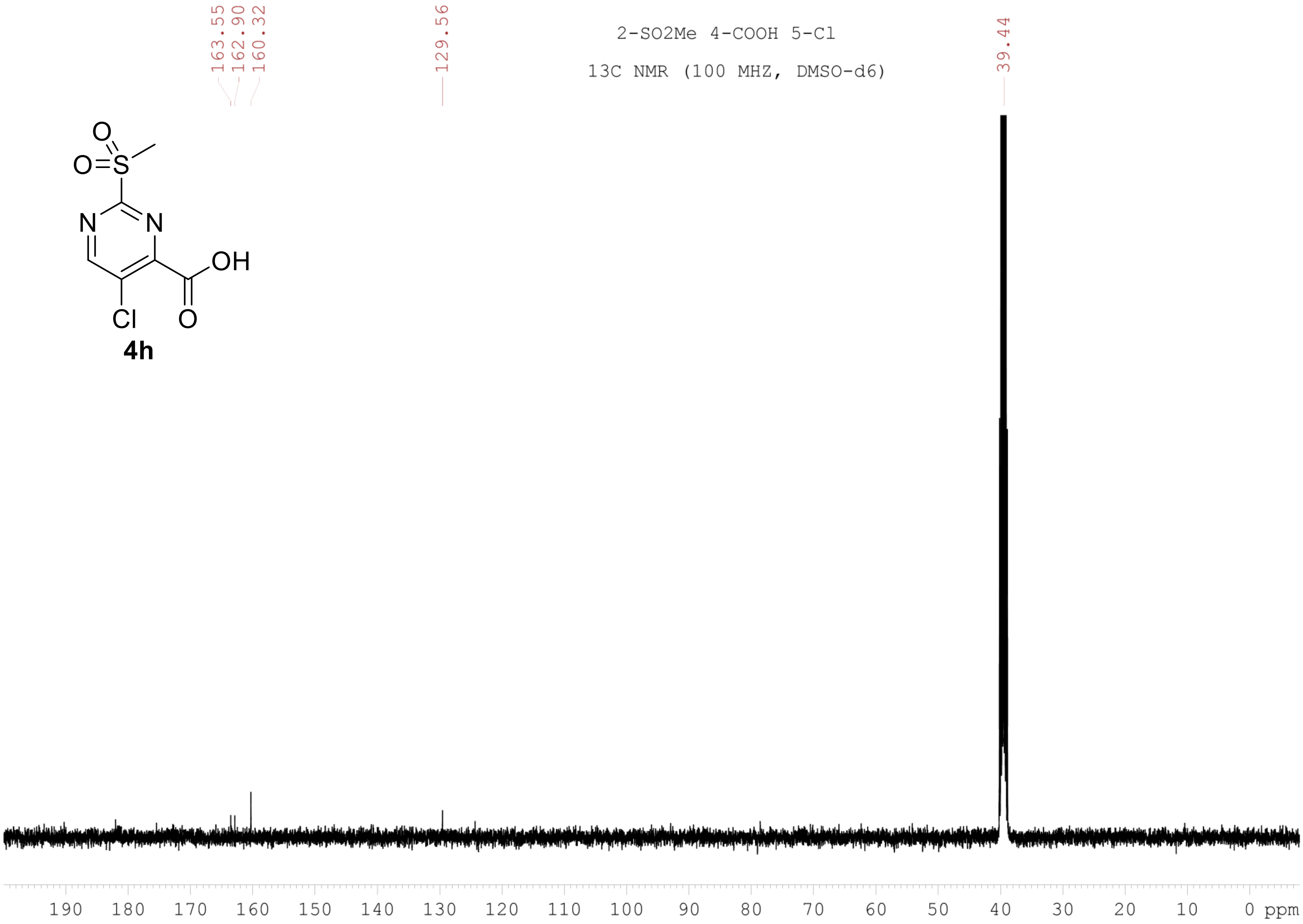

2-SO2Me 4-COOH 5-Cl
13C NMR (100 MHZ, DMSO-d6)
163.55
162.90
160.32
129.56
39.44
4h
190 180 170 160 150 140 130 120 110 100 90 80 70 60 50 40 30 20 10 0 ppm

1H NMR (400 MHZ, DMSO-d6)

2-SO2Me 4-C(O)NH2

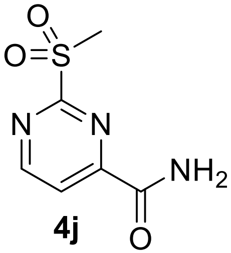

4j

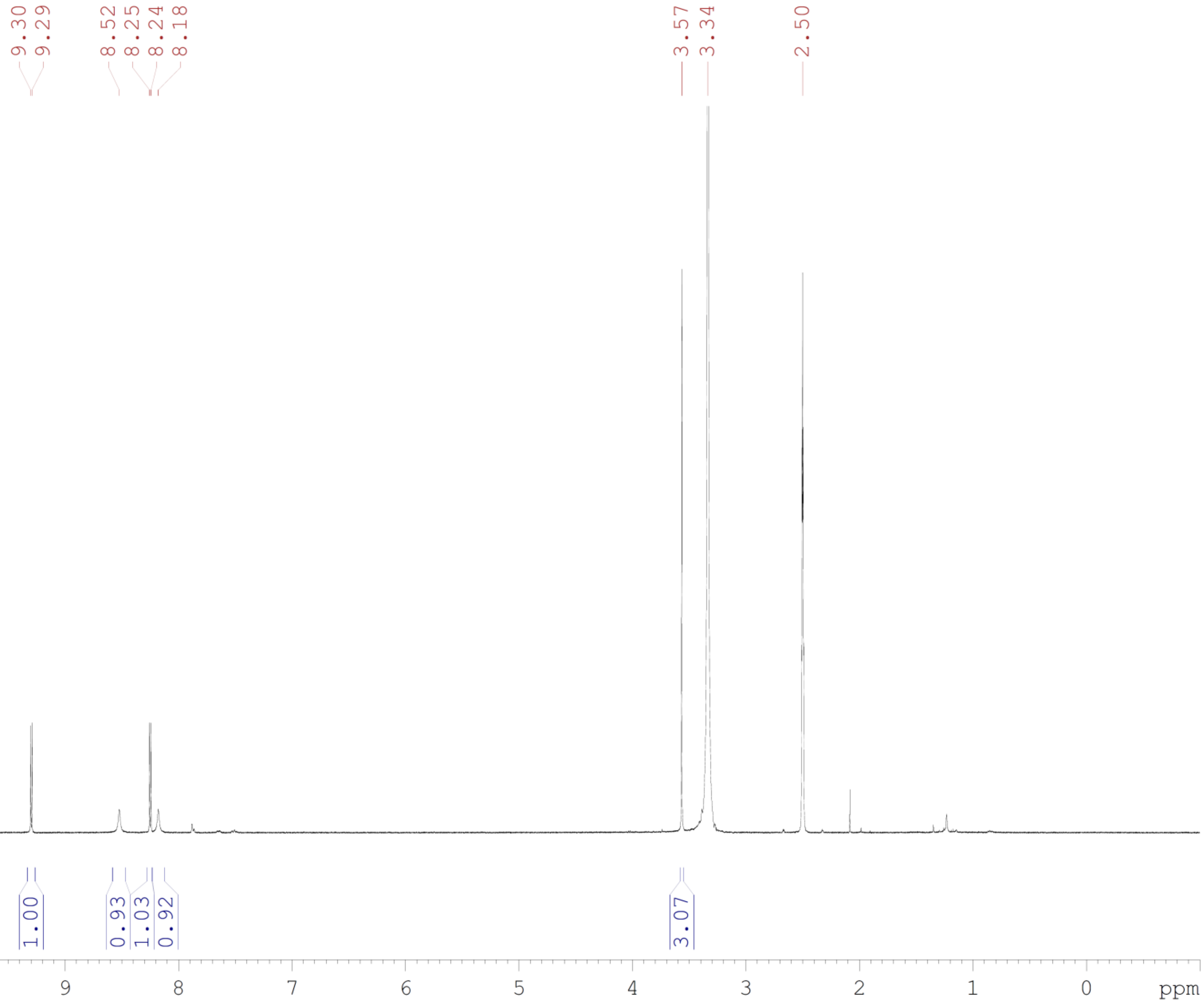

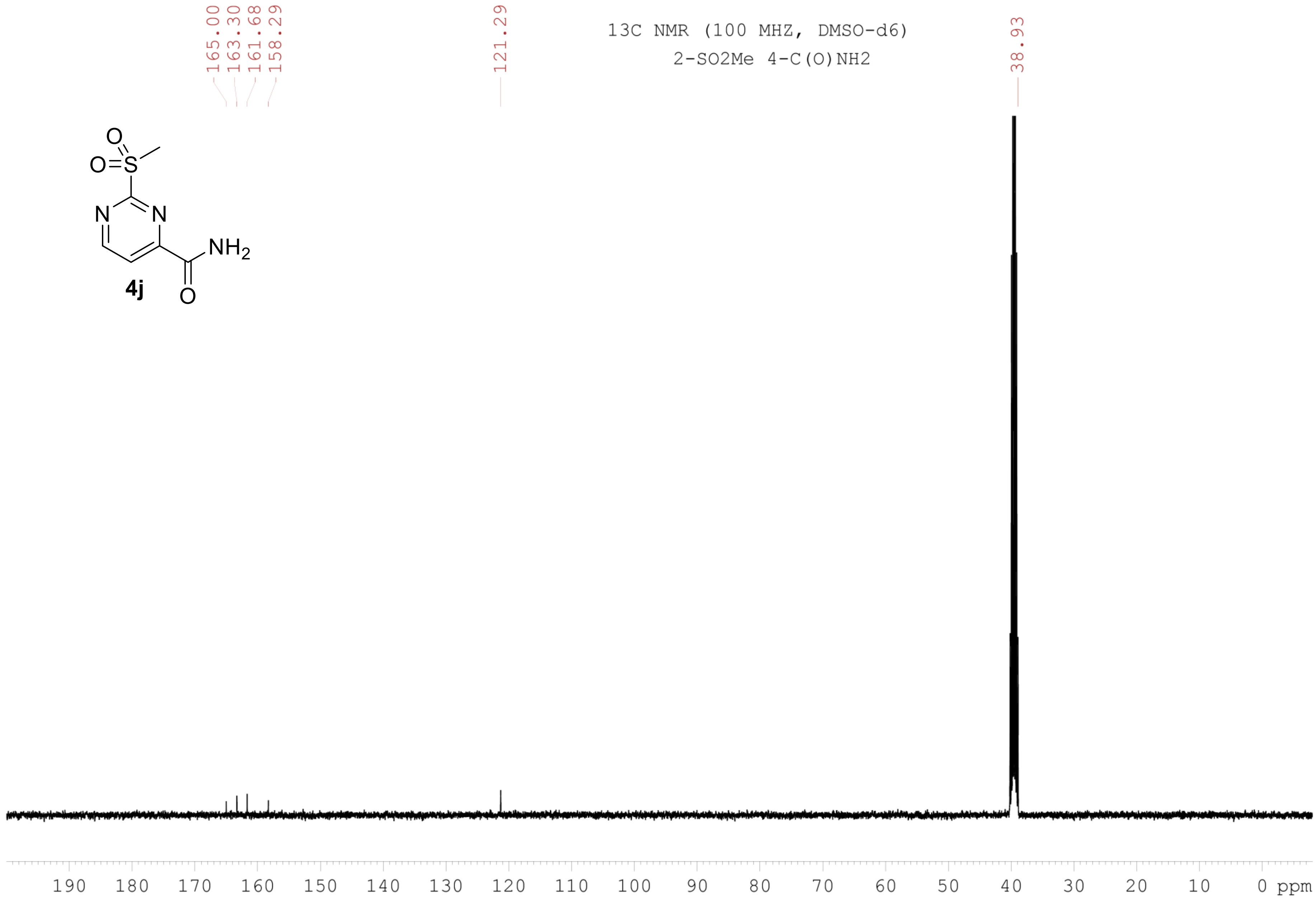
13C NMR (100 MHZ, DMSO-d6)
2-SO2Me 4-C(O)NH2
165.00
163.30
161.68
158.29
121.29
38.93
4j
190 180 170 160 150 140 130 120 110 100 90 80 70 60 50 40 30 20 10 0 ppm

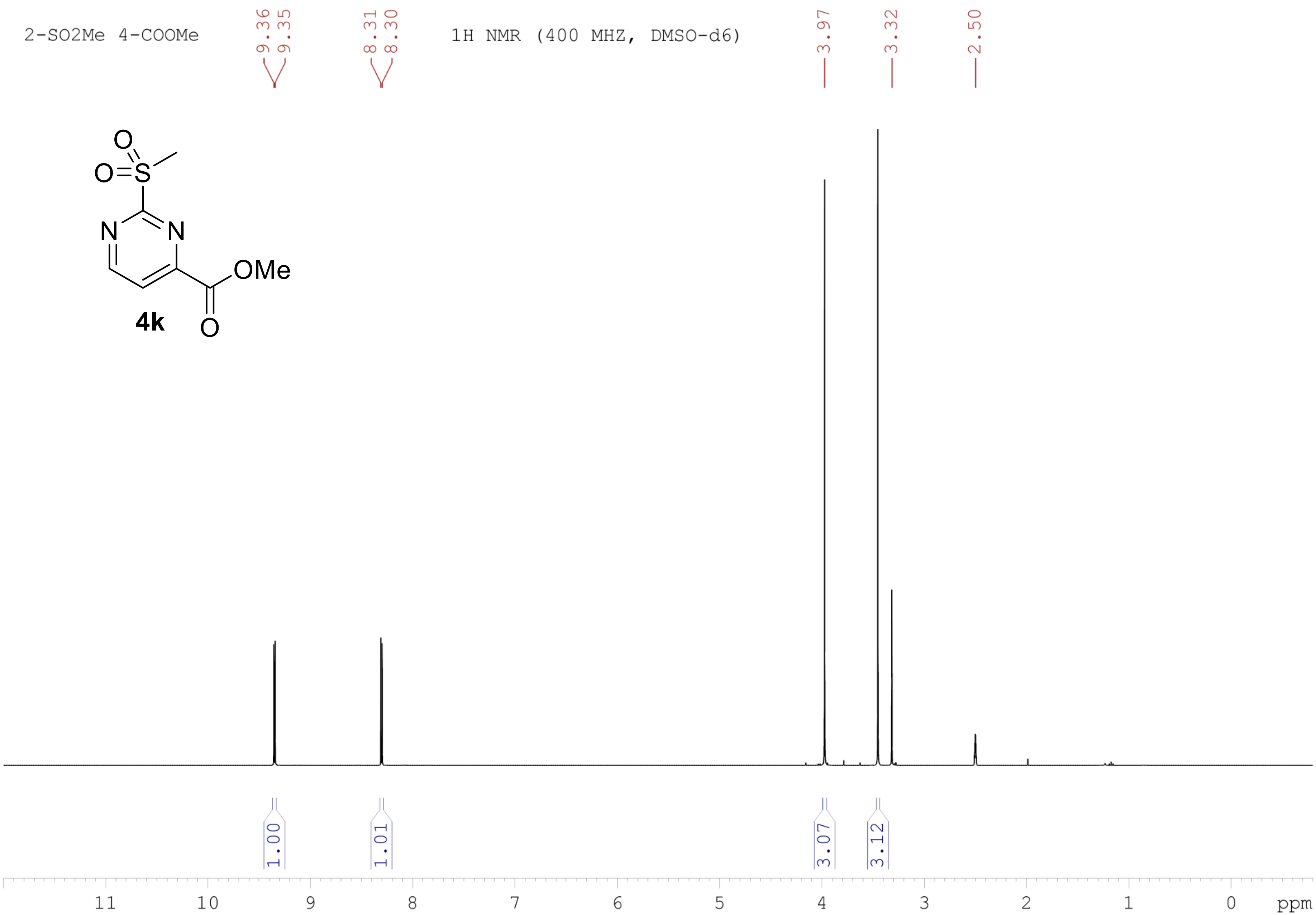
2-SO2Me 4-COOMe
1H NMR (400 MHZ, DMSO-d6)
9.36
9.35
8.31
8.30
3.97
3.32
2.50
1.00
1.01
3.07
3.12
11
10
9
8
7
6
5
4
3
2
1
0
ppm
OMe
N
N
O
S
O
O
4k

2-SO2Me 4-COOMe

13C NMR (100 MHZ, DMSO-d6)

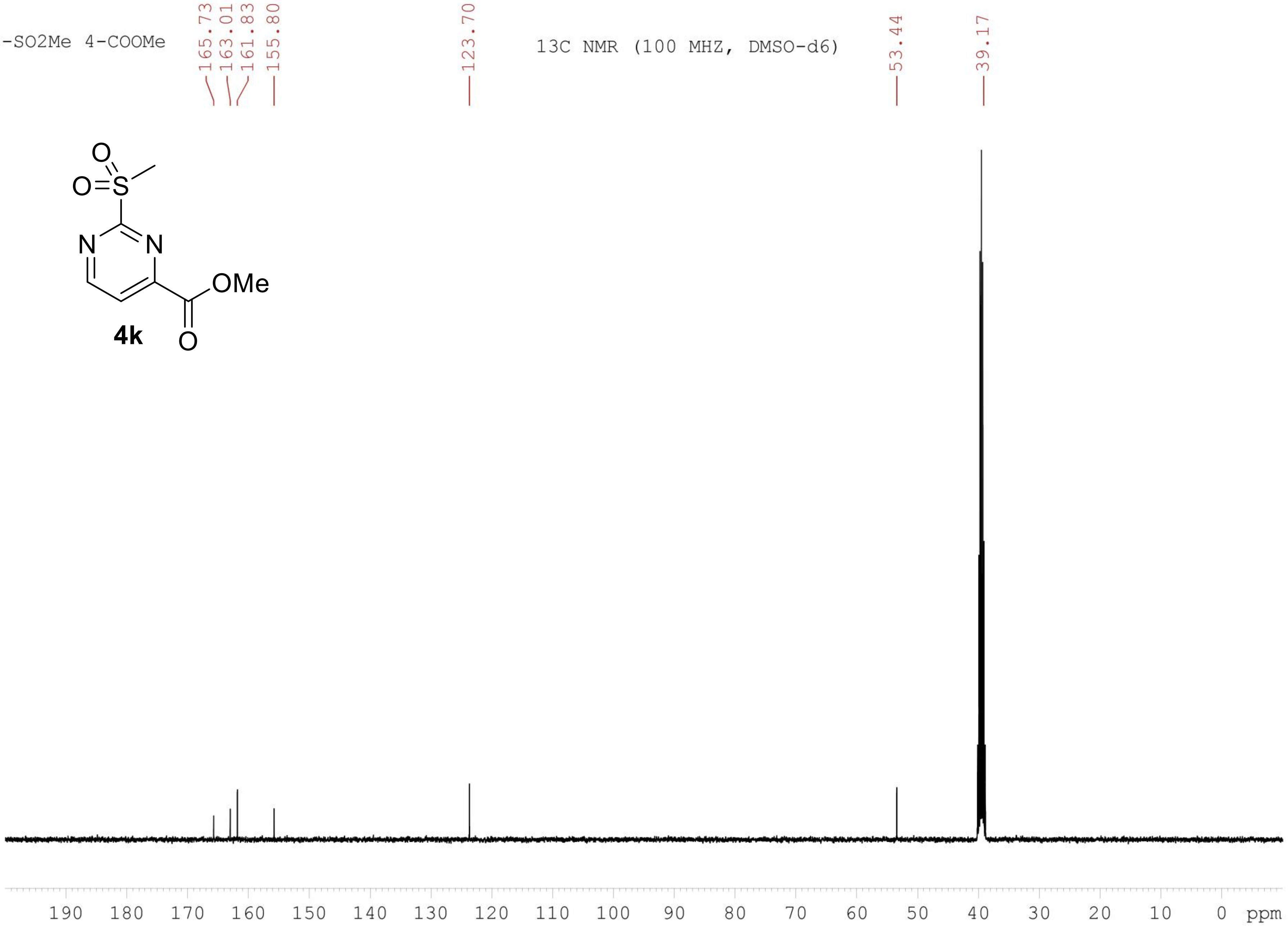

4k

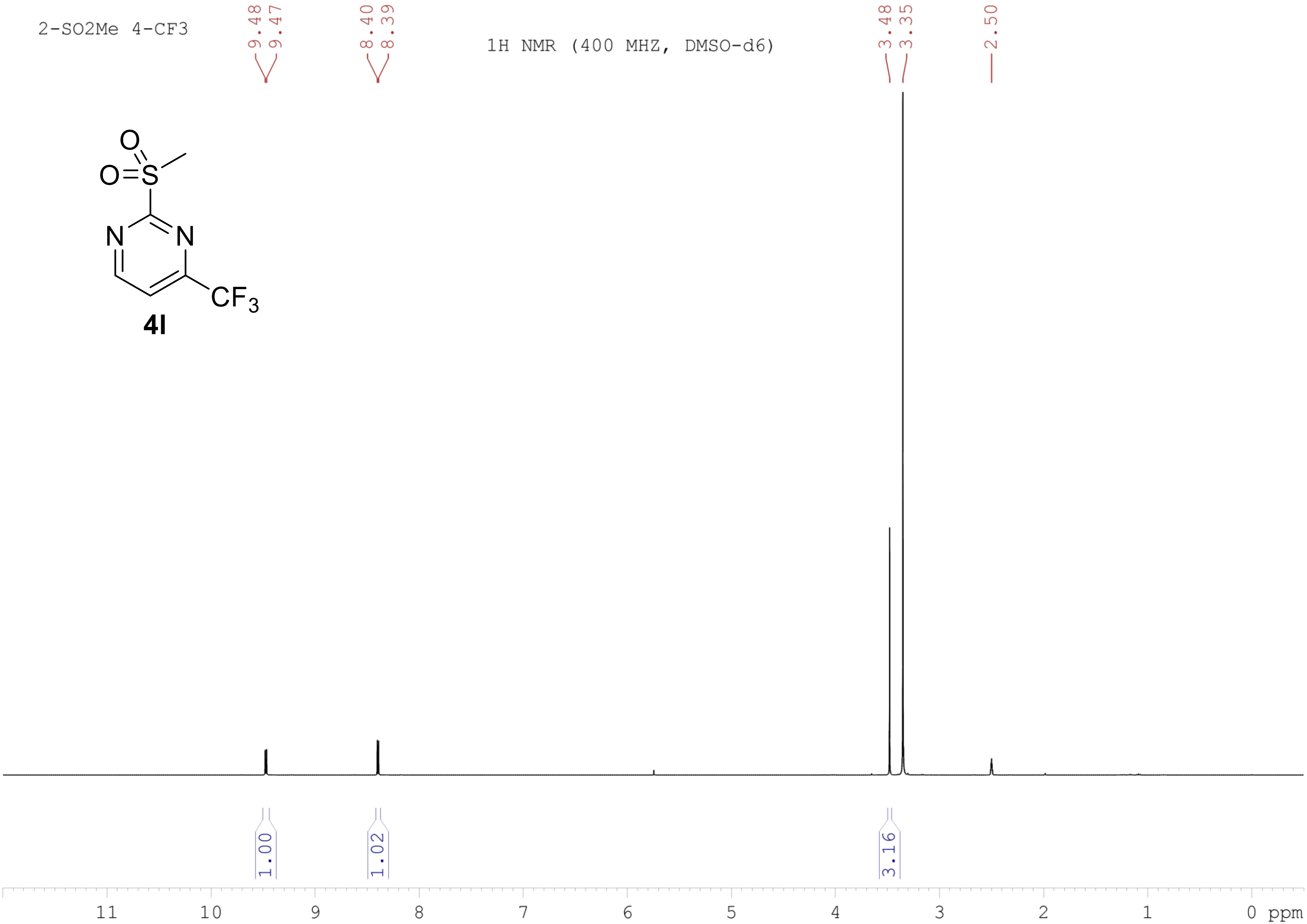

2-SO2Me 4-CF3
1H NMR (400 MHZ, DMSO-d6)
9.48
9.47
8.40
8.39
3.48
3.35
2.50
4l
1.00
1.02
3.16
11
10
9
8
7
6
5
4
3
2
1
0 ppm

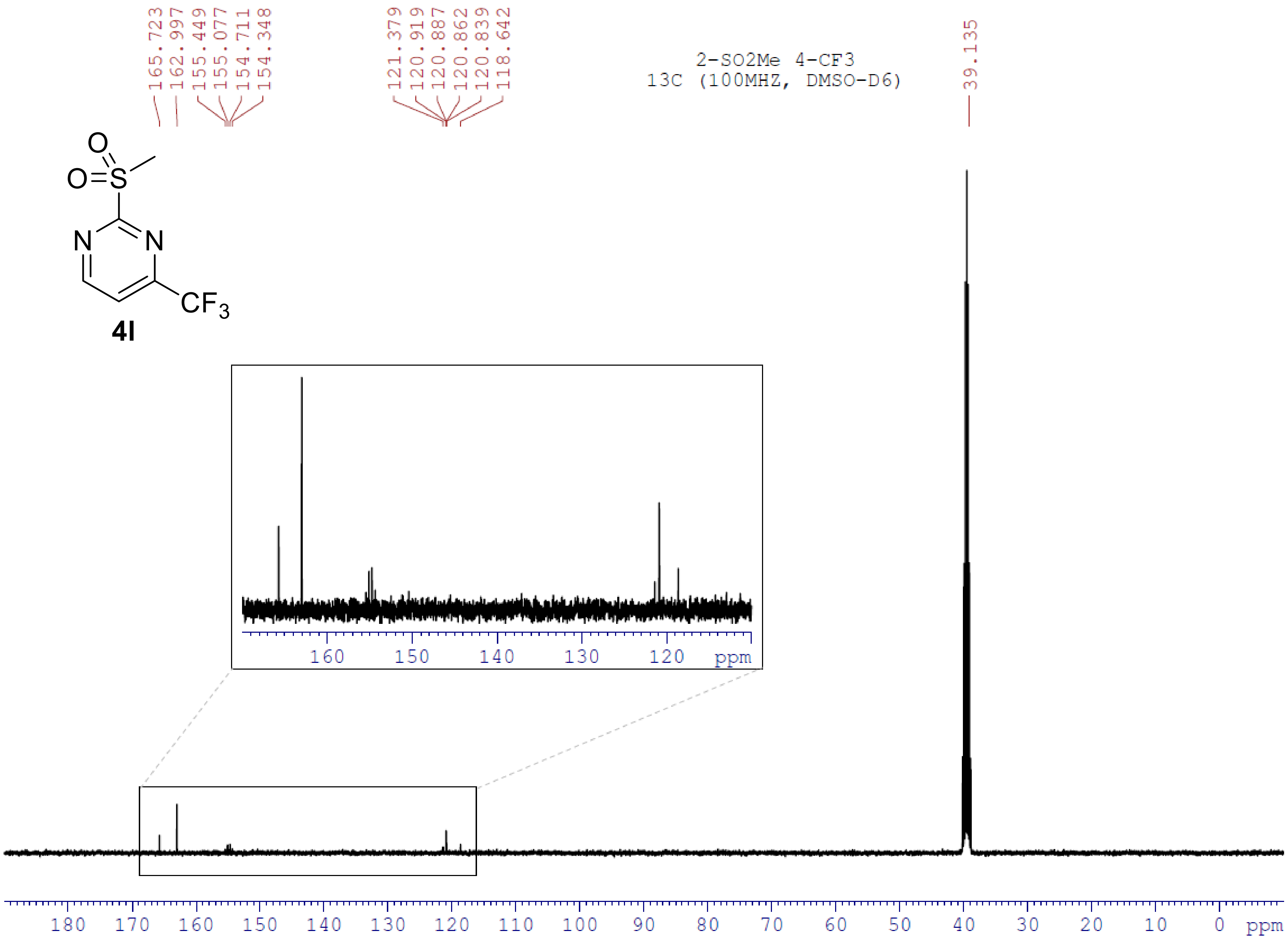
165.723
162.997
155.449
155.077
154.711
154.348
121.379
120.919
120.887
120.862
120.839
118.642
39.135
2-SO2Me 4-CF3
13C (100MHZ, DMSO-D6)
4l
160
150
140
130
120
ppm
180
170
160
150
140
130
120
110
100
90
80
70
60
50
40
30
20
10
0
ppm

2-SO2Me 4-CF3

19F NMR (376 MHZ, DMSO-d6)

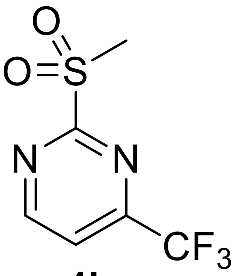

4l

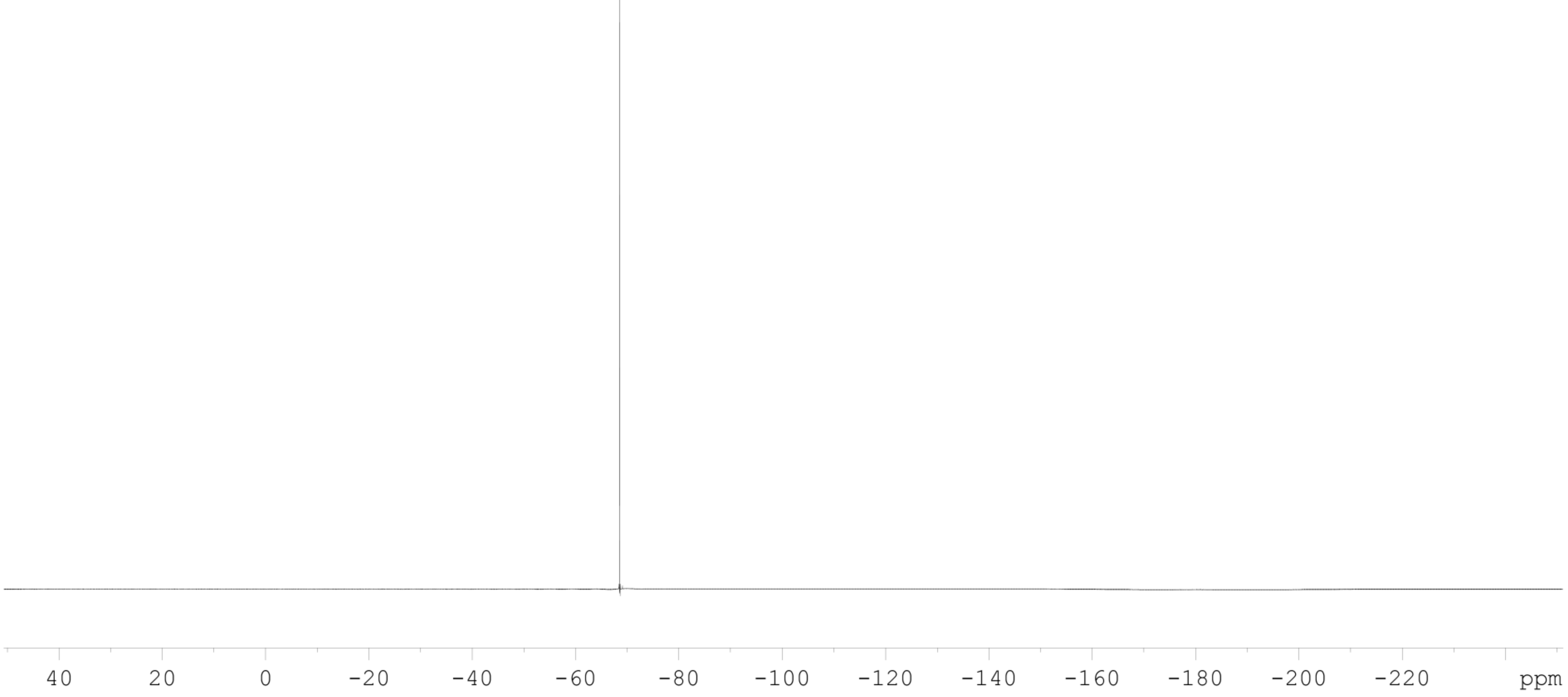

2-SO2Me 5-NH2

1H NMR (400 MHZ, DMSO-d6)

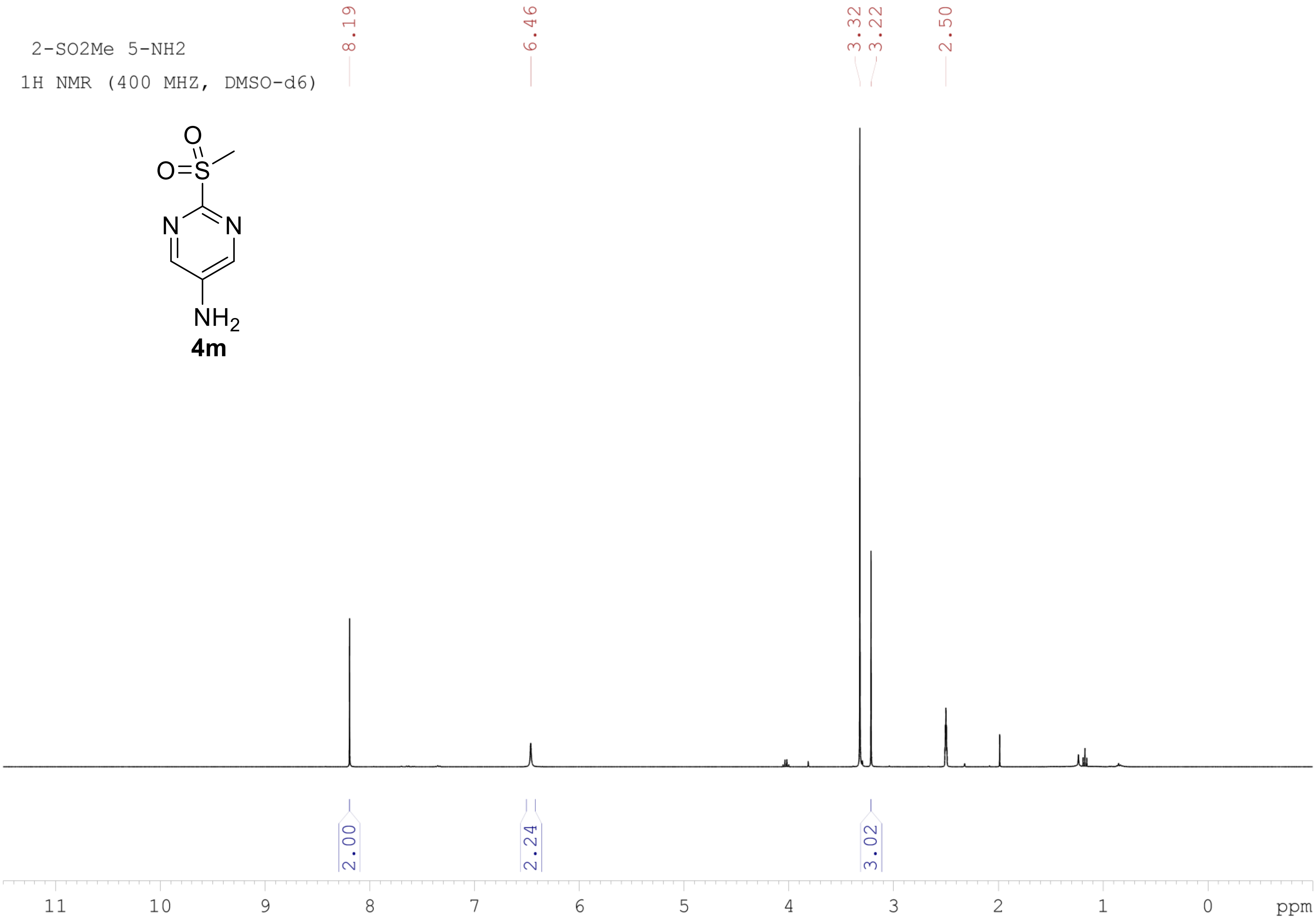

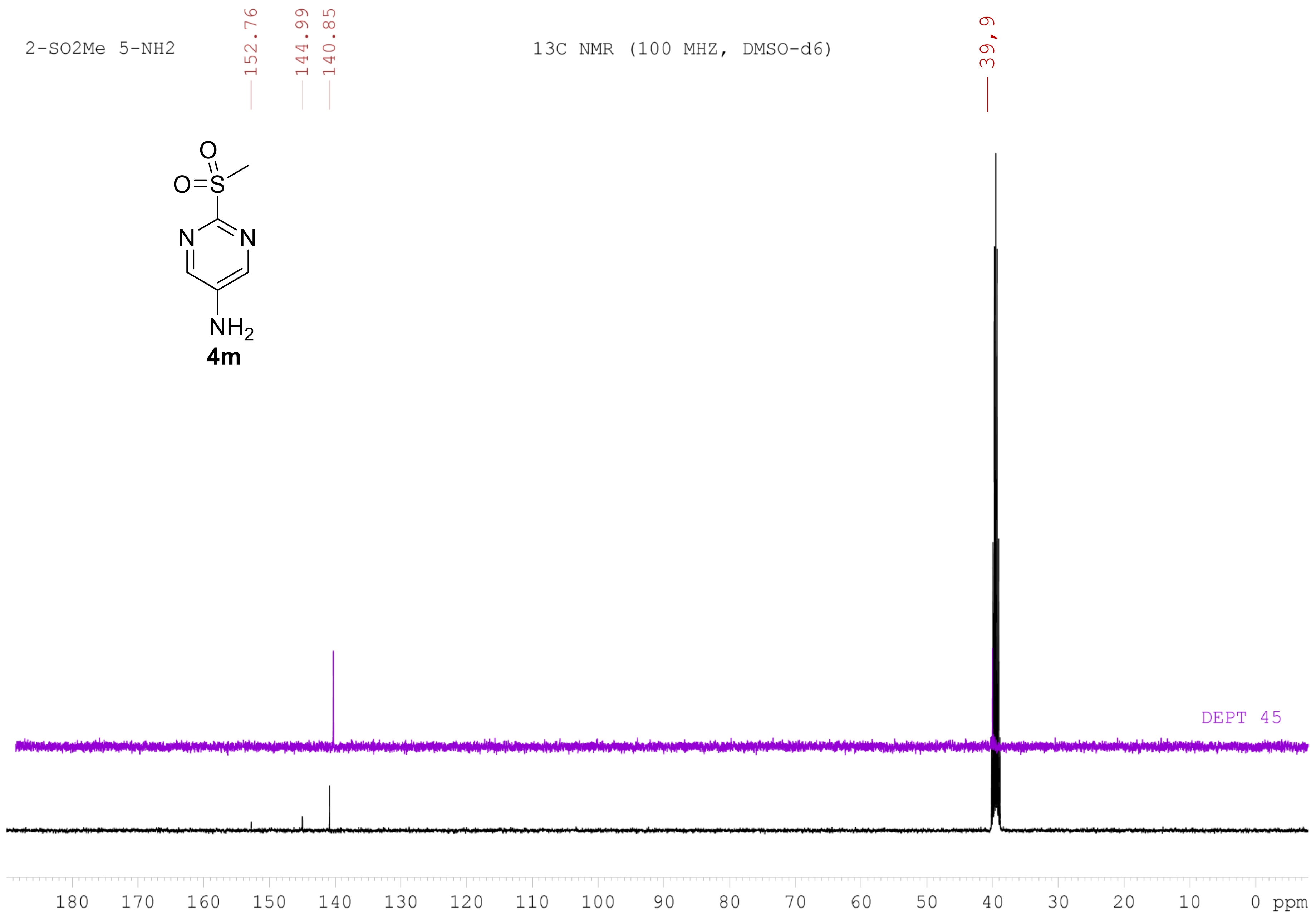
2-SO2Me 5-NH2
152.76
144.99
140.85
13C NMR (100 MHZ, DMSO-d6)
39,9
O
O=S
N
N
NH2
4m
DEPT 45
180
170
160
150
140
130
120
110
100
90
80
70
60
50
40
30
20
10
0 ppm

2-SO2Me 5-Me

1H NMR (400 MHZ, DMSO-d6)

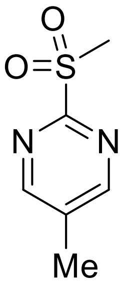

4o

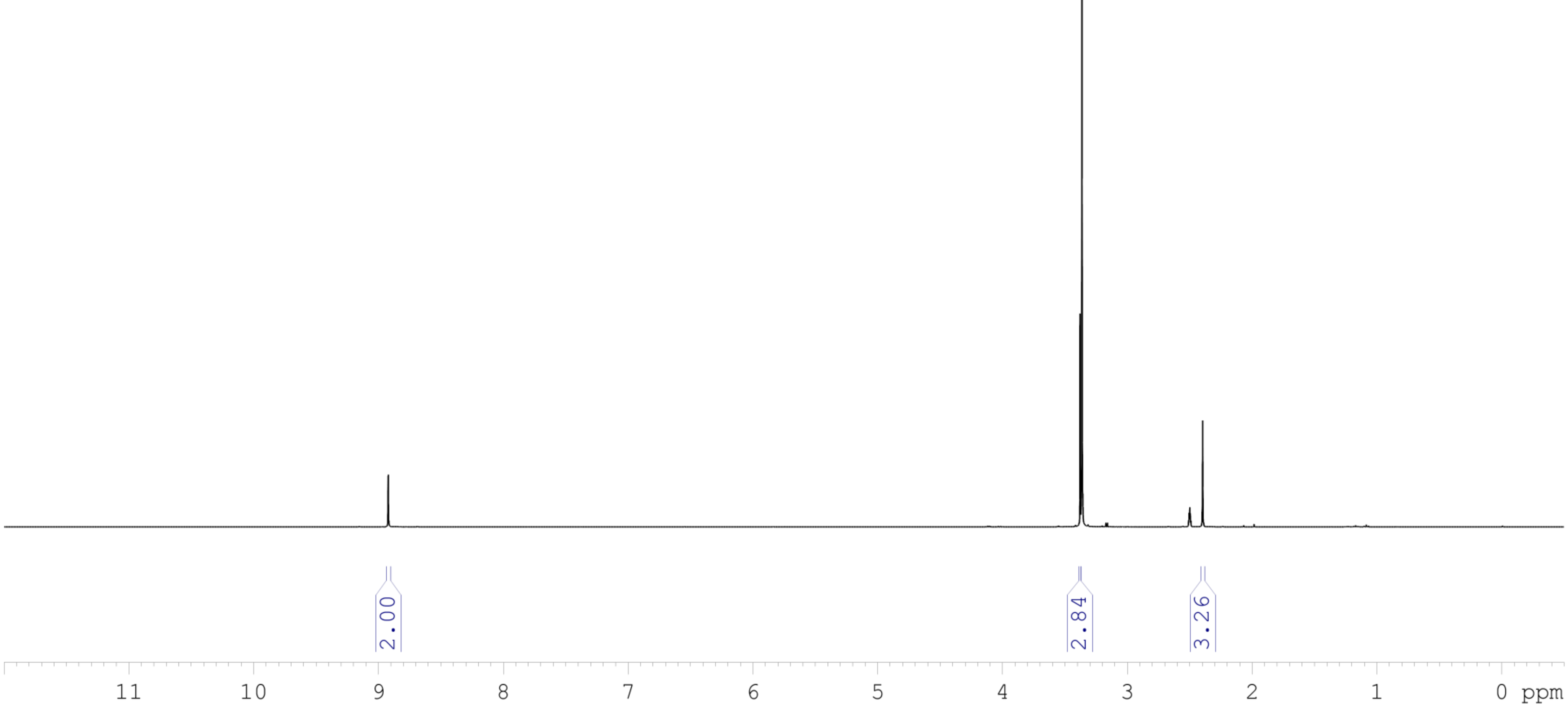

2-SO2Me 5-Me

13C NMR (100 MHZ, DMSO-d6)

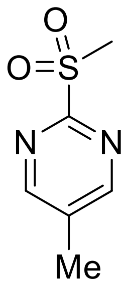

4o

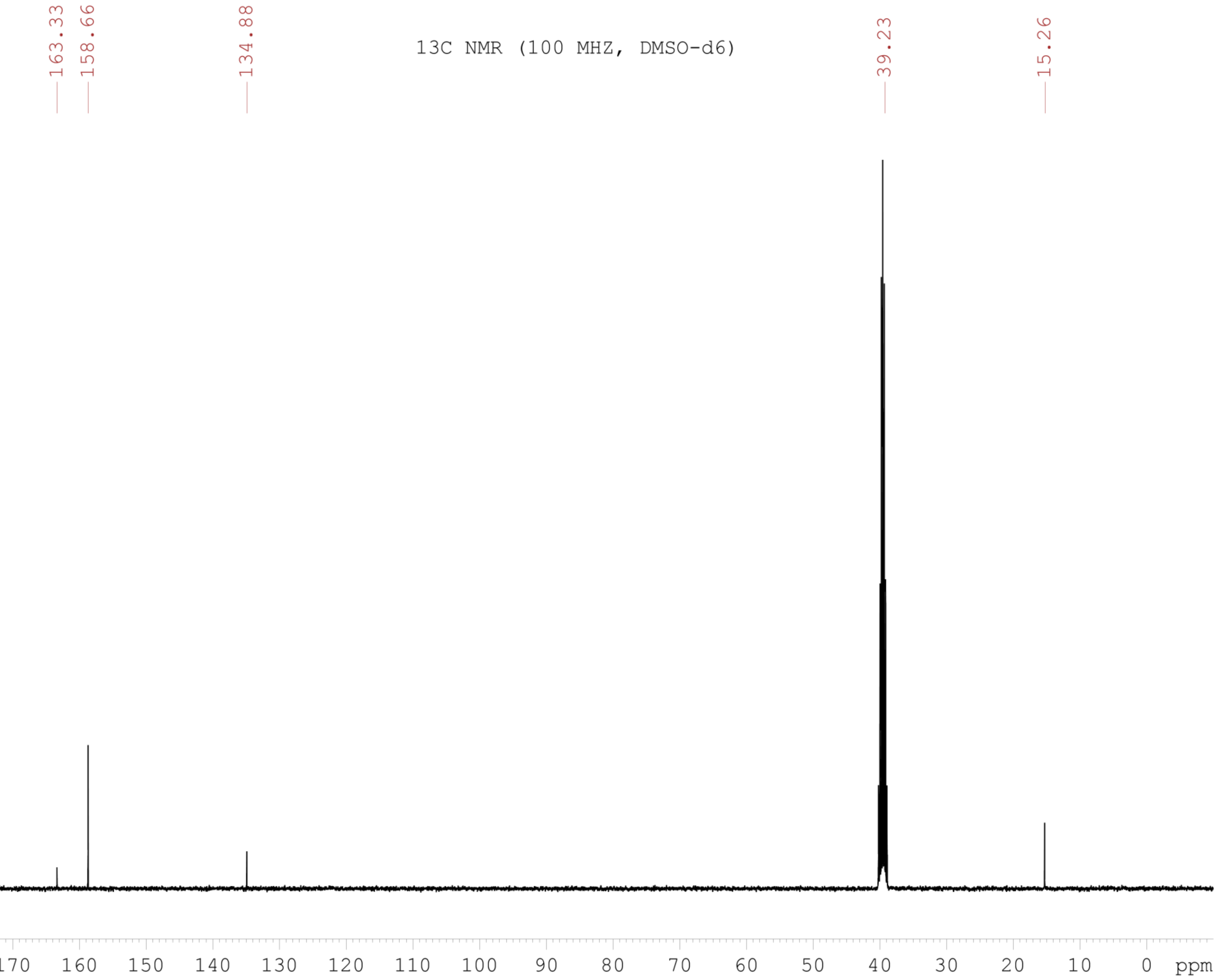

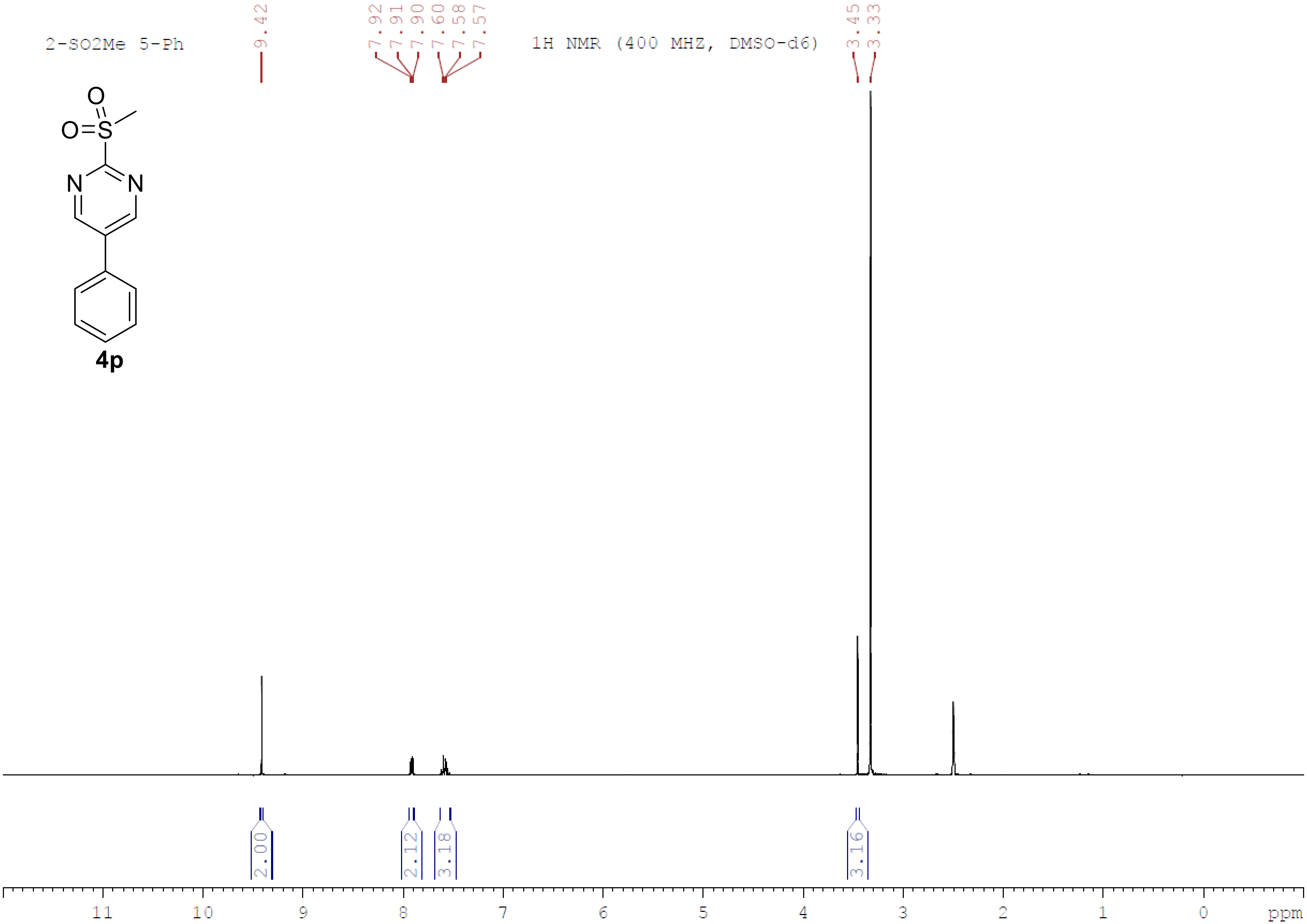
2-SO2Me 5-Ph
4p
1H NMR (400 MHZ, DMSO-d6)
9.42
7.92
7.91
7.90
7.60
7.58
7.57
3.45
3.33
2.00
2.12
3.18
3.16
11
10
9
8
7
6
5
4
3
2
1
0
ppm

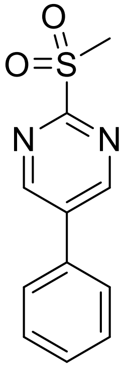

4p

2-SO2Me 5-Ph

13C NMR (100 MHZ, DMSO-d6)

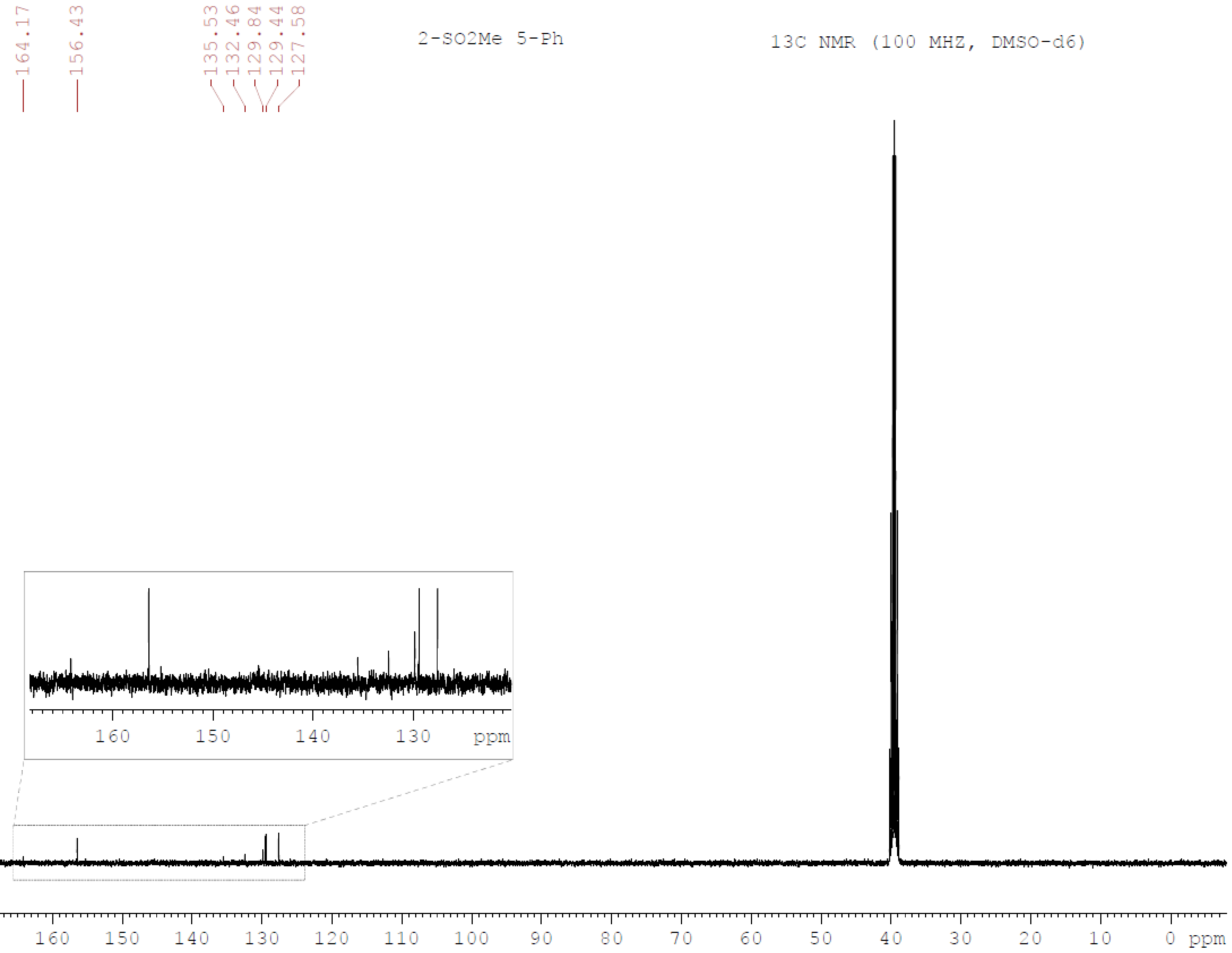

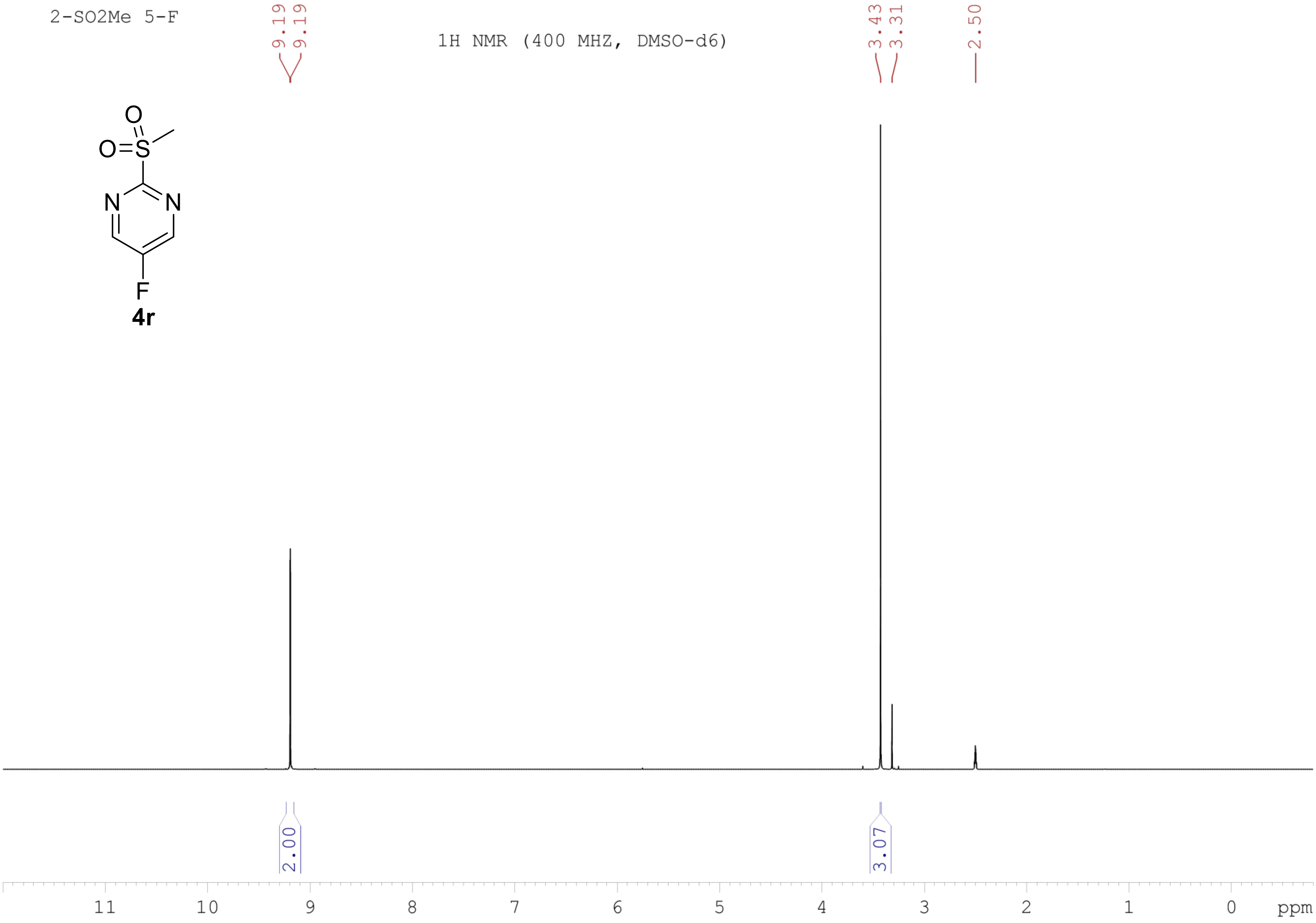
2-SO2Me 5-F
1H NMR (400 MHZ, DMSO-d6)
9.19
9.19
3.43
3.31
2.50
O
O=S
N
N
F
4r
2.00
3.07
11
10
9
8
7
6
5
4
3
2
1
0
ppm

2-SO2Me 5-F

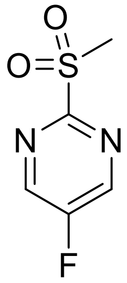

4r

13C NMR (100 MHZ, DMSO-d6)

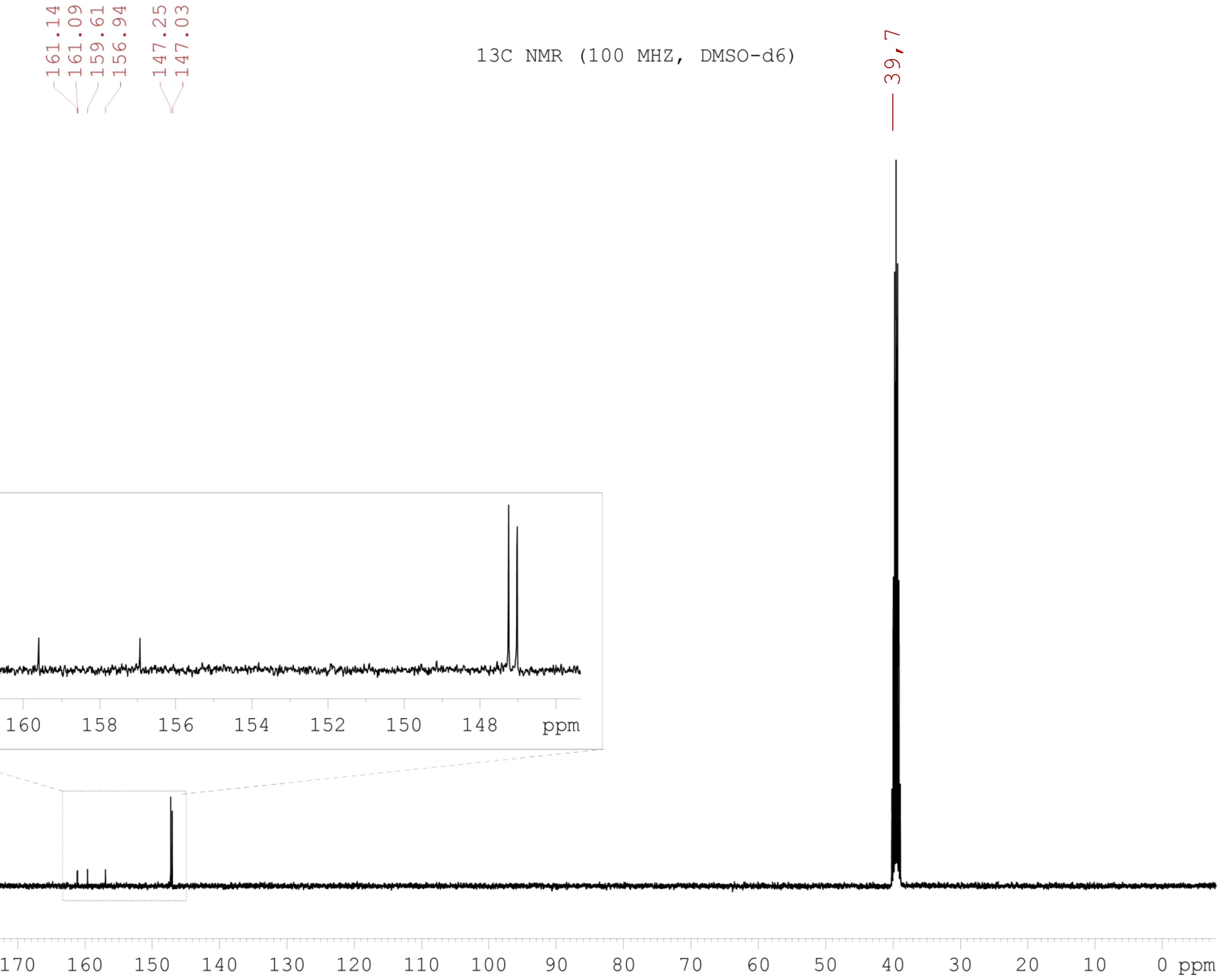

2-SO2Me 5-F

19F NMR (376 MHZ, DMSO-d6)

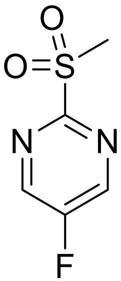

4r

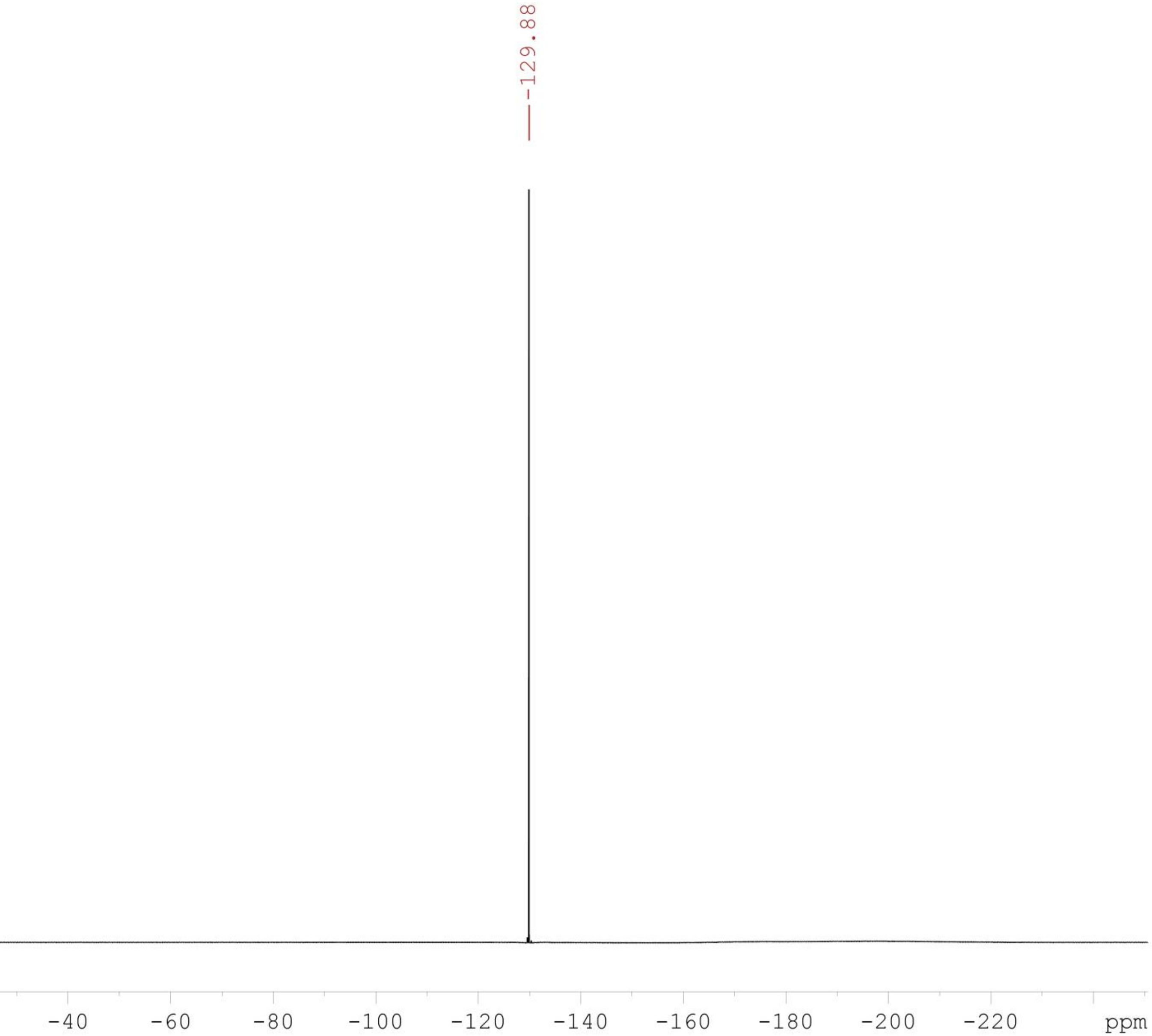

2-SO2Me 5-Cl

1H NMR (400 MHZ, DMSO-d6)

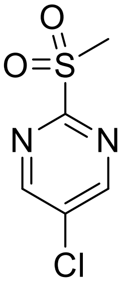

4s

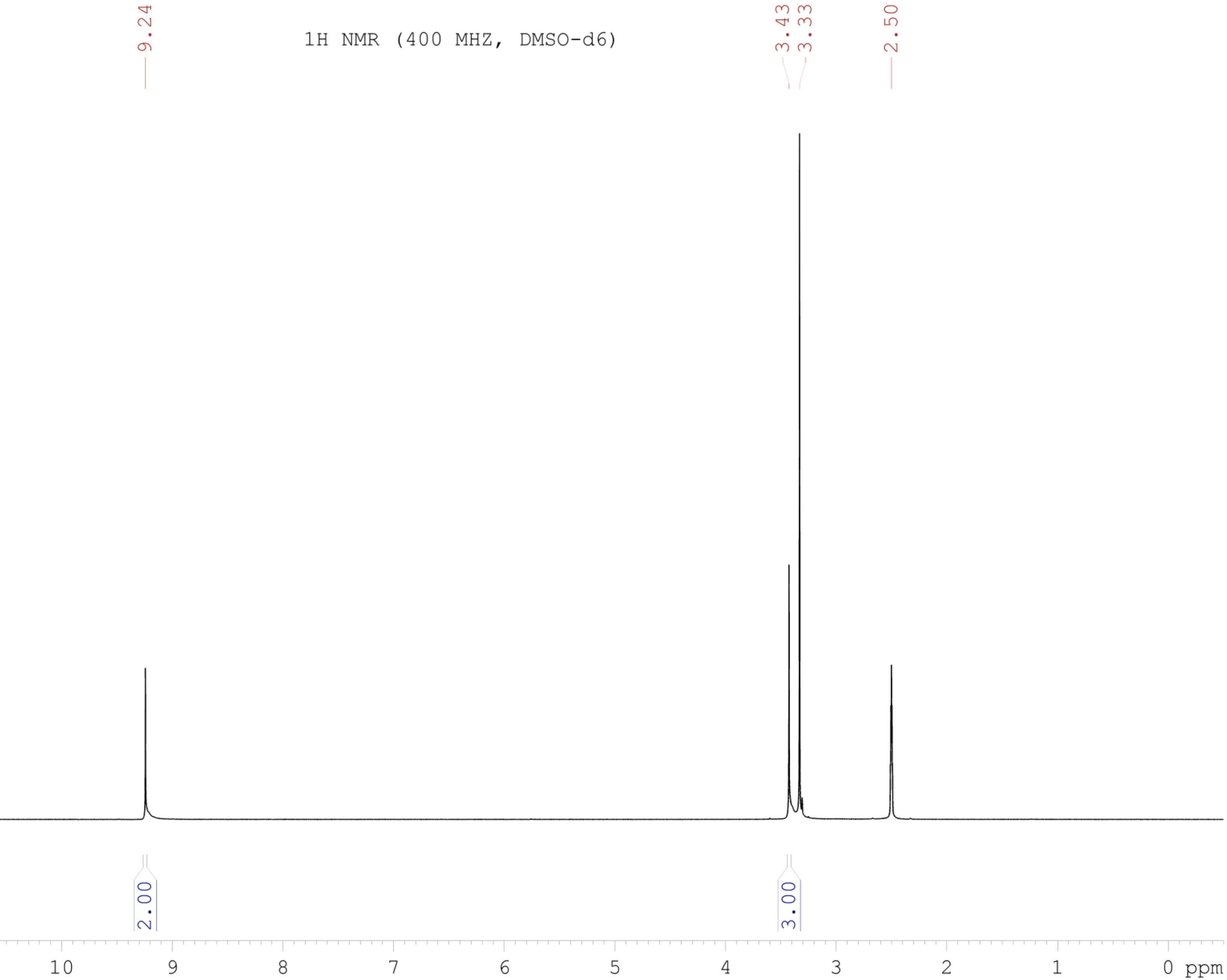

2-SO2Me 5-Br

1H NMR (400 MHZ, DMSO-d6)

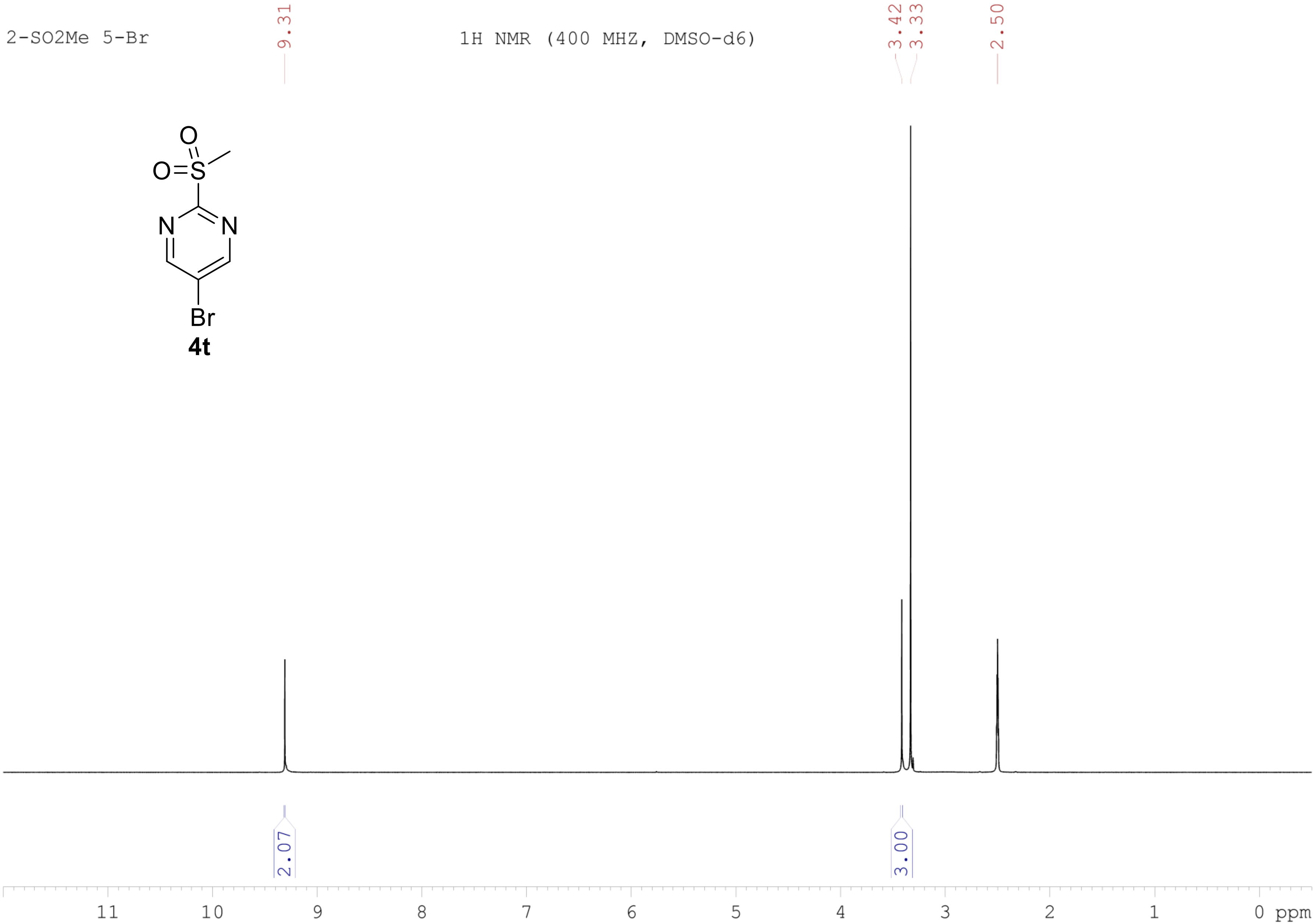

4t

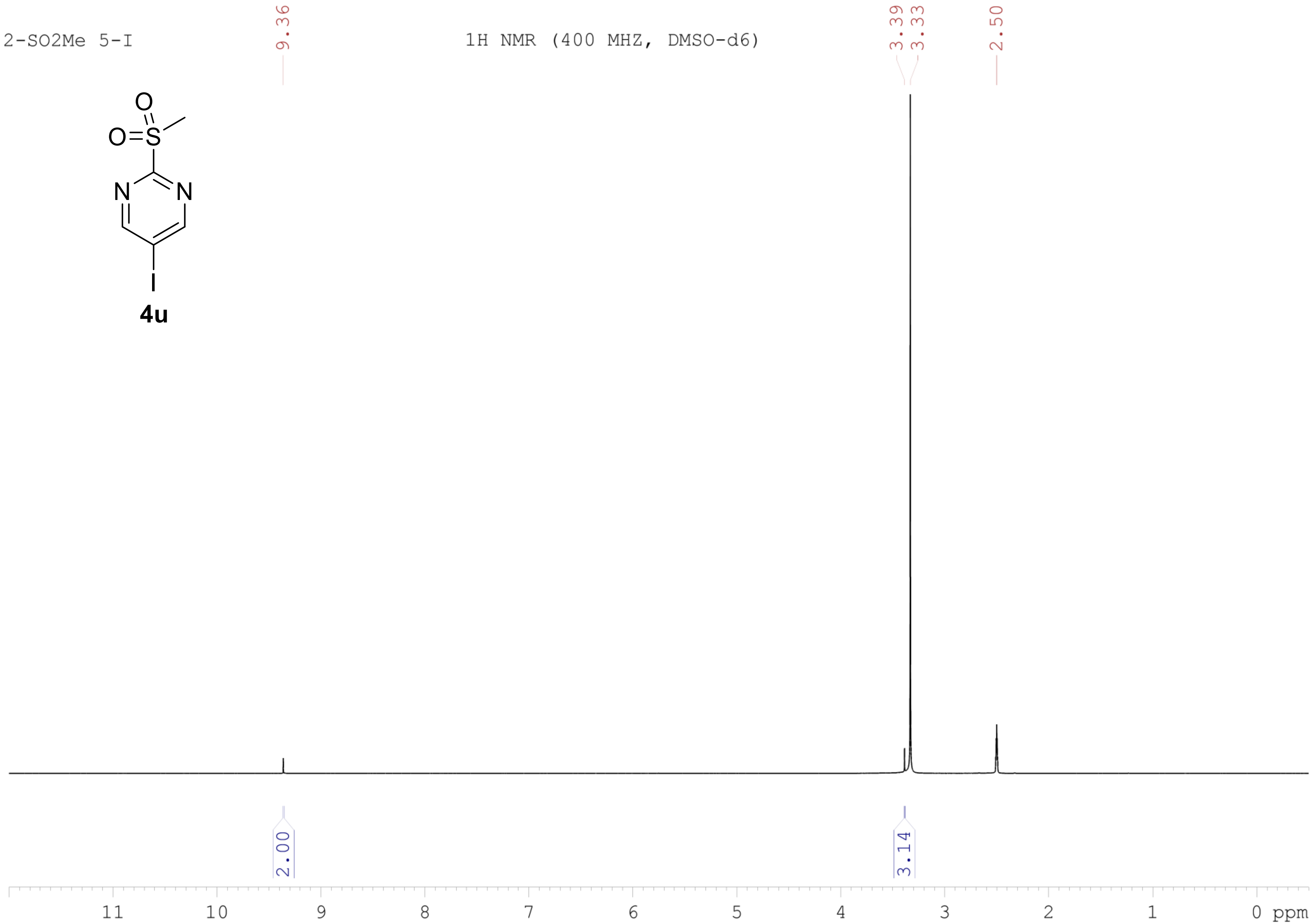
2-SO2Me 5-I
1H NMR (400 MHZ, DMSO-d6)
9.36
3.39
3.33
2.50
4u
2.00
3.14
11 10 9 8 7 6 5 4 3 2 1 0 ppm

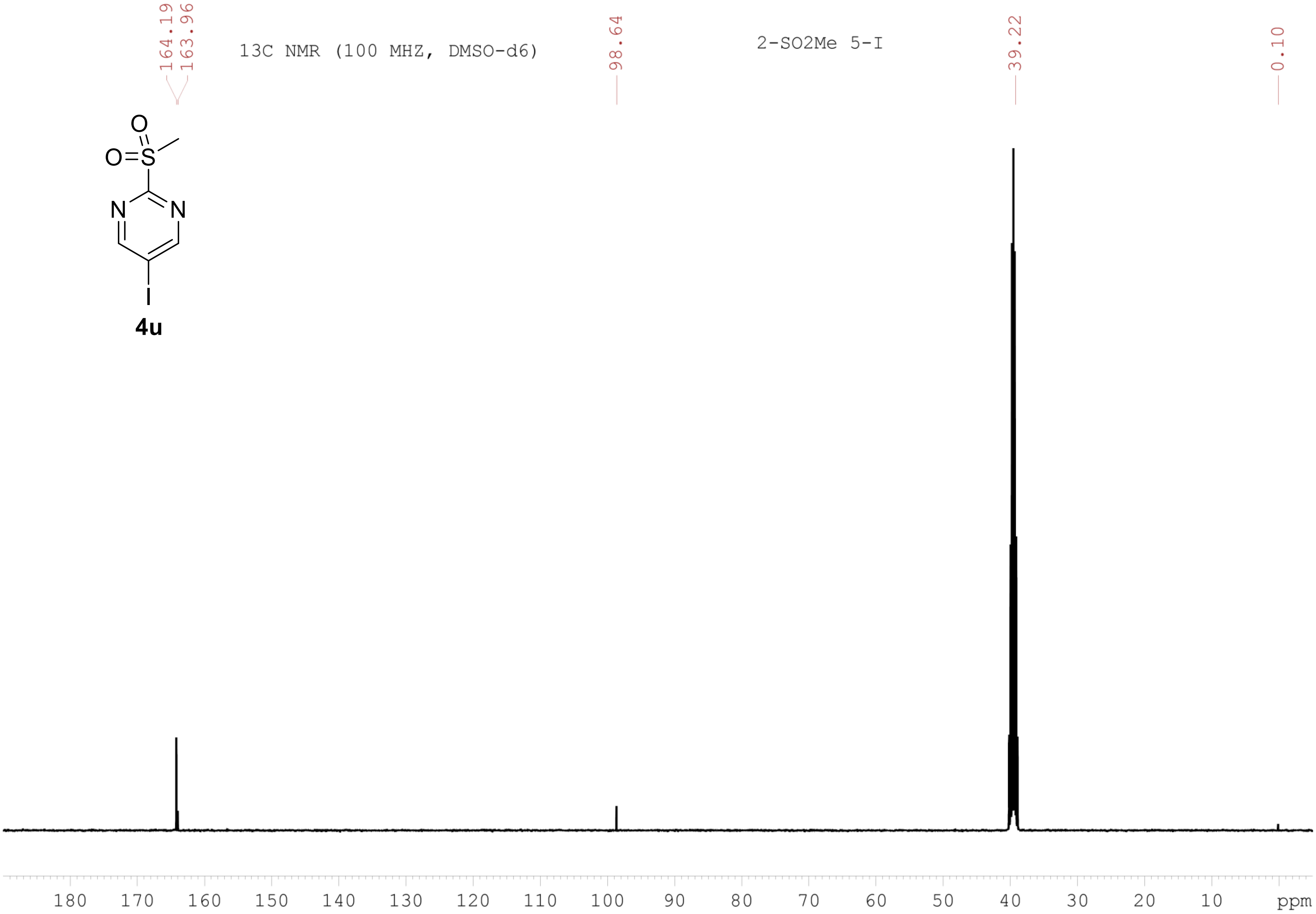
13C NMR (100 MHZ, DMSO-d6)
2-SO2Me 5-I
164.19
163.96
98.64
39.22
0.10
4u
180 170 160 150 140 130 120 110 100 90 80 70 60 50 40 30 20 10 ppm

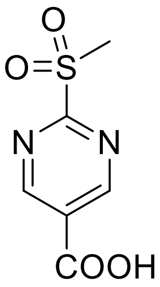
O
O=S
N
N
COOH

4v

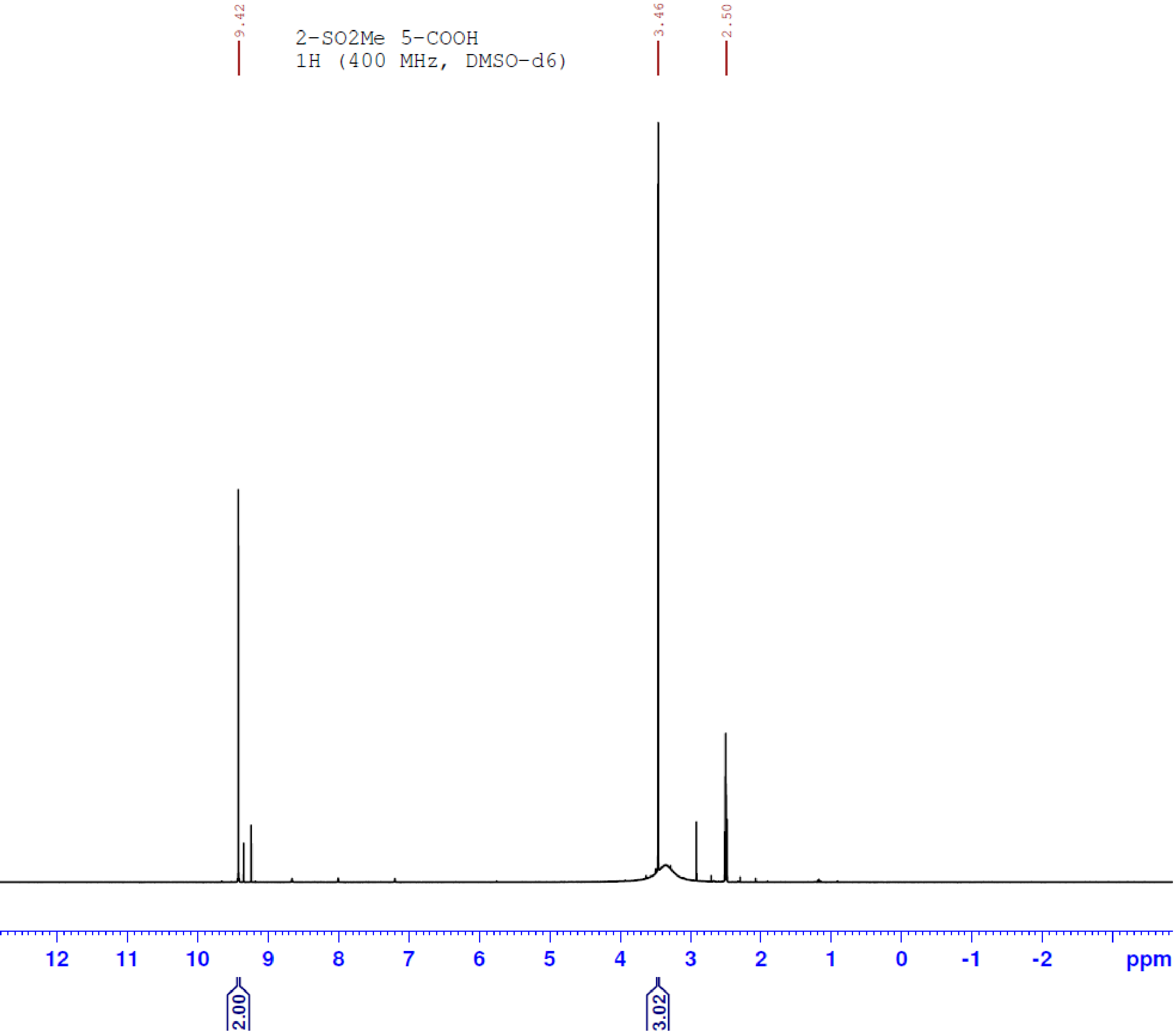
9.42
2-SO2Me 5-COOH
1H (400 MHz, DMSO-d6)
3.46
2.50
16 15 14 13 12 11 10 9 8 7 6 5 4 3 2 1 0 -1 -2 ppm
2.00
3.02

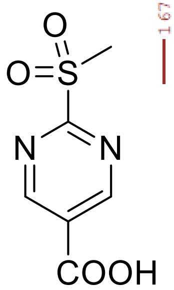

O
O=S
N
N
COOH

4v

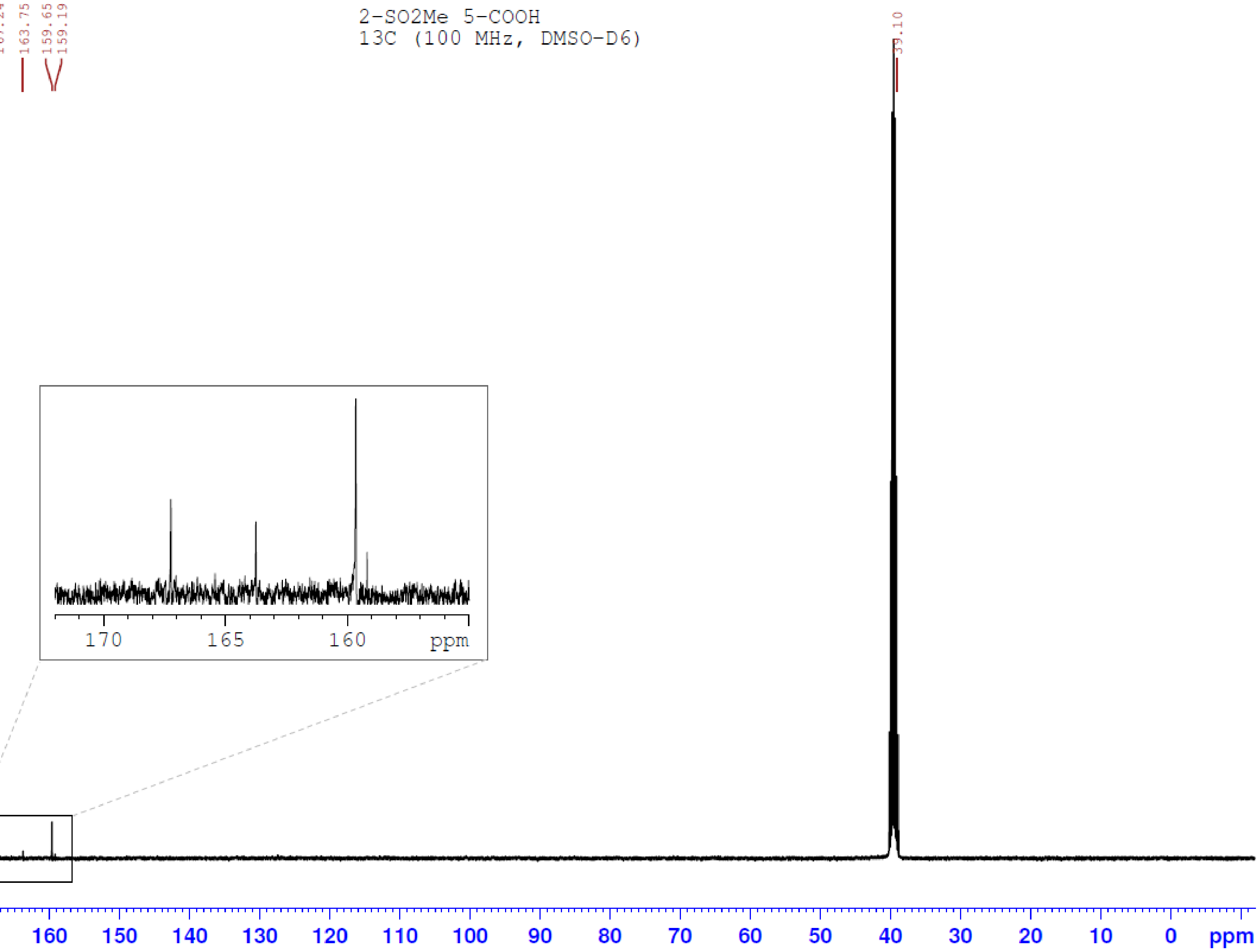

2-SO2Me 5-COOH
13C (100 MHz, DMSO-D6)
167.24
163.75
159.65
159.19
39.10
170
165
160
ppm
180
170
160
150
140
130
120
110
100
90
80
70
60
50
40
30
20
10
0
ppm

2-SO2Me 5-NO2
1H (400 MHz, CDCl3)

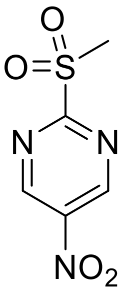

4w

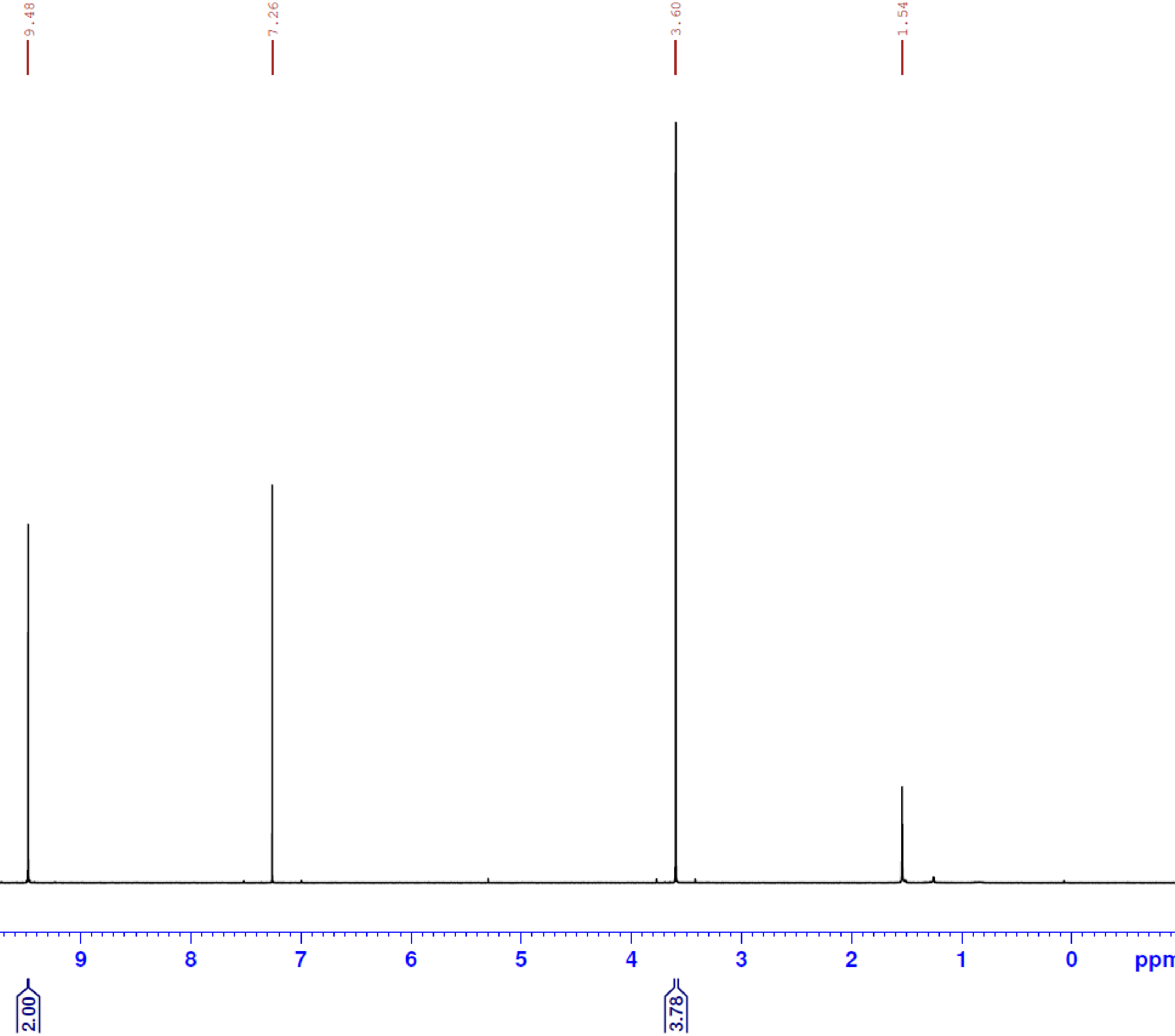

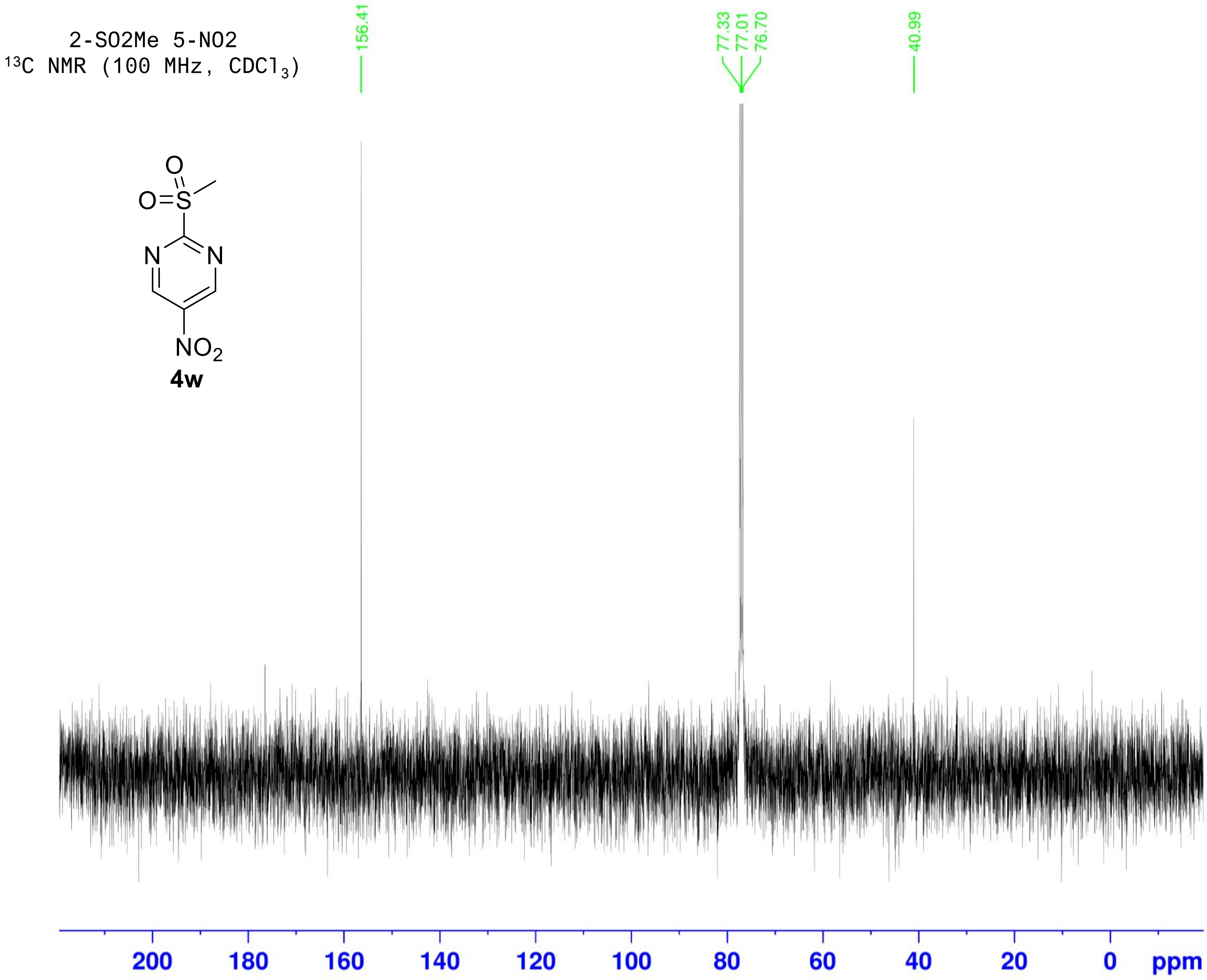
2-SO2Me 5-NO2
13C NMR (100 MHz, CDCl3)
156.41
77.33
77.01
76.70
40.99
O
O=S
N
N
NO2
4w
200
180
160
140
120
100
80
60
40
20
0
ppm

2-SO2Me 5-CF3

1H NMR (400 MHZ, DMSO-d6)

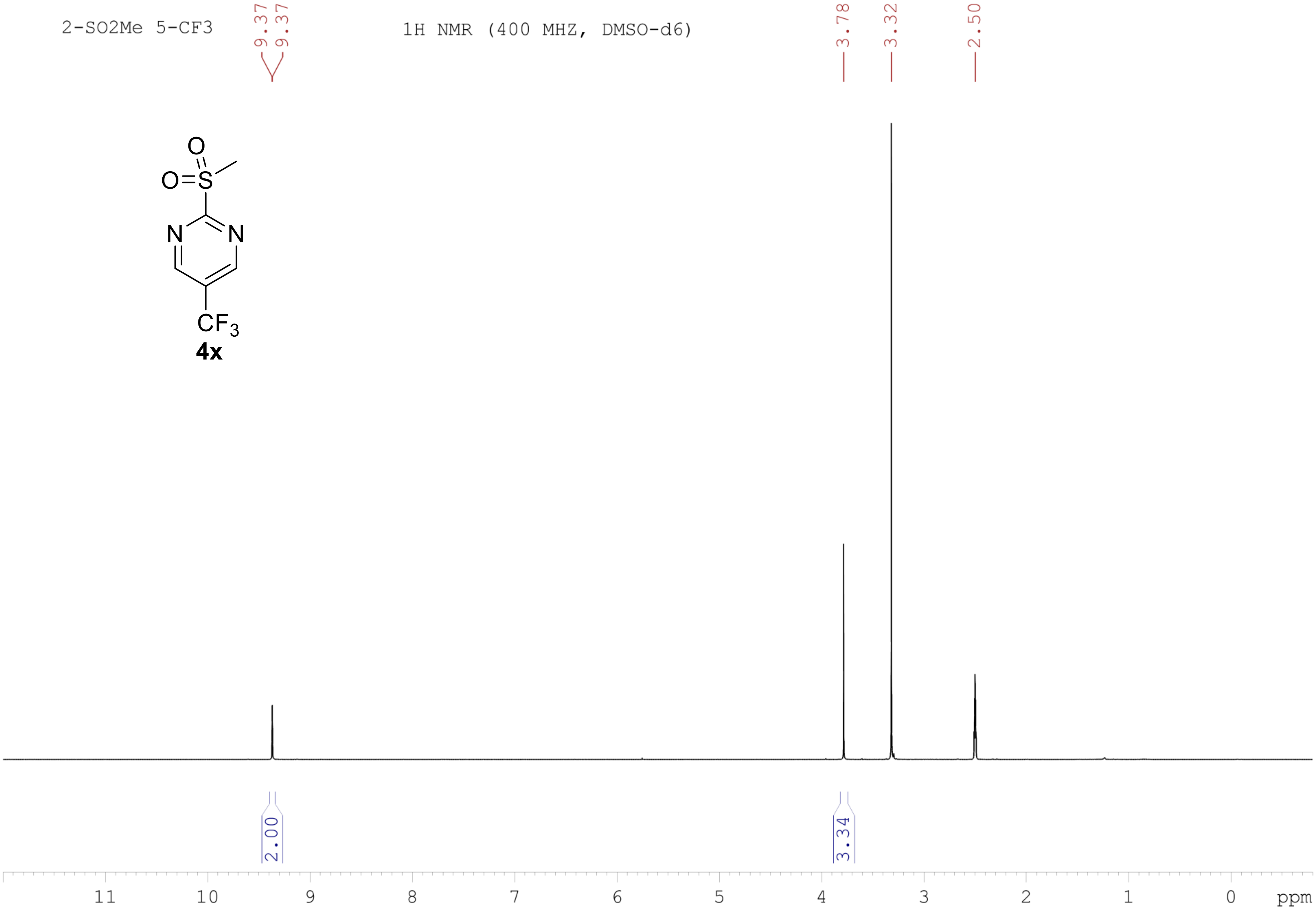

2-SO2Me 5-CF3

13C NMR (100 MHZ, DMSO-d6)

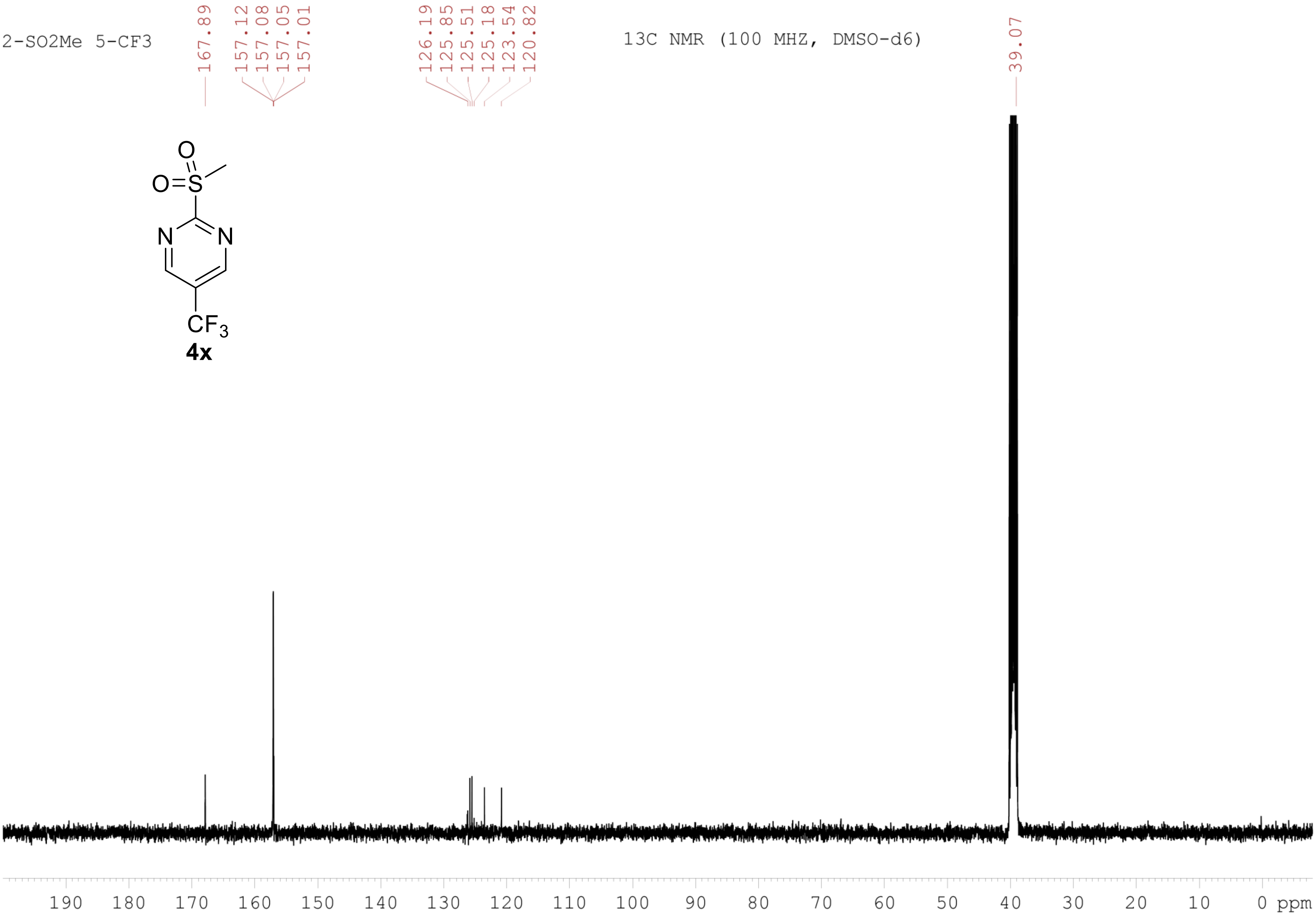

4x

2-SO2Me 5-CF3

19F NMR (376 MHZ, DMSO-d6)

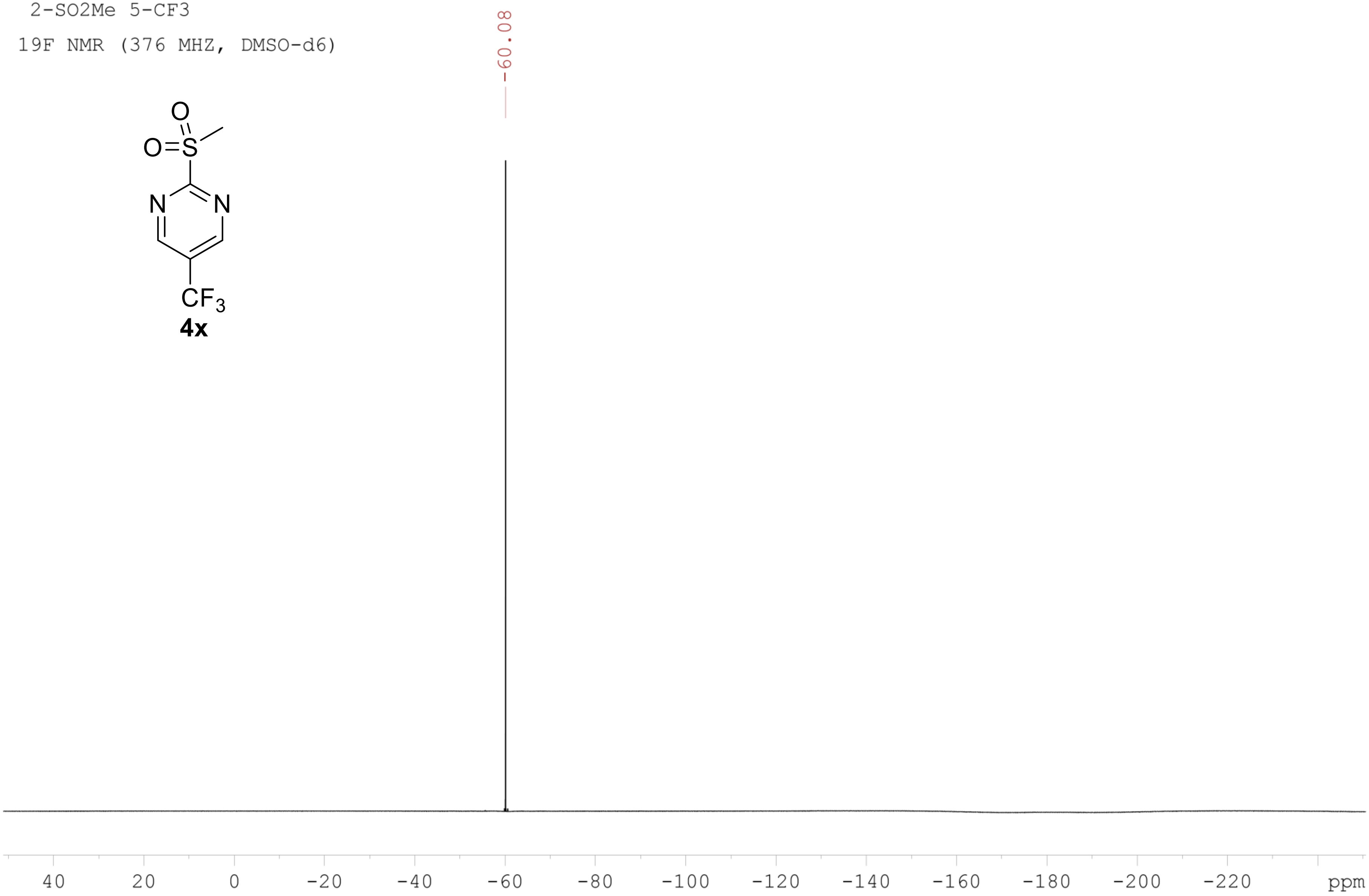

2-SO2Me 5-COOMe

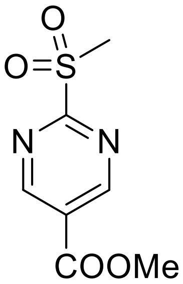

4y

1H NMR (400 MHZ, DMSO-d6)

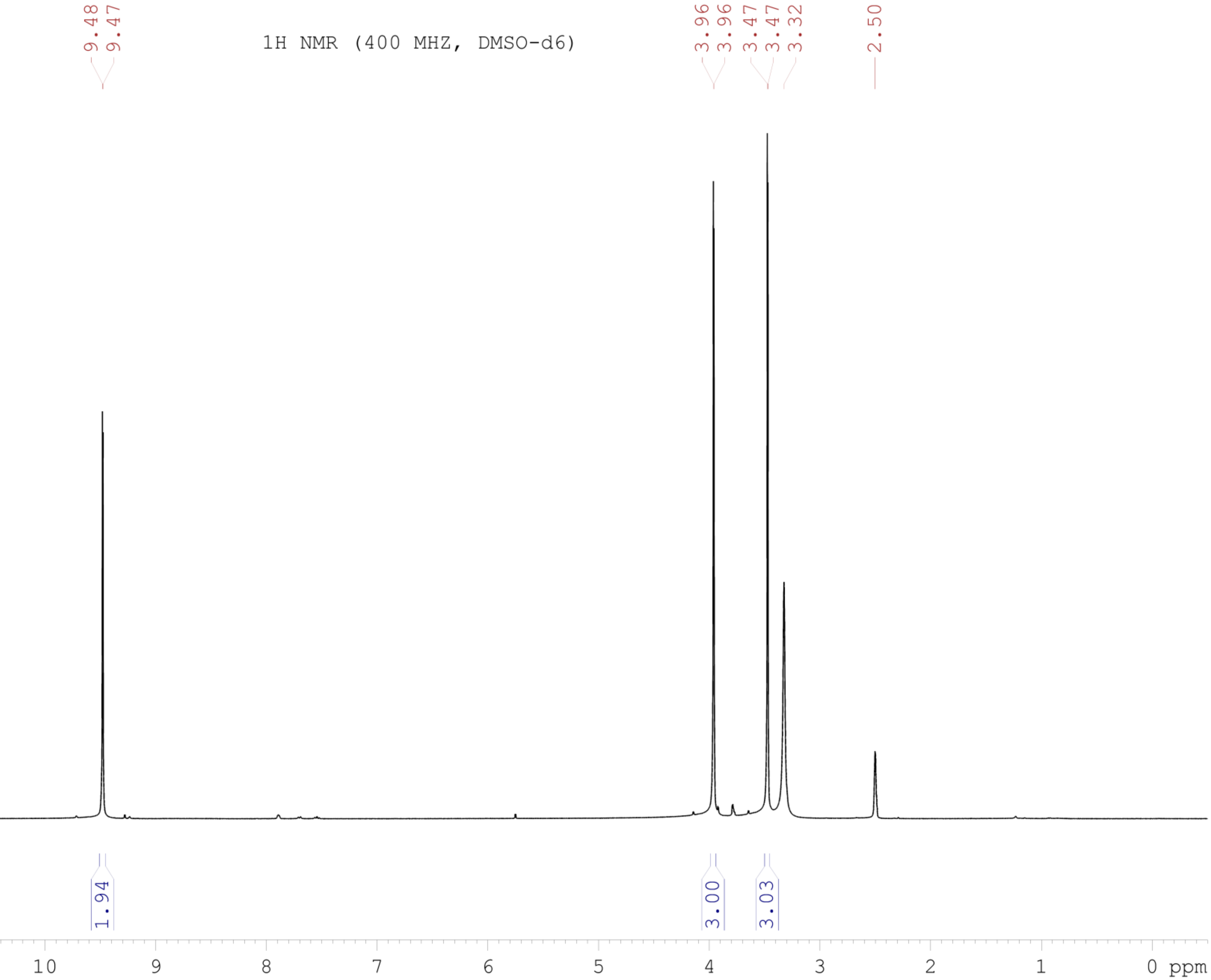

2-SO2Me 5-COOMe

13C NMR (100 MHZ, DMSO-d6)

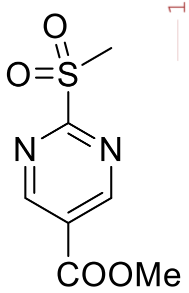

4y

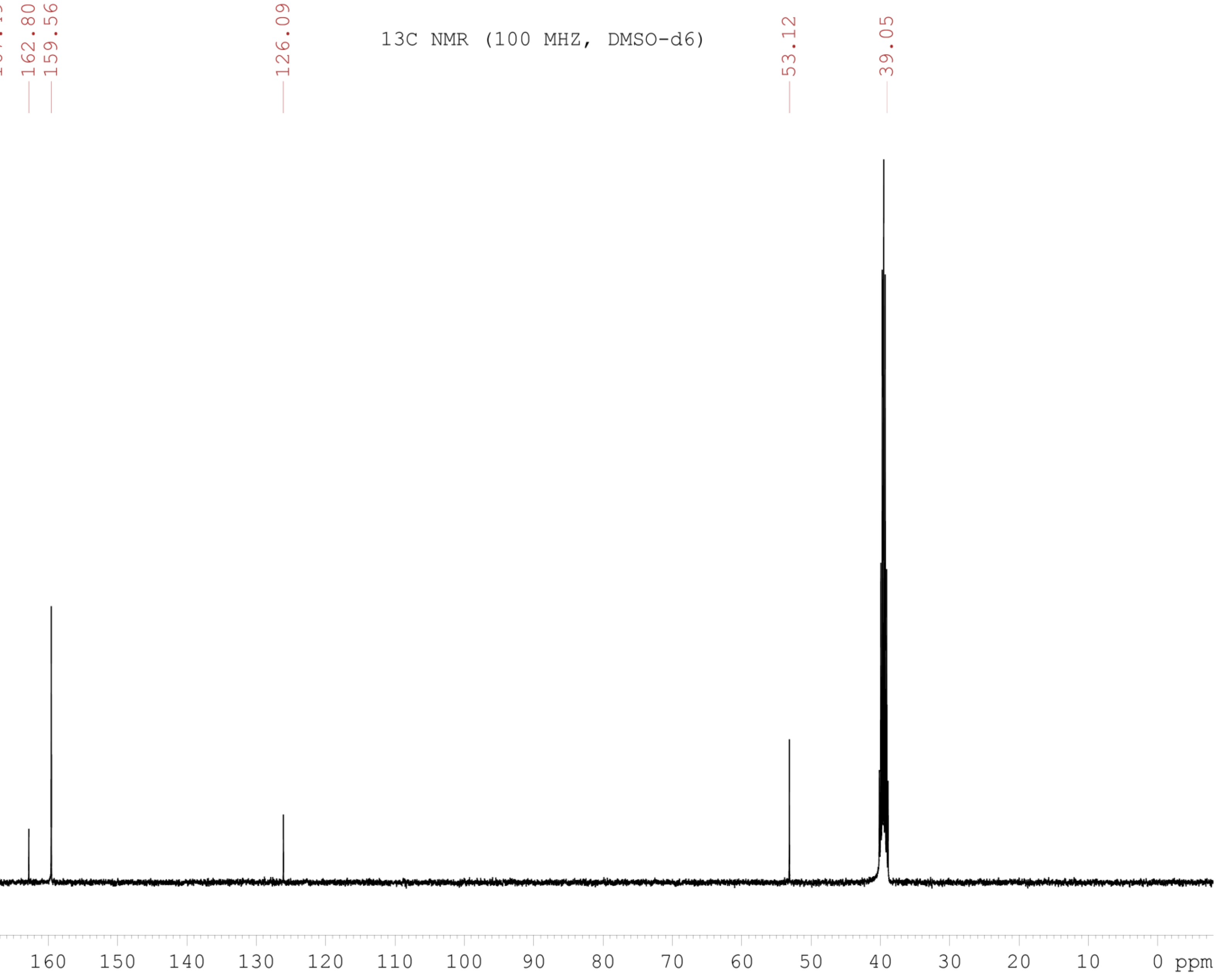

2-SO2tBu pyrimidine

1H NMR (400 MHZ, DMSO-d6)

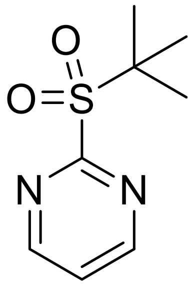

5a

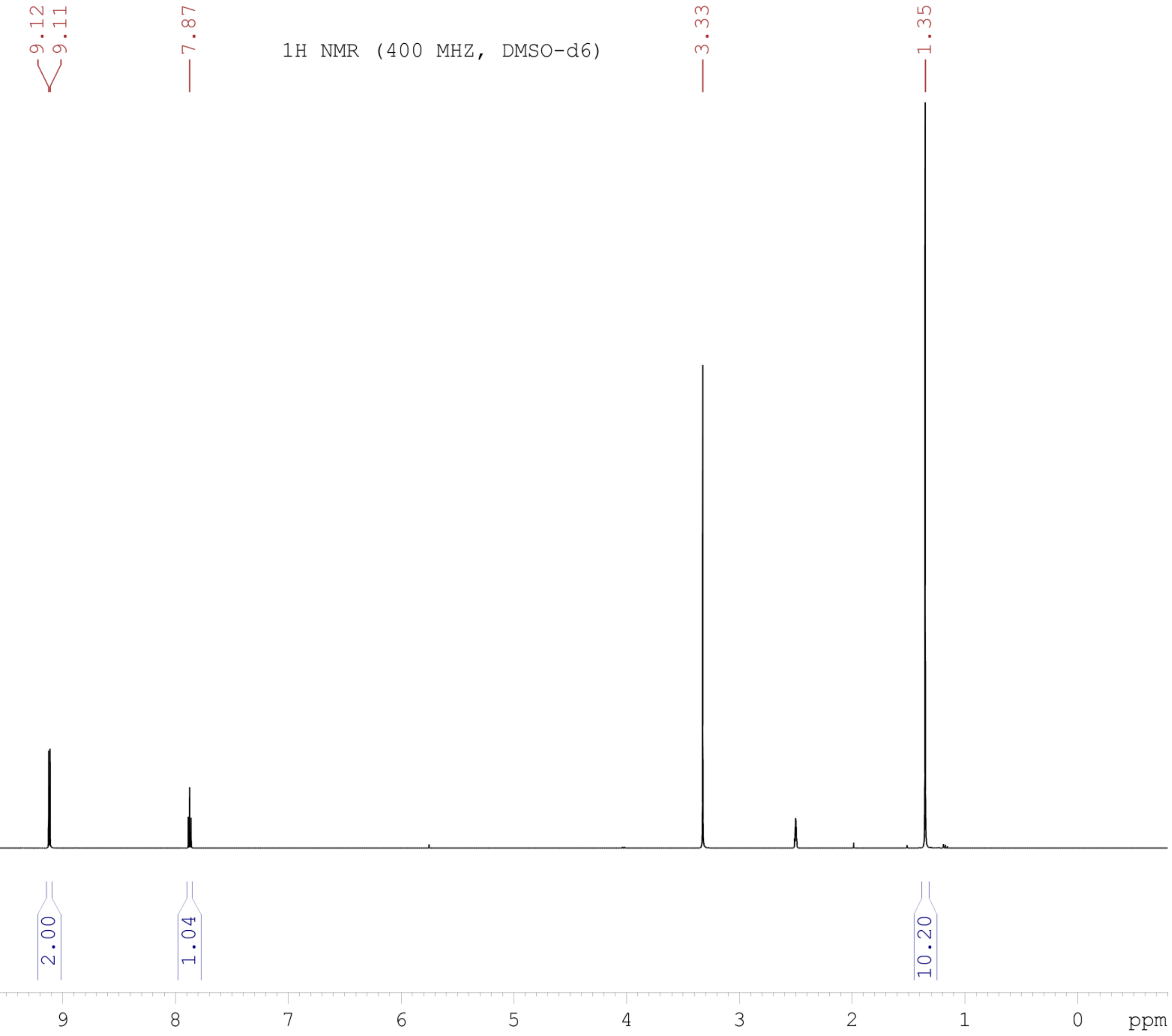

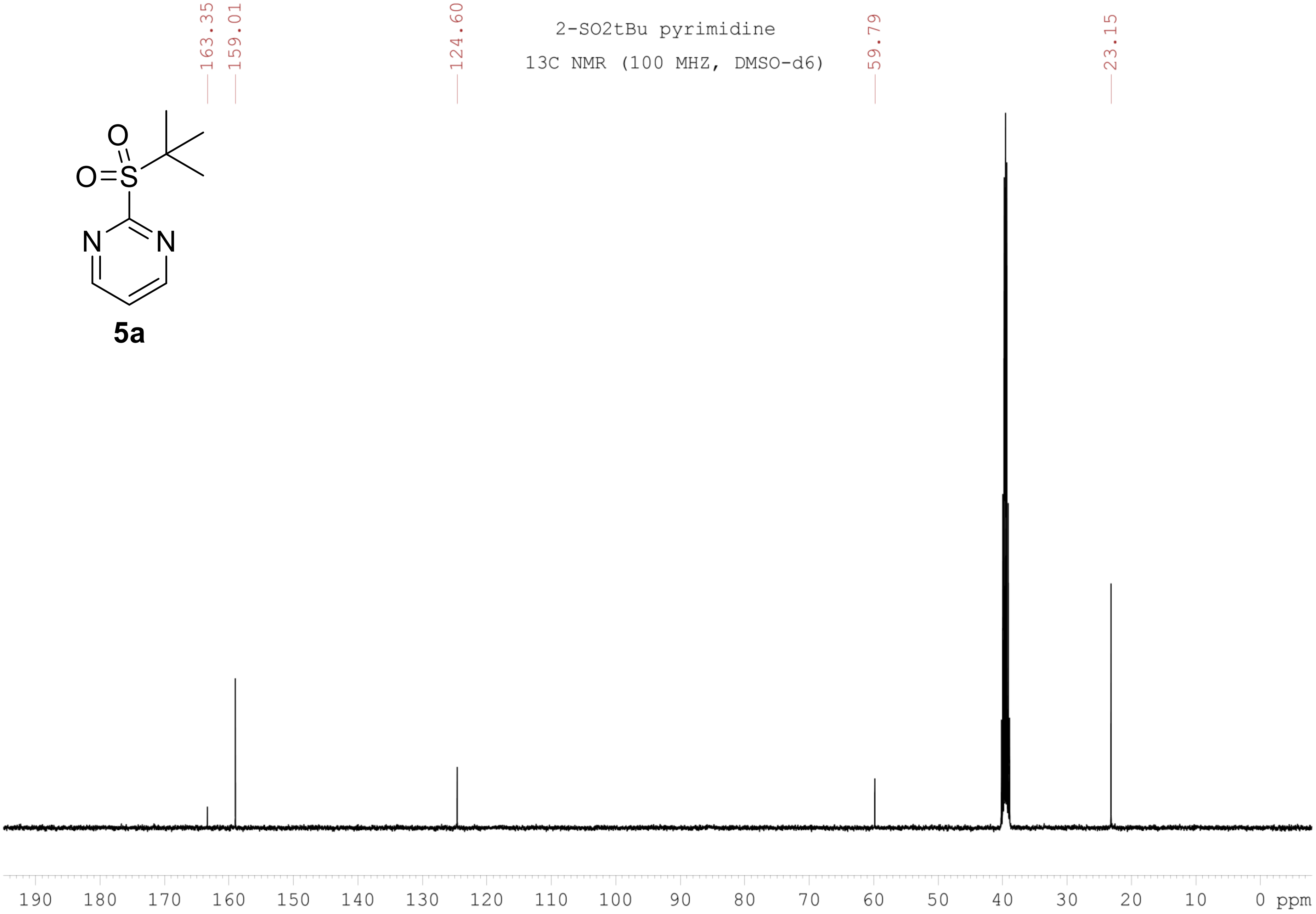
2-SO2tBu pyrimidine
13C NMR (100 MHZ, DMSO-d6)
163.35
159.01
124.60
59.79
23.15
5a
190 180 170 160 150 140 130 120 110 100 90 80 70 60 50 40 30 20 10 0 ppm

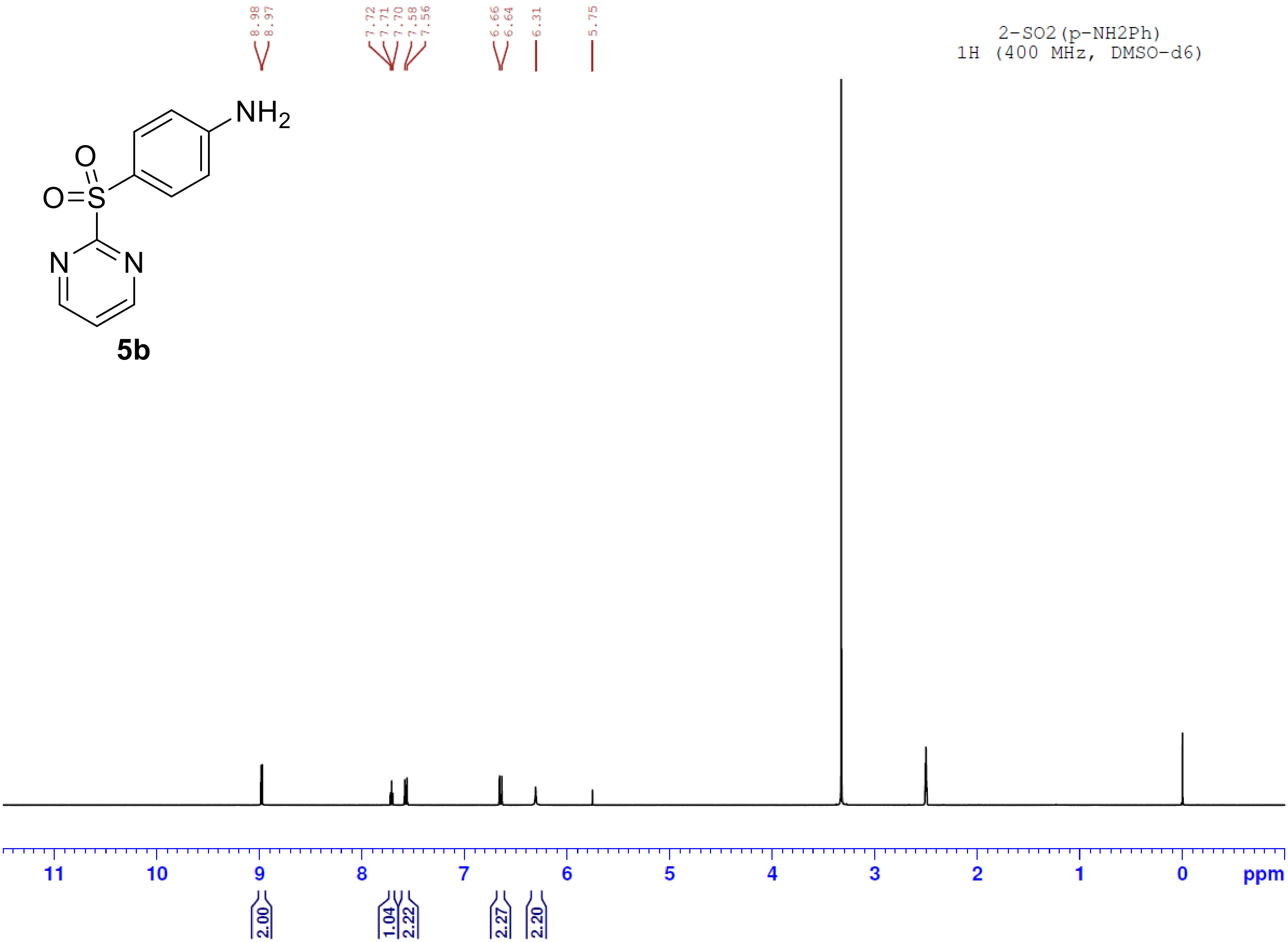
2-SO2(p-NH2Ph)
1H (400 MHz, DMSO-d6)
NH2
O
O=S
N
N
5b
8.98
8.97
7.72
7.71
7.70
7.58
7.56
6.66
6.64
6.31
5.75
11
10
9
8
7
6
5
4
3
2
1
0
ppm
2.00
1.04
2.22
2.27
2.20

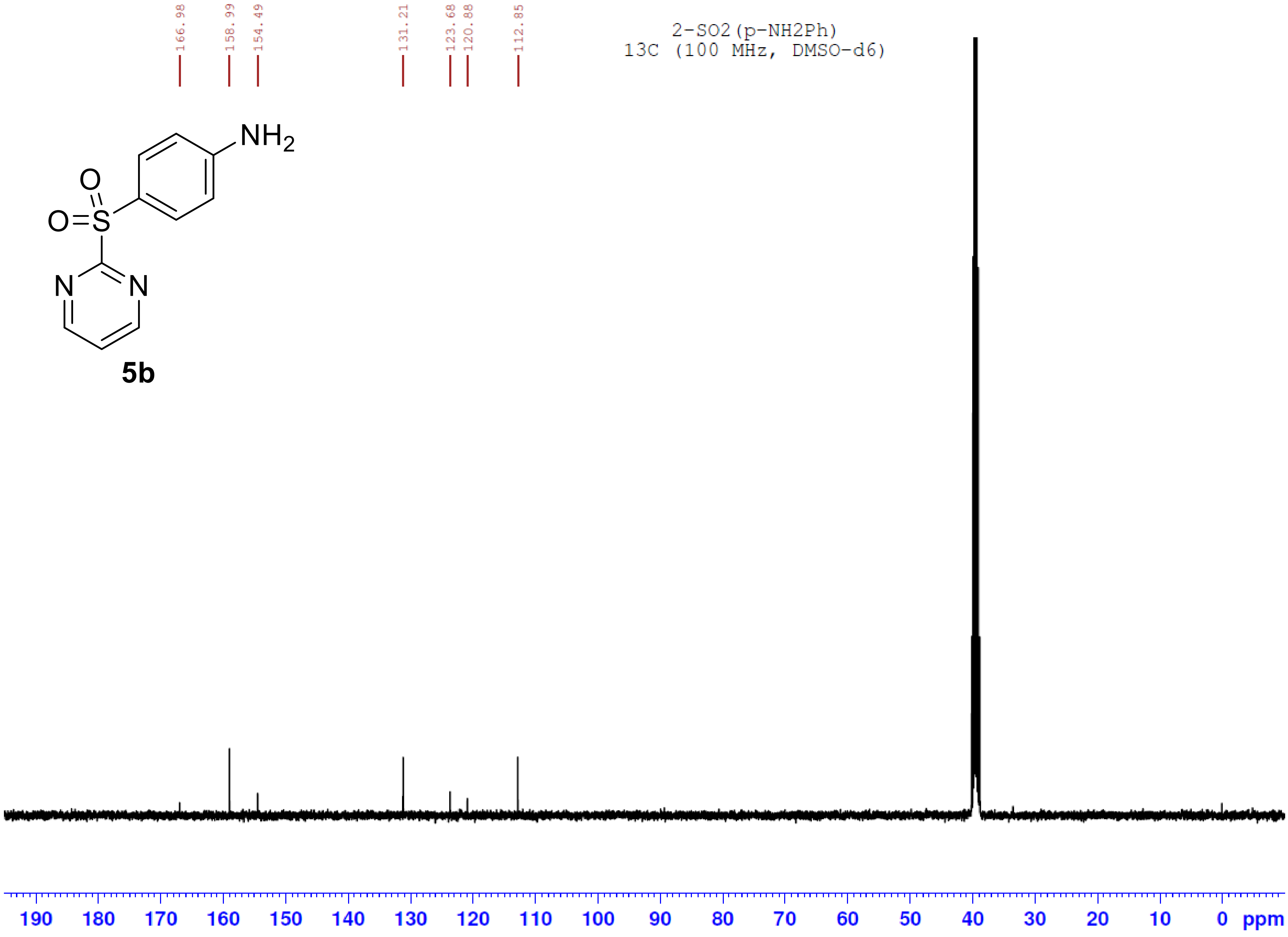
166.98
158.99
154.49
131.21
123.68
120.88
112.85
2-SO2(p-NH2Ph)
13C (100 MHz, DMSO-d6)
NH2
O
O=S
N
N
5b
190 180 170 160 150 140 130 120 110 100 90 80 70 60 50 40 30 20 10 0 ppm

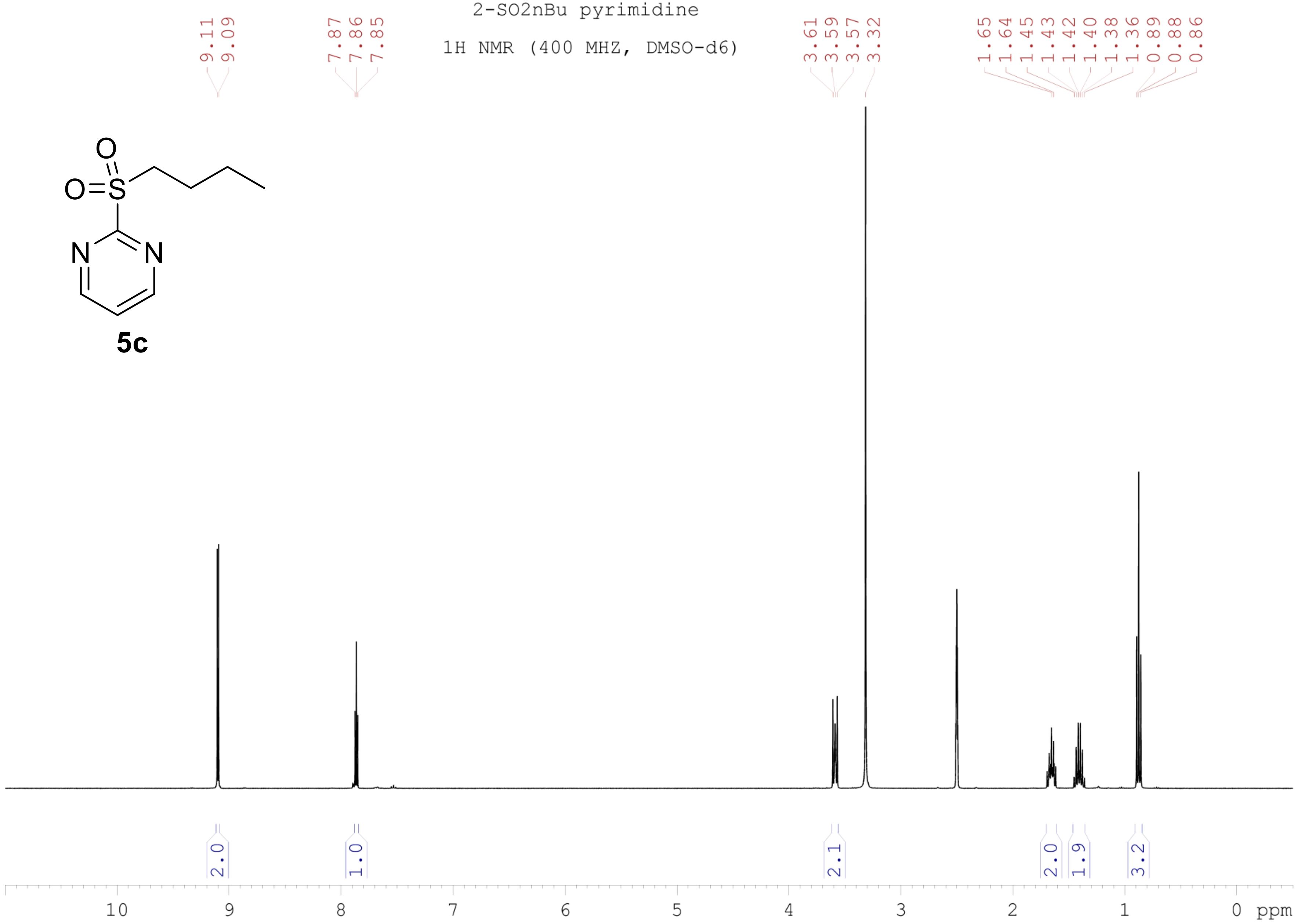

2-SO2nBu pyrimidine
1H NMR (400 MHZ, DMSO-d6)
9.11
9.09
7.87
7.86
7.85
3.61
3.59
3.57
3.32
1.65
1.64
1.45
1.43
1.42
1.40
1.38
1.36
0.89
0.88
0.86
O
O=S
N
N
5c
2.0
1.0
2.1
2.0
1.9
3.2
10
9
8
7
6
5
4
3
2
1
0 ppm

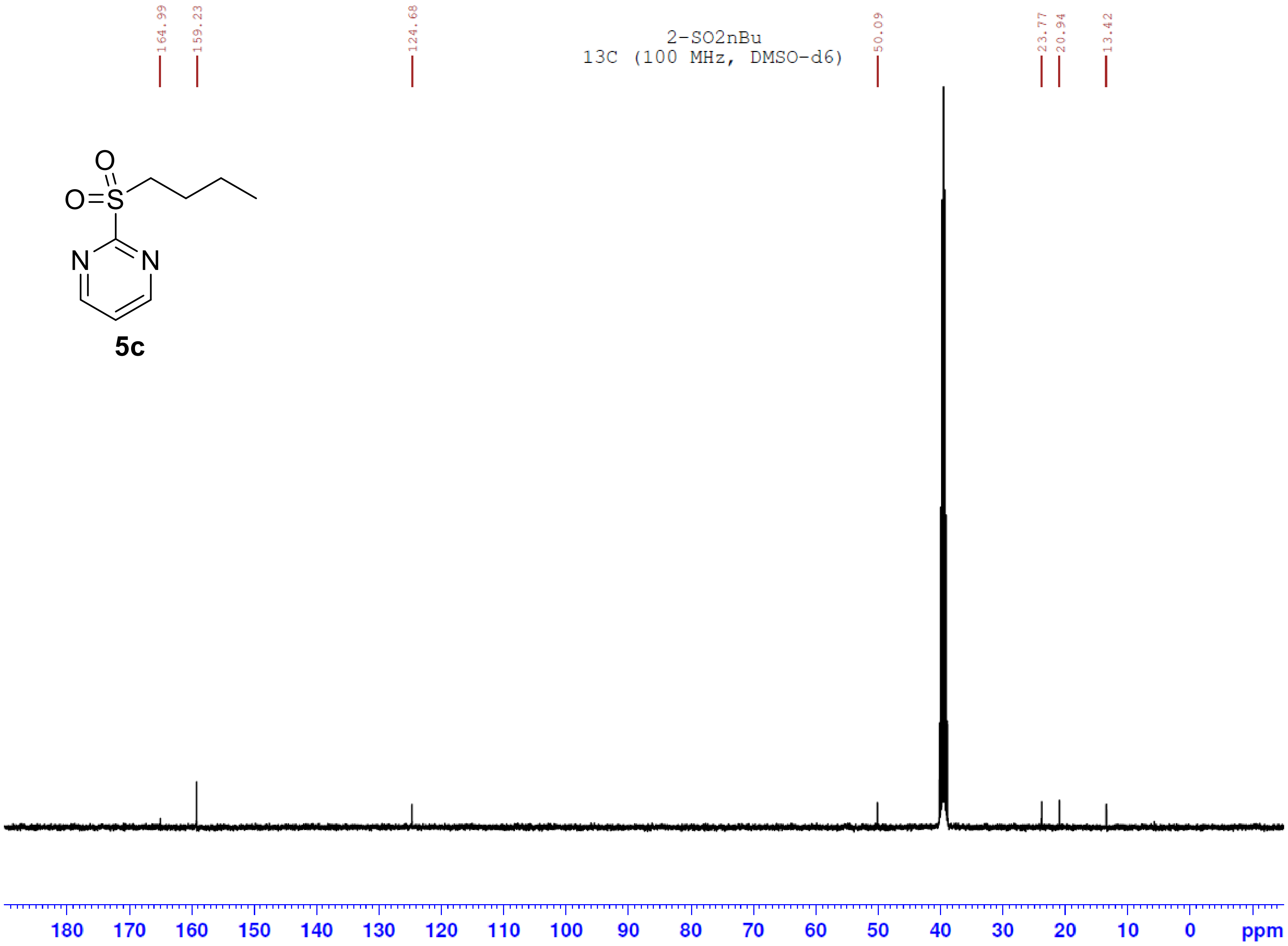
5c
164.99
159.23
124.68
2-SO2nBu
13C (100 MHz, DMSO-d6)
50.09
23.77
20.94
13.42
180
170
160
150
140
130
120
110
100
90
80
70
60
50
40
30
20
10
0
ppm

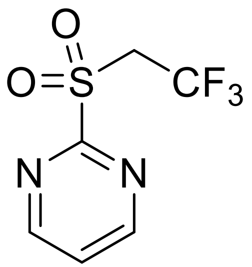

5e

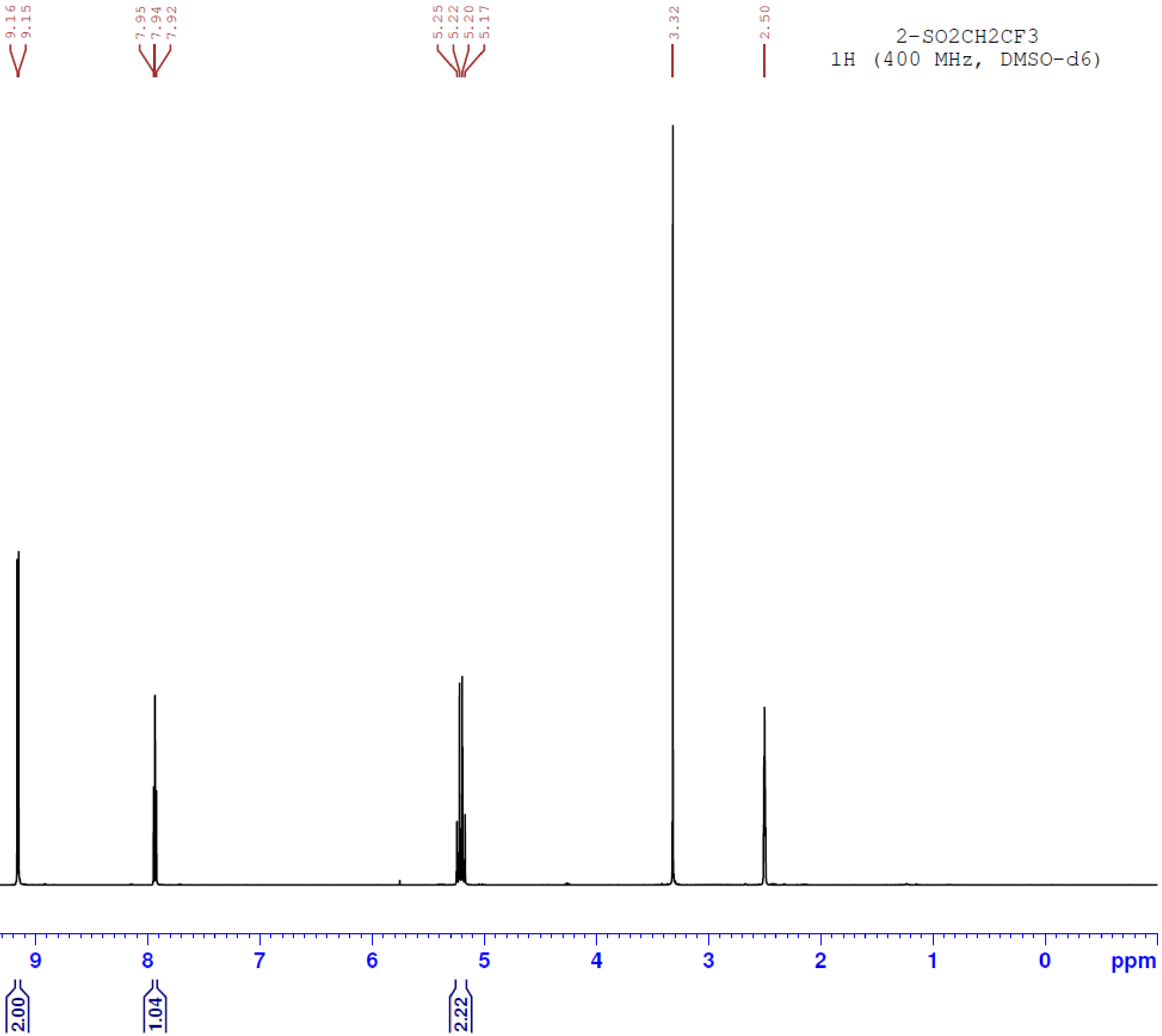

9.16
9.15
7.95
7.94
7.92
5.25
5.22
5.20
5.17
3.32
2.50
2-SO2CH2CF3
1H (400 MHz, DMSO-d6)
11
10
9
8
7
6
5
4
3
2
1
0
ppm
2.00
1.04
2.22

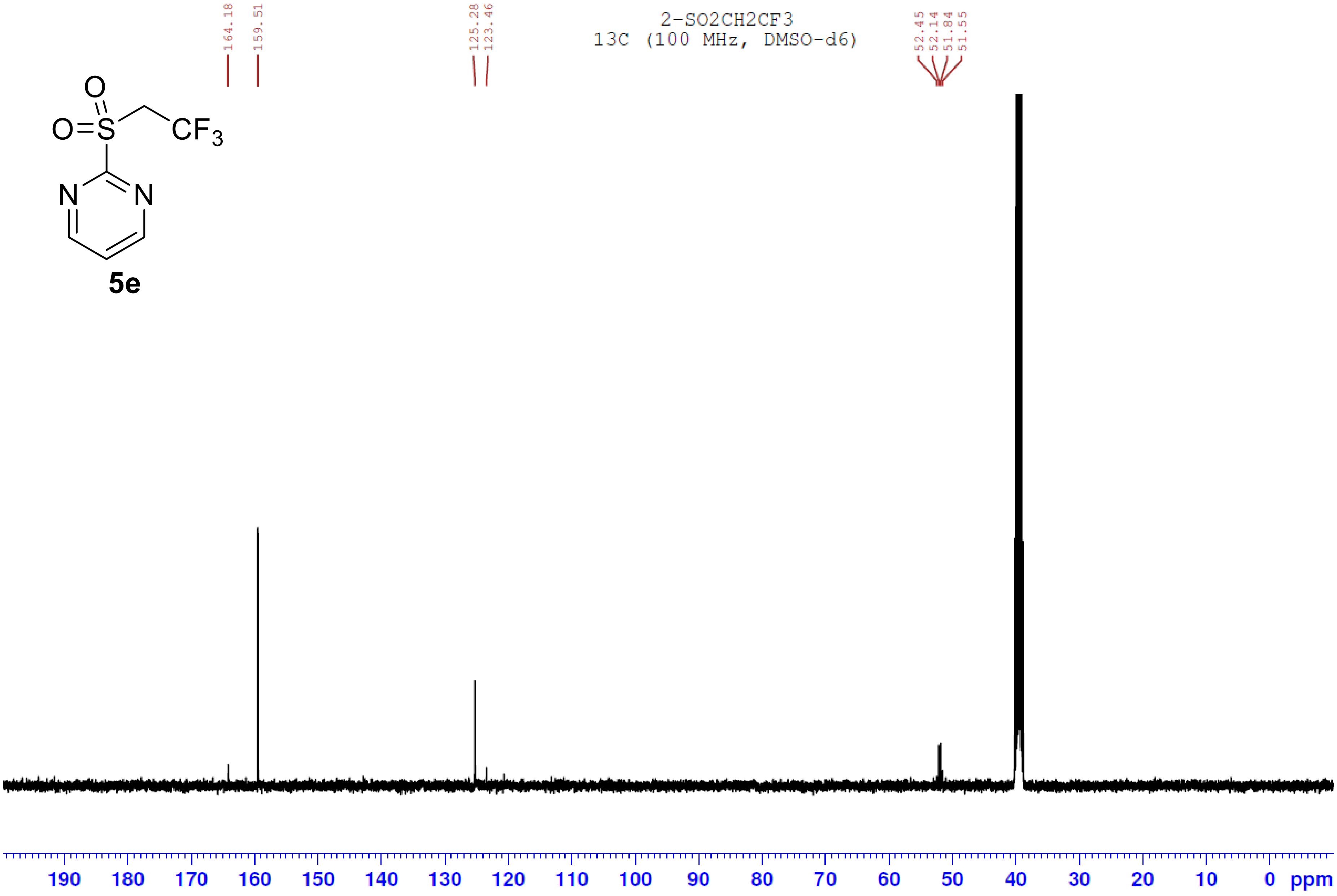
2-SO2CH2CF3
13C (100 MHz, DMSO-d6)
164.18
159.51
125.28
123.46
52.45
52.14
51.84
51.55
O=S(=O)CH2CF3
N
N
5e
190 180 170 160 150 140 130 120 110 100 90 80 70 60 50 40 30 20 10 0 ppm

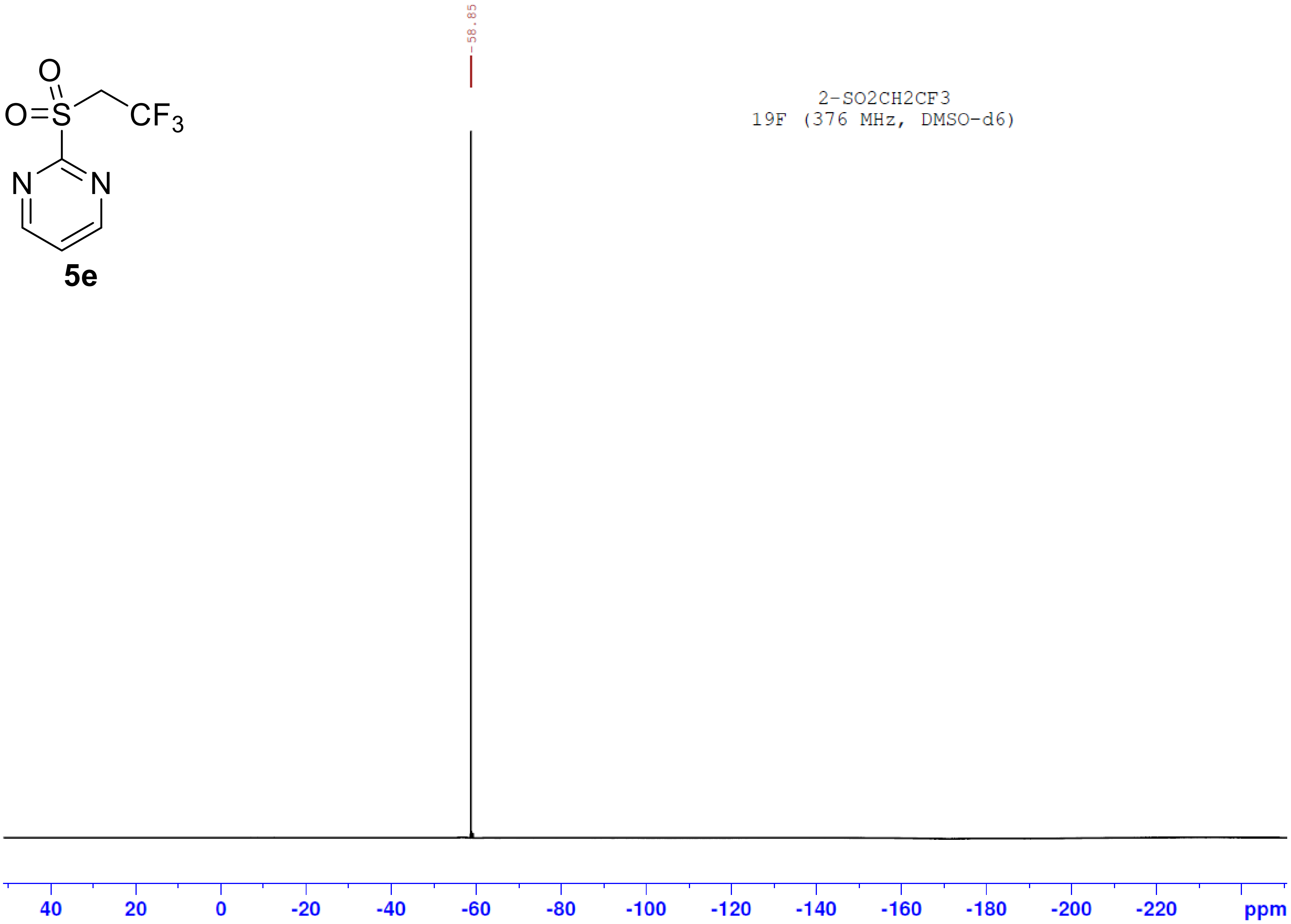

-58.85
O
O=S
CF3
N
N
5e
2-SO2CH2CF3
19F (376 MHz, DMSO-d6)
40
20
0
-20
-40
-60
-80
-100
-120
-140
-160
-180
-200
-220
ppm

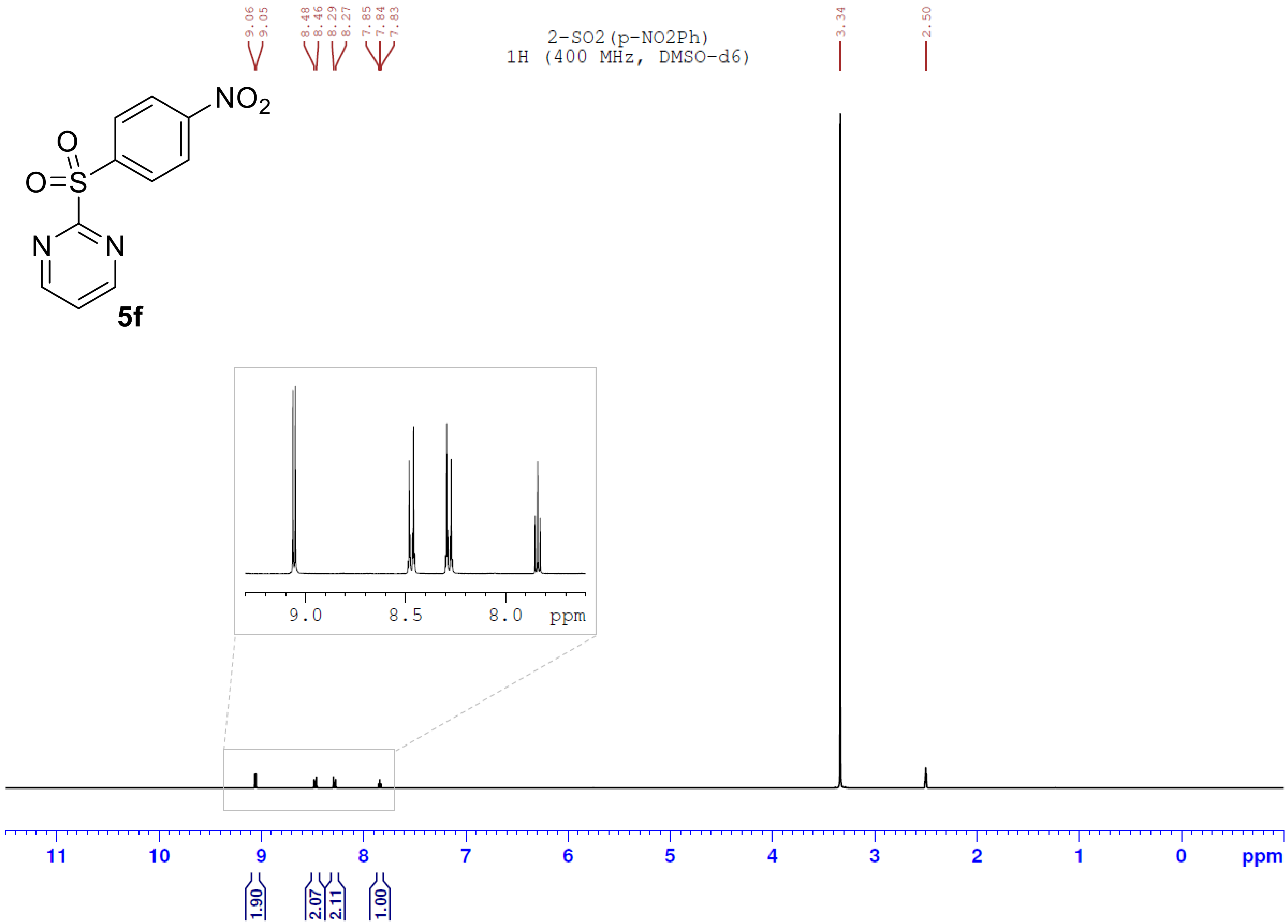
9.06
9.05
8.48
8.46
8.29
8.27
7.85
7.84
7.83
2-SO2(p-NO2Ph)
1H (400 MHz, DMSO-d6)
3.34
2.50
NO2
O
O=S
N
N
5f
9.0
8.5
8.0
ppm
11
10
9
8
7
6
5
4
3
2
1
0
ppm
1.90
2.07
2.11
1.00

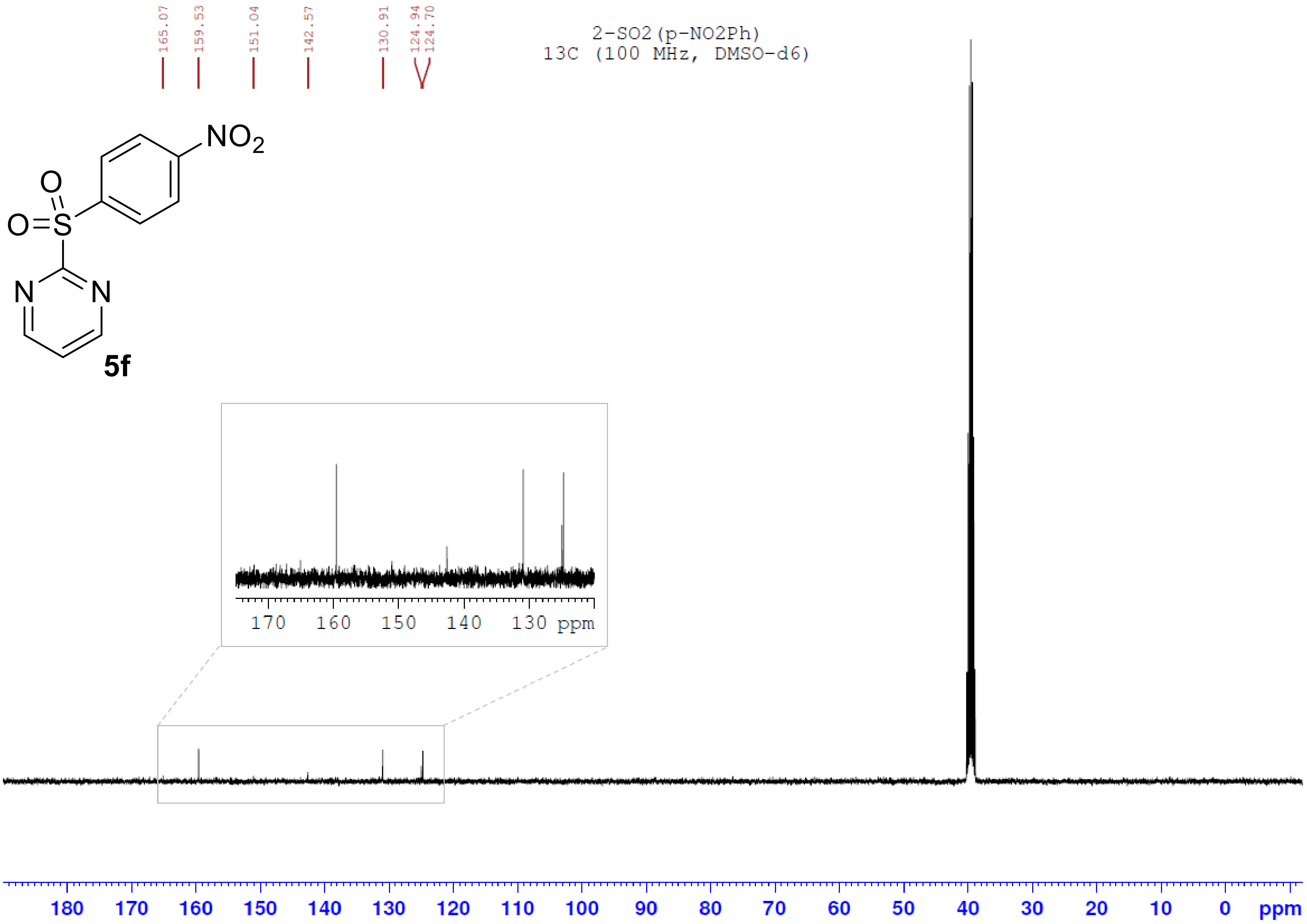
165.07
159.53
151.04
142.57
130.91
124.94
124.70
2-SO2(p-NO2Ph)
13C (100 MHz, DMSO-d6)
NO2
O
O=S
N
N
5f
170 160 150 140 130 ppm
180 170 160 150 140 130 120 110 100 90 80 70 60 50 40 30 20 10 0 ppm

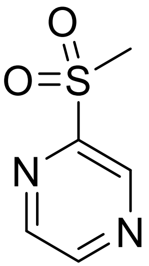

18

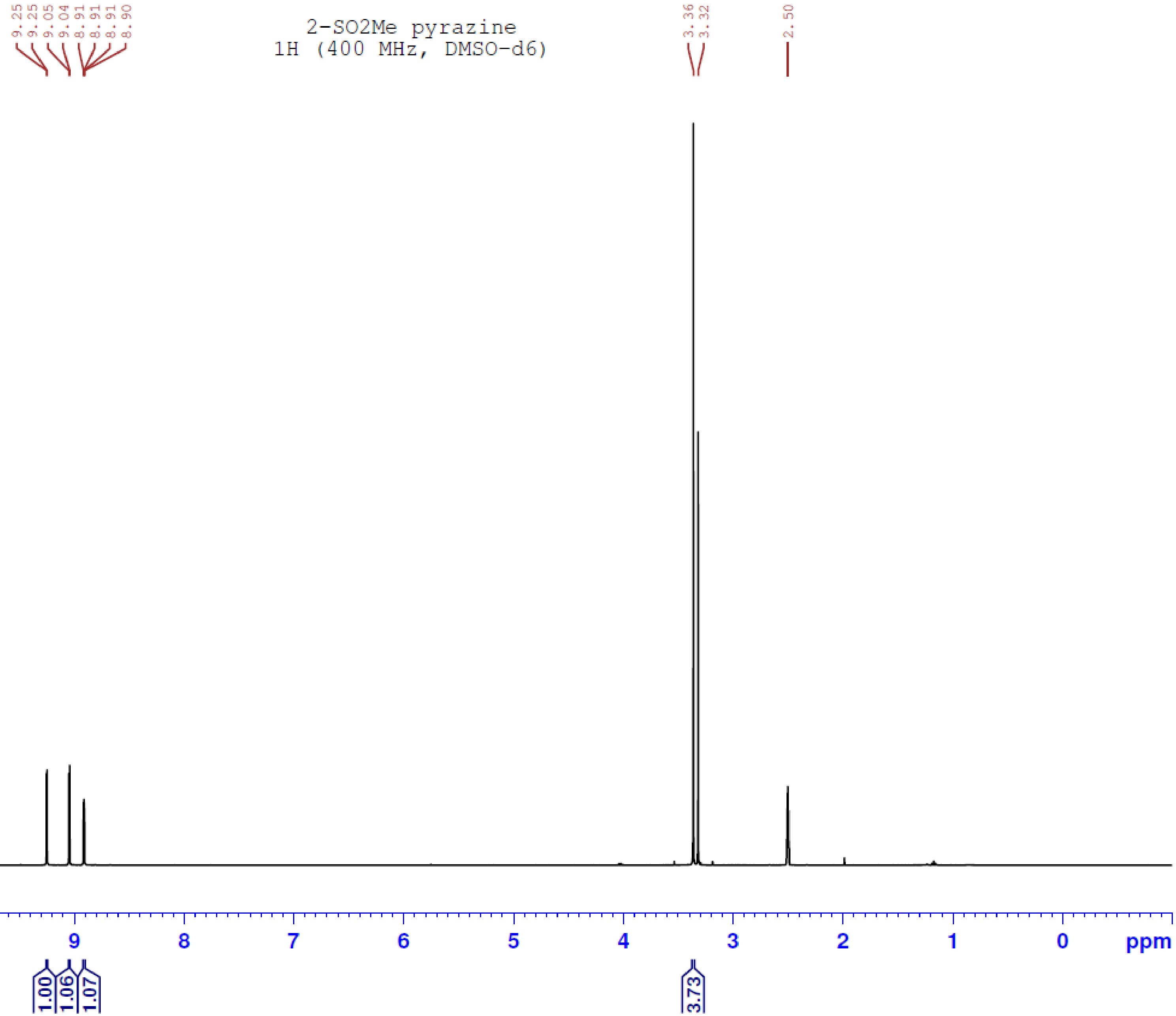

2-SO2Me pyrazine
1H (400 MHz, DMSO-d6)
9.25
9.25
9.05
9.04
8.91
8.91
8.91
8.90
3.36
3.32
2.50
11
10
9
8
7
6
5
4
3
2
1
0
ppm
1.00
1.06
1.07
3.73

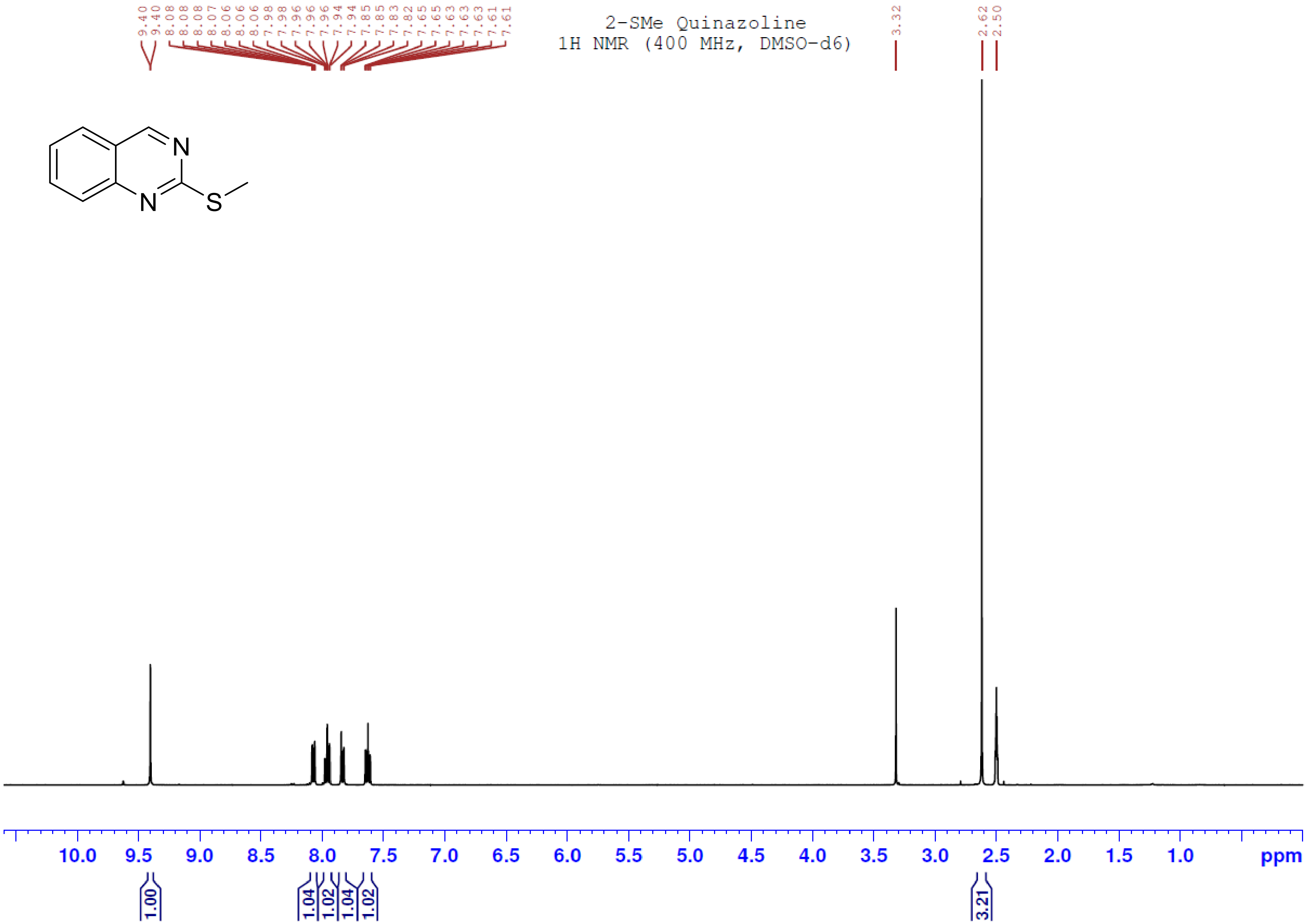
2-SMe Quinazoline
1H NMR (400 MHz, DMSO-d6)
9.40
9.40
8.08
8.08
8.07
8.06
8.06
8.06
7.98
7.98
7.96
7.96
7.96
7.94
7.94
7.85
7.85
7.83
7.82
7.65
7.65
7.63
7.63
7.63
7.61
7.61
3.32
2.62
2.50
10.0
9.5
9.0
8.5
8.0
7.5
7.0
6.5
6.0
5.5
5.0
4.5
4.0
3.5
3.0
2.5
2.0
1.5
1.0
ppm
1.00
1.04
1.02
1.04
1.02
3.21
N
N
S

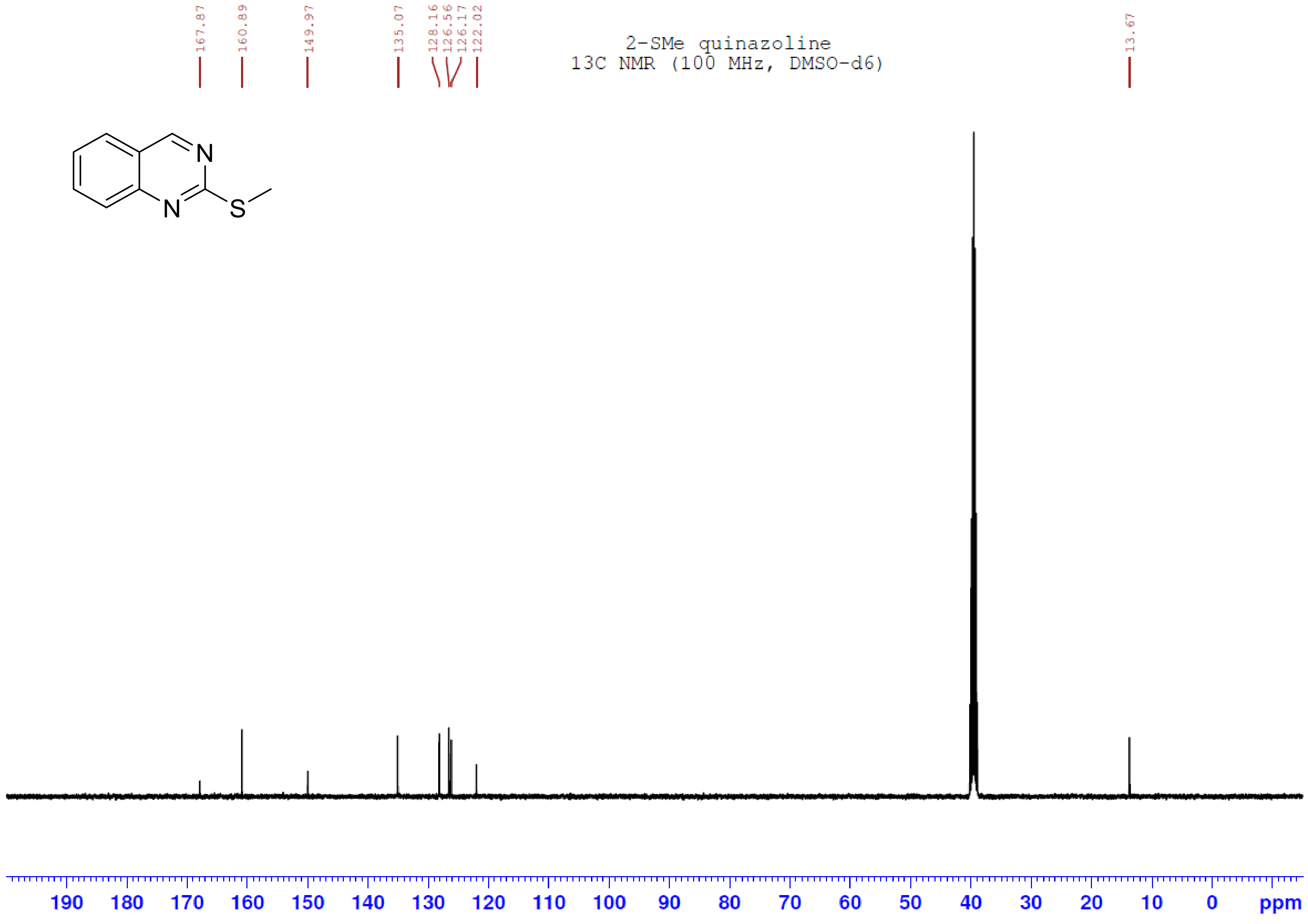

2-SMe quinazoline
13C NMR (100 MHz, DMSO-d6)
167.87
160.89
149.97
135.07
128.16
126.56
126.17
122.02
13.67
N
N
S
190
180
170
160
150
140
130
120
110
100
90
80
70
60
50
40
30
20
10
0
ppm

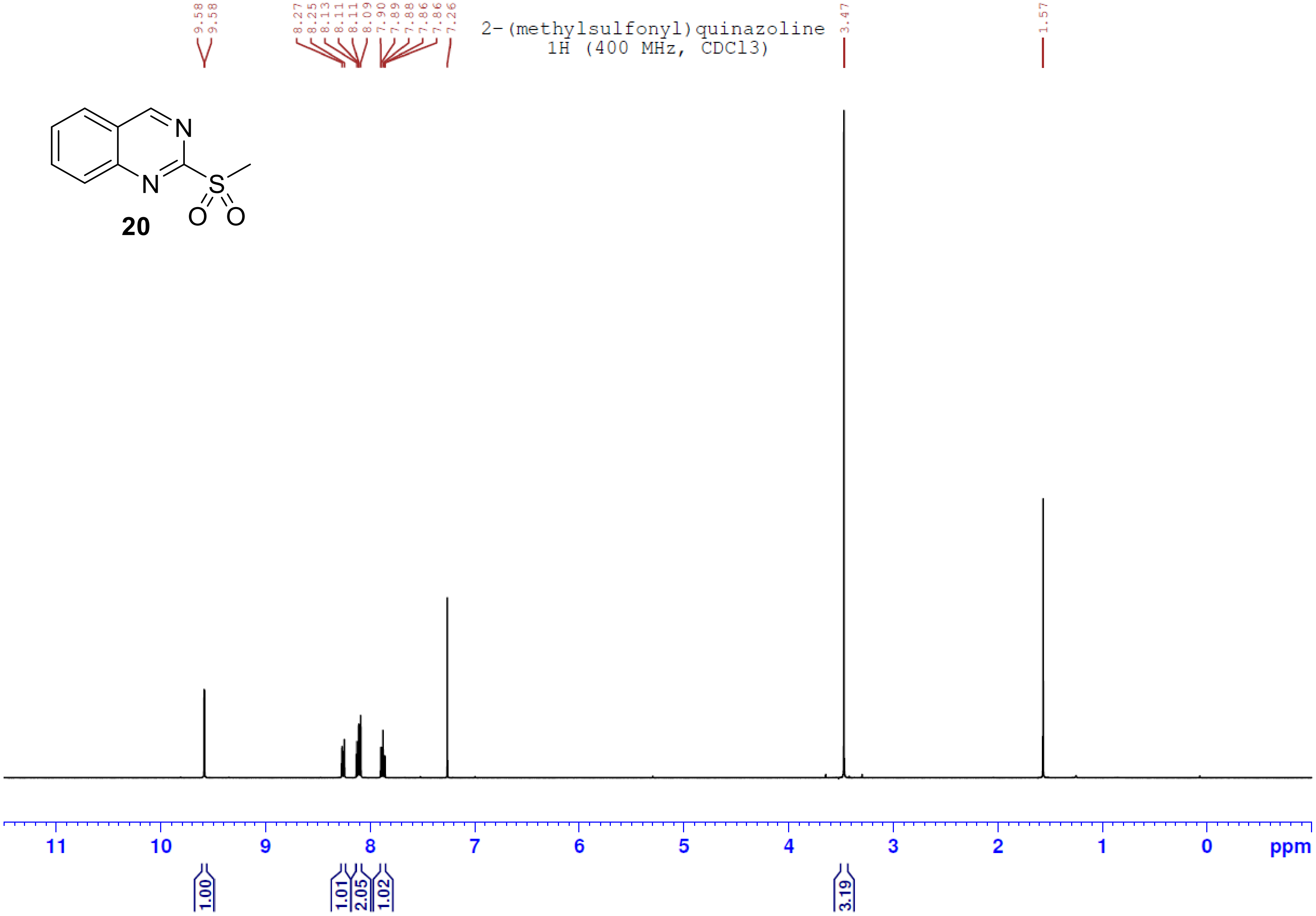

9.58
9.58
8.27
8.25
8.13
8.11
8.11
8.09
7.90
7.89
7.88
7.86
7.86
7.26
2-(methylsulfonyl)quinazoline
1H (400 MHz, CDCl3)
3.47
1.57
N
N
S
O
O
20
11
10
9
8
7
6
5
4
3
2
1
0
ppm
1.00
1.01
2.05
1.02
3.19
